# Ruehssiosides A–R, 18 Previously Undescribed Pregnane Glycosides from *Ruehssia caatingae*: Structural Elucidation, Potential Anti-Inflammatory Activity, and Insights into Structure–Activity Relationships

**DOI:** 10.1021/acsomega.6c02994

**Published:** 2026-09-16

**Authors:** Jociano da Silva Lins, Thalisson Amorim de Souza, Karoline Cristina Jatobá da Silva, Arthur Ribeiro de Souza, Cristiane Flora Villarreal, Lucas Silva Abreu, Fernando Martins dos Santos Junior, José Iranildo Miranda de Melo, Marcus Tullius Scotti, Marcelo Sobral da Silva, Josean Fechine Tavares, José Maria Barbosa Filho

**Affiliations:** † Health Sciences Center, Federal University of Paraiba, Program of Post-graduation on Natural and Synthetic Bioactive Products (PgPNSB) 58050-585, João Pessoa-PB, Brazil; ‡ Postgraduate Program in Ecology and Conservation, State University of Paraiba 58429-500, Campina Grande-PB, Brazil; § Department of Organic Chemistry, Fluminense Federal University 24020-141, Niteroi-RJ, Brazil; ∥ Faculty of Pharmacy, Federal University of Bahia 40170-115, Salvador-BA, Brazil

## Abstract

Nature has always inspired the discovery of novel compounds to treat human inflammatory processes, which are related to many diseases in acute and chronic conditions. Found in some Apocynaceae species, pregnane glycosides (PGs) have been attracting attention due to their promising anti-inflammatory potential. *Ruehssia* is a neotropical genus endemic to arid regions and, in Brazil, the Caatinga serves as a major hotspot for its occurrence. However, no in-depth chemical or pharmacological investigations have yet been conducted on *Ruehssia* species. Based on this, the present work sought to isolate, characterize, and investigate the potential anti-inflammatory activity of PGs obtained from the roots of *R. caatingae*. To achieve this goal, classical and instrumental chromatographic techniques were applied, followed by 1D and 2D NMR experiments, as well as IR and HR-ESI-MS analyses to assist structural elucidation. To assess potential anti-inflammatory activity, murine macrophage-like J774.A1 cells, stimulated with lipopolysaccharide (LPS) and interferon-gamma (IFN-γ), were used. As a result, 18 previously undescribed PGs were obtained, named ruehssiosides A–R (1–18). Among them, ruehssioside J (compound 10) stood out as the most potent of the series, exhibiting a half-maximal inhibitory concentration (IC_50_) of 17.5 μM for nitric oxide inhibition and significantly reducing IL-6 and IL-1β production. Additionally, structure–activity relationship (SAR) analysis was carried out, highlighting the structural elements contributing to the observed anti-inflammatory effects. This work encourages the continued study of Apocynaceae species, especially from underinvestigated environments, as sources of substances with anti-inflammatory potential.

## Graphical abstract

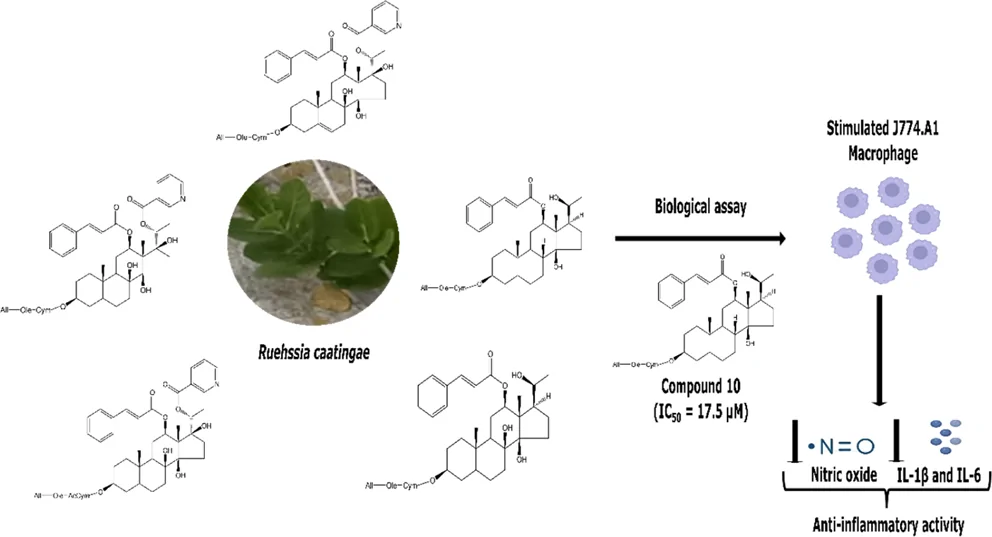

## 1 Introduction

Inflammation is a natural response of the organism to protect itself and recover from damage, infections, or other harmful stimuli, manifesting through the classical signs of redness, swelling, heat, and pain in the affected area. However, when the immune system fails to properly distinguish between endogenous and exogenous elements, or when it reacts excessively to external stimuli over a prolonged period, disorders such as cardiovascular and respiratory diseases, autoimmune conditions, neurodegenerative disorders, metabolic disturbances, and certain types of cancer may arise.^1–3^ The family Apocynaceae is among the most representative of the angiosperms, comprising more than 424 genera and approximately 4600 species, with a predominantly tropical and subtropical distribution worldwide.^4^ This feature reflects its remarkable chemodiversity, which has served as a source of bioactive molecules with anti-inflammatory and antitumor potential, particularly pregnane glycosides.^4^

Pregnane glycosides are amphiphilic compounds consisting of a hydrophobic moiety formed by the C21 pregnane steroid nucleus, which features a cyclopentanoperhydrophenanthrene (C17) skeleton with additional β-oriented methyl substituents at C-10 and C-13, as well as an ethyl group bound to C-17, and a hydrophilic moiety composed of sugar units in chains ranging from one to six. These sugars are usually linked at C-3 or C-20 of the aglycone through the ether bond.^5^ Pregnane glycosides are widely distributed in the plant kingdom, particularly in the subfamily Asclepiadoideae (Apocynaceae).^6^

Recent phylogenetic studies demonstrated that American species of *Marsdenia* form a monophyletic group distinct from the Asian taxon represented by *M. tinctoria*. Consequently, the genus *Ruehssia* H. Karst was reinstated to accommodate the American clade Marsdenieae marked by its morphological heterogeneity, *Ruehssia* comprises about 110 species, among which 44 occur in northeastern Brazil. It makes this region a center of diversity and endemism for this plant group, eight species are reported only in the Caatinga biome such as *R. caatingae*.^7^

In this context, our group has reported the isolation, characterization, and activity of saponins from different species in the semi-arid region of Brazil, and recently, we elucidated the structures of five previously undescribed pregnane glycosides from *Mandevilla dardanoi* (Apocynaceae), named dardanoids A–E.^8–10^ To the best of our knowledge, only a preliminary study has been carried out on the *Ruehssia* genus.^11^ Continuing the study of pregnane glycosides from Brazilian species of the Apocynaceae family, this work describes the isolation and structural elucidation of 18 previously undescribed C21 pregnane glycosides (Figure 1) from the roots of *R. caatingae*, as well as their potential anti-inflammatory effects.

**Figure 1:**
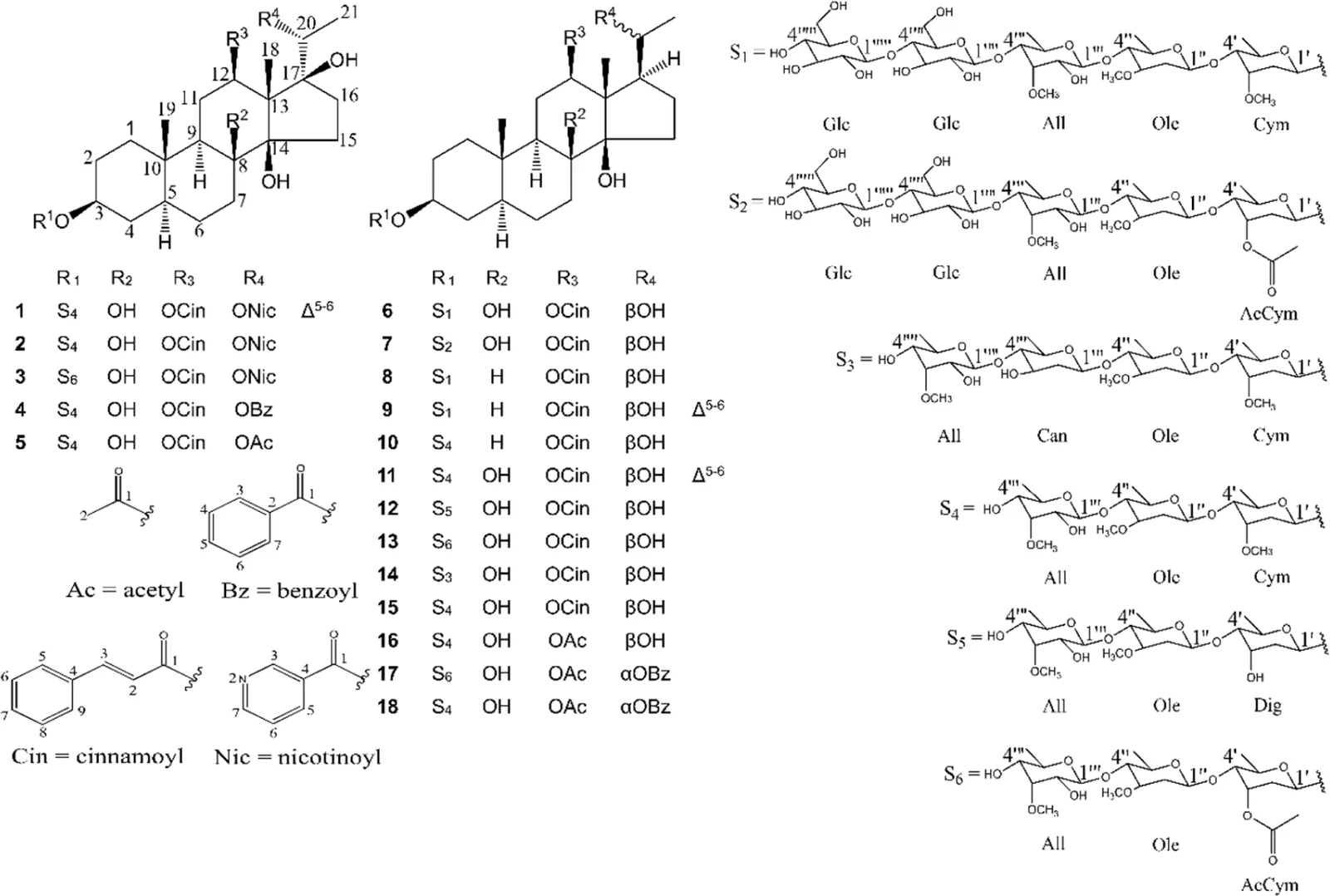
Chemical structures of **1**–**18** from the root of *R. caatingae* *.* Abbreviations shown below each sugar unit indicate the corresponding monosaccharides (Cym = d-cymarose, Ole = d-oleandrose, Dig = d-digitoxose, Can = d-canarose, Glc = d-glucose, All = 6-deoxy-3-*O*-methyl- d-allose; AcCym = *O*-acetyl-d-cymarose).

## 2 Results and Discussion

### 2.1 Isolation of Previously Undescribed Pregnane Glycosides

Phytochemical investigation of the chloroform phase obtained from the roots of *R. caatingae* led to the isolation of 18 previously undescribed pregnane glycosides through successive chromatographic procedures, including Sephadex LH-20, silica gel column chromatography, and preparative HPLC. The isolated compounds were subsequently evaluated for their potential anti-inflammatory effects in vitro.

### 2.2 Structure Elucidation of **1**–**18**

Compound **1** was obtained as white amorphous powder with an optical rotation of [α]_D_^20^ + 78.7 (c 0.10, MeOH). Its molecular formula was established as C_57_H_80_NO_18_ based on HR-ESI-MS [M + H]^+^ at *m*/*z* 1066.5348, calcd 1066.5370, Δ = 2.1 ppm, with the hydrogen deficiency index (HDI) equal to 19 (Figure S2). The IR spectrum showed absorptions corresponding to hydroxyl groups (3432 cm^–1^), conjugated ester carbonyl (1708 cm^–1^), aromatic ring bands (1640, 1447 cm^–1^), olefinic bond (1594 cm^–1^), and ether (1077 cm^–1^) (Figure S1). The presence of these functional groups was corroborated by the ^1^H NMR spectrum (Table 1 and Figure S3), which displayed three methyl signals at: δ_H_ 2.04 (*s*, CH_3_-18), 1.23 (*s*, CH_3_-19), and 1.50 (*d*, *J* = 6.5 Hz, CH_3_-21); an olefinic proton at: δ_H_ 5.28 (*dd*, *J* = 12.5, 4.0 Hz, H-6); oxymethine proton signals at: δ_H_ 3.78 (*m*, H-3), 5.25 (*dd*, J = 12.5; 4.0 Hz, H-12), 5.21 (*m*, H-20); a methine proton at: δ_H_ 1.70 (*d*, *J* = 3.0 Hz, H-9); and multiple methylene protons at δ_H_ 1.00 (*td*, *J* = 14.0, 4.0 Hz, H-1a), 1.65 (*m*, H-1b), 1.73 (*m*, H-2a), 2.00 (*m*, H-2b), 2.38 (*d*, *J* = 13.0 Hz, H-4a), 2.49 (*dd*, *J* = 13.5, 4.0 Hz, H-4b), 2.32 (*m*, H-7a), 2.43 (*br s*, H-7b), 1.96 (*m*, H-11a), 2.29 (*m*, H-11b), 2.08 (2H *m*, H-15), and 2.06 (2H, *m*, H-16).

**Table 1:** ^1^H NMR Data for the Aglycone Moieties of Compounds **1**–**6** (δ in ppm, *J* in Hz, C_5_D_5_N)

no	1^a^	2^a^	3^b^	4^a^	5^b^	6^b^
1	1.00 (td, 14.0; 4.0) 1.65 (*m*)	0.80 (*td*, 13.5; 3.5) 1.47 (*m*)	0.82 (*m*) 1.53 (*m*)	0.83 (*td*, 13.5; 4.0) 1.53 (*m*)	0.86 (*m*) 1.58 (*m*)	0.88 (*m*) 1.57 (*m*)
2	1.73 (*m*) 2.00 (*m*)	1.60 (*m*) 1.91 (*m*)	1.61 (*m*) 1.98 (*m*)	1.68 (*m*) 1.95 (*m*)	1.66 (*m*) 1.97 (*m*)	1.68 (*m*) 2.01 (*m*)
3	3.78 (*m*)	3.81 (*m*)	3.83 (*m*)	3.84 (*m*)	3.84 (*m*)	3.88 (*m*)
4	2.38 (*d*, 13.0) 2.49 (*dd*, 13.5; 4.0)	1.43 (*m*) 1.67 (br s)	1.43 (*m*) 1.69 (*d*, 7.6)	1.37 (*m*) 1.71 (*s*)	1.41 (*m*) 1.71 (*m*)	1.37 (*m*) 1.71 (*m*)
5	-	0.96 (*m*)	0.97 (*m*)	0.97 (*m*)	0.99 (*m*)	1.00 (*m*)
6	5.28 (*dd*, 12.5; 4.0)	1.09 (*m*) 1.72 (*m*)	1.11 (*s*) 1.76 (*s*)	1.10 (*m*) 1.77 (*m*)	1.10 (*m*) 1.76 (*m*)	1.06 (*m*) 1.80 (*m*)
7	2.32 (*m*) 2.43 (*br s*)	1.36 (*d*, 6.0) 2.13 (*m*)	1.36 (*t*, 6.4) 2.16 (*m*)	1.46 (*m*) 2.02 (*m*)	1.39 (*m*) 2.14 (*m*)	1.40 (*m*) 2.15 (*m*)
8	-	-	-	-	-	-
9	1.70 (*d*, 3.0)	1.33 (*br s*)	1.38 (*br s*)	1.36 (*br s*)	1.35 (*br s*)	1.38 (*d*, 4.0)
10	-	-	-	-	-	-
11	1.96 (*m*) 2.29 (*m*)	1.89 (*m*) 2.17 (*m*)	1.95 (*m*) 2.21 (*m*)	1.97 (*m*) 2.21 (*m*)	1.98 (*m*) 2.26 (*dd*, 10.0; 1.6)	1.84 (*m*) 2.25 (*m*)
12	5.25 (*dd*, 12.5; 4.0)	5.18 (*d*, 11.0)	5.18 (*d*, 8.0)	5.25 (*m*)	5.15 (*dd*, 11.2; 4.0)	5.09 (*dd*, 12.0; 4.4)
13	-	-	-	-	-	-
14	-	-	-	-	-	-
15	2.08 (*m*)	2.09 (*m*)	2.05 (*m*)	2.02 (*m*)	2.09 (*m*)	1.40 (*m*)
16	2.06 (*m*)	2.17 (*m*)	2.10 (*m*) 2.16 (*m*)	2.16 (*m*)	2.14 (*m*)	1.88 (*m*) 2.15 (*m*)
17	-	-	-	-	-	2.06 (*m*)
18	2.04 (*s*)	2.02 (*s*)	2.06 (*s*)	2.10 (*s*)	2.00 (*s*)	1.71 (*s*)
19	1.23 (*s*)	1.05 (*s*)	1.09 (*s*)	1.09 (*s*)	1.13 (*s*)	1.16 (*s*)
20	5.21 (*m*)	5.21 (*m*)	5.23 (*m*)	5.20 (*m*)	5.19 (*m*)	4.19 (*m*)
21	1.50 (*d*, 6.5)	1.50 (*d*, 6.0)	1.51 (*br s*)	1.50 (*d*, 6.0)	1.53 (*d*, 6.0)	1.14 (*br s*)
12-O	Cin	Cin	Cin	Cin	Cin	Cin
1	-	-	-	-	-	-
2	6.48 (*d*, 16.0)	6.46 (*d*, 16.0)	6.51 (*d*, 16.4)	6.46 (*d*, 16.0)	6.81 (*d*, 16.0)	6.89 (*d*, 16.0)
3	7.78 (*d*, 16.0)	7.74 (*d*, 16.0)	7.77 (*d*, 16.4)	7.81 (*d*, 16.0)	8.00 (*d*, 16.0)	8.03 (*d*, 16.0)
4	-	-	-	-	-	-
5	7.37 (*m*)	7.33 (*m*)	7.36 (*m*)	7.34 (*m*)	7.67 (*m*)	7.61 (*m*)
6	7.32 (*m*)	7.30 (*m*)	7.31 (*m*)	7.32 (*m*)	7.33 (*m*)	7.32 (*m*)
7	7.32 (*m*)	7.30 (*m*)	7.31 (*m*)	7.32 (*m*)	7.33 (*m*)	7.32 (*m*)
8	7.32 (*m*)	7.30 (*m*)	7.31 (*m*)	7.32 (*m*)	7.33 (*m*)	7.32 (*m*)
9	7.37 (*m*)	7.33 (*m*)	7.36 (*m*)	7.34 (*m*)	7.67 (*m*)	7.61 (*m*)
20-O	Nic	Nic	Nic	Bz	Ac	-
1	-	-	-	-	-	-
2	8.79 (*dd*, ;4.5; 1.5)	8.76 (*dd*, ;4.5; 1.5)	8.83 (*m*)	-	1.92 (*s*)	-
3	-	-	-	8.15 (*dd*, 8.5; 1.5)	-	-
4	8.29 (*dt*, ;8.0; 2.0)	8.26 (*dt*, ;8.0; 2.0)	8.34 (*d*, 6.0)	7.28 (*m*)	-	-
5	7.19 (*d*, 4.5)	7.16 (*dd*, 8.0; 4.5)	7.14 (*d*, 8.0)	7.47 (*tt*, 7.5; 1.5)	-	-
6	9.48 (*d*, 2.0)	9.45 (*d*, 2.0)	9.55 (*m*)	7.28 (*m*)	-	-
7	-	-	-	8.15 (*dd*, 8.5; 1.5)	-	-

a Measured at 500 MHz.

b Measured at 400 MHz: Cin = cinnamoyl; Nic = nicotinoyl; Bz = benzoyl; Ac = acetyl.

Additionally, the ^13^C NMR spectrum (Figures S4, S5 and Table 2) and HSQC (Figure S6) correlations showed three methyl carbons at: δ_C_ 11.2 (C-18), 17.7 (C-19), and 15.1 (C-21); two trisubstituted olefinic carbons at: δ_C_ 138.9 (C-5) and 119.1 (C-6); six oxygenated carbons at: δ_C_ 77.3 (C-3), 74.0 (C-8), 74.3 (C-12), 88.7 (C-14), 87.1 (C-17), and 76.1 (C-20); two tertiary carbons at: δ_C_ 43.7 (C-9), 56.8 (C-13) and secondary carbons at: δ_C_ 38.4 (C-1), 29.5 (C-2), 38.9 (C-4), 34.5 (C-7), 25.4 (C-11), and 33.4 (C-15). These findings indicated that compound 1 is a steroidal C21 pregnane-type. The aglycone was identified as sarcostin based on NMR analysis and comparison with the reported data.^12,13^

**Table 2:** ^13^C NMR Data for the Aglycone Moieties of Compounds **1**–**18** (δ in ppm, C_5_D_5_N)

no	1^a^	2^a^	3^b^	4^a^	5^b^	6^b^	7^b^	8^b^	9^b^	10^b^	11b	12b	13b	14b	15a	16a	17a	18b
1	38.4	37.5	37.6	37.7	37.7	37.9	38.0	37.4	37.1	37.4	38.9	38.0	38.0	38.1	37.8	38.0	37.9	38.0
2	29.5	29.1	29.3	29.4	29.3	29.4	29.5	29.8	29.9	29.9	29.9	29.5	29.5	29.5	29.3	29.5	29.3	29.5
3	77.3	76.4	76.8	76.1	76.5	76.5	76.9	76.4	77.0	76.5	77.8	76.6	76.9	76.6	76.5	76.6	76.9	76.7
4	38.9	34.0	34.1	34.2	34.3	34.3	34.4	34.6	38.9	34.7	39.2	34.4	34.4	34.4	34.2	34.4	34.3	34.4
5	138.9	44.8	45.0	45.0	45.0	45.2	45.4	44.3	139.2	44.4	138.9	45.3	45.4	45.4	45.1	45.3	45.1	45.2
6	119.1	24.7	24.4	25.0	24.9	25.2	25.3	28.8	122.2	28.9	119.7	25.2	25.3	25.3	25.1	25.3	25.0	25.2
7	34.5	34.1	34.2	34.3	34.1	35.1	35.2	27.9	27.5	28.0	36.1	35.2	35.2	35.2	35.0	35.4	35.4	35.5
8	74.0	75.7	75.8	75.9	75.8	75.6	75.7	40.0	36.5	40.1	73.9	75.7	75.7	75.7	75.5	75.6	76.0	76.1
9	43.7	46.4	46.5	46.6	46.6	47.7	47.8	46.3	43.4	46.5	45.0	47.8	47.8	47.8	47.6	47.7	48.0	48.1
10	36.9	36.0	36.2	36.2	36.2	36.5	36.6	35.8	35.7	35.9	37.1	36.5	35.9	36.6	36.4	36.5	36.4	36.5
11	25.4	24.2	24.9	24.4	24.5	23.8	23.9	26.7	26.5	26.8	24.8	23.9	23.9	23.9	23.7	23.7	23.6	23.7
12	74.3	74.8	74.8	75.9	74.5	78.4	78.4	77.9	77.4	78.0	77.8	78.4	78.4	78.5	78.3	78.2	81.0	81.1
13	56.8	57.1	57.2	57.3	57.1	53.8	53.9	53.0	52.8	53.1	53.7	53.8	53.9	53.9	53.7	53.6	52.6	52.7
14	88.7	88.5	88.5	88.6	88.5	85.3	85.4	83.9	84.1	83.9	85.5	85.4	85.4	85.4	85.2	85.3	86.0	86.2
15	33.4	33.7	33.1	33.8	33.4	35.3	35.5	32.9	33.4	33.0	36.0	35.4	35.5	35.5	35.3	35.1	34.0	34.1
16	33.7	33.1	33.7	33.4	33.3	19.0	19.1	18.8	18.5	19.0	33.6	19.1	19.1	19.1	18.9	19.1	23.5	23.6
17	87.1	87.2	87.3	87.6	87.3	51.8	51.8	51.6	51.7	51.8	52.0	51.8	51.9	51.9	51.7	51.7	52.2	52.3
18	11.2	11.6	11.7	11.8	11.7	12.1	12.2	9.8	9.6	9.9	11.8	12.1	12.1	12.2	12.0	12.0	11.1	11.2
19	17.7	12.5	12.6	12.7	12.7	12.9	13.0	11.9	19.2	12.0	18.2	13.0	13.0	13.1	12.9	13.0	13.1	13.2
20	76.1	76.0	76.1	75.6	75.0	65.4	65.5	65.2	65.1	65.3	65.4	65.5	65.3	65.5	65.3	65.4	71.7	71.9
21	15.1	15.1	15.2	15.3	15.1	22.2	22.3	22.5	22.4	22.7	22.3	22.3	22.3	22.3	22.1	22.3	19.8	20.0
12-O	Cin	Cin	Cin	Cin	Cin	Cin	Cin	Cin	Cin	Cin	Cin	Cin	Cin	Cin	Cin	Ac	Ac	Ac
1	166.5	166.4	166.5	166.7	166.7	167.0	167.1	166.9	166.9	167.0	167.1	167.0	167.0	167.1	167.0	-	-	-
2	119.8	119.8	119.9	120.2	120.4	119.3	119.4	119.2	119.1	119.3	119.4	119.4	119.4	119.4	119.1	-	-	-
3	143.8	143.7	143.8	143.7	143.7	144.8	144.9	144.9	144.9	144.9	144.9	144.9	144.9	144.9	144.8	-	-	-
4	134.5	134.4	134.5	134.8	134.9	134.9	134.9	134.7	135.0	134.9	134.9	135.0	134.9	134.9	134.6	171.0	171.0	171.0
5	128.2	128.2	128.3	128.4	128.4	128.4	128.5	128.5	128.4	128.6	128.6	128.5	128.5	128.5	128.4	21.1	21.0	21.1
6	129.0	128.9	129.0	129.0	129.2	129.1	129.2	129.1	129.1	129.2	129.2	129.2	129.2	129.2	129.1	-	-	-
7	130.4	130.3	130.4	130.3	130.3	130.5	130.6	130.5	130.5	130.6	130.6	130.5	130.5	130.1	130.4	-	-	-
8	129.0	128.9	129.0	129.0	129.2	129.1	129.2	129.1	129.1	129.2	129.2	129.2	129.2	129.2	129.1	-	-	-
9	128.2	128.2	128.3	128.4	128.4	128.4	128.5	128.5	128.4	128.6	128.6	128.5	128.5	128.5	128.4	-	-	-
20-O	Nic	Nic	Nic	Bz	Ac	-	-	-	-	-	-	-	-	-	-	-	Bz	Bz
1	164.4	164.3	164.4	165.5	170.5	-	-	-	-	-	-	-	-	-	-	-	166.0	166.1
2	-	-	-	131.1	21.3	-	-	-	-	-	-	-	-	-	-	-	131.4	131.6
3	151.1	151.0	151.1	130.1	-	-	-	-	-	-	-	-	-	-	-	-	130.0	130.1
4	126.6	126.5	126.6	128.6	-	-	-	-	-	-	-	-	-	-	-	-	128.5	128.6
5	137.2	137.1	137.2	133.1	-	-	-	-	-	-	-	-	-	-	-	-	132.9	133.0
6	123.6	123.6	122.9	128.6	-	-	-	-	-	-	-	-	-	-	-	-	128.5	128.6
7	153.5	153.4	153.6	130.1	-	-	-	-	-	-	-	-	-	-	-	-	130.0	130.1

a Measured at 125 MHz.

b Measured at 100 MHz: Cin = cinnamoyl; Nic = nicotinoyl; Bz = benzoyl; Ac = acetyl.

Proton signals at: δ_H_ 6.48 (*d*, *J* = 16.0 Hz, Cin-H-2), 7.78 (*d*, *J* = 16.0 Hz, Cin-H-3), 7.37 (*m*, Cin-H-5,9), 7.32 (*m*, Cin-H-6,7,8), 8.79 (*dd*, *J* = 4.5, 1.5 Hz, Nic-H-2), 8.29 (*dt*, *J* = 8.0, 2.0 Hz, Nic-H-4), 7.19 (*d*, *J* = 4.5 Hz, Nic-H-5), and 9.48 (*d*, *J* = 2.0 Hz, Nic-H-6), together with carbon signals at: δ_C_ 166.5 (Cin-C-1), 119.8 (Cin-C-2), 143.8 (Cin-C-3), 134.5 (Cin-C-4), 128.2 (Cin-C-5,9), 129.0 (Cin-C-6,8), 130.4 (Cin-C-7), 164.4 (Nic-C-1), 151.1 (Nic-C-3), 126.6 (Nic-C-4), 137.2 (Nic-C-5), 123.6 (Nic-C-6), and 153.5 (Nic-C-7), confirmed the presence of cinnamoyl and nicotinoyl groups.^14^ These substituents were assigned to C-12 and C-20, respectively, based on HMBC correlations (Figures 2 and S7) between δ_H_ 5.25 (H-12) and δ_C_ 166.5 (Cin-C-1) and between δ_H_ 5.19 (H-20) and δ_C_ 164.4 (Nic-C-1).^14,15^

**Figure 2:**
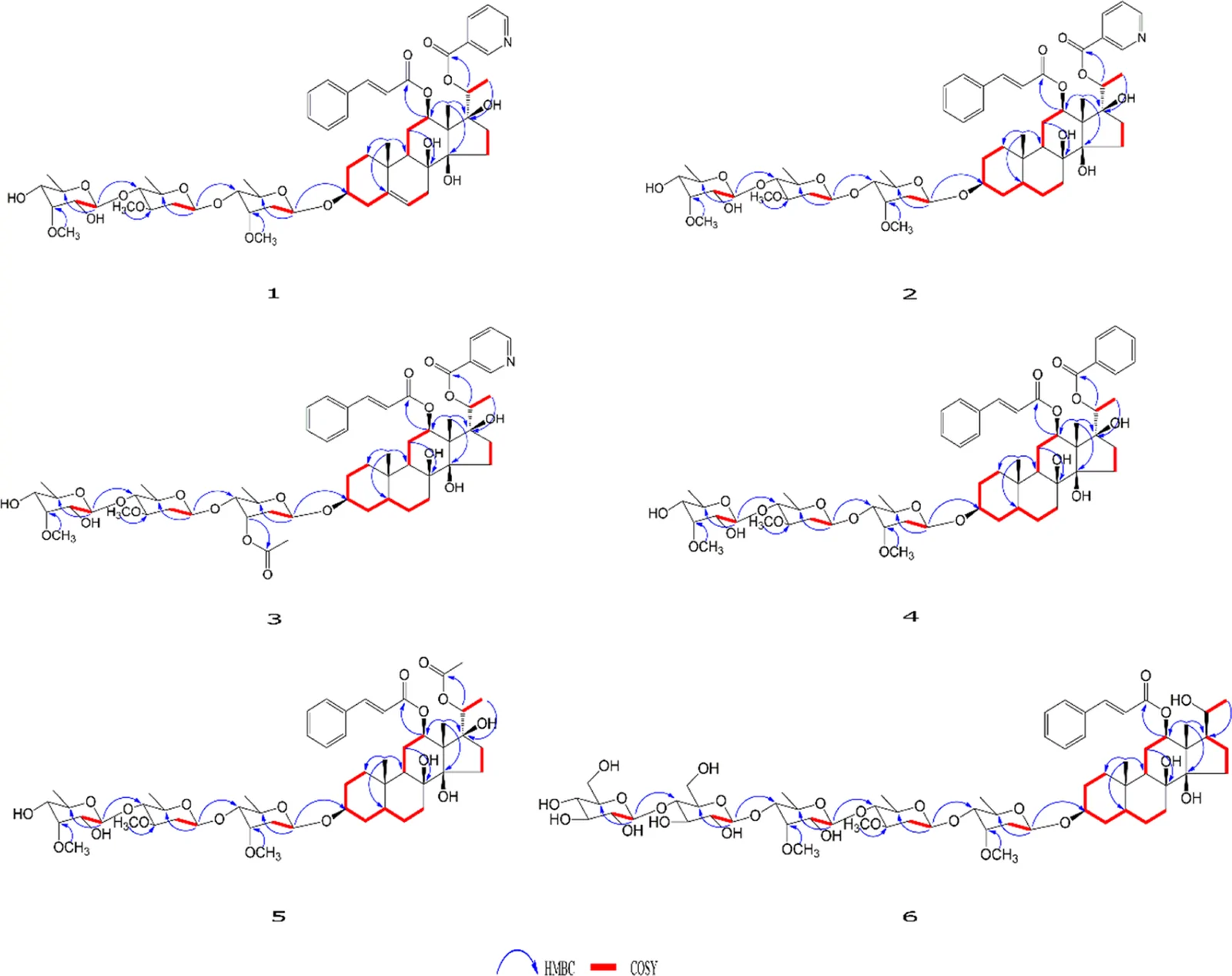
Key ^1^H–^13^C HMBC and ^1^H–^1^H COSY correlations of compounds **1**–**6**

The relative configuration of compound 1 was deduced from its NOESY spectrum (Figure 3 and S10), showing correlations between H-3/H-1b, H-1b/H-9, H-9/H-12, H-19/H-2a, H-19/H-11b, H-18/H-11b, H-18/H-20, and H-21/H-16. These correlations indicated that the configurations at H-3, H-1b, H-2a, H-9, H-12, and H-11b were assigned as *α*-oriented, while H-20 was assigned as *β*-oriented. The *β*-orientations of the hydroxyl group at C-17 and the methyl groups at C-18 and C-19 were assigned by comparison of the aglycone NMR data with those of previously reported sarcostin-derived pregnane glycosides of established stereochemistry. Literature data report that the chemical shifts of C-20 and C-16 in 20*R*-configured 20-hydroxy-C/D-cis-pregnane steroids are δ_C_ 75.8 and 39.0 ppm, respectively, which are consistent with the values observed for compound **1**, as C-20 and C-16 resonated at δ_C_ 76.1 and 33.7, respectively.^16,17^

**Figure 3:**
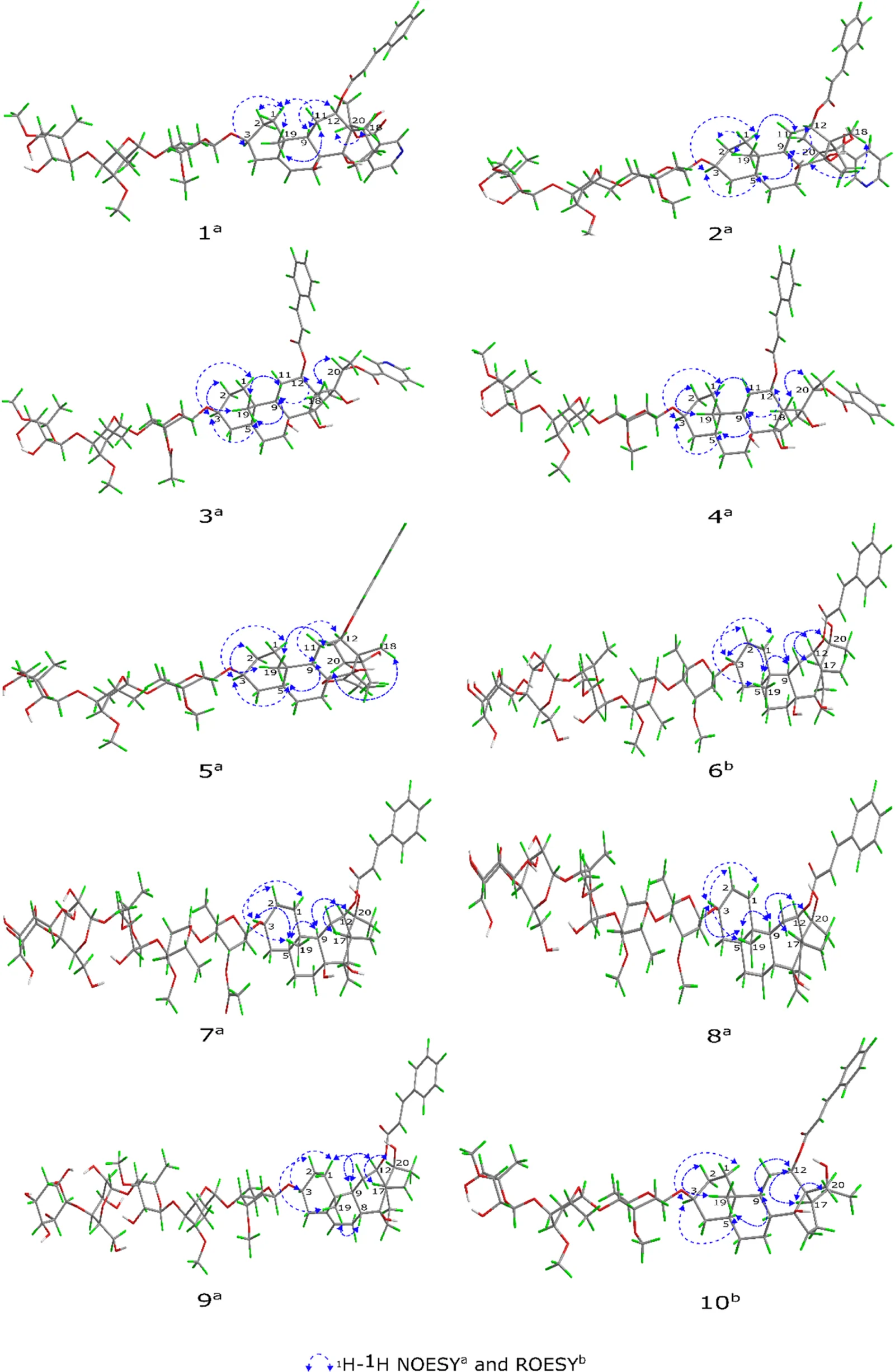
Key ^1^H–^1^H NOESY^a^ and ^1^H–^1^H ROESY^b^ correlations of compounds **1**–**10**. Superscripts a and b identify the compounds for which the corresponding ^1^H–^1^H NOESY and ^1^H–^1^H ROESY experiments were acquired.

In the ^1^H NMR spectrum (Table 3), three anomeric protons were observed at: δ_H_ 5.19 (*br s*, H-1′), 4.65 (*dd*, *J* = 9.5, 1.5 Hz, H-1″), and 5.24 (*d*, *J* = 8.0 Hz, H-1‴), which in the HSQC spectrum corresponded to carbons at: δ_C_ 96.1 (C-1′), 101.6 (C-1″), and 101.7 (C-1‴), respectively. Additional signals included methoxy groups at: δ_H_ 3.50 (*s*), 3.47 (*s*), and 3.79 (*s*), as well as three methyl groups at: δ_H_ 1.38 (*d*, *J* = 6.0 Hz, H-6′), 1.59 (*d*, *J* = 5.5 Hz, H-2″), and 1.51 (*d*, *J* = 6.5 Hz, H-6‴). Together with ^13^C NMR signals at: δ_C_ 36.8 (C-2′) and 37.2 (C-2″) which were more deshielded than the typical value of 34.0 ppm and the large coupling constant (>7.0 Hz) for: δ_H_ 5.24 (H-1‴), supported by TOCSY (Figure S9) and COSY correlations, these findings suggested that the sugar chain consists of *β*-cymarose, *β*-oleandrose, and *β*-6-deoxy-3-*O*-methyl-allose.^15,18–21^ Consistently, all sugar units were assigned to the D series based on comparison with reported pregnane glycosides and established structural patterns in Apocynaceae.^6,22–24^

**Table 3:** ^1^H NMR Data the Sugar Moieties of Compounds for **1**–**9** (δ in ppm, *J* in Hz, C_5_D_5_N)

no	1^a^	2^a^	3^b^	4^a^	5^b^	6^b^	7^b^	8^b^	9^b^
	Cym	Cym	AcCym	Cym	Cym	Cym	AcCym	Cym	Cym
1′	5.19 (*br s*)	5.22 (*m*)	5.20 (*d*, 8.0)	5.26 (*m*)	5.28 (*br s*)	5.25 (*dd*, 10.0; 1.6	5.24 (*dd*, 9.2; 1.6)	5.26 (*d*, 2.0)	5.22 (*br s*)
2′	1.81 (*m*) 2.24 (dt, 12.0; 4.0)	1.81 (*m*) 2.22 (dt, 12.0; 3.5)	1.82 (*m*) 2.25 (*s*)	1.85 (*t*, 3.0) 2.27 (*m*)	1.85 (*m*) 2.29 (*m*)	1.81 (*m*) 2.25 (*m*)	2.01 (*m*) 2.41 (*m*)	1.84 (*m*) 2.25 (*m*)	1.82 (*m*) 2.22 (*m*)
3′	3.97 (*q*, 3.0)	3.98 (*q*, 3.0)	5.71 (*s*)	4.01 (*q*, 2.5)	4.02 (*q*, 2.8)	3.99 (*q*, 3.6)	5.73 (*q*, 3.2)	4.00 (*m*)	3.97 (*m*)
4′	3.42 (*m*)	3.43 (*m*)	3.60 (*m*)	3.47 (*dd*, 5.0; 2.0)	3.47 (*m*)	3.44 (*m*)	3.60 (*m*)	3.43 (*m*)	3.41 (*m*)
5′	4.16 (*m*)	4.20 (*m*)	4.20 (*m*)	4.23 (*m*)	4.24 (*m*)	4.10 (*m*)	4.22 (*m*)	4.14 (*m*)	4.18 (*m*)
6′	1.38 (*d*, 6.0)	1.42 (*d*, 6.5)	1.44 *(br s*)	1.44 (*d*, 6.0)	1.44 (*d*, 6.4)	1.41 (*d*, 6.0)	1.49 (*d*, 6.0)	1.42 (*d*, 6.4)	1.38 (*d*, 6.4)
OMe	3.50 (*s*)	3.51 (*s*)	-	3.54 (*s*)	3.55 (*s*)	3.52 (*s*)	-	3.54 (*s*)	3.50 (*s*)
–CO	-	-	-	-	-	-	-	-	-
–CH_3_	-	-	2.07 (s)	-	-	-	2.07 (s)	-	-
	Ole	Ole	Ole	Ole	Ole	Ole	Ole	Ole	Ole
1″	4.65 (*dd*, 9.5; 1.5)	4.64 (*dd*, 9.5; 2.0)	4.69 (*t*, 10.8)	4.67 (*dd*, 10.0; 2.0)	4.66 (*dd*, 9.6; 2.0)	4.63 (*dd*, 9.2; 1.2)	4.68 (*dd*, 9.6; 1.2)	4.65 (*dd*, 9.6; 1.6)	4.64 (*d*, 9.6)
2″	1.68 (*d*, 4.0) 2.41 (*m*)	1.69 (*m*) 2.44 (*m*)	1.71 (*m*) 2.39 (*s*)	1.75 (*dd*, 12.0; 3.0) 2.45 (*m*)	1.73 (*m*) 2.47 (*m*)	1.68 (*m*) 2.44 (*m*)	1.71 (*m*)	1.68 (*m*) 2.42 (*m*)	1.66 (*m*) 2.40 (*m*)
3″	3.54 (*t*, 1.5)	3.54 (*br s*)	3.55 (*m*)	3.57 (*m*)	3.58 (*m*)	3.50 (*m*)	3.41 (*m*)	3.53(*m*)	3.51 (*m*)
4″	3.56 (*br s*)	3.53 (*s*)	3.52 (*br s*)	3.58 (*m*)	3.58 (*m*)	3.51 (*m*)	3.49 (*m*)	3,52 (*m*)	3.50 (*m*)
5″	3.52 (*q*, 2.5)	3.53 (*s*)	3.52 (*br s*)	3.55 (*m*)	3.53 (*m*)	3.50 (*m*)	3.48 (*m*)	3.53 (*m*)	3.50 (*m*)
6″	1.59 (*d*, 5.5)	1.59 (*d*, 5.0)	1.59 (*br s*)	1.61 (*d*, 6.0)	1.62 (*d*, 6.0)	1.55 (*d*, 5.6)	1.53 (*d*, 6.4)	1.55 (*d*, 6.0)	1.55 (*d*, 4.8)
OMe	3.47 (*s*)	3.46 (*s*)	3.46 (*s*)	3.48 (*s*)	3.50 (*s*)	3.47 (*s*)	3.41 (*s*)	3.48 (*s*)	3.45 (*s*)
	All	All	All	All	All	All	All	All	All
1‴	5.24 (*d*, 8.0)	5.24 (*m*)	5.26 (*d*, 7.6)	5.27 (*m*)	5.26 (*br s*)	5.22 (*d*, 8.0)	5.19 (*dd*, 8.0; 1.6)	5.24 (*d*, 8.0)	5.16 (*br s*)
2‴	3.83 (*dd*, 8.5; 3.0)	3.83 (*dd*, 8.0; 3.0)	3.81 (*br s*)	3.86 (*m*)	3.87 (*br s*)	3.79 (*dd*, 8.0; 2.8)	3.78 (*m*)	3.80 (*d*, 3.2)	3.79 (*m*)
3‴	4.04 (*t*, 2.5)	4.04 (*t*, 2.5)	4.03 (*br s*)	4.05 (*t*, 3.0)	4.05 (*t*, 3.2)	4.38 (*t*, 2.8)	4.37 (*t*, 2.8)	4.39 (*t*, 2.8)	4.38 (*t*, 3.2)
4‴	3.58 (*d*, 3.0)	3.58 (*dd*, 10.0; 3.0)	3.55 (*br s*)	3.60 (*br s*)	3.60 (*m*)	3.63 (*dd*, 9.6; 2.4)	3.65 (*dd*, 10.0; 2.0)	3.66 (*dd*, 9.6; 2.4)	3.62 (*d*, 9.6))
5‴	4.12 (*m*)	4.12 (*m*)	4.12 (*m*)	4.14 (*m*)	4.14 (*m*)	4.22 (*m*)	4.14 (*m*)	4.22 (*m*)	4.19 (*m*)
6‴	1.51 (*d*, 6.5)	1.50 (*d*, 6.0)	1.50 (*s*)	1.53 (*d*, 6.0)	1.53 (*d*, 6.0)	1.59 (*d*, 6.4)	1.60 (*d*, 6.4)	1.58 (*d*, 5.6)	1.58 (*d*, 6.0)
OMe	3.79 (*s*)	3.79 (*s*)	3.79 (*s*)	3.81 (*s*)	3.81 (*s*)	3.77 (*s*)	3.76 (*s*)	3.78 (*s*)	3.77 *(s*)
	-	-	-	-	-	Glc	Glc	Glc	Glc
1‴′	-	-	-	-	-	4.86 (*d*, 7.6)	4.89 (*d*, 7.6)	4.89 (*d*, 8.4)	4.86 (*d*, 8.0)
2‴′	-	-	-	-	-	4.05 (*t*, 8.4)	4.06 (*m*)	4.05 (*m*)	3.95 (*m*)
3‴′	-	-	-	-	-	4.22 (*m*)	4.24 (*m*)	4.30 (*m*)	4.22 (*m*)
4‴′	-	-	-	-	-	4.23 (*m*)	4.26 (*m*)	4.24 (*m*)	4.21 (*m*)
5‴′	-	-	-	-	-	4.19 (*m*)	4.20 (*m*)	3.98 (*m*)	4.18 (*m*)
6‴′	-	-	-	-	-	4.44 (*dd*, 10.0; 3.6)	4.44 (*dd*, 14.4; 3.6)	4.44 (*dd*, 12.0; 3.2)	4.41 (*m*)
						Glc	Glc	Glc	Glc
1‴″	-	-	-	-	-	5.16 (*br s*)	5.17 (*dd*, 8.0; 1.6)	5.10 (*d*, 8.0)	5.12 (*br s*)
2‴″	-	-	-	-	-	3.97 (*m*)	3.98 (*d*, 8.4)	3.96 (*m*)	4.04 (*d*, 8.8)
3‴″	-	-	-	-	-	3.88 (*m*)	3.91 (*m*)	3.90 (*m*)	3.8 (*m*)
4‴″	-	-	-	-	-	4.13 (*m*)	4.17 (*m*)	4.12 (*m*)	4.11 (*m*)
5‴″	-	-	-	-	-	3.95 (*m*)	3.95 (*m*)	3.92 (*m*)	4.17 (*m*)
6‴″	-	-	-	-	-	4.23 (*m*) 4.49 (*dd*, 12.0; 2.0)	4.28 (*m*) 4.51 (*dd*, 11.6; 2.0)	4.24 (*m*) 4.50 (*d*, 2.4)	4.22 (*m*) 4.50 (*dd*, 11.2; 2.4)

a Measured at 500 MHz.

b Measured at 400 MHz: Cym = d-cymarose; AcCym = *O*-acetyl-d-cymarose; Ole = d-oleandrose; Glc = d-glucose; All = 6-deoxy-3-*O*-methyl-d-allose.

The sequence of the three sugars linked at C-3 of the aglycone 12-*O*-cinnamoyl-20-*O*-nicotinoyl-sarcostin was established by HMBC correlations: δ_H_ 5.19 (cymaropyranosyl, H-1′) with δ_C_ 77.3 (C-3), δ_H_ 4.65 (oleandropyranosyl, H-1″) with δ_C_ 83.1 (cymaropyranosyl, C-4′), and δ_H_ 5.24 (6-deoxy-3-*O*-methyl-allopyranosyl, H-1‴) with δ_C_ 82.6 (oleandropyranosyl, C-4″). Thus, compound **1** was identified as 12-*O*-cinnamoyl-20-*O*-nicotinoyl-(20*R*)-sarcostin-3-*O*-*β*-D-6-deoxy-3-*O*-methyl-allopyranosyl-(1 → 4)-*β*-d-oleandropyranosyl-(1 → 4)-*β*-d-cymaropyranoside, named ruehssioside A.

Compound **2** was obtained as white amorphous powder with an optical rotation of [α]_D_^20^ + 95.3 (c 0.10, MeOH). Its molecular formula was established as C_57_H_82_NO_18_ based on HR-ESI-MS [M + H]^+^ at *m*/*z* 1068.5499, calcd 1068.5526, Δ = 2.6 ppm, HDI = 18 (Figure S12). The ^1^H NMR spectrum (Table 1 and Figure S13), ^13^C NMR spectrum (Table 2 and Figures S14 and S15), and 2D NMR experiments (Figures S16, S18 and S19) were highly similar to those of ruehssioside A. Analysis of these spectra showed that compound **2** possesses the same sugar moiety attached at C-3 as compound **1**. However, the absence of olefinic signals and the presence of a methine signal [δ_H_ 0.96 (H-5); δ_C_ 44.8 (C-5)] and methylene signals [δ_H_ 1.09 and 1.72 (H-6a and H-6b); δ_C_ 24.7 (C-6)] suggested the lack of a double bond between C-5 and C-6. This assignment was confirmed by HMBC correlations (Figure S17) from δ_H_ 1.05 (19-CH_3_) and δ_H_ 3.81 (H-3) to δ_C_ 44.8 (C-5) (Figure 2). The relative configuration of compound **2** showed the same NOESY correlations as compound **1** (Figures 3 and S20), with additional correlations involving H-5/H-3 and H-5/H-9. These indicated that H-5 was assigned an *α*-orientation. Accordingly, the structure of 12-*O*-cinnamoyl-20-*O*-nicotinoyl-(20*R*)-5,6-dihydrosarcostin-3-*O*-*β*-D-6-deoxy-3-*O*-methyl-allopyranosyl-(1 → 4)-*β*-d-oleandropyranosyl-(1 → 4)-*β*-d-cymaropyranoside (ruehssioside B) was established.

Compound **3** was obtained as white amorphous powder with an optical rotation of [α]_D_^20^ + 87.5 (c 0.10, MeOH). Its molecular formula was established as C_58_H_81_NNaO_19_ based on HR-ESI-MS [M + Na]^+^ at *m*/*z* 1118.5291, calcd 1118.5295, Δ = 0.3 ppm, HDI = 19 (Figure S22). The IR spectrum showed absorptions consistent with hydroxyl groups (3452 cm^–1^), ester carbonyl (1731 cm^–1^), conjugated ester carbonyl (1717 cm^–1^), aromatic rings (1637, 1453 cm^–1^), olefinic bonds (1594 cm^–1^), and ether groups (1079 cm^–1^) (Figure S21). The ^1^H NMR spectrum (Table 1 and Figure S23) and ^13^C NMR spectrum (Table 2, Figures S24 and S25) of the aglycone portion of 3 were identical to those of compound **2**. However, in the sugar chain of **3**, when compared with **2**, the characteristic methoxy signal was absent and new signals corresponding to an acetyl group were observed [δ_H_ 2.07 (*s*, –CH_3_), δ_C_ 170.0 (–OCO), and δ_C_ 21.0 (–CH_3_)]. The position of the acetyl group was established by HMBC correlations (Figure S27) between δ_H_ 5.71 (H-3′) and δ_H_ 2.07 (–CH_3_) with δ_C_ 170.0 (C—O) (Figure 2). Accordingly, the structure of 12-*O*-cinnamoyl-20-*O*-nicotinoyl-(20*R*)-5,6-dihydrosarcostin-3-*O*-*β*-D-6-deoxy-3-*O*-methyl-allopyranosyl-(1 → 4)-*β*-d-oleandropyranosyl-(1 → 4)-*β*-D-3′-*O*-acetyl-cymaropyranoside (ruehssioside C) was established.

Compound **4** was obtained as white amorphous powder with an optical rotation of [α]_D_^20^ + 57.2 (c 0.10, MeOH). Its molecular formula was established as C_58_H_82_NaO_18_ based on HR-ESI-MS [M + Na]^+^ at *m*/*z* 1089.5383, calcd 1089.5393, Δ = 0.9 ppm, HDI = 18 (Figure S32). The NMR data of the aglycone of **4** (Tables 1, 2 and Figures S33–S35) were similar to those of compound **2**, except for the absence of nicotinoyl group signals and the presence of resonances consistent with a benzoyl group [δ_H_ 8.15 (*dd*, 8.5, 1.5 Hz, H-3,7), 7.28 (*m*, H-4,6), 7.47 (*tt*, 7,5, 1.5 Hz, H-5): δ_C_ 165.5 (C-1), 131.1 (C-2), 130.1 (C-3,7), 128.6 (C-4,6), and 133.1 (C-5)].^15^ HMBC correlations (Figure S37) between H-20 (δ_H_ 5.20) and C-1 (δ_C_ 165.5) (Bz) established the location of the benzoyl group at C-20 (Figure 2). The remaining signals of compound **4** were attributed to the sugar moiety, which showed a pattern highly similar to that of compound **2**, suggesting that both share the same sugar chain (Tables 3 and 4). Accordingly, the structure of **4** was defined as 12-*O*-cinnamoyl-20-*O*-benzoyl-(20*R*)-5,6-dihydrosarcostin-3-*O*-*β*-D-6-deoxy-3-*O*-methyl-allopyranosyl-(1 → 4)-*β*-d-oleandropyranosyl-(1 → 4)-*β*-d-cymaropyranoside (ruehssioside D).

**Table 4:** ^13^C NMR Data for the Sugar Moieties of Compounds **1**–**18** (δ in ppm, C_5_D_5_N)

no	1^a^	2^a^	3^b^	4^a^	5^b^	6^b^	7^b^	8^b^	9^b^	10^b^	11b	12b	13b	14b	15a	16a	17a	18b
	Cym	Cym	AcCym	Cym	Cym	Cym	AcCym	Cym	Cym	Cym	Cym	Dig	AcCym	Cym	Cym	Cym	AcCym	Cym
1′	96.1	95.6	95.8	95.8	95.7	95.7	95.9	95.7	96.0	95.9	96.3	95.8	95.9	95.8	95.7	95.8	95.9	95.9
2′	36.8	36.8	36.6	36.1	37.1	37.0	36.8	37.0	36.8	37.2	37.1	39.1	36.8	37.1	36.9	37.1	36.6	37.1
3′	77.5	77.5	70.5	77.7	77.7	77.6	70.6	77.6	77.5	77.8	77.7	67.4	70.6	77.8	77.6	77.7	70.5	77.8
4′	83.1	83.1	79.9	83.4	83.3	83.3	80.0	83.2	83.1	83.4	83.3	83.6	80.0	83.5	83.1	83.4	79.9	83.4
5′	68.7	68.6	69.2	68.8	68.8	68.7	69.3	68.7	68.7	68.8	68.8	68.3	68.8	68.8	68.7	68.8	69.2	68.8
6′	18.3	18.3	18.4	18.5	18.4	18.5	18.5	18.4	18.3	18.5	18.5	18.5	18.5	18.1	18.3	18.5	18.4	18.6
OMe	58.2	58.4	-	58.6	58.6	58.5	-	58.6	58.5	58.7	58.7	-	-	58.7	58.5	58.6	-	58.7
–OCO	-	-	170.0	-	-	-	170.1	-	-	-	-	-	170.1	-	-	-	170.0	-
–CH_3_	-	-	21.0	-	-	-	21.1	-	-	-	-	-	21.1	-	-	-	21.0	-
	Ole	Ole	Ole	Ole	Ole	Ole	Ole	Ole	Ole	Ole	Ole	Ole	Ole	Ole	Ole	Ole	Ole	Ole
1″	101.6	101.5	100.9	101.7	101.7	101.6	101.0	101.6	101.6	101.8	101.8	101.3	101.0	101.8	101.6	101.7	100.9	101.8
2″	37.2	37.2	37.0	37.4	37.4	37.4	37.2	37.4	37.3	37.2	37.4	37.1	37.1	37.6	37.2	37.4	37.0	37.5
3″	79.0	78.9	79.0	79.1	79.1	79.1	79.1	79.0	79.0	79.1	79.1	79.1	79.1	78.9	79.0	79.1	79.0	79.1
4″	82.6	82.6	82.5	82.8	82.7	82.8	82.6	82.8	82.7	82.8	82.7	82.9	82.6	82.9	82.6	82.8	82.5	82.8
5″	71.7	71.6	71.8	71.8	71.8	71.7	71.9	71.7	71.6	71.9	71.9	71.9	72.0	71.4	71.7	71.9	71.8	71.8
6″	18.6	18.4	18.4	18.5	18.5	18.6	18.6	18.6	18.6	18.6	18.8	18.8	18.6	18.6	18.4	18.8	18.4	18.8
OMe	56.9	56.9	56.9	57.1	57.0	57.1	57.1	57.1	57.0	57.1	57.1	57.1	57.0	57.3	56.9	57.1	56.9	57.1
	All	All	All	All	All	All	All	All	All	All	All	All	All	Can	All	All	All	All
1‴	101.7	101.7	101.7	101.9	101.8	101.6	101.7	101.6	101.6	101.9	101.9	101.9	101.8	100.2	101.7	101.9	101.7	101.9
2‴	72.9	72.8	72.9	73.1	73.0	72.4	72.5	72.4	72.3	73.1	73.1	73.1	73.1	39.8	72.9	73.1	72.9	73.1
3‴	83.7	83.7	83.7	83.9	83.8	82.8	82.9	82.8	82.7	83.9	83.9	83.9	83.8	71.3	83.7	83.9	83.7	83.9
4‴	74.3	74.2	74.3	74.4	74.4	83.2	83.2	83.2	83.1	74.5	74.5	74.4	74.4	88.2	74.3	74.4	74.3	74.5
5‴	70.6	70.6	70.7	70.8	70.7	69.2	69.3	69.2	69.2	70.8	70.8	70.8	70.8	70.1	70.6	70.8	70.7	70.9
6‴	18.3	18.6	18.5	18.8	18.7	18.0	18.1	18.0	17.9	18.8	18.5	18.6	18.6	18.6	18.6	18.6	18.5	18.5
OMe	61.8	61.8	61.9	61.9	61.9	61.5	61.5	61.5	61.5	62.0	62.0	62.0	61.9	-	61.8	62.0	61.9	62.0
						Glc	Glc	Glc	Glc					All				
1‴′	-	-	-	-	-	105.9	106.0	105.9	105.8	-				103.2				
2‴′	-	-	-	-	-	74.6	74.7	74.6	74.7	-				72.5				
3‴′	-	-	-	-	-	76.2	76.3	76.2	76.1	-				83.8				
4‴′	-	-	-	-	-	81.2	81.3	81.2	81.2	-				74.1				
5‴′	-	-	-	-	-	78.0	78.1	78.0	77.9	-				71.1				
6‴′	-	-	-	-	-	62.0	62.2	62.0	61.9	-				18.6				
OMe														62.1				
						Glc	Glc	Glc	Glc					-				
1‴″	-	-	-	-	-	104.7	104.8	104.7	104.6	-				-				
2‴″	-	-	-	-	-	74.7	74.8	74.7	74.6	-				-				
3‴″	-	-	-	-	-	76.3	76.4	76.3	76.2	-				-				
4‴″	-	-	-	-	-	71.3	71.4	71.3	71.3	-				-				
5‴″	-	-	-	-	-	78.3	78.4	78.3	78.2	-				-				
6‴″	-					62.2	62.3	62.2	62.1					-				

a Measured at 125 MHz.

b Measured at 100 MHz; Cym = d-cymarose; AcCym = *O*-acetyl-d-cymarose; Dig = d-digitoxose; Ole = d-oleandrose; All = 6-deoxy-3-*O*-methyl-d-allose; Can = d-canarose; Glc = d-glucose.

Compound **5** was obtained as white amorphous powder with an optical rotation of [α]_D_^20^ + 15.4 (c 0.10, MeOH). Its molecular formula was established as C_53_H_80_NaO_18_ based on HR-ESI-MS [M + Na]^+^ at *m*/*z* 1027.5208, calcd 1027.5237, Δ = 2.8 ppm, HDI = 14 (Figure S42). The ^1^H NMR spectrum (Table 1 and Figure S43), ^13^C NMR spectrum (Table 2, Figures S44 and S45), and 2D NMR data (Figures 2, 3 and S46–S50) of **5** exhibited signals and correlations very similar to those of compound **4**, indicating that both shared the same sugar chain and a C_21_ pregnane-type steroidal aglycone. However, the characteristic signals of the benzoyl group were absent, while typical signals of an acetyl group were observed [δ_H_ 1.92 (H-2); δ_C_ 170.5 (C-1) and 21.3 (C-2)]. The attachment of the acetyl group to C-20 was confirmed by the HMBC correlation (Figure S47) between δ_H_ 5.19 (H-20) and δ_C_ 170.5 (C—O) (Figure 2). Accordingly, the structure of **5** was established as 12-*O*-cinnamoyl-20-*O*-acetyl-(20*R*)-5,6-dihydrosarcostin-3-*O*-*β*-D-6-deoxy-3-*O*-methyl-allopyranosyl-(1 → 4)-*β*-d-oleandropyranosyl-(1 → 4)-*β*-d-cymaropyranoside (ruehssioside E).

Compound **6** was obtained as white amorphous powder with an optical rotation of [α]_D_^20^ + 30.5 (c 0.10, MeOH). Its molecular formula was established as C_63_H_98_NaO_26_ based on HR-ESI-MS [M + Na]^+^ at *m*/*z* 1293.6199, calcd 1293.6239, Δ = 3.0 ppm, HDI = 15 (Figure S52). Extensive analysis of the ^1^H and ^13^C NMR spectra (Tables 1, 2 and Figures S53–S55) revealed that its aglycone was highly similar to that of compound **5**, except at C-17. In compound **5**, this position bore an oxygenated function, as evidenced by a downfield shift (δ_C_ 87.6, C-17), whereas in compound **6**, C-17 resonated upfield at δ_C_ 51.8. This difference was confirmed by HMBC correlations (Figure S57) from δ_H_ 5.09 (H-12), δ_H_ 1.71 (18-CH_3_), and δ_H_ 1.14 (21-CH_3_) to δ_C_ 51.8 (C-17). In addition, the signals of the acetyl group were absent.

The relative configuration of compound **6** was deduced from its ROESY spectrum (Figures 3 and S60), showing correlations between H-3/H-1a, H-3/H-5, H-5/H-9, H-9/H-12, H-12/H-17, H-21/H-17, H-21/H-16a, H-20/H-17, H-19/H-7b, H-19/H-2b, and H-19/H-6a. These correlations indicated that H-3, H-5, H-9, H-12, H-16a, H-17, H-20, and H-21 were *α*-oriented, while H_3_-18 and H_3_-19 were *β*-oriented. Literature data report that, in 20-hydroxy-C/D-cis-pregnane steroids, steric hindrance between C-16 methylene (H-16), the substituent at C-20, and the methyl groups at C-18 and C-21 in the cyclopentane ring can cause characteristic differences in the ^13^C chemical shifts that indicate the absolute configuration at C-20. Reported values for C-20 and C-16 in 20-hydroxy-C/D-cis-pregnane steroids bearing a hydrogen at C-17 with 20*S* configuration are δ_C_ 65.0 and 19.0, respectively. These match the observed values for compound **6**, as C-20 and C-16 resonated at δ_C_ 65.4 and 19.0, respectively.^17,25^

Additionally, five anomeric protons were observed (Table 3) at δ_H_ 5.25 (*dd*, J = 10.0, 1.6 Hz, H-1′), 4.63 (*dd*, J = 9.2, 1.2 Hz, H-1″), 5.22 (*d*, J = 8.0 Hz, H-1‴), 4.86 (*d*, J = 7.6 Hz, H-1‴′), and 5.16 (*br s*, H-1‴″), which correlated in the HSQC spectrum (Figure S56) with carbons at δ_C_ 95.7 (C-1′), 101.6 (C-1″), 101.6 (C-1‴), 105.9 (C-1‴′), and 104.7 (C-1‴″), respectively. Comparison of the ^1^H and ^13^C NMR data of these sugars with those reported in the literature,^15,19–21^ together with TOCSY (Figure S59) and COSY (Figure S58) correlations, indicated the presence of one cymarose (Cym), one oleandrose (Ole), one 6-deoxy-3-*O*-methyl-allose (All), and two glucose (Glc) units.

The downfield ^13^C chemical shifts, along with coupling constants greater than 7.0 Hz for the glucose units,^15,18^ confirmed their *β*-anomeric configuration. The glucopyranosyl unit was characterized by NMR, and its D-configuration was assigned based on comparison with literature data and the predominant occurrence of d-glucose in pregnane glycosides from Apocynaceae.^6,22–24^ The sugars were therefore identified as *β*-d-cymarose, *β*-d-oleandrose, *β*-D-6-deoxy-3-*O*-methyl-allose, *β*-d-glucose, and *β*-d-glucose. The sequence of these five sugars was confirmed by HMBC correlations: δ_H_ 5.25 (cymaropyranosyl, H-1′) with δ_C_ 77.3 (C-3); δ_H_ 4.63 (oleandropyranosyl, H-1″) with δ_C_ 83.3 (cymaropyranosyl, C-4′); δ_H_ 5.22 (6-deoxy-3-*O*-methyl-allopyranosyl, H-1‴) with δ_C_ 82.8 (oleandropyranosyl, C-4″); δ_H_ 4.86 (6-deoxy-3-*O*-methyl-allopyranosyl, H-1‴′) with δ_C_ 83.2 (6-deoxy-3-*O*-methyl-allopyranosyl, C-4‴); and δ_H_ 5.16 (glucopyranosyl, H-1‴′′) with δ_C_ 81.2 (glucopyranosyl, C-4‴′). Thus, compound **6** was elucidated as 12-*O*-cinnamoyl-(20*S*)-5,6,17-trihydrosarcostin-3-*O*-*β*-d-glucopyranosyl-(1 → 4)-*β*-D glucopyranosyl-(1 → 4)-*β*-D-6-deoxy-3-*O*-methyl-allopyranosyl-(1 → 4)-*β*-D oleandropyranosyl-(1 → 4)-*β*-d-cymaropyranoside (ruehssioside F).

Compound **7** was obtained as white amorphous powder with an optical rotation of [α]_D_^20^ + 81.6 (c 0.10, MeOH). Its molecular formula was established as C_64_H_98_NaO_27_ based on HR-ESI-MS [M + Na]^+^ at *m*/*z* 1321.6167, calcd 1321.6188, Δ = 1.6 ppm, HDI = 16 (Figure S62). Analysis of the ^1^H and ^13^C NMR spectra (Tables 2, 5 and Figures S63–S65) revealed that compound **7** possessed the same aglycone as compound **6**. The sugar portion of both compounds was also very similar, except that **7** lacked the characteristic methoxy signals of the cymarose unit and instead showed resonances corresponding to an acetyl group [δ_C_ 170.1 (–OCO), δ_C_ 21.1 (–CH_3_), and δ_H_ 2.07 (*s*, –CH_3_)] (Tables 2 and 5). The position of the acetyl group was established by HMBC correlations (Figures 4 and S67) between δ_H_ 5.73 (H-3′) and δ_C_ 170.1 (–OCO). Accordingly, the structure of compound 7 was determined as 12-*O*-cinnamoyl-(20*S*)-5,6,17-trihydrosarcostin-3-*O*-*β*-d-glucopyranosyl-(1 → 4)-*β*-d-glucopyranosyl-(1 → 4)-*β*-D-6-deoxy-3-*O*-methyl-allopyranosyl-(1 → 4)-*β*-d-oleandropyranosyl-(1 → 4)-*β*-D-3′-*O*-acetyl-cymaropyranoside (ruehssioside G).

**Table 5:** ^1^H NMR Data for the Aglycone Moieties of Compounds **7**–**12** (δ in ppm, *J* in Hz, C_5_D_5_N)^a^

No	7^b^	8^b^	9^b^	10^b^	11^b^	12^b^
1	0.92 (*m*) 1.63 (*m*)	0.88 (*td*, 14.0; 4.8) 1.48 (*m*)	1.00 (*m*) 1.66 (*m*)	0.87 (*td*, 14.0; 4.0) 1.51 (*m*)	1.09 (*m*) 1.80 (*m*)	0.91 (*m*) 1.61 (*m*)
2	1.70 (*m*) 2.03 (*m*)	1.71 (*m*) 2.07 (*m*)	1.64 (*m*) 2.03 (*m*)	1.58 (*m*) 2.03 (*m*)	1.81 (*m*) 2.09 (*m*)	1.67 (*m*) 2.01 (*m*)
3	3.88 (*m*)	3.81 (*m*)	3.74 (*m*)	3.83 (*m*)	3.84 (*m*)	3.81 (*m*)
4	1.38 (*m*) 1.71 (*m*)	1.25 (*m*) 1.68 (*m*)	2.26 (*d*, 12.8) 2.45 (*dd*, 13.6; 6.0)	1.25 (*m*) 1.65 (*m*)	2.36 (*d*, 12.8) 2.55 (*d*, 13.2; 4.4)	1.40 (*m*) 1.73 (*m*)
5	1.03 (*m*)	0.98 (*m*)	-	0.95 (*m*)	-	0.98 (*m*)
6	1.08 (*m*) 1.80 (*m*)	1.22 (*m*)	5.36 (*d*, 2.4)	1.22 (*m*)	5.35 (*d*, 4.4)	1.08 (*m*) 1.78 (*m*)
7	1.87 (*m*) 2.17 (*m*)	1.84 (*m*) 2.16 (*m*)	2.36 (*m*) 2.59 (*d*, 16.0)	1.16 (*d*, 6.4) 2.39 (*d*, 5.6)	2.35 (*m*) 2.58 (*dd*, 12.0; 4.4)	1.87 (*m*) 2.16 (*m*)
8	-	1.87 (*d*, 2.0)	2.06 (*m*)	1.87 (*d*, 2.4)	-	-
9	1.44 (*br s*)	1.06 (*d*, 3.2)	1.39 (*br s*)	1.06 (*d*, 2.8)	1.77 (*d*, 3.2)	1.40 (*br s*)
10	-	-	-	-	-	-
11	1.95 (*m*) 2.29 (*m*)	1.51 (*m*) 1.94 (*m*)	1.66 (*m*) 1.97 (*m*)	1.50 (*m*) 1.97 (*m*)	2.00 (*m*) 2.38 (*m*)	1.92 (*m*) 2.27 (*m*)
12	5.13 (*dd*, 12.0; 3.2)	5.00 (*m*)	5.08 (*m*)	5.00 (*dd*, 11.6; 3.6)	5.19 (*dd*, 11.6; 3.6)	5.09 (*m*)
13	-	-	-	-	-	-
14	-	-	-	-	-	-
15	1.44 (*m*)	1.16 (*br s*) 2.37 (*m*)	1.88 (*m*)	1.86 (*m*) 2.09 (*m*)	2.07 (*m*)	1.42 (*m*)
16	1.90 (*m*) 2.17 (*m*)	1.91 (*m*) 2.25 (*m*)	1.86 (*m*) 2.17 (*m*)	1.93 (*m*) 2.16 (*m*)	2.11 (*m*)	1.90 (*m*) 2.19 (*m*)
17	2.10 (*m*)	2.15 (*m*)	2.15 (*m*)	2.14 (*m*)	2.14 (*m*)	2.08 (*m*)
18	1.73 (*s*)	1.49 (*s*)	1.48 (*s*)	1.49 (*s*)	1.73 (*s*)	1.72 (*s*)
19	1.19 (*s*)	0.73 (*s*)	0.94 (*s*)	0.73 (*s*)	1.38 (*s*)	1.17 (*s*)
20	4.21 (*m*)	4.22 (*m*)	4.21 (*m*)	4.24 (*m*)	4.21 (*m*)	4.21 (*m*)
21	1.17 (*d*, 6.4)	1.20 (*d*, 6.4)	1.18 (*d*, 6.4)	1.20 (*d*, 6.4)	1.17 (*d*, 6.0)	1.16 (*d*, 6.0)
12-O	Cin	Cin	Cin	Cin	Cin	Cin
1	-	-	-	-	-	-
2	6.93 (*d*, 16.0)	6.93 (*d*, 16.0)	6.89 (*d*, 16.0)	6.91 (*d*, 16.0)	6.92 (*d*, 16.0)	6.91 (*d*, 16.0)
3	8.07 (*d*, 16.0)	8.07 (*d*, 16.0)	8.03 (*d*, 16.0)	8.05 (*d*, 16.0)	8.06 (*d*, 16.0)	8.05 (*d*, 16.0)
4	-	-	-	-	-	-
5	7.65 (*m*)	7.34 (*m*)	7.64 (*m*)	7.66 (*m*)	7.65 (*m*)	7.64 (*m*)
6	7.35 (*m*)	7.65 (*m*)	7.33 (*m*)	7.33 (*m*)	7.35 (*m*)	7.33 (*m*)
7	7.35 (*m*)	7.65 (*m*)	7.33 (*m*)	7.33 (*m*)	7.35 (*m*)	7.33 (*m*)
8	7.35 (*m*)	7.65 (*m*)	7.33 (*m*)	7.33 (*m*)	7.35 (*m*)	7.33 (*m*)
9	7.65 (*m*)	7.34 (*m*)	7.64 (*m*)	7.66 (*m*)	7.65 (*m*)	7.64 (*m*)

a Measured at 400 MHz; Cin = cinnamoyl.

**Figure 4:**
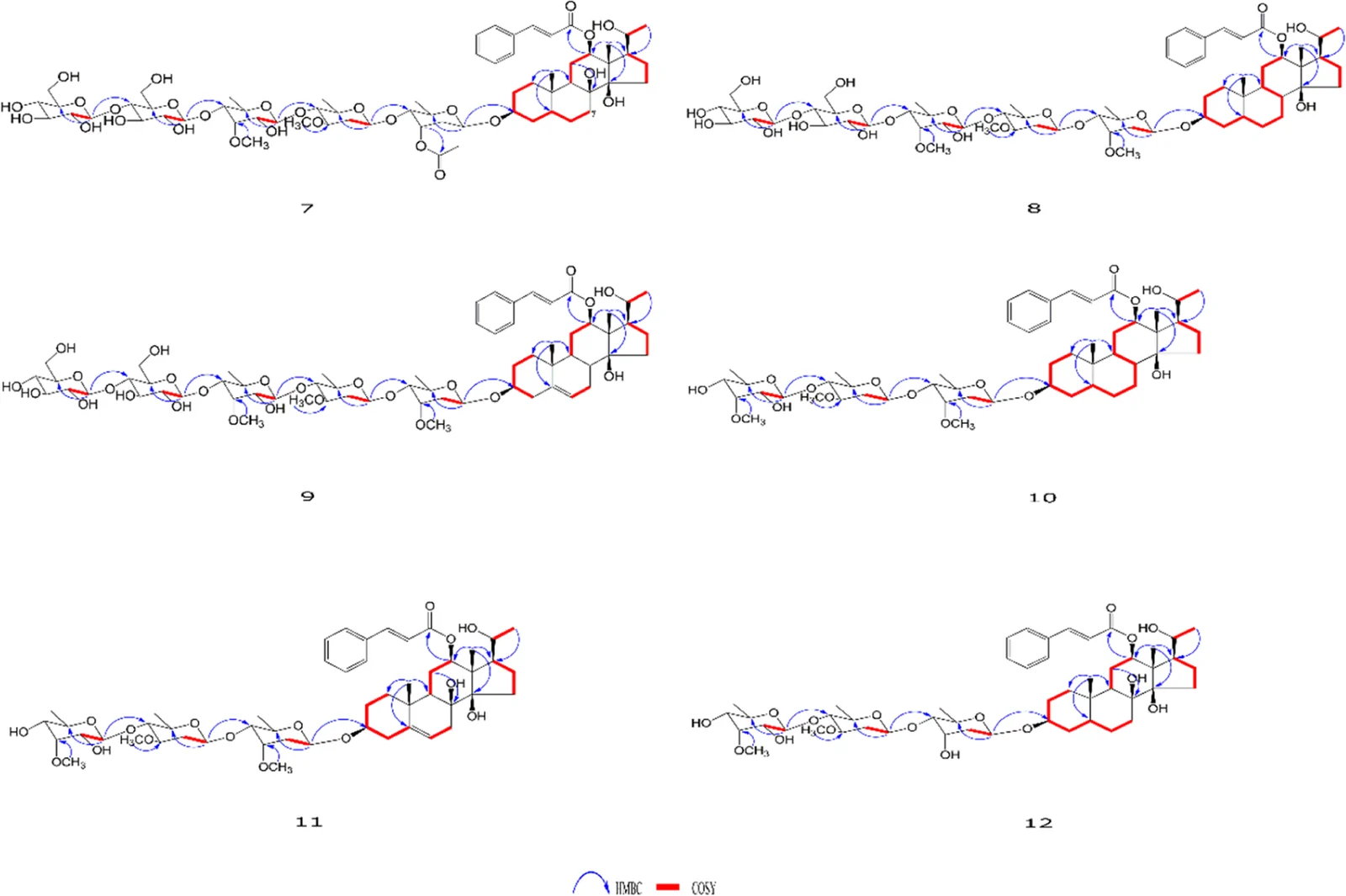
Continuation of Figure 2: key ^1^H–^13^C HMBC and ^1^H–^1^H COSY correlations of compounds **7**–**12**.

Compound **8** was obtained as white amorphous powder with an optical rotation of [α]_D_^20^ – 12.4 (c 0.10, MeOH). Its molecular formula was established as C_63_H_98_NaO_25_ based on HR-ESI-MS [M + Na]^+^ at *m*/*z* 1277.6269, calcd 1277.6289, Δ = 1.6 ppm, HDI = 15 (Figure S72). The ^1^H, ^13^C (Figures S73–S75), and 2D NMR spectra (Figures S76–S80) showed that the sugar portion of **8** was identical to that of compound **6**. However, in the pregnane aglycone moiety, the oxygenated function present at C-8 in compound **6** was not observed in the NMR spectrum of **8**. Instead, signals at δ_H_ 1.87 and δ_C_ 40.0 were attributed to H-8 and C-8, respectively, consistent with literature data.^20^ This assignment was confirmed by COSY correlations (Figure S78) between δ_H_ 1.87 (H-8) and δ_H_ 1.06 (H-9), as well as HMBC correlations (Figure S32) from δ_H_ 1.51 (H-11) and δ_H_ 1.16 (H-15) to δ_C_ 40.0 (C-8).

The relative configuration of compound **8** was consistent with all the NOESY correlations (Figures 3 and S80) observed for compound **6**, with additional correlations between H-8/H-19 and H-8/H-16b. These data indicated that H-8 was *β*-oriented. Accordingly, the structure of compound **8** was determined as 12-*O*-cinnamoyl-(20*S*)-5,6,8,17-tetrahydrosarcostin-3-*O*-*β*-d-glucopyranosyl-(1 → 4)-*β*-d-glucopyranosyl-(1 → 4)-*β*-D-6-deoxy-3-*O*-methyl-allopyranosyl-(1 → 4)-*β*-d-oleandropyranosyl-(1 → 4)-*β*-d-cymaropyranoside (ruehssioside H).

Compound **9** was obtained as white amorphous powder with an optical rotation of [α]_D_^20^ + 10.4 (c 0.10, MeOH). Its molecular formula was established as C_63_H_96_NaO_25_ based on HR-ESI-MS [M + Na]^+^ at *m*/*z* 1275.6097, calcd 1275.6133, Δ = 2.8 ppm, HDI = 16 (Figure S82). The ^1^H, ^13^C (Figures S83–S85), and 2D NMR spectra (Figures S86–S90) showed that the sugar portion of **9** was identical to that of compound **8**. The other signals indicated a C_21_ pregnane-type steroidal nucleus and a cinnamoyl group, the latter confirmed to be attached at C-12 of the aglycone by the HMBC correlation (Figure S87) of δ_H_ 5.08 (H-12) with δ_C_ 166.9 (C-1). In compound 9, additional characteristic olefinic signals were observed in δ_H_ 5.36 (H-6), δ_C_ 139.2 (C-5), and δ_C_ 122.2 (C-6)], which were not present in compound **8**. The location of the unsaturation was determined to be between C-5 and C-6 based on COSY correlations (Figure S88) of δ_H_ 5.36 (H-6) with δ_H_ 2.59 (H-7) and HMBC correlations of δ_H_ 3.74 (H-3), δ_H_ 2.06 (H-8), δ_H_ 1.39 (H-9), and δ_H_ 0.94 (H-19) with δ_C_ 139.2 (C-5). Thus, the structure of compound **9** was confirmed as 12-*O*-cinnamoyl-(20*S*)-8,17-dihydrosarcostin-3-*O*-*β*-d-glucopyranosyl-(1 → 4)-*β*-d-glucopyranosyl-(1 → 4)-*β*-D-6-deoxy-3-*O*-methyl-allopyranosyl-(1 → 4)-*β*-d-oleandropyranosyl-(1 → 4)-*β*-d-cymaropyranoside (ruehssioside I).

Compound **10** was obtained as white amorphous powder with an optical rotation of [α]_D_^20^ – 5.8 (c 0.10, MeOH). Its molecular formula was established as C_51_H_78_NaO_15_ based on HR-ESI-MS [M + Na]^+^ at *m*/*z* 953.5219, calcd 953.5233, Δ = 1.5 ppm, HDI = 13 (Figure S92). A detailed comparison of the ^1^H and ^13^C NMR data (Tables 2, 5 and Figures S93–S95) of **10** and **8** showed no significant differences in the aglycone portion, confirming that both shared the same nucleus, 12-*O*-cinnamoyl-5,6,8,17-tetrahydrosarcostin. The signals corresponding to the sugar chain (Tables 4 and 6) and 2D correlations (Figures S96–S100) revealed that compound **10** contained the same sugar units cymarose (Cym), oleandrose (Ole), and 6-deoxy-3-*O*-methyl-allose (All) in the same sequence as compounds **1**, **2**, **4**, and **5**, with this chain attached at C-3 of the aglycone. Accordingly, the structure of compound **10** was established as 12-*O*-cinnamoyl-(20*S*)-5,6,8,17-tetrahydrosarcostin-3-*O*-*β*-D-6-deoxy-3-*O*-methyl-allopyranosyl-(1 → 4)-*β*-d-oleandropyranosyl-(1 → 4)-*β*-d-cymaropyranoside (ruehssioside J).

**Table 6:** ^1^H NMR Data the Sugar Moieties of Compounds for **10**–**18** (δ in ppm, *J* in Hz, C_5_D_5_N)

No	10^b^	11^b^	12^b^	13^b^	14^b^	15^a^	16^a^	17^a^	18^b^
	Cym	Cym	Dig	AcCym	Cym	Cym	Cym	AcCym	Cym
1′	5.23 (*d*, 2.0)	5.24 (*d*, 10.0)	5.49 (*dd*, 9.2; 2.0)	5.24 (*dd*, 9.2; 2.4)	5.28 (*dd*, 9.6; 1.6)	5.25 (*m*)	5.25 (*m*)	5.16 (*d*, 10.0)	5.26 (*dd*, 9.6; 2.0)
2′	1.84 (*dd*, 13.2; 2.4) 2.27 (*m*)	1.85 (*m*) 2.25 (*m*)	2.01 (*m*) 2.36 (*m*)	1.90 (*d*, 2.4) 2.38 (*m*)	1.87 (*m*) 2.31 (*m*)	1.83 (*d*, 3.0) 2.26 (*m*)	1.84 (*m*) 2.28 (*m*)	1.98 (*m*) 2.37 (*m*)	1.87 (*m*) 2.29 (*m*)
3′	4.02 (*q*, 2.4)	4.00 (*m*)	4.61 (*q*, 2.8)	5.73 (*q*, 3.2)	4.03 (*q*, 2.8)	3.99 (*q*, 3.0)	4.01 (*m*)	5.70 (*m*)	4.01 (*q*, 3.2)
4′	3.44 (*m*)	3.45 (*m*)	3.47 (*m*)	3.61 (*m*)	3.47 (*m*)	3.44 (*m*)	3.47 (*m*)	3.59 (*m*)	3.47 (*m*)
5′	4.22 (*m*)	4.19 (*m*)	4.34 (*m*)	4.25 (*m*)	4.26 (*m*)	4.22 (*m*)	4.23 (*m*)	4.19 (*m*)	4.25 (*m*)
6′	1.42 (*d*, 6.4)	1.40 (*d*, 7.6)	1.48 (*d*, 6.4)	1.42 (*d*, 6.0)	1.44 (*d*, 4.4)	1.47 (*d*, 6.5)	1.43 (*d*, 6.0)	1.39 (*d*, 4.8)	1.44 (*d*, 6.0)
OMe	3.55 (*s*)	3.52 (*s*)	-	-	3.52 (*s*)	3.52 (*s*)	3.54 (*s*)	-	3.54 (*s*)
–OCO	-	-	-	-	-	-	-	-	-
-CH_3_	-	-	-	2.08 (s)	-	-	-	2.06 (s)	-
	Ole	Ole	Ole	Ole	Ole	Ole	Ole	Ole	Ole
1″	4.67 (*dd*, 10.0; 2.0)	4.66 (*dd*, 9.2; 1.6)	4.70 (*dd*, 9.6; 1.6)	4.69 (*m*)	4.66 (*dd*, 9.6; 1.6)	4.64 (*dd*, 10.0; 2.0)	4.67 (*dd*, 9.5; 2.0)	4.67 (*dd*, 8.0; 1.2)	4.68 (*dd*, 9.6; 2.0)
2″	1.73 (*m*) 2.44 (*m*)	1.72 (*m*) 2.35 (*m*)	1.69 (*m*) 2.44 (*m*)	1.74 (*m*) 2.43 (*m*)	1.71 (*m*) 2.42 (*m*)	1.68 (*m*) 2.43 (*m*)	1.71 (*m*) 2.44 (*m*)	1.71 (*m*) 2.41 (*m*)	1.72 (*m*) 2.47 (*m*)
3″	3.56 (*m*)	3.56 (*m*)	3.57 (*m*)	3.54 (*m*)	3.50 (*m*)	3.55 (*m*)	3.56 (*m*)	3.55 (*m*)	3.56 (*m*)
4″	3.57 (*m*)	3.57 (*m*)	3.54 (*m*)	3.53 (*m*)	3.44 (*m*)	3.54 (*m*)	3.57 (*m*)	3.52 (*m*)	3.57 (*m*)
5″	3.52 (*m*)	3.53 (*m*)	3.54 (*m*)	3.52 (*m*)	3.44 (*m*)	3.54 (*m*)	3.54 (*m*)	3.51 (*m*)	3.53 (*m*)
6″	1.62 (*d*, 5.6)	1.62 (*d*, 5.6)	1.57 (*d*, 6.4)	1.58 (*d*, 6.0)	1.58 (*d*, 6.0)	1.57 (*d*, 6.0)	1.60 (*br s*)	1.58 (*d*, 4.4)	1.62 (*d*, 5.6)
OMe	3.49 (*s*)	3.49 (*s*)	3.49 (*s*)	3.46 (*s*)	3.45 (*s*)	3.47 (*s*)	3.49 (*s*)	3.45 (*s*)	3.50 (*s*)
	All	All	All	All	Can	All	All	All	All
1‴	5.27 (*d*, 8.0)	5.27 (*d*, 8.0)	5.27 (*d*, 8.4)	5.21 (*d*, 8.0)	4.90 (*d*, 10.4)	5.26 (*d*, 8.5)	5.27 (*d*, 8.5)	5.21 (*d*, 6.0)	5.28 (*d*, 8.0)
2‴	3.85 (*m*)	3.85 (*m*)	3.85 (*m*)	3.84 (*m*)	1.93 (*m*) 2.54 (*dd*, 5.2; ;1.2)	3.82 (*d*, 3.0)	3.85 (*m*)	3.80 (*m*)	3.87 (*m*)
3‴	4.05 (*t*, 2.8)	4.05 (*t*, 2.8)	4.04 (*t*, 2.8)	4.05 (*t*, 2.8)	3.56 (*m*)	4.04 (*t*, 2.5)	4.05 (*t*, 2.0)	4.03 (*t*, 2.4)	4.05 (*t*, 2.8)
4‴	3.58 (*d*, 1.2)	3.60 (*br s*)	3.61 (*d*, 2.8)	3.58 (*d*, 2.0)	3.32 (*m*)	3.56 (*br s*)	3.58 (*m*)	3.57 (*d*, 2.0)	3.60 (*d*, 2.8)
5‴	4.13 (*m*)	4.13 (*m*)	4.14 (*m*)	4.13 (*m*)	4.00 (*m*)	4.12 (*m*)	4.14 (*m*)	4.12 (*m*)	4.16 (*m*)
6‴	1.51 (*d*, 6.4)	1.53 (*d*, 6.0)	1.53 (*d*, 6.4)	1.48 (*d*, 5.6)	1.36 (*d*, 6.0)	1.50 (*d*, 6.5)	1.53 (*d*, 6.0)	1.49 (*d*, 5.2)	1.53 (*d*, 6.0)
OMe	3.81 (*s*)	3.81(*s*)	3.80 (*s*)	3.79 (*s*)	-	3.80 (*s*)	3.81 (*s*)	3.79 (*s*)	3.81 (*s*)
					All				
1‴′					5.08 (*d*, 8.4)				
2‴′					3.93 (*m*)				
3‴′					4.05 (*q*, 2.8)				
4‴′					3.57 (*m*)				
5‴′					4.18 (*m*)				
6‴′					1.46 (*d*, 4.4)				
OMe					3.80 (*s*)				

a Measured at 500 MHz.

b Measured at 400 MHz: Cym = cymarose; AcCym = *O*-acetyl-d-cymarose; Dig = digitoxose; Ole = oleandrose; All = 6-deoxy-3-*O*-methyl-allose; Can = canarose.

Compound **11** was obtained as white amorphous powder with an optical rotation of [α]_D_^20^ – 3.4 (c 0.10, MeOH). Its molecular formula was established as C_51_H_76_NaO_16_ based on HR-ESI-MS [M + Na]^+^ at *m*/*z* 967.5007, calcd 967.5026, Δ = 2.0 ppm, HDI = 14 (Figure S102). The ^1^H, ^13^C (Tables 4, 6 and Figures S103–S105), and 2D NMR spectra (Figures 4, 5 and S106–S110) showed that the sugar portion of **11** was identical to that of compound **10**.

**Figure 5:**
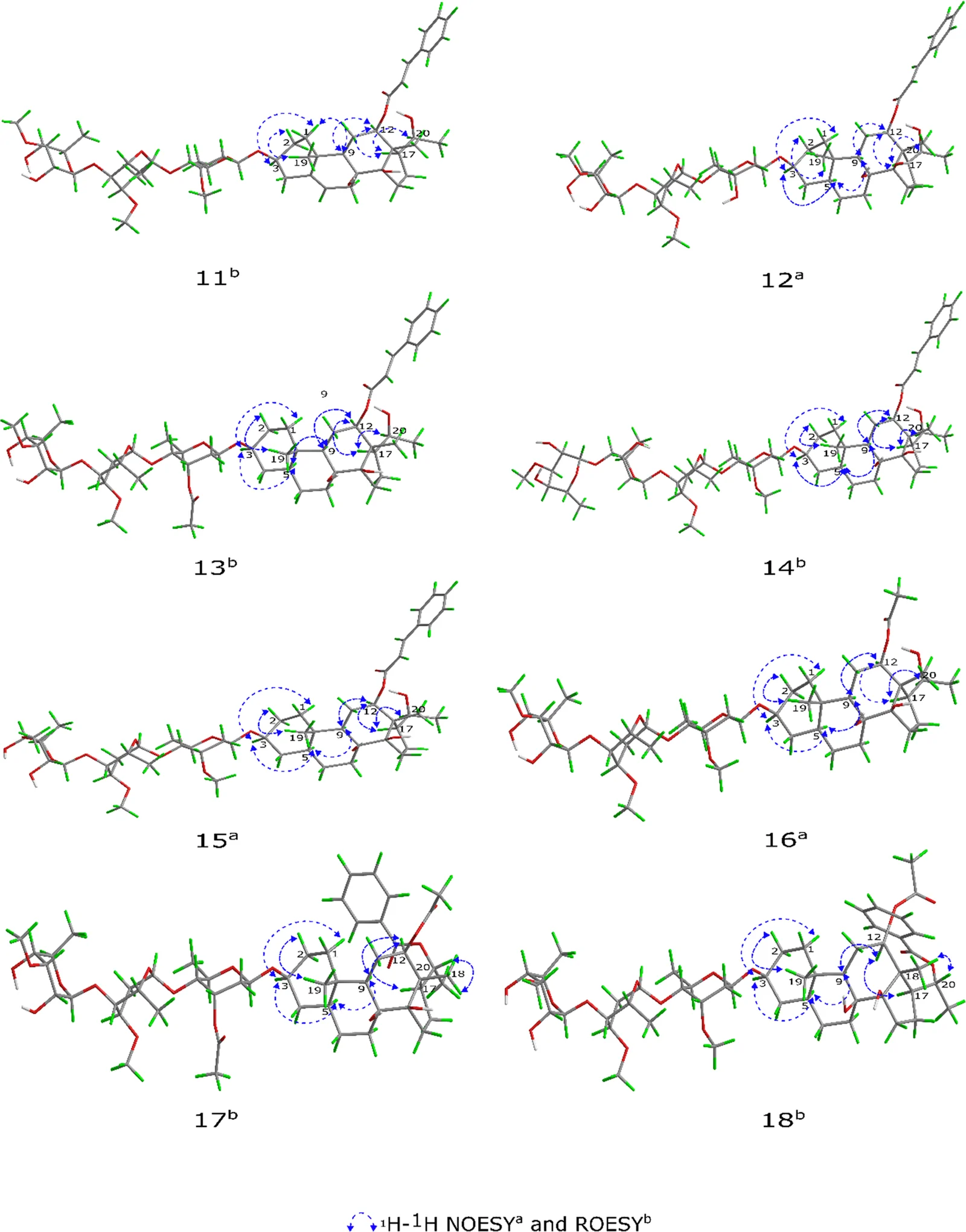
Continuation of Figure 3: key ^1^H–^1^H NOESY^a^ and ^1^H–^1^H ROESY^b^ correlations of compounds **11-18**.

The remaining signals were characteristic of a C21 pregnane-type steroidal aglycone. The aglycone of **11** was highly similar to that of compound **6**; however, compound **11** displayed additional characteristic olefinic signals: δ_H_ 5.35 (H-6), δ_C_ 138.9 (C-5), and δ_C_ 119.7 (C-6), which were not observed in compound **6**. The position of the double bond was assigned between C-5 and C-6 based on COSY correlations (Figure S108) of δ_H_ 5.35 (H-6) with δ_H_ 2.35 (H-7a) and δ_H_ 2.58 (H-7b) and HMBC correlations (Figure S107) of δ_H_ 3.84 (H-3), δ_H_ 1.77 (H-9) and δ_H_ 1.38 (H-19) with δ_C_ 138.9 (C-5) (Figure 4). The sugar chain of **11** was identified as the same as that of compound **10**, attached to the aglycone at C-3, as determined from 1D and 2D NMR analyses. Accordingly, the structure of compound **11** was established as 12-*O*-cinnamoyl-(20*S*)-17-hydrosarcostin-3-*O*-*β*-D-6-deoxy-3-*O*-methyl-allopyranosyl-(1 → 4)-*β*-d-oleandropyranosyl-(1 → 4)-*β*-d-cymaropyranoside (ruehssioside K).

Compound **12** was obtained as white amorphous powder with an optical rotation of [α]_D_^20^ + 23.4 (c 0.10, MeOH). Its molecular formula was established as C_5__0_H_7__6_NaO_1__6_ based on HR-ESI-MS [M + Na]^+^ at *m*/*z* 955.5000, calcd 955.5026, Δ = 2.7 ppm, HDI = 13 (Figure S112). Comparison of the 1D NMR (Figures S113–S115) signals (Tables 1, 2, 5, and 7) and the key 2D correlations (Figures S116–S120) demonstrated that, in addition to compounds **6** and **7**, substances **12**, **13**, **14**, and **15** shared the same aglycone, 12-*O*-cinnamoyl-5,6,17-trihydrosarcostin. The other signals assigned to the sugar units (Tables 1, 4, and 6) of **12** were very similar to those of compounds **1**, **2**, **4**, **5**, **10**, and **11**. However, the characteristic methoxy group signals were absent in compound **12**, indicating the presence of a *β*-d-digitoxopyranosyl unit. This assignment was supported by the ^1^H and ^13^C NMR data, which were consistent with those reported for *β*-d-digitoxopyranosyl residues in pregnane glicosides.^6,14,23^

**Table 7:** ^1^H NMR Data for the Aglycone Moieties of Compounds **13**–**18** (δ in ppm, *J* in Hz, C_5_D_5_N)

no	13^b^	14^b^	15^a^	16^a^	17^a^	18^b^
1	0.93 (*m*) 1.62 (*m*)	0.93 (*m*) 1.62 (*m*)	0.88 (*m*) 1.57 (*m*)	0.85 (*m*) 1.58 (*m*)	0.84 (*m*) 1.60 (*m*)	0.85 (*td*, 13.2; 3.6) 1.59 (*m*)
2	1.68 (*m*) 2.04 (*m*)	1.69 (*m*) 2.03 (*m*)	1.66 (*m*) 1.99 (*m*)	1.69 (*m*) 1.97 (*m*)	1.67 (*m*) 1.97 (*m*)	1.66 (*m*) 1.98 (*m*)
3	3.87 (*m*)	3.89 (*m*)	3.86 (*m*)	3.86 (*m*)	3.85 (*m*)	3.85 (*m*)
4	1.42 (*m*) 1.71 (*m*)	1.39 (*m*) 1.75 (*m*)	1.37 (*m*) 1.68 (*m*)	1.71 (*m*)	1.38 (*m*) 1.68 (m)	1.34 (*m*) 1.72 (m)
5	1.01 (*m*)	1.02 (*m*)	0.98 (*m*)	0.98 (*m*)	0.99 (*m*)	0.98 (*m*)
6	1.09 (*m*) 1.84 (*m*)	1.12 (*m*) 1.79 (*m*)	1.05 (*m*) 1.75 (*m*)	1.06 (*m*) 1.76 (*m*)	1.05 (*m*) 1.74 (*m*)	1.09 (*m*) 1.82 (*m*)
7	1.87 (*m*) 2.16 (*m*)	1.91 (*m*) 2.09 (*m*)	1.85 (*m*) 2.14 (*m*)	1.84 (*m*) 2.16 (*m*)	1.42 (*m*) 2.12 (*m*)	1.40 (*m*) 2.10 (*m*)
8	-	-	-	-	-	-
9	1.43 (*br s*)	1.43 (*br s*)	1.38 (*br s*)	1.33 (*d*, 3.5)	1.25 (*br s*)	1.28 (*br s*)
10	-	-	-	-	-	-
11	1.98 (*m*) 2.27 (*d*, 11.6)	1.93 (*m*) 2.28 (*m*)	1.89 (*m*) 2.23 (*m*)	1.81 (*m*) 2.15 (*m*)	1.76 (*m*) 1.99 (*m*)	1.76 (*m*) 2.00 (*m*)
12	5.11 (*dd*, 11.6; 3.6)	5.14 (*m*)	5.10 (*dd*, 12.0; 4.0)	4.91 (*dd*, 12.5; 4.5)	4.89 (*dd*, 9.6; 3.2)	4.89 (*dd*, 12.8; 3.2)
13	-	-	-	-	-	-
14	-	-	-	-	-	-
15	1.43 (*m*)	1.41 (*m*) 2.16 (*m*)	1.39 (*m*) 2.14 (*m*)	1.39 (*m*)	1.36 (*m*)	1.36 (*m*)
16	1.90 (*m*) 2.20 (*m*)	1.87 (*m*) 2.10 (*m*)	1.88 (*m*) 2.14 (*m*)	1.86 (*m*) 2.13 (*m*)	2.12 (*m*) 2.41 (*m*)	2.13 (*m*) 2.40 (*m*)
17	2.10 (*m*)	2.09 (*m*)	2.03 (*m*)	2.00 (*m*)	2.17 (*m*)	2.19 (*td*, 8.8; 3.2)
18	1.73 (*s*)	1.72 (*s*)	1.69 (*s*)	1.60 (*s*)	1.55 (*s*)	1.57 (*s*)
19	1.19 (*s*)	1.19 (*s*)	1.14 (*s*)	1.15 (*s*)	1.10 (*s*)	1.12 (*s*)
20	4.23 (*m*)	4.22 (*m*)	4.19 (*m*)	4.17 (*m*)	5.53 (*m*)	5.55 (*m*)
21	1.17 (*d*, 6.0)	1.15 (*d*, 6.4)	1.14 (*d*, 6.0)	1.18 (*d*, 6.5)	1.36 (*d*, 4.8)	1.38 (*d*, 6.0)
12-O	Cin	Cin	Cin	Ac	Ac	Ac
1	-	-	-	-	-	-
2	6.91 (*d*, 16.0)	6.87 (*d*, 16.0)	6.87 (*d*, 16.0)	-	-	-
3	8.05 (*d*, 16.0)	8.01 (*d*, 16.0)	8.01 (*d*, 16.0)	-	-	-
4	-	-	-	-	-	-
5	7.64 (*m*)	7.62 (*m*)	7.62 (*m*)	2.12 (*s*)	2.09 (*s*)	2.09 (s)
6	7.33 (*m*)	7.33 (*m*)	7.33 (*m*)	-	-	-
7	7.33 (*m*)	7.33 (*m*)	7.33 (*m*)	-	-	-
8	7.33 (*m*)	7.33 (*m*)	7.33 (*m*)	-	-	-
9	7.64 (*m*)	7.62 (*m*)	7.62 (*m*)	-	-	-
20-O	-	-	-	-	Bz	Bz
1	-	-	-	-	-	-
2	-	-	-	-	-	-
3	-	-	-	-	8.34 (*d*, 5.6)	8.35 (*dd*, 8.4; 1.2)
4	-	-	-	-	7.28 (*m*)	7.28 (*m*)
5	-	-	-	-	7.44 (*tt*, 5.6; 1.2)	7.45 (tt, 7.2; 1.6)
6	-	-	-	-	7.28 (*m*)	7.28 (*m*)
7	-	-	-	-	8.34 (*d*, 5.6)	8.35 (*dd*, 8.4; 1.2)

a Measured at 500 MHz.

b Measured at 400 MHz: Cin = cinnamoyl; Bz = benzoyl; Ac = acetyl.

The sugar chain was attached to the aglycone at C-3, as indicated by the HMBC correlation between δ_H_ 3.82 (H-3) and δ_C_ 95.8 (C-1′). Accordingly, the structure of compound **12** was determined as 12-*O*-cinnamoyl-(20*S*)-5,6,17-trihydrosarcostin-3-*O*-*β*-D-6-deoxy-3-*O*-methyl-allopyranosyl-(1 → 4)-*β*-d-oleandropyranosyl-(1 → 4)-*β*-d-digitoxopyranoside (ruehssioside L).

Compound **13** was obtained as white amorphous powder with an optical rotation of [α]_D_^20^ + 59.7 (c 0.10, MeOH). Its molecular formula was established as C_52_H_78_NaO_17_ based on HR-ESI-MS [M + Na]^+^ at *m*/*z* 997.5124, calcd 997.5131, Δ = 0.7 ppm, HDI = 14 (Figure S122). The sugar chain of **13** was identified as being the same as that of compound **3** and was attached to the aglycone at C-3, according to 1D NMR (Tables 2, 7 and Figures S123–S125) and 2D NMR analyses (Figures 5, 6 and S126–S130). Accordingly, the structure of compound **13** was determined as 12-*O*-cinnamoyl-(20*S*)-5,6,17-trihydrosarcostin-3-*O*-*β*-D-6-deoxy-3-*O*-methyl-allopyranosyl-(1 → 4)-*β*-d-oleandropyranosyl-(1 → 4)-*β*-D-3′-*O*-acetyl-cymaropyranoside (ruehssioside M).

**Figure 6:**
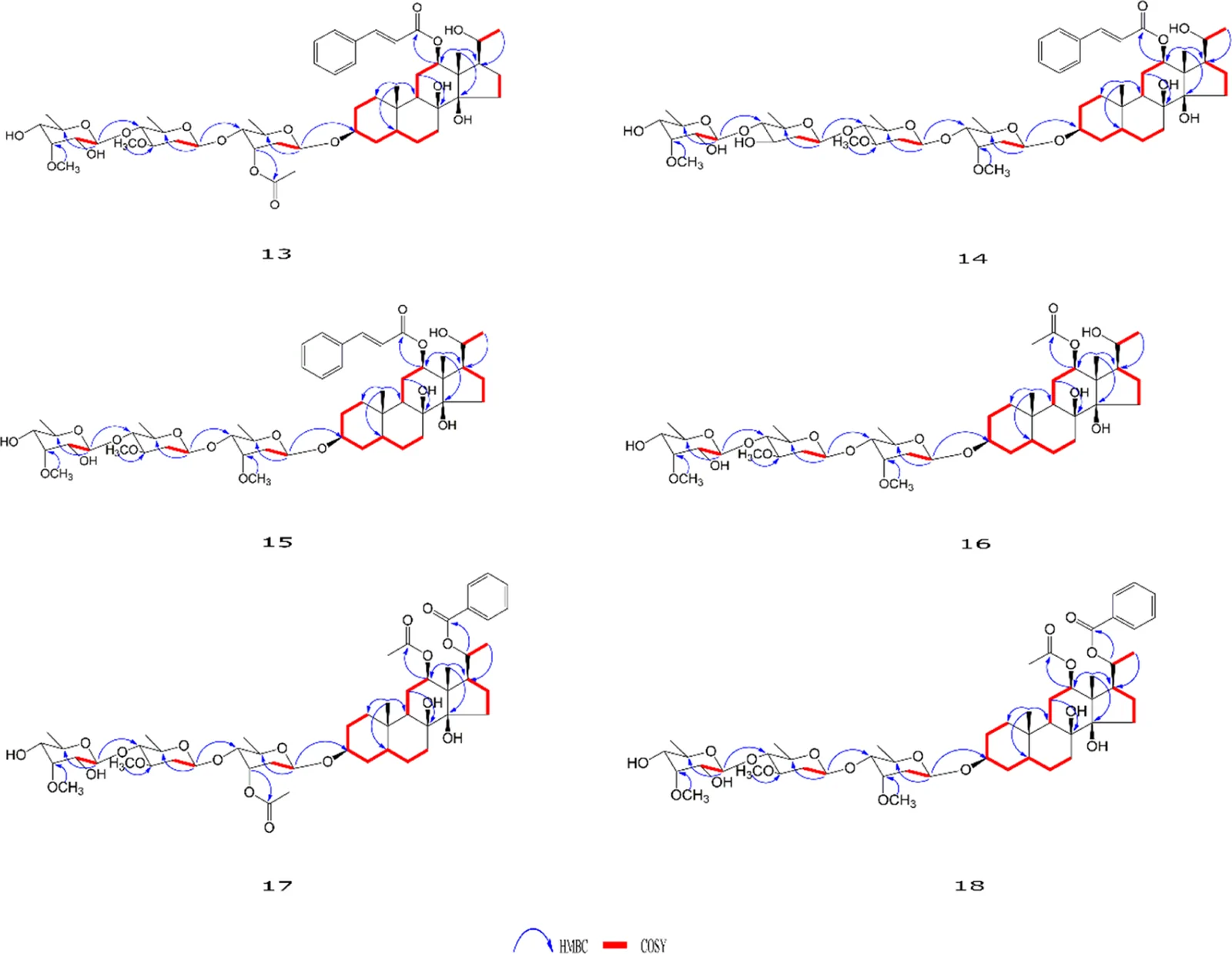
Continuation of Figure 4: key ^1^H–^13^C HMBC and ^1^H–^1^H COSY correlations of compounds **13**–**18**.

Compound **14** was obtained as white amorphous powder with an optical rotation of [α]_D_^20^ + 9.4 (c 0.10, MeOH). Its molecular formula was established as C_57_H_88_NaO_19_ based on HR-ESI-MS [M + Na]^+^ at *m*/*z* 1099.5812, calcd 1099.5812, Δ = 0.0 ppm, HDI = 14 (Figure S132). Analysis of the ^1^H and ^13^C NMR spectra (Tables 2, 7 and Figures S133–S135) of the sugar chain of **14** revealed signals corresponding to four anomeric protons at δ_H_ 5.28 (*dd*, *J* = 9.6, 1.6 Hz, H-1′), 4.66 (*dd*, *J* = 9.6, 1.6 Hz, H-1″), 4.90 (*d*, *J* = 10.4 Hz, H-1‴), and 5.08 (*d*, *J* = 8.4 Hz, H-1‴′), which correlated with carbon signals at δ_C_ 95.8 (C-1′), 101.8 (C-1″), 100.2 (C-1‴), and 103.2 (C-1‴′), respectively, as confirmed by HSQC correlations (Figure S136).

Comparison with NMR data reported in the literature^15,19–21,26^ allowed the sugar chain to be identified as *β*-d-cymarose, *β*-d-oleandrose, *β*-d-canarose, and *β*-D-6-deoxy-3-*O*-methyl-allose. The canaropyranosyl unit was characterized by NMR, and its D-configuration was assigned based on comparison with literature data and the predominant occurrence of d-canarose in pregnane glycosides from Apocynaceae.^6,23^ The sequence of these four sugars was confirmed by HMBC correlations (Figure 6 and S137): δ_H_ 5.28 (cymaropyranosyl, H-1′) with δ_C_ 76.6 (C-3), δ_H_ 4.66 (oleandropyranosyl, H-1″) with δ_C_ 83.5 (cymaropyranosyl, C-4′), δ_H_ 4.90 (canaropyranosyl, H-1‴) with δ_C_ 82.9 (oleandropyranosyl, C-4″), and δ_H_ 5.08 (6-deoxy-3-*O*-methyl-*β*-allopyranosyl, H-1‴′) with δ_C_ 88.2 (canaropyranosyl, C-4‴). Accordingly, the structure of compound **14** was established as 12-*O*-cinnamoyl-(20*S*)-5,6,17-trihydrosarcostin-3-*O*-*β*-D-6-deoxy-3-*O*-methyl-allopyranosyl-(1 → 4)-*β*-D-canaropyranosyl-(1 → 4)-*β*-d-oleandropyranosyl-*β*-d-cymaropyranoside (ruehssioside N).

Compound **15** was obtained as white amorphous powder with an optical rotation of [α]_D_^20^ + 8.6 (c 0.10, MeOH). Its molecular formula was established as C_51_H_78_NaO_16_ based on HR-ESI-MS [M + Na]^+^ at *m*/*z* 969.5137, calcd 969.5182, Δ = 4.7 ppm, HDI = 13 (Figure S142). The sugar chain of **15** was identified as being the same as that of compound **1** and was attached to the aglycone at C-3, as confirmed by 1D NMR (Tables 2, 6 and Figures S143–S145) and 2D NMR analyses (Figures 5, 6 and S146–S150). Accordingly, the structure of compound **15** was established as 12-*O*-cinnamoyl-(20*S*)-5,6,17-trihydrosarcostin-3-*O*-*β*-D-6-deoxy-3-*O*-methyl-allopyranosyl-(1 → 4)-*β*-d-oleandropyranosyl-(1 → 4)-*β*-d-cymaropyranoside (ruehssioside O).

Compound **16** was obtained as white amorphous powder with an optical rotation of [α]_D_^20^ + 19.6 (c 0.10, MeOH). Its molecular formula was established as C_44_H_74_NaO_16_ based on HR-ESI-MS [M + Na]^+^ at *m*/*z* 881.4853, calcd 881.4869, Δ = 1.8 ppm, HDI = 13 (Figure S152). The IR spectrum showed absorptions consistent with hydroxyl groups (3440 cm^–1^), ester carbonyl (1726 cm^–1^), aromatic rings (1640, 1447 cm^–1^), and ether groups (1065 cm^–1^) (Figure S151). Comparison of the ^1^H and ^13^C NMR spectra (Tables 7, 2 and Figures S153–S155) revealed that **16** possessed a C21 pregnane-type steroidal aglycone identical to that of compound **12**. However, the cinnamoyl group signals present in **12** were absent in **16**. Instead, signals corresponding to an acetyl unit were observed by the HMBC correlation (Figure S157) of δ_H_ 4.91 (H-12) with δ_C_ 171.0 (C—O) (Figure 6). The sugar chain of **16** was confirmed to be identical to that of compound **1** and was attached to the aglycone at C-3, as determined from 1D NMR (Tables 4 and 6) and 2D NMR analyses (Figures 5, 6 and S156–S160). Accordingly, the structure of compound **16** was established as 12-*O*-acetyl-(20*S*)-5,6,17-trihydrosarcostin-3-*O*-*β*-D-6-deoxy-3-*O*-methyl-allopyranosyl-(1 → 4)-*β*-d-oleandropyranosyl-(1 → 4)-*β*-d-cymaropyranoside (ruehssioside P).

Compound **17** was obtained as white amorphous powder with an optical rotation of [α]_D_^20^ + 42.6 (c 0.10, MeOH). Its molecular formula was established as C_52_H_78_NaO_18_ based on HR-ESI-MS [M + Na]^+^ at *m*/*z* 1013.5080, calcd 1013.5080, Δ = 0.1 ppm, HDI = 14 (Figure S162). The 1D (Tables 7, 2 and Figures S163–S165) and 2D NMR spectra (Figures 5, 6 and S166–S170) of **17** were similar to those of compound **16** but displayed additional signals consistent with a benzoyl unit: δ_H_ 8.34 (*d*, *J* = 5.6 Hz, H-3), 7.28 (*m*, H-4), 7.44 (*tt*, J = 5.6; 1.2 Hz, H-5), 7.28 (*m*, H-6), 8.34 (*d*, *J* = 5.6 Hz, H-7); δ_C_ 166.0 (C-1), 131.4 (C-2), 130.0 (C-3,7), 128.5 (C-4, 6), and 132.9 (C-5). This group was confirmed to be attached at C-12 based on the HMBC correlation (Figure S167) of δ_H_ 5.53 (H-20) with δ_C_ 166.0 (C-1, Bz). According to the literature, chemical shifts of C-20 and C-16 in 20-hydroxy-C/D-cis-pregnane steroids with a hydrogen at C-17 in the 20*R* configuration are typically: δ_C_ 71.0 and 27.0, respectively. These values are consistent with those of compound **17**, where C-20 and C-16 resonated at δ_C_ 71.7 and 23.5, respectively.^17,25^

The sugar chain of **17** was identified as being the same as that of compounds **3** and **13** and was attached to the aglycone at C-3, as confirmed by 1D (Tables 4 and 6) and 2D NMR analyses. Accordingly, the structure of compound **17** was determined as 12-*O*-acetyl-20-*O*-benzoyl-(20*R*)-5,6,17-trihydrosarcostin-3-*O*-*β*-D-6-deoxy-3-*O*-methyl-allopyranosyl-(1 → 4)-*β*-d-oleandropyranosyl-(1 → 4)-*β*-D-3′-*O*-acetyl-cymaropyranoside (ruehssioside Q).

Compound **18** was obtained as white amorphous powder with an optical rotation of [α]_D_^20^ + 11.2 (c 0.10, MeOH). Its molecular formula was established as C_51_H_78_NaO_17_ based on HR-ESI-MS [M + Na]^+^ at *m*/*z* 985.5114, calcd 985.5131, Δ = 1.7 ppm, HDI = 13 (Figure S172). Comparison of the 1D NMR signals (Tables 2, 7 and Figures S173–S175) and the key 2D correlations (Figures 5, 6 and S176–S180) of compounds **17** and **18** revealed that they shared the same aglycone, 12-*O*-acetyl-20-*O*-benzoyl-5,6,17-trihydrosarcostin. The sugar chain of **18** was determined to be identical to that of compound **16** and was attached to the aglycone at C-3, as confirmed by 1D (Tables 4 and 6) and 2D NMR analyses. Accordingly, the structure of compound **18** was established as 12-*O*-acetyl-20-*O*-benzoyl-(20*R*)-5,6,17-trihydrosarcostin-3-*O*-*β*-D-6-deoxy-3-*O*-methyl-allopyranosyl-(1 → 4)-*β*-d-oleandropyranosyl-(1 → 4)-*β*-d-cymaropyranoside (ruehssioside R).

### 2.3 Potential Anti-inflammatory Effects

First, the non-cytotoxic concentration range was determined by testing the cellular viability of macrophages treated with test compounds. Defining this non-cytotoxic concentration range before subsequent experiments is an important step as the reduction of cellular viability would implicate a reduction in inflammatory mediators production, which could be wrongly acknowledged as a possible anti-inflammatory effect. Present data show that compounds **1**, **2**, **3**, **4**, and **6** did not induce cytotoxic effects in J774 macrophages at concentrations up to 500 μM. Compounds **16** and **17** showed only mild cytotoxicity at the highest concentrations, with CC_50_ values > 500 μM (Table 8 and Figure 7). Compounds **7** and **13** did not exhibit cytotoxicity at the concentrations tested. However, due to the limited amount of purified material available, these compounds were evaluated only up to a maximum concentration of 250 μM, precluding direct comparisons of their cytotoxicity with the other compounds in the series. The remaining compounds exhibited variable cytotoxicity at the highest concentrations tested. Based on their CC_50_ values, the most cytotoxic compounds in the series were: **10**, **11**, and **15**. Importantly, the DMSO concentrations (0.5–0.0039%) used in this study did not affect cell viability, demonstrating that the vehicle itself did not interfere with the biological assays (see Figure 8).

**Table 8:** Cytotoxicity and Inhibitory Effect of Test Compounds on NO Production in Stimulated J774.A1 Macrophages

compounds	CC_50_ (μM)^a^	IC_50_ (μM)^b^	efficacy (%)^c^
1	>500	24.5	71.9
2	>500	32.1	71.8
3	>500	29.1	68.0
4	>500	136.9	40.0
6	>500	>250	32.1
7	>250	106.4	47.9
10	>62.5	17.5	66.1
11	>62.5	47.3	51.8
12	>250	111.4	62.0
13	>250	42.4	59.3
15	>62.5	33.7	62.9
16	>500	>62.5	33.4
17	>500	46.3	38.6
18	>125	66.2	48.8
Dexa^d^	>500	4.8	51.6

a CC_50_, half maximal cytotoxic concentration.

b IC_50_, half maximal inhibitory concentration.

c Efficacy: maximum effect (represented as % of the maximum possible effect).

d Dexa: gold standard drug, dexamethasone.

**Figure 7:**
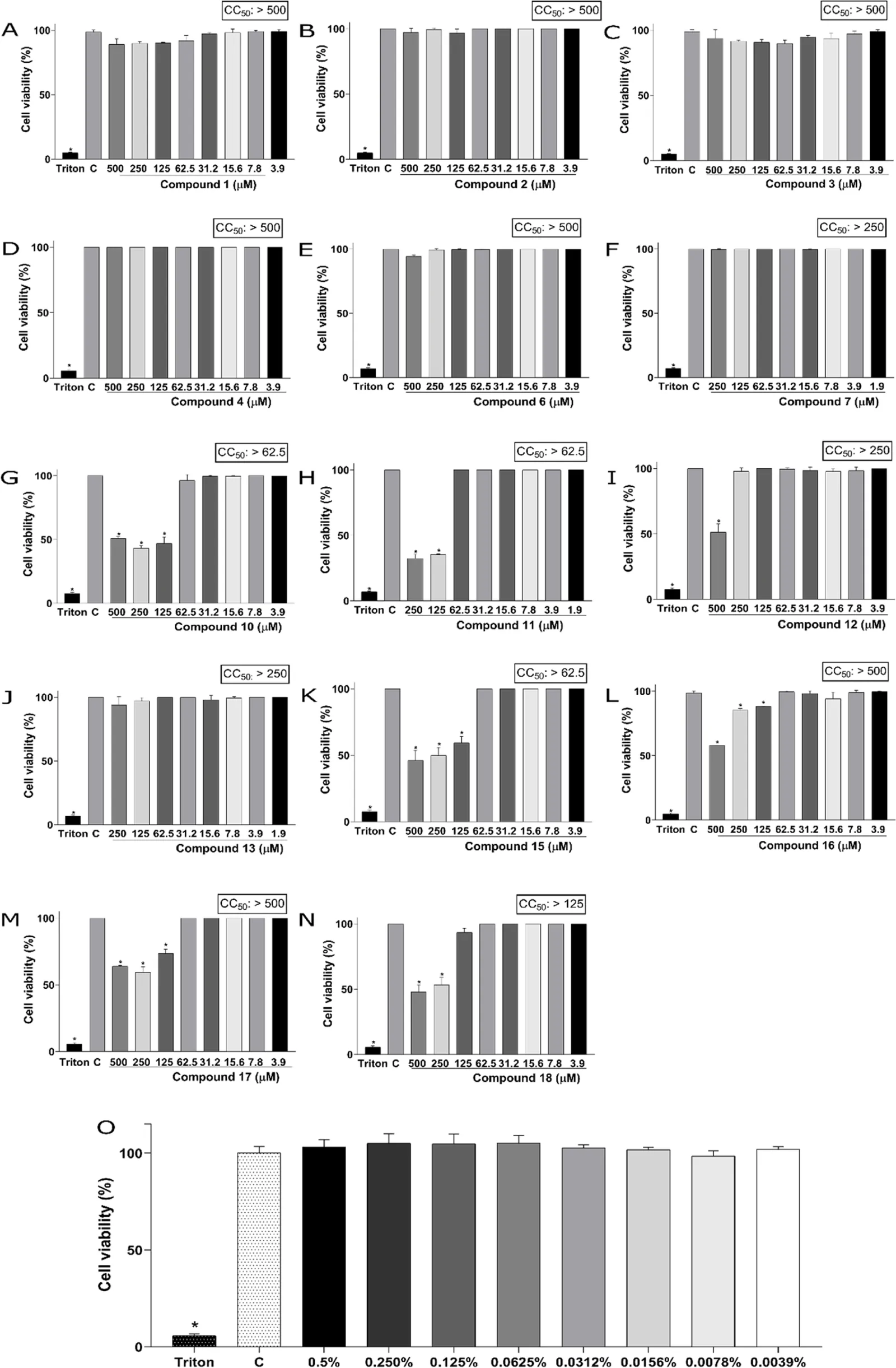
(A–N) Effect of compounds **1**–**4**, **6** and **7**, **10**–**13**, and **15**–**18** on the viability of J774.A1 macrophages. Different concentrations of the compounds (3.9–500 μM), DMSO (0.5–0.0039%), vehicle (C; control; 0.5% DMSO in saline), or 5% Triton (positive control) were added to macrophage cultures stimulated with LPS (500 ng/mL) + IFN-γ (5 ng/mL) and incubated for 24 h. Compounds **7**, **11**, and **13** were evaluated only up to a maximum concentration of 250 μM due to the limited amount of purified material available for testing. (O) The DMSO panel shows the effect of the vehicle alone (0.5–0.0039%) on J774.A1 macrophage viability under the same experimental conditions, demonstrating that the DMSO concentrations used did not affect cell viability. All data are expressed as mean ± standard deviation. *Significantly different from the C group (p < 0.05), as determined by ANOVA followed by Tukey’s test.

**Figure 8:**
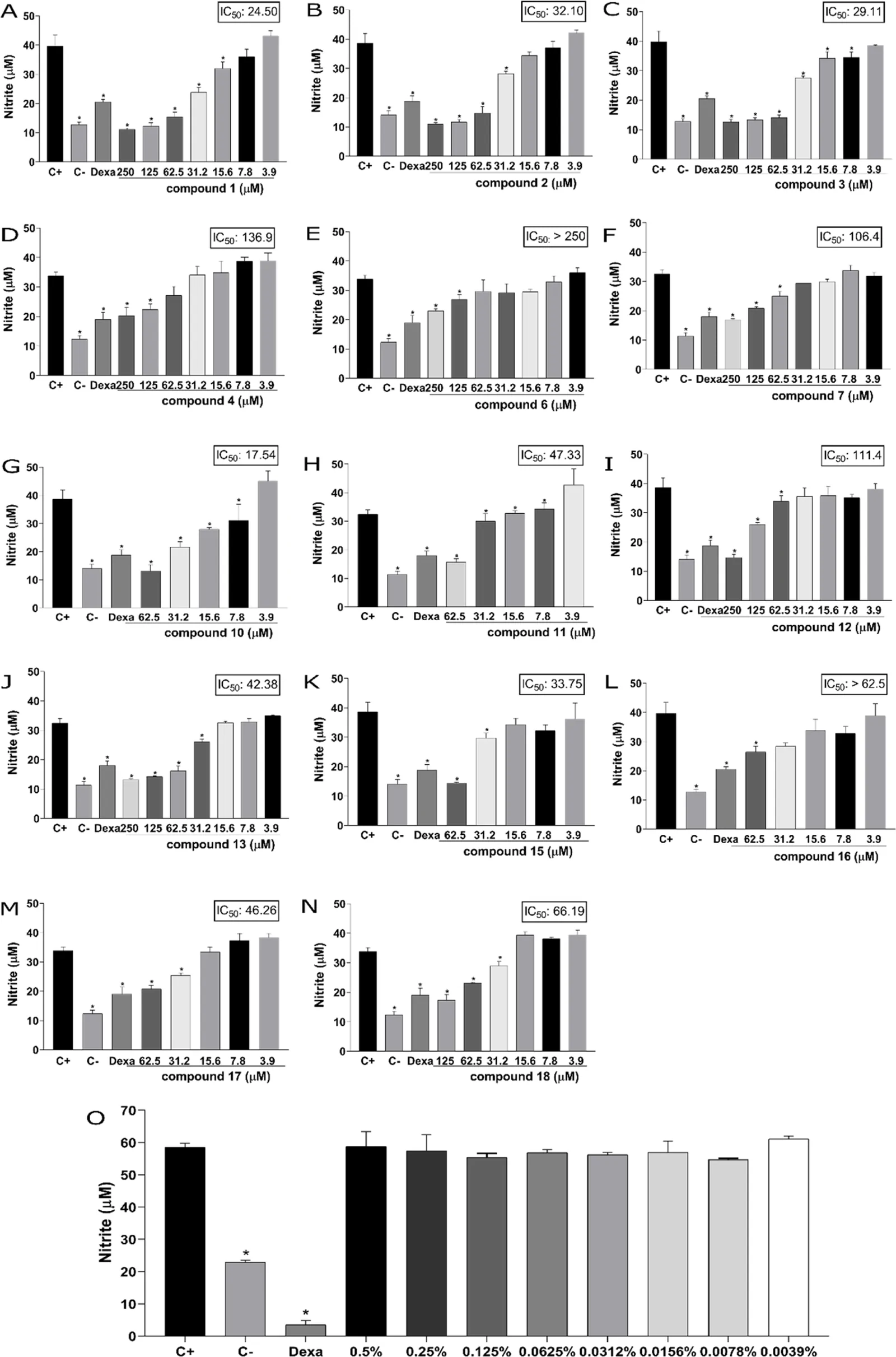
(A–N) Effect of compounds **1**–**4**, **6**, and **7**, **10**–**13**, and **15**–**18** on nitric oxide production by stimulated J774.A1 macrophages. The *X*-axis represents the different treatments, and the *Y*-axis represents nitrite levels in the culture supernatant. Cultures were stimulated with LPS (500 ng/mL) + IFN-γ (5 ng/mL) and treated with vehicle (C+; control group, 0.5% DMSO in saline), DMSO (0.5–0.0039%), test compounds, and dexamethasone (Dexa, 40 μM, reference drug). The compounds were tested over concentration ranges determined based on their cytotoxicity profiles. Compounds **10**, **11**, **15**, **16**, and **17** were evaluated at 3.9–62.5 μM, compound **18** at 3.9–125 μM, and the remaining compounds at 3.9–250 μM. The C– group represents unstimulated and untreated cells. (O) Effect of DMSO alone (0.5–0.0039%) on nitric oxide production by LPS/IFN-γ-stimulated J774.A1 macrophages under the same experimental conditions, demonstrating that the DMSO concentrations used in this study did not interfere with nitric oxide production. Data are expressed as mean ± SD from experiments performed in triplicate. Statistical analysis was conducted using one-way ANOVA followed by Tukey’s post hoc test. *p < 0.05 compared with the C+ group.

Next, the following assays were carried out within the noncytotoxic concentration range of each compound. The potential anti-inflammatory effects of the test compounds were assessed by using stimulated J774.A1 macrophages as an in vitro model. Macrophages are immune cells that are implicated in inflammation and immune regulation processes and contribute to tissue homeostasis.^27,28^ Upon activation by inflammatory stimuli, macrophages produce a myriad of pro-inflammatory mediators, such as chemokines, cytokines, and nitric oxide (NO).^27^ Therefore, the effect of compounds on the production of pro-inflammatory mediators by macrophages was evaluated.

Untreated macrophages stimulated with LPS and IFN-γ show increased levels of NO in comparison to unstimulated cells. The DMSO concentrations used in this study did not affect nitrite levels in the cell cultures, indicating that the vehicle did not interfere with the assessment of nitric oxide production. All compounds in the series were able to reduce the NO production by stimulated macrophages; however, compounds exhibited distinct profiles in the cell-based essay. From the dose–response curves of compounds, standardized by NO inhibition in J774 macrophage cultures, the IC_50_ and efficacy values were obtained. Compounds **12**, **4**, **6**, and **7** exhibited the lowest potency, with IC_50_ values above 100 μM. Compounds **11**, **13**, **16**, **17**, and **18** also exhibited low potency, with IC_50_ values ranging from 40 to 70 μM. Compounds **1**, **2**, **3**, and **15** exhibited moderate potency, with IC_50_ values between 20 and 35 μM, while compound **10** was the most potent of the series, with an IC_50_ of 17.5 μM. All compounds were less potent than dexamethasone (Dexa), the gold standard drug, with an IC_50_ of 4.8 μM (Table 8 and Figure 8).

Regarding pharmacological efficacy, that is, the maximum achievable response, the compounds presented a better profile. Only compounds **4**, **6**, **7**, **16**, **17**, and **18** had an efficacy of less than 50% in inhibiting NO production, thus lower than that of dexamethasone. The efficacy of compounds **3**, **10**, **11**, **12**, **13**, and **15** varied within the range of 50 to 70% inhibition, with values similar to or higher than those of Dexa. Compounds **1** and **2** exhibited the highest efficacies of the series with inhibition above 70%.

Compounds **1**, **2**, and **3** had moderate potency but the greatest efficacy, while compound **10** was the most potent, with slightly lower efficacy, although still superior to that of Dexa.

NO is a molecule with a ubiquitous role, but during inflammatory events, it will have mainly a pro-inflammatory role. NO will act as an oxidant agent or a signaling mediator, and it amplifies the inflammatory response by activating cascades that lead to the production of more inflammatory mediators.^29^ Thus, the ability to inhibit NO production by stimulated macrophages is a property that highlights the anti-inflammatory potential of new compounds. In this sense, the present results indicate that the investigated compounds have significant potential anti-inflammatory effects. In agreement with the present results, other compounds that share structural similarity with compounds **1** and **18** were found to have anti-inflammatory activity.^10,30–32^

To further evaluate the potential anti-inflammatory activity of test compounds, the levels of the proinflammatory cytokines IL-1β and IL-6 produced by activated macrophages were measured. Untreated macrophages stimulated with LPS and IFN-γ (Ct + group) showed increased levels of IL-1β (A) and IL-6 (B) compared to unstimulated cells, indicated as the Ct- group (Figure 9, p < 0.05). As observed for NO, the inhibitory effect of compounds on the release of IL-1β and IL-6 was also observed. Stimulated macrophages treated with **1**, **2**, **3**, **4**, **10**, **11**, **13**, **15**, **16**, **17**, and **18** at 62.5 μM showed a significant reduction in the production of IL-1β (p < 0.05, Figure 9A) when compared to stimulated untreated cells. Compounds **6**, **7**, and **12** did not reduce IL-1β production in stimulated macrophages. A similar inhibitory profile was observed for the effects of the test compounds on IL-6 production (p < 0.05, Figure 9B), except for compounds **4** and **16**, which reduced IL-1β production but not IL-6. The inhibitory effects of **1**, **2**, **3**, and **10** on IL-1β and IL-6 production were comparable to those of Dexa (40 μM), the gold-standard drug. Notably, compounds **1**, **2**, **3**, and **10** also exhibited the highest potency and efficacy in inhibiting NO production, further supporting their promising anti-inflammatory potential.

**Figure 9:**
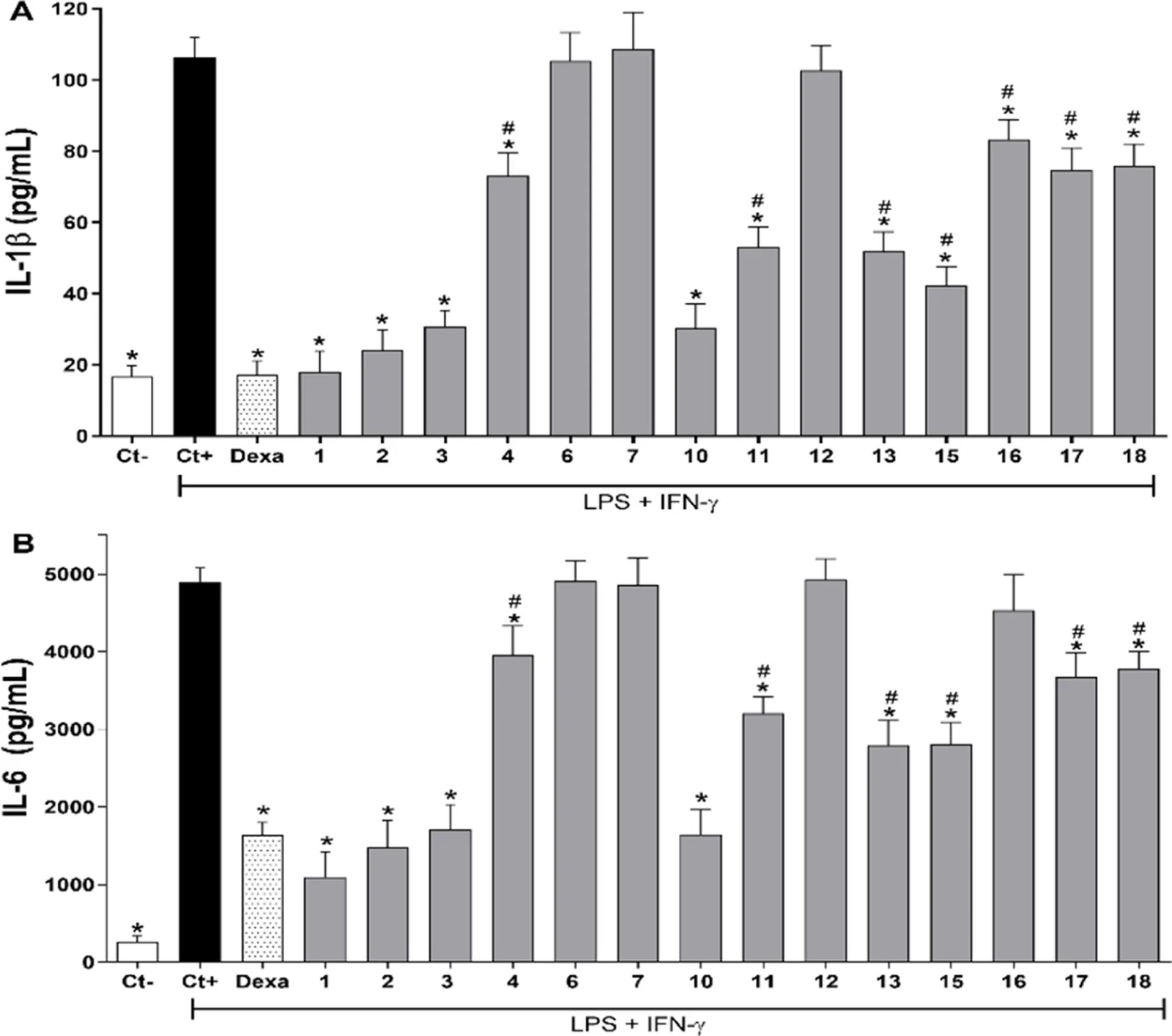
Inhibitory effect of compounds on the production of IL-1β and IL-6 by stimulated J774.A1 macrophages. Panels show quantification data of IL-1β (A) and IL-6 (B) by ELISA. Compounds (62.5 μM) or dexamethasone (Dexa; 40 μM, reference drug) were added to J774 macrophages cultures stimulated with LPS (500 ng/mL) + IFN-γ (5 ng/mL). The control group (Ct+) represents untreated cells stimulated with LPS + IFN-γ. The Ct- group shows data from untreated and unstimulated cells. IL-1β and IL-6 levels were determined 24 h after treatments. *Different from the control group (p < 0.05); ^#^different from the dexamethasone group (p < 0.05). Data are expressed as mean ± SD of 3 replicates. ANOVA one-way followed by Tukey’s test.

IL-1β and IL-6 are implicated as key cytokines in the inflammatory response, playing an essential role in the development of inflammatory signs and symptoms. Cytokines act as final mediators of inflammation and can induce their own production on a positive feedback loop that increases the inflammatory signaling.^33,34^ Enhanced secretion of IL-1β and IL-6 has been associated with the pathogenesis of autoimmune and cardiovascular diseases, metabolic syndromes, acute inflammation, chronic inflammation, and malignancy.^35–37^ In fact, the ability to decrease the levels of these cytokines during the inflammatory response is a relevant property of drugs with clinical success in controlling inflammatory diseases. Therefore, the ability of the compounds tested in the present study to inhibit the release of inflammatory cytokines supports their anti-inflammatory potential in this cell-based model.

### 2.4 Structure–Activity Relationship (SAR) of the Isolated Compounds

The analysis of the isolated compounds tested for biological activity (**1**–**4**, **6** and **7**, **10**–**13** and **15**–**18**) established a clear relationship between chemical structure, potency (IC_50_), efficacy, and cytotoxicity (CC_50_), allowing the identification of structural elements that are favorable or detrimental to the observed anti-inflammatory effects.

In this regard, compounds bearing sugar chains coded as S4 and S6 generally exhibited greater potency, with IC_50_ values below 35 μM. The most representative examples were compounds **1**, **3**, and **10**, with IC_50_ values of 24.5, 29.1, and 17.5 μM, respectively. In contrast, compounds containing sugar chains coded as S1 and S2 (e.g., compounds **6** and **7**) showed a marked loss of potency, with IC_50_ values ≥106.4 μM. These findings suggest that the reduced potency may be associated with the presence of terminal glucose residues and/or the higher number of sugar units in these sugar chains, as observed in S1 and S2. In addition, compounds bearing acyl substituents on the sugar moieties (compounds **3**, **13**, and **17**) generally exhibited moderate to high potency in the cell-based assay. However, compound **7**, despite also containing an acyl substituent on the sugar moiety, displayed lower potency. This finding suggests that the potential beneficial effect of acylation may be counterbalanced by the S2 sugar chain, particularly by the presence of terminal glucose residues and the higher number of sugar units, which appear to exert a greater negative influence on activity than acylation itself. These results demonstrate that substitution at C-3 exerts a strong influence on the biological activity (Table 8 and Figure 10), which may be related to compound availability in the biophase or affinity for a potential molecular target. Further studies are required to confirm these hypotheses.

**Figure 10:**
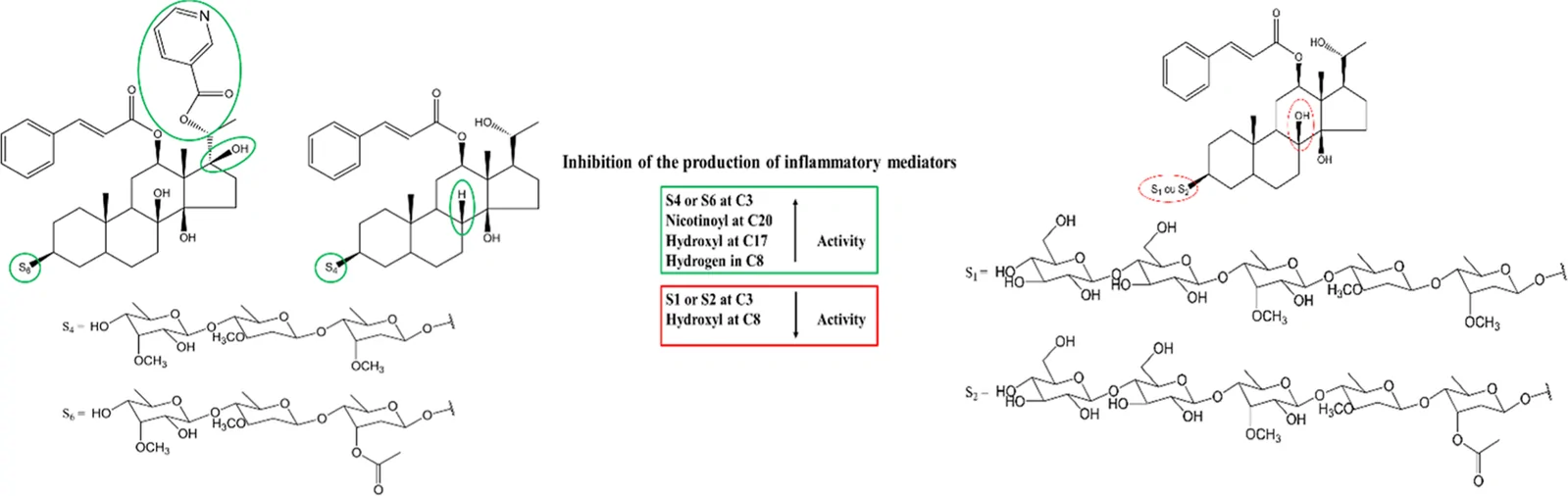
Structure–activity relationship of isolated pregnane glycosides.

Regarding substitution at C-20, the presence of a nicotinoyl (Nic) group, a hydroxyl at C-17 in compounds **1**, **2**, and **3**, and a hydrogen at C-8 in compound **10** proved to be critical for enhancing potency and efficacy. Indeed, compounds **1**, **2**, **3**, and **10** displayed the highest efficacy values (71.9, 68.0, 71.8, and 66.1%, respectively) and strong potency, with compound **10** being the most potent of the series (IC_50_ = 17.5 μM). Reports in the literature have also described pregnane glycosides evaluated in similar models, in which inhibition of NO production in LPS-stimulated macrophages was associated with anti-inflammatory effects.^38–40^

Regarding cytotoxicity, most pregnane glycosides exhibited a favorable safety profile, indicating that the structural modifications within this series were generally well tolerated. Compounds bearing a nicotinoyl group at R4 and a hydroxyl group at C-17 exhibited low cytotoxicity (CC_50_ > 500 μM), suggesting that these substituents are compatible with a favorable safety profile. Likewise, compounds containing a benzoyl group at R4 generally showed low cytotoxicity, although the lower CC_50_ threshold observed for compound **18** suggests that the influence of this substituent may depend on the overall molecular framework rather than on the acyl group itself. The highest cytotoxicity within the series was observed for compounds **10**, **11**, and **15**, suggesting that the absence of an acyl substituent at C-20 (R4 = H) may negatively influence the safety profile of these pregnane glycosides. In contrast, variations in the sugar moiety (R1) and substitutions at R2 and R3 were not consistently associated with cytotoxicity, indicating that structural modifications at C-20 exert a greater influence on this property. However, additional studies with a larger number of analogues are required to confirm these preliminary structure–toxicity relationships.

Taken together, the data indicate that modifications at R1 and R4 exert the greatest influence on the biological effects observed in the cell-based assays. The presence of R1 = S4 or S6 combined with R4 = nicotinoyl results in more potent and effective derivatives (Table 8 and Figure 10). These findings provide a rational basis for future structural optimization aimed at obtaining more promising compounds.

## 3 Conclusion

This study reports the isolation and structural characterization of 18 previously undescribed pregnane glycosides from the roots of *R. caatingae*, named ruehssiosides A–R (**1**–**18**). Among these, 14 compounds were assessed for potential anti-inflammatory effects in activated macrophage models, exhibiting low cytotoxicity and effective inhibition of IL-1β, IL-6, and nitric oxide production. Notably, compound 10 emerged as the most potent derivative (IC_50_ = 17.5 μM). Structure–activity relationship analysis highlighted the critical role of substitutions at C-3 and C-20, as well as the presence of a hydrogen at C-8, in enhancing potency and efficacy.

This work constitutes the first report of pregnane glycosides in the genus *Ruehssia* and underscores the Caatinga biome as a promising source of structurally unique and biologically active metabolites. Moreover, this reinforces the relevance of the Apocynaceae family as a valuable source of new anti-inflammatory agents. This body of evidence supports a rational framework for further investigations, encompassing mechanistic studies, molecular modeling, and the design of synthetic analogues, to advance pregnane glycosides as potential therapeutic leads.

## 4 Materials and Methods

### 4.1 General Experimental Procedures

The specific rotations of the substances were measured on a JASCO Polarimeter, model P-200. NMR experiments were carried out in pyridine-d_5_ on Bruker AVANCE III HD spectrometers (500/400 and 125/100 MHz for ^1^H and ^13^C, respectively), using TMS as the internal standard. High-resolution electrospray ionization mass spectra (HR-ESI-MS) were recorded on a MicroTOF II Bruker Daltonics mass spectrometer (Billerica, MA, USA) in the positive-ion mode. The instrument was calibrated using ESI TuneMix prior to analysis to ensure mass accuracy. Samples were dissolved in MeOH/H_2_O (1:1, v/v) and analyzed without the addition of 0.1% (v/v) formic acid. The capillary voltage was set at 4.5 kV, with an end plate offset of 500 V, a nebulizer pressure of 40 psi, a dry gas (N_2_) flow rate of 8 L/min, and a source temperature of 200 °C. Mass spectra were acquired in the *m*/*z* range of 50 a 3000. ATR-IR spectra were obtained on a Fourier-transform infrared spectrophotometer (FTIR, IRSpirit-X, Shimadzu). HPLC analyses were performed on a Shimadzu Prominence liquid chromatograph equipped with a diode array detector (DAD) SPD-M10A, monitored at 205 and 254 nm, using a YMC Actus Triart C-18 column (250 mm × 4.6 mm i.d., 5 μm particle size) coupled to a Security Guard Gemini C-18 pre-column (4 mm × 4.6 mm i.d., 5 μm particle size) for preparative scale separations. Column chromatography (CC) was conducted on Merck silica gel 7734 (particle size 0.063–0.200 mm) and Sephadex LH-20 (Merck). Thin-layer chromatography (TLC) was performed on Merck silica gel 70 plates containing a fluorescent indicator F_254_.

### 4.2 Plant Material

The roots of *R. caatingae* were collected in Pocinhos, state of Paraiba, Brazil. The plant material was identified by Prof. Dr. José Iranildo Miranda de Melo, and a voucher specimen was deposited in the Manuel de Arruda Câmara Herbarium at the State University of Paraíba under the registration code HACAM 662. This study was registered in the National System for the Management of Genetic Heritage and Associated Traditional Knowledge (SISGEN) under the number A5B0BFC.

### 4.3 Extraction and Isolation

Dried roots of *R. caatingae* (450 g), obtained after oven-drying with circulating air, were ground and subjected to maceration (3 × 72 h each) with 95% ethanol (21.0 L). The extractive solution was concentrated under reduced pressure by using a rotary evaporator to afford a crude ethanolic extract (32 g). The extract (31 g) was dissolved in an EtOH/H_2_O mixture (7:3, v/v) and partitioned successively with hexane (Hex), chloroform (CHCl_3_), ethyl acetate (EtOAc), and n-butanol (*n*-BuOH), yielding the corresponding phases.

The chloroform phase (5 g) was subjected to Sephadex column chromatography (CC) and eluted with methanol (MeOH), affording 40 fractions (Fr-1–Fr-40), which were combined according to their TLC retention profiles. The pooled fractions Fr-9–18 (3 g) were further chromatographed on silica gel CC, eluted with pure Hex, EtOAc, and MeOH as well as binary mixtures of increasing polarity, yielding 14 fractions (Fr-9–18.1–Fr-9–18.14), which were combined based on TLC analysis.

Fractions Fr-9–18.9 and Fr-9–18.10 (304 mg) were subjected to preparative HPLC using ACN/H_2_O (0.1% formic acid, v/v) as the mobile phase under the following gradient: 5–29% ACN (5 min), 29% ACN (25 min), 29–43% ACN (5 min), 43% ACN (20 min), 43–52% ACN (5 min), 52% ACN (30 min), and 52–100% ACN (5 min). This procedure afforded compounds **6** (Rt = 74 min, 4.5 mg), **7** (Rt = 75 min, 1.8 mg), **8** (Rt = 72 min, 1.0 mg) and **9** (Rt = 71 min, 1.0 mg).

Fraction Fr-9–18.3 (1426 mg) was chromatographed on silica gel CC, eluted with pure Hex, EtOAc, and MeOH as well as binary mixtures of increasing polarity, yielding 18 fractions (Fr-9–18.3.1–Fr-9–18.3.18), which were combined based on TLC.

Fraction Fr-9–18.3.7 (410 mg) was further purified by preparative HPLC using ACN/H_2_O (0.1% formic acid, v/v) as the mobile phase under the following gradient: 5–55% ACN (20 min), 55–59% ACN (10 min), 59% ACN (20 min), 59–71% ACN (10 min), 71% ACN (20 min), and 71–100% ACN (10 min). This procedure afforded compounds **1** (Rt = 26 min, 12 mg), **2** (Rt = 30 min, 15.3 mg), **3** (Rt = 29 min, 3.6 mg), **4** (Rt = 65 min, 6.2 mg), **5** (Rt = 27 min, 2.2 mg), **10** (Rt = 45 min, 2.6 mg), **11** (Rt = 42 min, 1.4 mg), **12** (Rt = 38 min, 1.4 mg), **13** (Rt = 48 min, 1.0 mg), **14** (Rt = 55 min, 1.7 mg), **15** (Rt = 50 min, 6.5 mg), **16** (Rt = 22 min, 5.4 mg), **17** (Rt = 58 min, 6.0 mg), and **18** (Rt = 67 min, 2.2 mg).

### 4.4 Cytotoxicity to Mammalian Cells

To determine the cytotoxicity of compounds **1**–**4**, **6**–**7**, **10**–**13**, and **15**–**18**, murine macrophage-like J774.A1 cells were seeded into 96-well plates at a density of 2 × 10^5^ cells per well in Dulbecco’s Modified Eagle’s Medium (DMEM; Life Technologies, GIBCO-BRL, Gaithersburg, MD, USA) supplemented with 10% fetal bovine serum (FBS; GIBCO) and 50 μg/mL gentamicin (Novafarma, Anápolis, GO, Brazil). Cells were incubated for 72 h at 37 °C in a 5% CO_2_ atmosphere, as previously described.^41^ They were then stimulated with LPS (500 ng/mL, Sigma Chemical Co., St. Louis, MO, USA) and IFN-γ (5 ng/mL, Sigma). Compounds were added to the medium at eight concentrations ranging from 1.9 to 500 μM, in triplicate, and incubated for 24 h. Subsequently, 20 μL per well of Alamar Blue (Invitrogen, Carlsbad, CA, USA) was added to the plates for 6 h. Compounds **7**, **11**, and **13** were evaluated only up to a maximum concentration of 250 μM due to the limited amount of purified material available for testing. Colorimetric readings were performed at 570 and 600 nm. Triton X-100 (5%) was used as the positive control.

### 4.5 Assessment of Cytokine and Nitric Oxide Production by Macrophages

For cytokine and nitric oxide evaluations, J774.A1 cells were seeded in 96-well tissue culture plates at 2 × 10^5^ cells per well in DMEM supplemented with 10% FBS and 50 μg/mL gentamicin and incubated for 2 h at 37 °C in a 5% CO_2_ atmosphere, as previously described.^41^ Cells were then stimulated with LPS (500 ng/mL) and IFN-γ (5 ng/mL) in the presence of either the test compounds at different concentrations (3.9–250 μM), the medium alone, or dexamethasone (40 μM, reference drug) and further incubated at 37 °C. Cell-free supernatants were collected 24 h after incubation for IL-1β, IL-6, and nitrite quantification. Cytokine concentrations in the supernatants of J774.A1 cultures were determined by enzyme-linked immunosorbent assay (ELISA) using the DuoSet kit (R&D Systems, Minneapolis, MN, USA), according to the manufacturer’s instructions. Results were expressed as picograms per milliliter (pg/mL) of IL-1β or IL-6. Quantification of nitrite as an indicator of nitric oxide production was performed using the Griess method.^42^ Three independent experiments were performed.^43–45^

### 4.6 Statistical Analysis

Data are presented as means ± standard deviation (SD). Comparisons between treatments were performed using one-way ANOVA followed by Tukey’s post hoc test. CC_50_ (half-maximal cytotoxic concentration) and IC_50_ (half-maximal inhibitory concentration) values were calculated from concentration–effect curves obtained in the cytotoxicity and nitrite quantification assays, respectively. All data were analyzed using Prism 9 software (GraphPad, San Diego, CA, USA). Differences were considered statistically significant at *p* < 0.05.

(ruehssioside A): (**1**) white amorphous powder; [α]_D_^20^ + 78.7 (c 0.10, MeOH); IR *v*_max_ 3432, 2932, 1717, 1637, 1594, 1453, 1372, and 1166 cm^–1^ (Figure S1), ^1^H NMR (C_5_D_5_N, 500 MHz) (Table 1); ^13^C NMR (C_5_D_5_N, 125 MHz) (Table 2), positive-ion HR-ESI-MS *m*/*z* 1066.5348 [M + H]^+^ (calcd for C_57_H_80_NO_18_, *m*/*z* 1066.5370, Δ = 2.1 ppm).
(ruehssioside B): (**2**) white amorphous powder; [α]_D_^20^ + 95.3 (c 0.10, MeOH); IR *v*_max_ 3423, 2932, 1708, 1640, 1594, 1447, 1372, 1166, and 1077 cm^–1^ (Figure S11), ^1^H NMR (C_5_D_5_N, 500 MHz) (Table 1); ^13^C NMR (C_5_D_5_N, 125 MHz) (Table 2), positive-ion HR-ESI-MS *m*/*z* 1068.5499 [M + H]^+^ (calcd for C_57_H_82_NO_18_, *m*/*z* 1068.5526, Δ = 2.6 ppm).
(ruehssioside C): (**3**) white amorphous powder; [α]_D_^20^ + 87.5 (c 0.10, MeOH); IR *v*_max_ 3452, 2935, 1717, 1637, 1594, 1453, 1372, and 1166 cm^–1^ (Figure S21) ^1^H NMR (C_5_D_5_N, 400 MHz) (Table 1); ^13^C NMR (C_5_D_5_N, 100 MHz) (Table 2), positive-ion HR-ESI-MS *m*/*z* 1118.5291 [M + Na]^+^ (calcd for C_58_H_81_NNaO_19_, 1118.5295, Δ = 0.3 ppm).
(ruehssioside D): (**4**) white amorphous powder; [α]_D_^20^ + 57.2 (c 0.10, MeOH); IR *v*_max_ 3455, 2935, 1711, 1640, 1453, 1372, and 1166 cm^–1^ (Figure S31), ^1^H NMR (C_5_D_5_N, 500 MHz) (Table 1); ^13^C NMR (C_5_D_5_N, 125 MHz) (Table 2), positive-ion HR-ESI-MS *m*/*z* 1089.5383 [M + Na]^+^ (calcd for C_58_H_82_NaO_18_, 1089.5393, Δ = 0.9 ppm).
(ruehssioside E): (**5**) white amorphous powd.er; [α]_D_^20^ + 15.4 (c 0.10, MeOH); IR *v*_max_ 3449, 2935, 1731, 1711, 1640, 1453, 1372, 1166, and 1065 cm^–1^ (Figure S41) ^1^H NMR (C_5_D_5_N, 400 MHz) (Table 1); ^13^C NMR (C_5_D_5_N, 100 MHz) (Table 2), positive-ion HR-ESI-MS *m*/*z* 1027.5208 [M + Na]^+^ (calcd for C_53_H_80_NaO_18_, 1027.5237, Δ = 2.8 ppm).
(ruehssioside F): (**6**) white amorphous powder; [α]_D_^20^ + 30.5 (c 0.10, MeOH); IR *v*_max_ 3483, 2932, 1708, 1637, 1594, 1450, 1312, 1166, and 1074 cm^–1^ (Figure S51) ^1^H NMR (C_5_D_5_N, 400 MHz) (Table 1); ^13^C NMR (C_5_D_5_N, 100 MHz) (Table 2), positive-ion HR-ESI-MS *m*/*z* 1293.6199 [M + Na]^+^ (calcd for C_63_H_98_NaO_26_, 1293.6239, Δ = 3.0 ppm).
(ruehssioside G): (**7**) white amorphous powder; [α]_D_^20^ + 81.6 (c 0.10, MeOH); IR *v*_max_ 3389, 2932, 1737, 1708; 1637, 1594, 1453, 1372, 1166, and 1065 cm^–1^ (Figure S61) ^1^H NMR (C_5_D_5_N, 400 MHz) (Table 5); ^13^C NMR (C_5_D_5_N, 100 MHz) (Table 2), positive-ion HR-ESI-MS *m*/*z* 1321.6167 [M + Na]^+^ (calcd for C_64_H_98_NaO_27_, 1321.6188, Δ = 1.6 ppm).
(ruehssioside H): (**8**) white amorphous powder; [α]_D_^20^ – 12.4 (c 0.10, MeOH); IR *v*_max_ 3383, 2918, 2852, 1711, 1637, 1576, 1539; 1453, 1370, 1166, and 1062 cm^–1^ (Figure S71) ^1^H NMR (C_5_D_5_N, 400 MHz) (Table 5); ^13^C NMR (C_5_D_5_N, 100 MHz) (Table 2), positive-ion HR-ESI-MS *m*/*z* 1277.6269 [M + Na]^+^ (calcd for C_63_H_98_NaO_25_, 1277.6289, Δ = 1.6 ppm).
(ruehssioside I): (**9**) white amorphous powder; [α]_D_^20^ + 10.4 (c 0.10, MeOH); IR *v*_max_ 3383, 2926, 2854, 1708, 1637, 1594, 1453, 1372, 1166, and 1074 cm^–1^ (Figure S81) ^1^H NMR (C_5_D_5_N, 400 MHz) (Table 5); ^13^C NMR (C_5_D_5_N, 100 MHz) (Table 2), positive-ion HR-ESI-MS *m*/*z* 1275.6097 [M + Na]^+^ (calcd for C_63_H_96_NaO_25_, 1275.6133, Δ = 2.8 ppm).
(ruehssioside J): (**10**) white amorphous powder; [α]_D_^20^ – 5.8 (c 0.10, MeOH); IR *v*_max_ 3426, 2932, 1708, 1640, 1447, 1372, 1166, and 1062 cm^–1^ (Figure S91) ^1^H NMR (C_5_D_5_N, 400 MHz) (Table 5); ^13^C NMR (C_5_D_5_N, 100 MHz) (Table 2), positive-ion HR-ESI-MS *m*/*z* 953.5219 [M + Na]^+^ (calcd for C_51_H_78_NaO_15_, 953.5233, Δ = 1.5 ppm).
(ruehssioside K): (**11**) white amorphous powder; [α]_D_^20^ – 3.4 (c 0.10, MeOH); IR *v*_max_ 3429, 2932, 1708; 1637, 1447, 1370, 1312, 1163, and 1062 cm^–1^ (Figure S101) ^1^H NMR (C_5_D_5_N, 400 MHz) (Table 5); ^13^C NMR (C_5_D_5_N, 100 MHz) (Table 2), positive-ion HR-ESI-MS *m*/*z* 967.5007 [M + Na]^+^ (calcd for C_51_H_76_NaO_16_, 967.5026, Δ = 2.0 ppm).
(ruehssioside L): (**12**) white amorphous powder; [α]_D_^20^ + 23.4 (c 0.10, MeOH); IR *v*_max_ 3443, 2932, 1708, 1640, 1596, 1447, 1372, 1166, and 1062 cm^–1^ (Figure S111) ^1^H NMR (C_5_D_5_N, 400 MHz) (Table 5); ^13^C NMR (C_5_D_5_N, 100 MHz) (Table 2), positive-ion HR-ESI-MS *m*/*z* 955.5000 [M + Na]^+^ (calcd for C_50_H_76_NaO_16_, 955.5026, Δ = 2.7 ppm).
(ruehssioside M): (**13**) white amorphous powder; [α]_D_^20^ + 59.7 (c 0.10, MeOH); IR *v*_max_ 3426, 2932, 1737, 1706; 1637, 1450, 1372, 1166, and 1062 cm^–1^ (Figure S121) ^1^H NMR (C_5_D_5_N, 400 MHz) (Table 4); ^13^C NMR (C_5_D_5_N, 100 MHz) (Table 2), positive-ion HR-ESI-MS *m*/*z* 997.5124 [M + Na]^+^ (calcd for C_52_H_78_NaO_17_, 997.5131, Δ = 0.7 ppm).
(ruehssioside N): (**14**) white amorphous powder; [α]_D_^20^ + 9.4 (c 0.10, MeOH); IR *v*_max_ 3423, 2932, 1708; 1640, 1447, 1375, 1166, and 1065 cm^–1^ (Figure S131) ^1^H NMR (C_5_D_5_N, 400 MHz) (Table 4); ^13^C NMR (C_5_D_5_N, 100 MHz) (Table 2), positive-ion HR-ESI-MS *m*/*z* 1099.5812 [M + Na]^+^ (calcd for C_57_H_88_NaO_19_, 1099.5812, Δ = 0.0 ppm).
(ruehssioside O): (**15**) white amorphous powder; [α]_D_^20^ + 8.6 (c 0.10, MeOH); IR *v*_max_ 3417, 2932, 1708; 1640, 1447, 1372, 1166, and 1062 cm^–1^ (Figure S141) ^1^H NMR (C_5_D_5_N, 500 MHz) (Table 4); ^13^C NMR (C_5_D_5_N, 125 MHz) (Table 2), positive-ion HR-ESI-MS *m*/*z* 969.5137 [M + Na]^+^ (calcd for C_51_H_78_NaO_16_, 969.5182, Δ = 4.7 ppm).
(ruehssioside P): (**16**) white amorphous powder; [α]_D_^20^ + 19.6 (c 0.10, MeOH); IR *v*_max_ 3440, 2935, 1726; 1640, 1594, 1447, 1381, and 1163 cm^–1^ (Figure S151) ^1^H NMR (C_5_D_5_N, 500 MHz) (Table 4; ^13^C NMR (C_5_D_5_N, 125 MHz) (Table 2), positive-ion HR-ESI-MS *m*/*z* 881.4853 [M + Na]^+^ (calcd for C_44_H_74_NaO_16_, 881.48694, Δ = 1.8 ppm).
(ruehssioside Q): (**17**) white amorphous powder; [α]_D_^20^ + 42.6 (c 0.10, MeOH); IR *v*_max_ 3463, 2932, 1731, 1717, 1637, 1605, 1453, 1372, 1163, and 1065 cm^–1^ (Figure S161) ^1^H NMR (C_5_D_5_N, 500 MHz) (Table 4); ^13^C NMR (C_5_D_5_N, 125 MHz) (Table 2), positive-ion HR-ESI-MS *m*/*z* 1013.5080 [M + Na]^+^ (calcd for C_52_H_78_NaO_18_, 1013.5080, Δ = 0.1 ppm).
(ruehssioside R): (**18**) white amorphous powder; [α]_D_^20^ + 11.2 (c 0.10, MeOH); IR *v*_max_ 3501, 2932, 1734, 1717, 1602, 1453, 1370, 1163, and 1065 cm^–1^ (Figure S171) ^1^H NMR (C_5_D_5_N, 400 MHz) (Table 4); ^13^C NMR (C_5_D_5_N, 100 MHz) (Table 2), positive-ion HR-ESI-MS *m*/*z* 985.5114 [M + Na]^+^ (calcd for C_51_H_78_NaO_17_, 985.5131, Δ = 1.7 ppm).

## Supplementary Material



## Notes

ATR-infrared spectra of compounds 1–18; HR-ESI-MS spectra; and 1D and 2D NMR spectra (^1^H, ^13^C-BB, ^13^C-DEPT 135, HSQC, HMBC, COSY, TOCSY, and NOESY) (PDF)
