# Supplementary material for: Ruehssiosides A–R, 18 Previously Undescribed Pregnane Glycosides from Ruehssia caatingae: Structural Elucidation, Potential Anti-Inflammatory Activity, and Insights into Structure–Activity Relationships
Source: ACS Omega. 2026 Sep 16;11(38):56866–90. doi: 10.1021/acsomega.6c02994 (PMC13629022; doi:10.1021/acsomega.6c02994)

SUPPLEMENTARY MATERIAL

Ruehssiosides A–R, eighteen previously undescribed pregnane glycosides from *Ruehssia caatingae*: structural elucidation, potential anti-inflammatory activity and insights into structure-activity relationships

Jociano da Silva Lins[a], Thalisson Amorim de Souza[a], Karoline Cristina Jatobá da Silva[d], Arthur Ribeiro de Souza[c], Cristiane Flora Villarreal[d], Lucas Silva Abreu[c], Fernando Martins dos Santos Junior[c], José Iranildo Miranda de Melo[b], Marcus Tullius Scotti[a], Marcelo Sobral da Silva[a], Josean Fechine Tavares[a*], José Maria Barbosa Filho[a].

[a]Federal University of Paraiba, Program of Post-graduation on Natural and Synthetic Bioactive Products (PgPNSB), Health Sciences Center, 58050-585, João Pessoa-PB, Brazil

[b]Postgraduate Program in Ecology and Conservation, State University of Paraiba, 58429-500, Campina Grande-PB, Brazil

[c]Department of Organic Chemistry, Fluminense Federal University, 24020-141, Niteroi-RJ, Brazil

[d]Faculty of Pharmacy, Federal University of Bahia, 40170-115, Salvador-BA, Brazil

*Corresponding author: Prof. Dr. Josean Fechine Tavares, Campus I Lot. Cidade Universitária, PB, 58051-900. E-mail: josean@ltf.ufpb.br

LIST OF FIGURES

Figure S1. ATR-Infrared spectrum of compound 1..9

Figure S2. HR-ESI-MS mass spectrum of compound 1..9

Figure S3. ^{1}H NMR spectrum (500 MHz, C_5D_5N) of compound 1.................................10

Figure S4. ^{13}C-BB NMR spectrum (125 MHz, C_5D_5N) of compound 1..........................10

Figure S5. ^{13}C-DEPT 135 NMR spectrum (125 MHz, C_5D_5N) of compound 1..............11

Figure S6. HSQC spectrum (500 x 125 MHz, C_5D_5N) of compound 1.........................11

Figure S7. HMBC spectrum (500 x 125 MHz, C_5D_5N) of compound 1........................12

Figure S8. COSY spectrum (500 x 500 MHz, C_5D_5N) of compound 1............................12

Figure S9. TOCSY spectrum (500 x 500 MHz, C_5D_5N) of compound 1.........................13

Figure S10. NOESY spectrum (500 x 500 MHz, C_5D_5N) of compound 1.......................13

Figure S11. ATR-Infrared spectrum of compound 2..14

Figure S12. HR-ESI-MS mass spectrum of compound 2...14

Figure S13. ^{1}H NMR spectrum (500 MHz, C_5D_5N) of compound 2...............................15

Figure S14. ^{13}C-BB NMR spectrum (125 MHz, C_5D_5N) of compound 2........................15

Figure S15. ^{13}C-DEPT 135 NMR spectrum (125 MHz, C_5D_5N) of compound 2............16

Figure S16. HSQC spectrum (500 x 125 MHz, C_5D_5N) of compound 2.......................16

Figure S17. HMBC spectrum (500 x 125 MHz, C_5D_5N) of compound 2......................17

Figure S18. COSY spectrum (500 x 500 MHz, C_5D_5N) of compound 2..........................17

Figure S19. TOCSY spectrum (500 x 500 MHz, C_5D_5N) of compound 2.......................18

Figure S20. NOESY spectrum (500 x 500 MHz, C_5D_5N) of compound 2.......................18

Figure S21. ATR-Infrared spectrum of compound 3..19

Figure S22. HR-ESI-MS mass spectrum of compound 3...19

Figure S23. ^{1}H NMR spectrum (400 MHz, C_5D_5N) of compound 3...............................20

Figure S24. ^{13}C-BB NMR spectrum (100 MHz, C_5D_5N) of compound 3........................20

Figure S25. ^{13}C-DEPT 135 NMR spectrum (100 MHz, C_5D_5N) of compound 3............21

Figure S26. HSQC spectrum (400 x 100 MHz, C_5D_5N) of compound 3.......................21

Figure S27. HMBC spectrum (400 x 100 MHz, C_5D_5N) of compound 3......................22

Figure S28. COSY spectrum (400 x 400 MHz, C_5D_5N) of compound 3..........................22

Figure S29. TOCSY spectrum (400 x 400 MHz, C_5D_5N) of compound 3.......................23

Figure S30. NOESY spectrum (400 x 400 MHz, C_5D_5N) of compound 3.......................23
Figure S31. ATR-Infrared spectrum of compound 4...24
Figure S32. HR-ESI-MS mass spectrum of compound 4..24
Figure S33. ^{1}H NMR spectrum (500 MHz, C_5D_5N) of compound 4...............................25
Figure S34. ^{13}C-BB NMR spectrum (125 MHz, C_5D_5N) of compound 4........................25
Figure S35. ^{13}C-DEPT 135 NMR spectrum (125 MHz, C_5D_5N) of compound 4............26
Figure S36. HSQC spectrum (500 x 125 MHz, C_5D_5N) of compound 4.......................26
Figure S37. HMBC spectrum (500 x 125 MHz, C_5D_5N) of compound 4......................27
Figure S38. COSY spectrum (500 x 500 MHz, C_5D_5N) of compound 4..........................27
Figure S39. TOCSY spectrum (500 x 500 MHz, C_5D_5N) of compound 4.......................28
Figure S40. NOESY spectrum (500 x 500 MHz, C_5D_5N) of compound 4.......................28
Figure S41. ATR-Infrared spectrum of compound 5...29
Figure S42. HR-ESI-MS mass spectrum of compound 5..29
Figure S43. ^{1}H NMR spectrum (400 MHz, C_5D_5N) of compound 5...............................30
Figure S44. ^{13}C-BB NMR spectrum (100 MHz, C_5D_5N) of compound 5........................30
Figure S45. ^{13}C-DEPT 135 NMR spectrum (100 MHz, C_5D_5N) of compound 5............31
Figure S46. HSQC spectrum (400 x 100 MHz, C_5D_5N) of compound 5.......................31
Figure S47. HMBC spectrum (400 x 100 MHz, C_5D_5N) of compound 5......................32
Figure S48. COSY spectrum (400 x 400 MHz, C_5D_5N) of compound 5..........................32
Figure S49. TOCSY spectrum (400 x 400 MHz, C_5D_5N) of compound 5.......................33
Figure S50. NOESY spectrum (400 x 400 MHz, C_5D_5N) of compound 5.......................33
Figure S51. ATR-Infrared spectrum of compound 6...34
Figure S52. HR-ESI-MS mass spectrum of compound 6..34
Figure S53. ^{1}H NMR spectrum (400 MHz, C_5D_5N) of compound 6...............................35
Figure S54. ^{13}C-BB NMR spectrum (100 MHz, C_5D_5N) of compound 6........................35
Figure S55. ^{13}C-DEPT 135 NMR spectrum (100 MHz, C_5D_5N) of compound 6............36
Figure S56. HSQC spectrum (400 x 100 MHz, C_5D_5N) of compound 6.......................36
Figure S57. HMBC spectrum (400 x 100 MHz, C_5D_5N) of compound 6......................37
Figure S58. COSY spectrum (400 x 400 MHz, C_5D_5N) of compound 6..........................37
Figure S59. TOCSY spectrum (400 x 400 MHz, C_5D_5N) of compound 6.......................38

Figure S60. ROESY spectrum (400 x 400 MHz, C_5D_5N) of compound 6........................38

Figure S61. ATR-Infrared spectrum of compound 7..39

Figure S62. HR-ESI-MS mass spectrum of compound 7..39

Figure S63. ^{1}H NMR spectrum (400 MHz, C_5D_5N) of compound 7................................40

Figure S64. ^{13}C-BB NMR spectrum (100 MHz, C_5D_5N) of compound 7........................40

Figure S65. ^{13}C-DEPT 135 NMR spectrum (100 MHz, C_5D_5N) of compound 7.............41

Figure S66. HSQC spectrum (400 x 100 MHz, C_5D_5N) of compound 7.......................41

Figure S67. HMBC spectrum (400 x 100 MHz, C_5D_5N) of compound 7......................42

Figure S68. COSY spectrum (400 x 400 MHz, C_5D_5N) of compound 7..........................42

Figure S69. TOCSY spectrum (400 x 400 MHz, C_5D_5N) of compound 7.......................43

Figure S70. NOESY spectrum (400 x 400 MHz, C_5D_5N) of compound 7.......................43

Figure S71. ATR-Infrared spectrum of compound 8..44

Figure S72. HR-ESI-MS mass spectrum of compound 8..44

Figure S73. ^{1}H NMR spectrum (400 MHz, C_5D_5N) of compound 8................................45

Figure S74. ^{13}C-BB NMR spectrum (100 MHz, C_5D_5N) of compound 8........................45

Figure S75. ^{13}C-DEPT 135 NMR spectrum (100 MHz, C_5D_5N) of compound 8.............46

Figure S76. HSQC spectrum (400 x 100 MHz, C_5D_5N) of compound 8.......................46

Figure S77. HMBC spectrum (400 x 100 MHz, C_5D_5N) of compound 8......................47

Figure S78. COSY spectrum (400 x 400 MHz, C_5D_5N) of compound 8..........................47

Figure S79. TOCSY spectrum (400 x 400 MHz, C_5D_5N) of compound 8.......................48

Figure S80. NOESY spectrum (400 x 400 MHz, C_5D_5N) of compound 8.......................48

Figure S81. ATR-Infrared spectrum of compound 9..49

Figure S82. HR-ESI-MS mass spectrum of compound 9..49

Figure S83. ^{1}H NMR spectrum (400 MHz, C_5D_5N) of compound 9................................50

Figure S84. ^{13}C-BB NMR spectrum (100 MHz, C_5D_5N) of compound 9........................50

Figure S85. ^{13}C-DEPT 135 NMR spectrum (100 MHz, C_5D_5N) of compound 9.............51

Figure S86. HSQC spectrum (400 x 100 MHz, C_5D_5N) of compound 9.......................51

Figure S87. HMBC spectrum (400 x 100 MHz, C_5D_5N) of compound 9......................52

Figure S88. COSY spectrum (400 x 400 MHz, C_5D_5N) of compound 9..........................52

Figure S89. TOCSY spectrum (400 x 400 MHz, C_5D_5N) of compound 9.......................53

Figure S90. NOESY spectrum (400 x 400 MHz, C_5D_5N) of compound 9........................53

Figure S91. ATR-Infrared spectrum of compound 10..54

Figure S92. HR-ESI-MS mass spectrum of compound 10..54

Figure S93. ^{1}H NMR spectrum (400 MHz, C_5D_5N) of compound 10..............................55

Figure S94. ^{13}C-BB NMR spectrum (100 MHz, C_5D_5N) of compound 10......................55

Figure S95. ^{13}C-DEPT 135 NMR spectrum (100 MHz, C_5D_5N) of compound 10...........56

Figure S96. HSQC spectrum (400 x 100 MHz, C_5D_5N) of compound 10.....................56

Figure S97. HMBC spectrum (400 x 100 MHz, C_5D_5N) of compound 10....................57

Figure S98. COSY spectrum (400 x 400 MHz, C_5D_5N) of compound 10........................57

Figure S99. TOCSY spectrum (400 x 400 MHz, C_5D_5N) of compound 10.....................58

Figure S100. ROESY spectrum (400 x 400 MHz, C_5D_5N) of compound 10...................58

Figure S101. ATR-Infrared spectrum of compound 11..59

Figure S102. HR-ESI-MS mass spectrum of compound 11..59

Figure S103. ^{1}H NMR spectrum (400 MHz, C_5D_5N) of compound 11............................60

Figure S104. ^{13}C-BB NMR spectrum (100 MHz, C_5D_5N) of compound 11....................60

Figure S105. ^{13}C-DEPT 135 NMR spectrum (100 MHz, C_5D_5N) of compound 11.........61

Figure S106. HSQC spectrum (400 x 100 MHz, C_5D_5N) of compound 11...................61

Figure S107. HMBC spectrum (400 x 100 MHz, C_5D_5N) of compound 11..................62

Figure S108. COSY spectrum (400 x 400 MHz, C_5D_5N) of compound 11......................62

Figure S109. TOCSY spectrum (400 x 400 MHz, C_5D_5N) of compound 11...................63

Figure S110. ROESY spectrum (400 x 400 MHz, C_5D_5N) of compound 11...................63

Figure S111. ATR-Infrared spectrum of compound 12..64

Figure S112. HR-ESI-MS mass spectrum of compound 12..64

Figure S113. ^{1}H NMR spectrum (400 MHz, C_5D_5N) of compound 12............................65

Figure S114. ^{13}C-BB NMR spectrum (100 MHz, C_5D_5N) of compound 12....................65

Figure S115. ^{13}C-DEPT 135 NMR spectrum (100 MHz, C_5D_5N) of compound 12.........66

Figure S116. HSQC spectrum (400 x 100 MHz, C_5D_5N) of compound 12...................66

Figure S117. HMBC spectrum (400 x 100 MHz, C_5D_5N) of compound 12..................67

Figure S118. COSY spectrum (400 x 400 MHz, C_5D_5N) of compound 12......................67

Figure S119. TOCSY spectrum (400 x 400 MHz, C_5D_5N) of compound 12...................68

Figure S120. NOESY spectrum (400 x 400 MHz, C_5D_5N) of compound 12....................68
Figure S121. ATR-Infrared spectrum of compound 13..69
Figure S122. HR-ESI-MS mass spectrum of compound 13..69
Figure S123. ^{1}H NMR spectrum (5400 MHz, C_5D_5N) of compound 13..........................70
Figure S124. ^{13}C-BB NMR spectrum (100 MHz, C_5D_5N) of compound 13....................70
Figure S125. ^{13}C-DEPT 135 NMR spectrum (100 MHz, C_5D_5N) of compound 13.........71
Figure S126. HSQC spectrum (400 x 100 MHz, C_5D_5N) of compound 13...................71
Figure S127. HMBC spectrum (400 x 100 MHz, C_5D_5N) of compound 13..................72
Figure S128. COSY spectrum (400 x 400 MHz, C_5D_5N) of compound 13......................72
Figure S129. TOCSY spectrum (400 x 400 MHz, C_5D_5N) of compound 13...................73
Figure S130. ROESY spectrum (400 x 400 MHz, C_5D_5N) of compound 13...................73
Figure S131. ATR-Infrared spectrum of compound 14..74
Figure S132. HR-ESI-MS mass spectrum of compound 14..74
Figure S133. ^{1}H NMR spectrum (400 MHz, C_5D_5N) of compound 14............................75
Figure S134. ^{13}C-BB NMR spectrum (100 MHz, C_5D_5N) of compound 14....................75
Figure S135. ^{13}C-DEPT 135 NMR spectrum (100 MHz, C_5D_5N) of compound 14.........76
Figure S136. HSQC spectrum (400 x 100 MHz, C_5D_5N) of compound 14...................76
Figure S137. HMBC spectrum (400 x 100 MHz, C_5D_5N) of compound 14..................77
Figure S138. COSY spectrum (400 x 400 MHz, C_5D_5N) of compound 14......................77
Figure S139. TOCSY spectrum (400 x 400 MHz, C_5D_5N) of compound 14...................78
Figure S140. ROESY spectrum (400 x 400 MHz, C_5D_5N) of compound 14...................78
Figure S141. ATR-Infrared spectrum of compound 15..79
Figure S142. HR-ESI-MS mass spectrum of compound 15..79
Figure S143. ^{1}H NMR spectrum (500 MHz, C_5D_5N) of compound 15............................80
Figure S144. ^{13}C-BB NMR spectrum (125 MHz, C_5D_5N) of compound 15....................80
Figure S145. ^{13}C-DEPT 135 NMR spectrum (125 MHz, C_5D_5N) of compound 15.........81
Figure S146. HSQC spectrum (500 x 125 MHz, C_5D_5N) of compound 15...................81
Figure S147. HMBC spectrum (500 x 125 MHz, C_5D_5N) of compound 15..................82
Figure S148. COSY spectrum (500 x 500 MHz, C_5D_5N) of compound 15......................82
Figure S149. TOCSY spectrum (500 x 500 MHz, C_5D_5N) of compound 15...................83

Figure S150. NOESY spectrum (500 x 500 MHz, C_5D_5N) of compound 15...................83
Figure S151. ATR-Infrared spectrum of compound 16..84
Figure S152. HR-ESI-MS mass spectrum of compound 16..84
Figure S153. ^{1}H NMR spectrum (500 MHz, C_5D_5N) of compound 16............................85
Figure S154. ^{13}C-BB NMR spectrum (125 MHz, C_5D_5N) of compound 16....................85
Figure S155. ^{13}C-DEPT 135 NMR spectrum (125 MHz, C_5D_5N) of compound 16.........86
Figure S156. HSQC spectrum (500 x 125 MHz, C_5D_5N) of compound 16...................86
Figure S157. HMBC spectrum (500 x 125 MHz, C_5D_5N) of compound 16..................87
Figure S158. COSY spectrum (500 x 500 MHz, C_5D_5N) of compound 16......................87
Figure S159. TOCSY spectrum (500 x 500 MHz, C_5D_5N) of compound 16...................88
Figure S160. NOESY spectrum (500 x 500 MHz, C_5D_5N) of compound 16...................88
Figure S161. ATR-Infrared spectrum of compound 17..89
Figure S162. HR-ESI-MS mass spectrum of compound 17..89
Figure S163. ^{1}H NMR spectrum (500 MHz, C_5D_5N) of compound 17............................90
Figure S164. ^{13}C-BB NMR spectrum (125 MHz, C_5D_5N) of compound 17....................90
Figure S165. ^{13}C-DEPT 135 NMR spectrum (125 MHz, C_5D_5N) of compound 17.........91
Figure S166. HSQC spectrum (500 x 125 MHz, C_5D_5N) of compound 17...................91
Figure S167. HMBC spectrum (500 x 125 MHz, C_5D_5N) of compound 17..................92
Figure S168. TOCSY spectrum (500 x 500 MHz, C_5D_5N) of compound 17...................92
Figure S169. ROESY spectrum (500 x 500 MHz, C_5D_5N) of compound 17..................93
Figure S170. ATR-Infrared spectrum of compound 18.. 93
Figure S171. HR-ESI-MS mass spectrum of compound 18..94
Figure S172. ^{1}H NMR spectrum (400 MHz, C_5D_5N) of compound 18............................94
Figure S173. ^{13}C-BB NMR spectrum (100 MHz, C_5D_5N) of compound 18....................95
Figure S174. ^{13}C-DEPT 135 NMR spectrum (100 MHz, C_5D_5N) of compound 18.........95
Figure S175. HSQC spectrum (400 x 100 MHz, C_5D_5N) of compound 18...................96
Figure S176. HMBC spectrum (400 x 100 MHz, C_5D_5N) of compound 18..................96
Figure S177. COSY spectrum (400 x 400 MHz, C_5D_5N) of compound 18......................97
Figure S178. TOCSY spectrum (400 x 400 MHz, C_5D_5N) of compound 18.................97
Figure S179. ROESY spectrum (400 x 400 MHz, C_5D_5N) of compound 18...................98

Figure S180. Effect of dimethyl sulfoxide (DMSO) concentration on nitric oxide (NO) production in LPS-stimulated J774.A1 macrophages. This control experiment demonstrates that the DMSO concentration used as the vehicle in the biological assays did not significantly affect NO production……………………………………………...98

Figure S1. ATR-Infrared spectrum of compound 1.

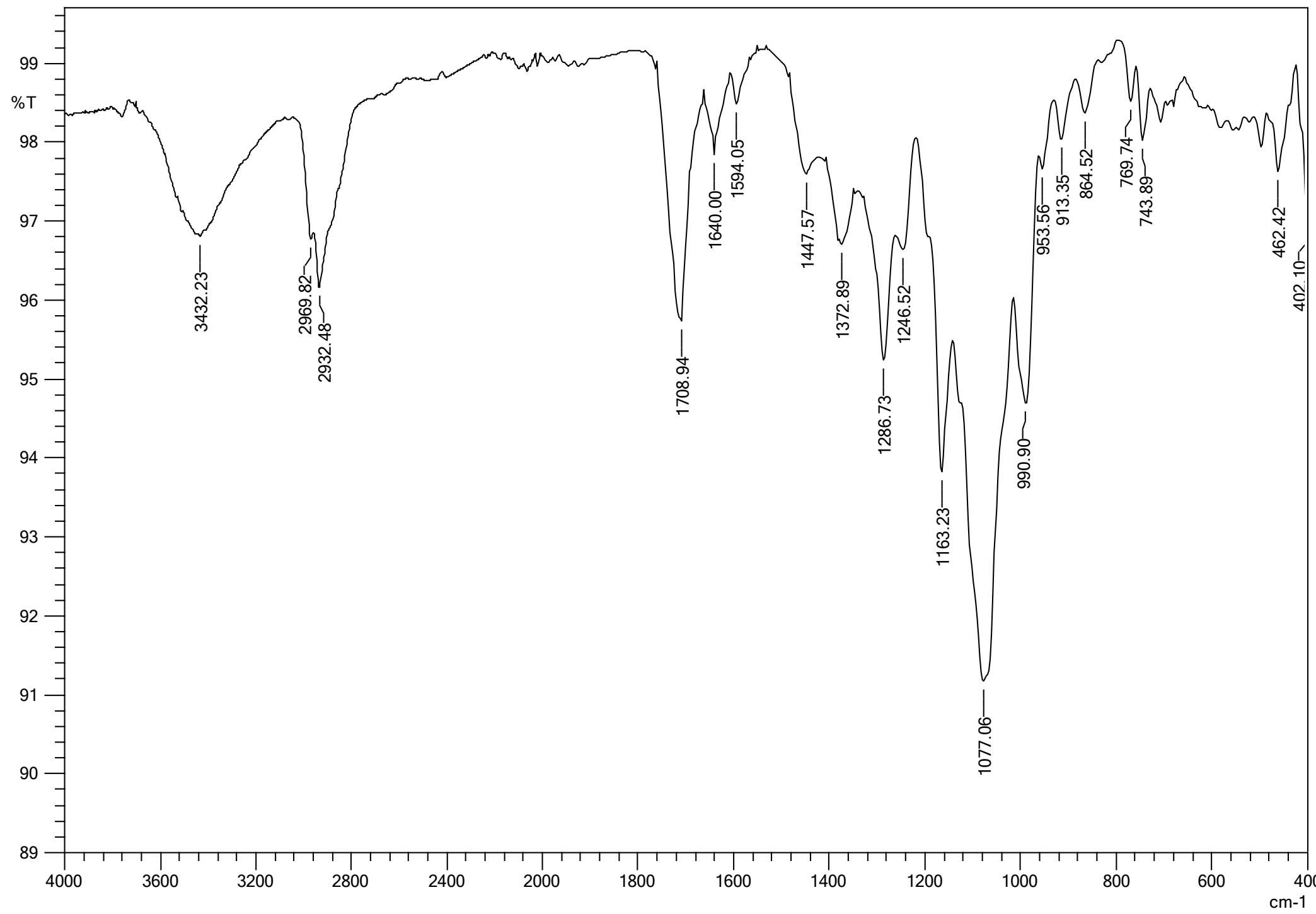

Figure S2. HR-ESI-MS mass spectrum of compound 1.

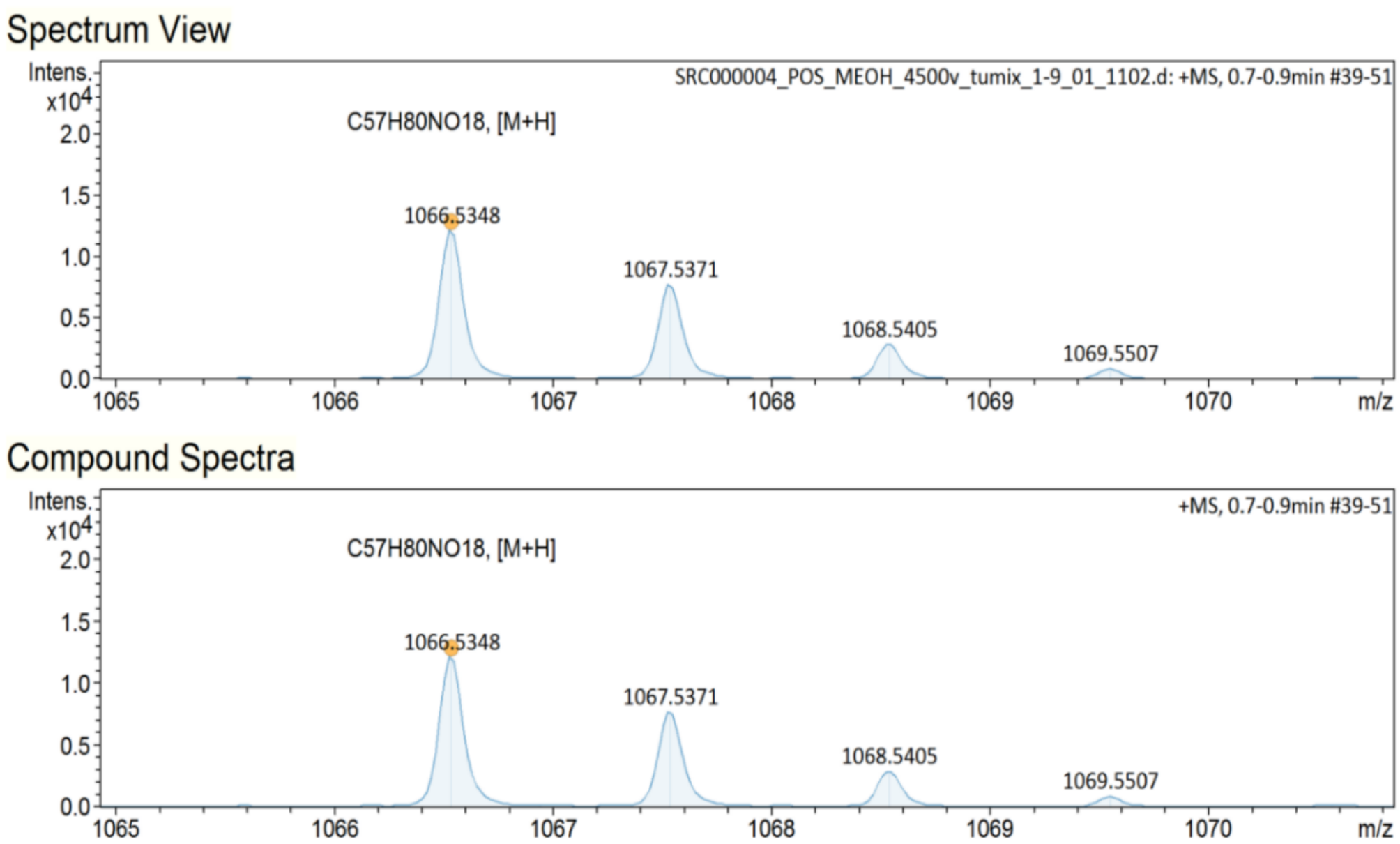

Meas. m/z	#	Ion Formula	m/z	err [ppm]	mSigma	# mSigma	Score	rdb	e⁻ Conf	N-Rule
1066.5348	1	C57H80NO18	1066.5370	2.1	7.2	1	100.00	18.5	even	ok

Figure S3. ^{1}H NMR spectrum (500 MHz, C_5D_5N) of compound 1.

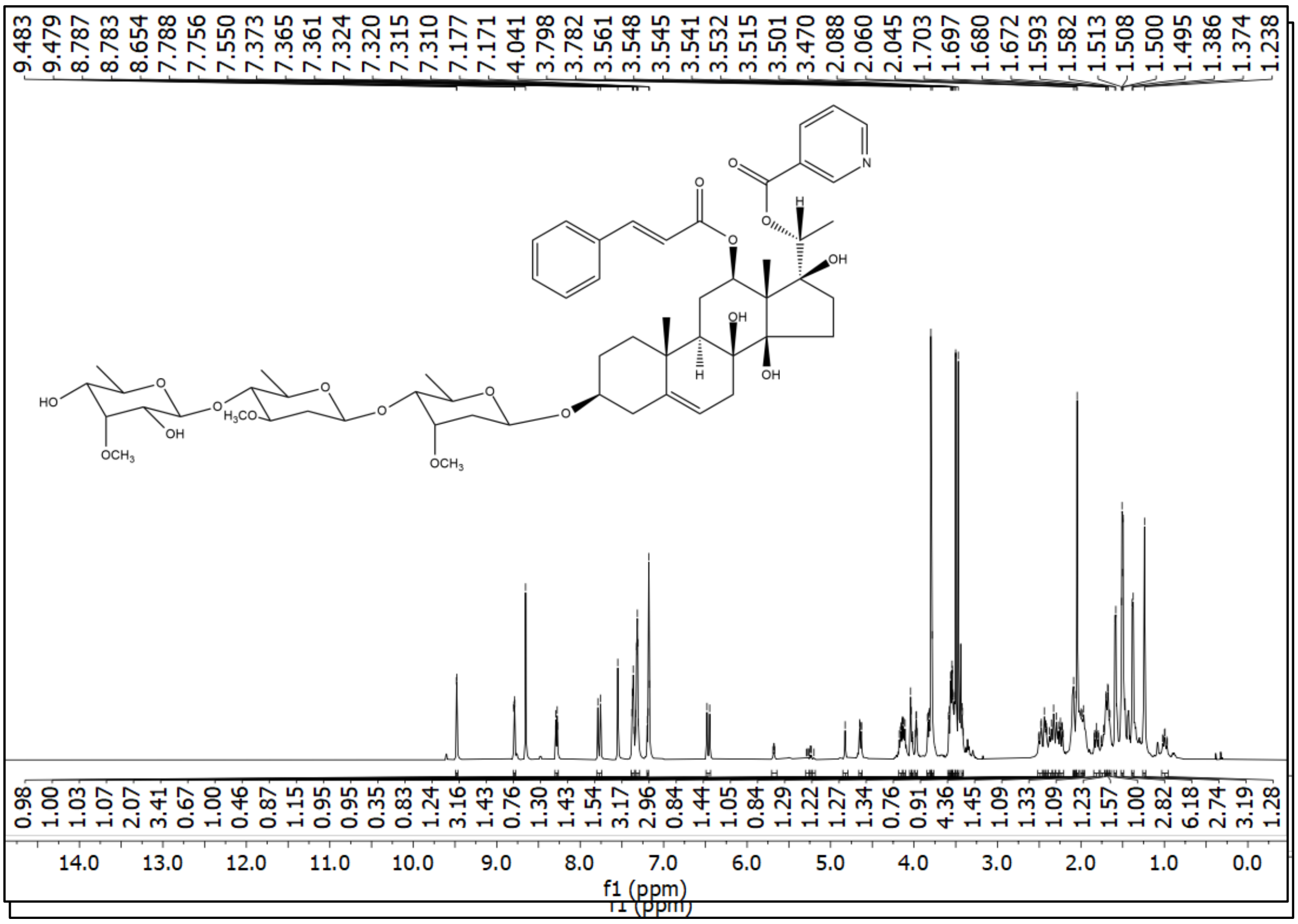

Figure S4. ^{13}C-BB NMR spectrum (125 MHz, C_5D_5N) of compound 1

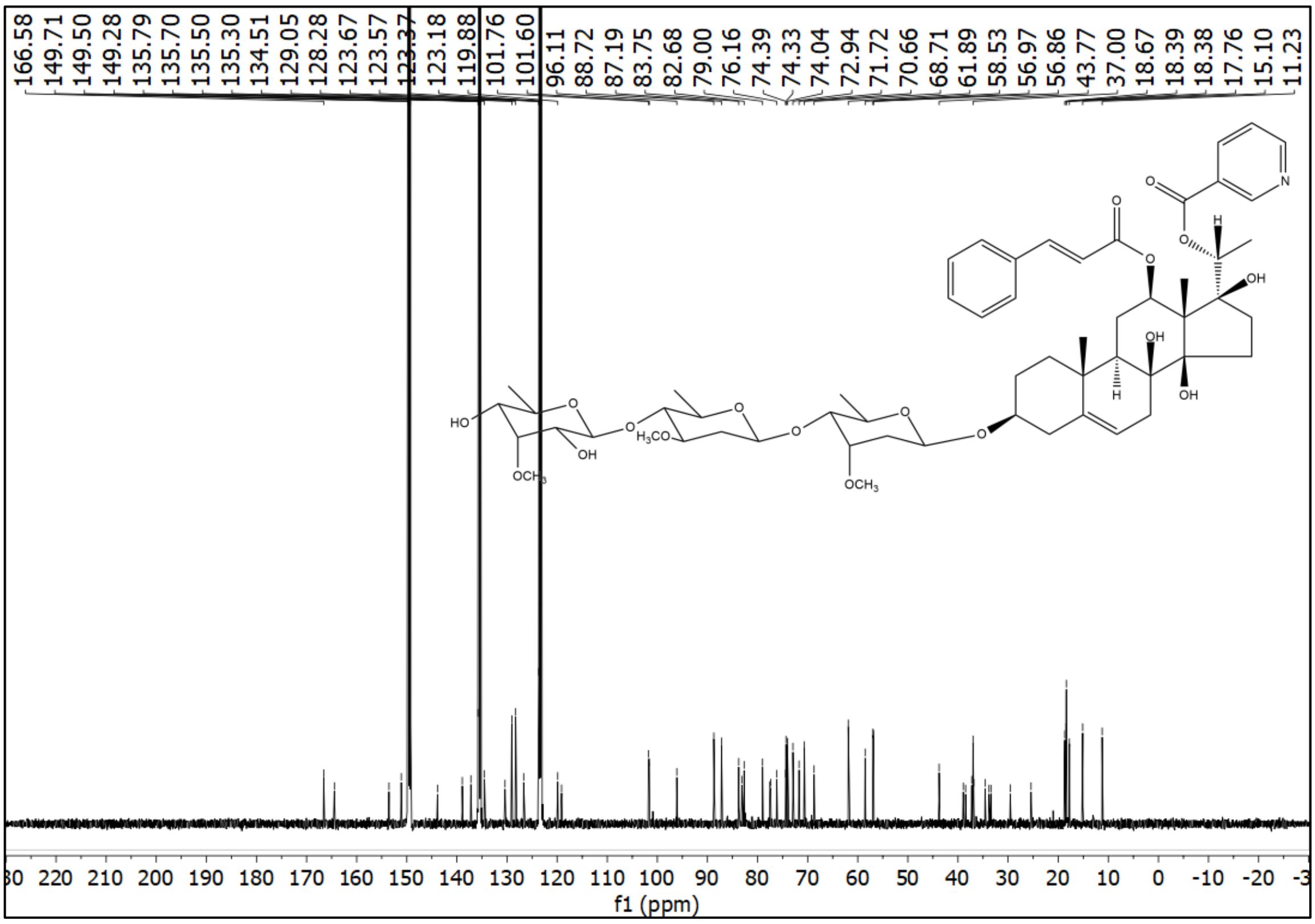

Figure S5. ^{13}C-DEPT135 NMR spectrum (125 MHz, C_5D_5N) of compound 1

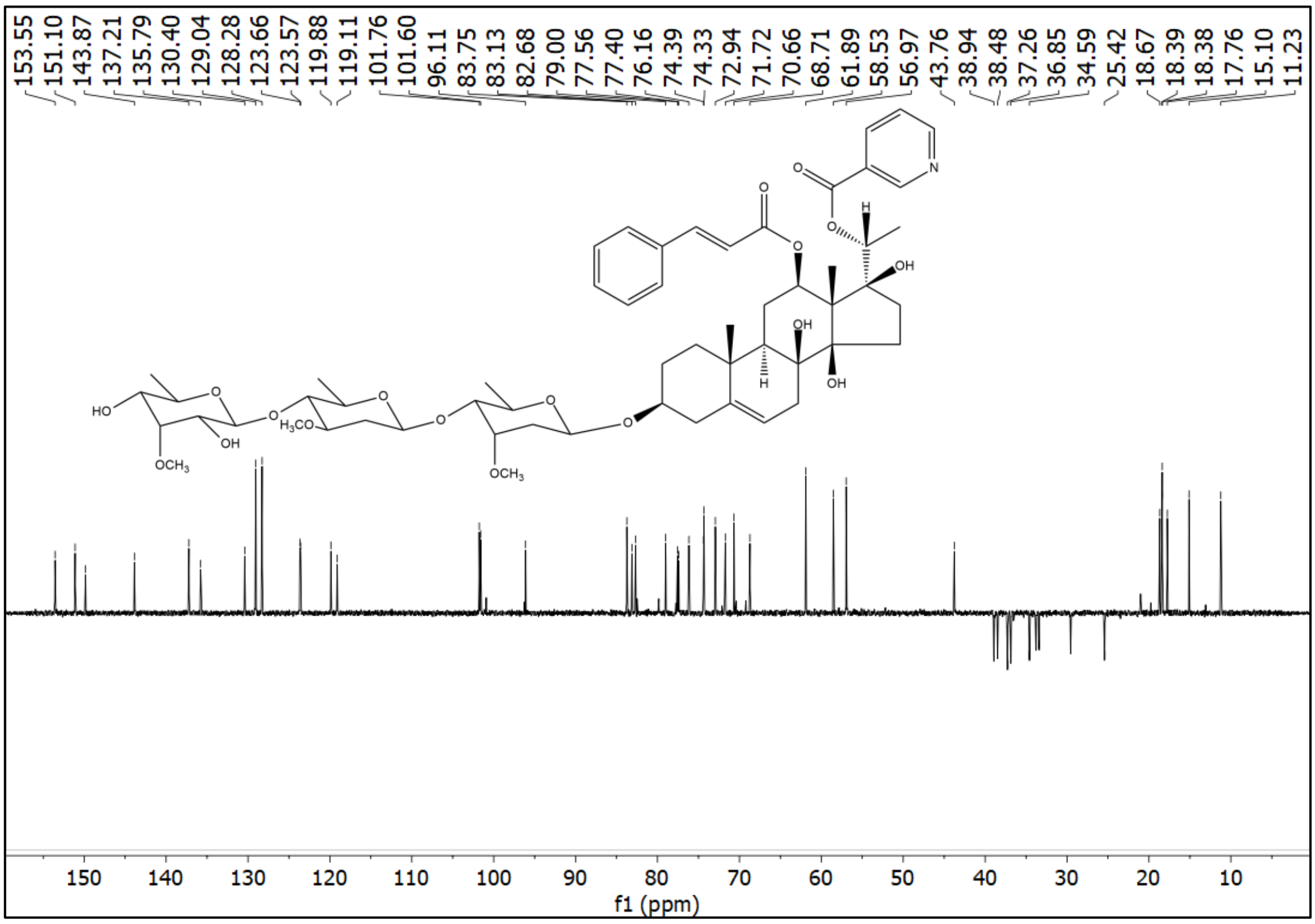

Figure S6. HSQC spectrum (500 x 125 MHz, C_5D_5N) of compound 1

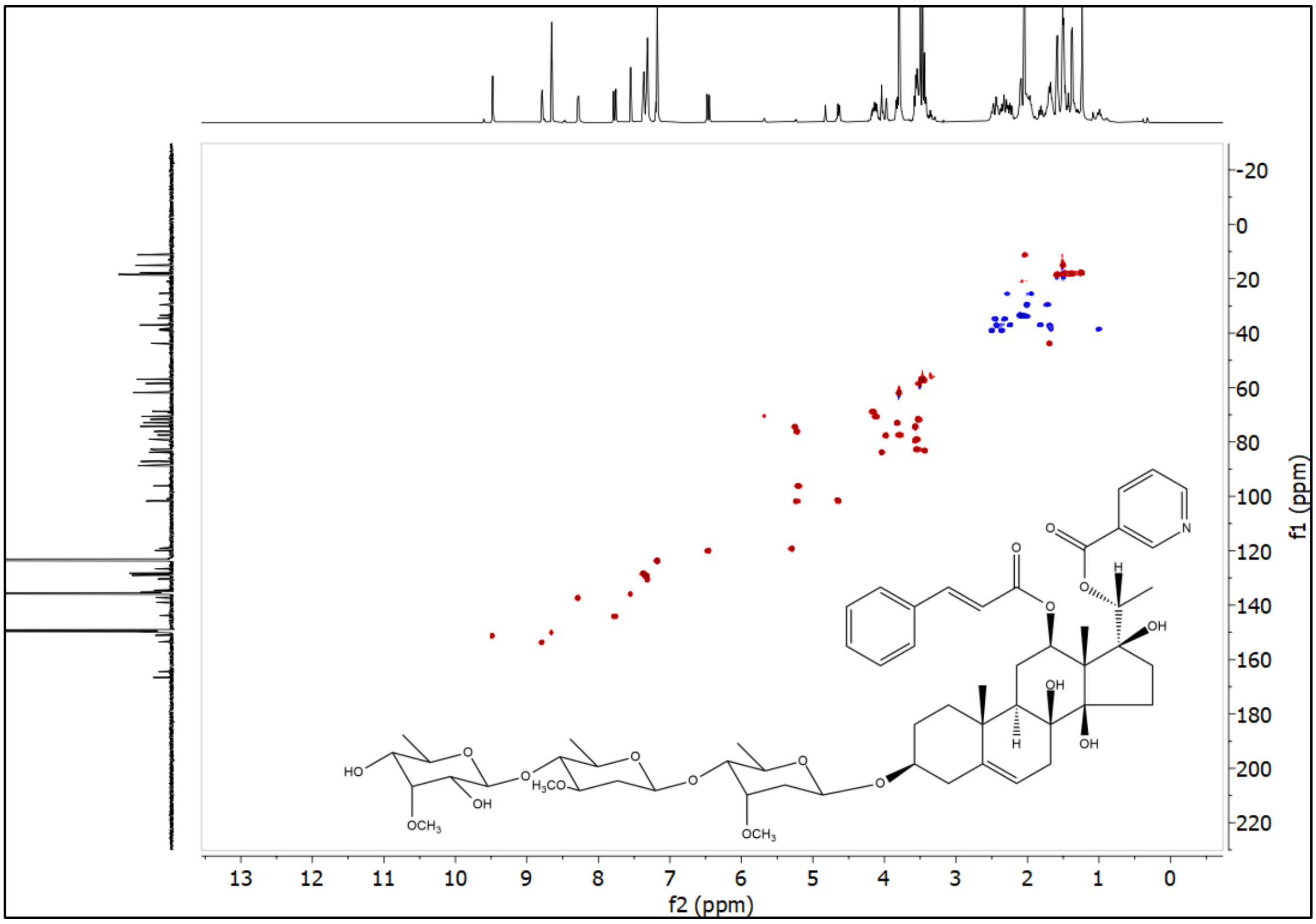

Figure S7. HMBC spectrum (500 x 125 MHz, C_5D_5N) of compound 1

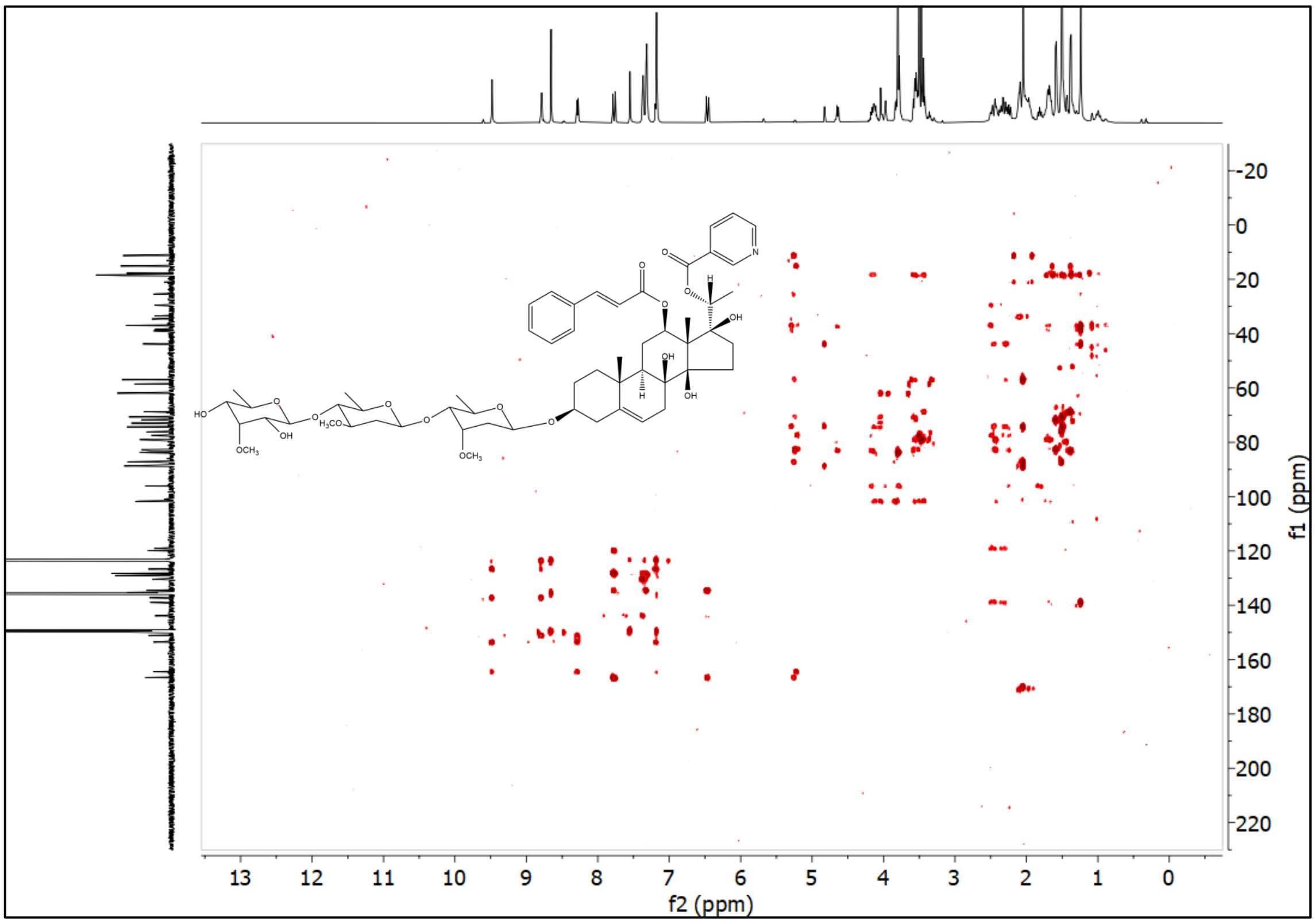

Figure S8. COSY spectrum (500 x 500 MHz, C_5D_5N) of compound 1.

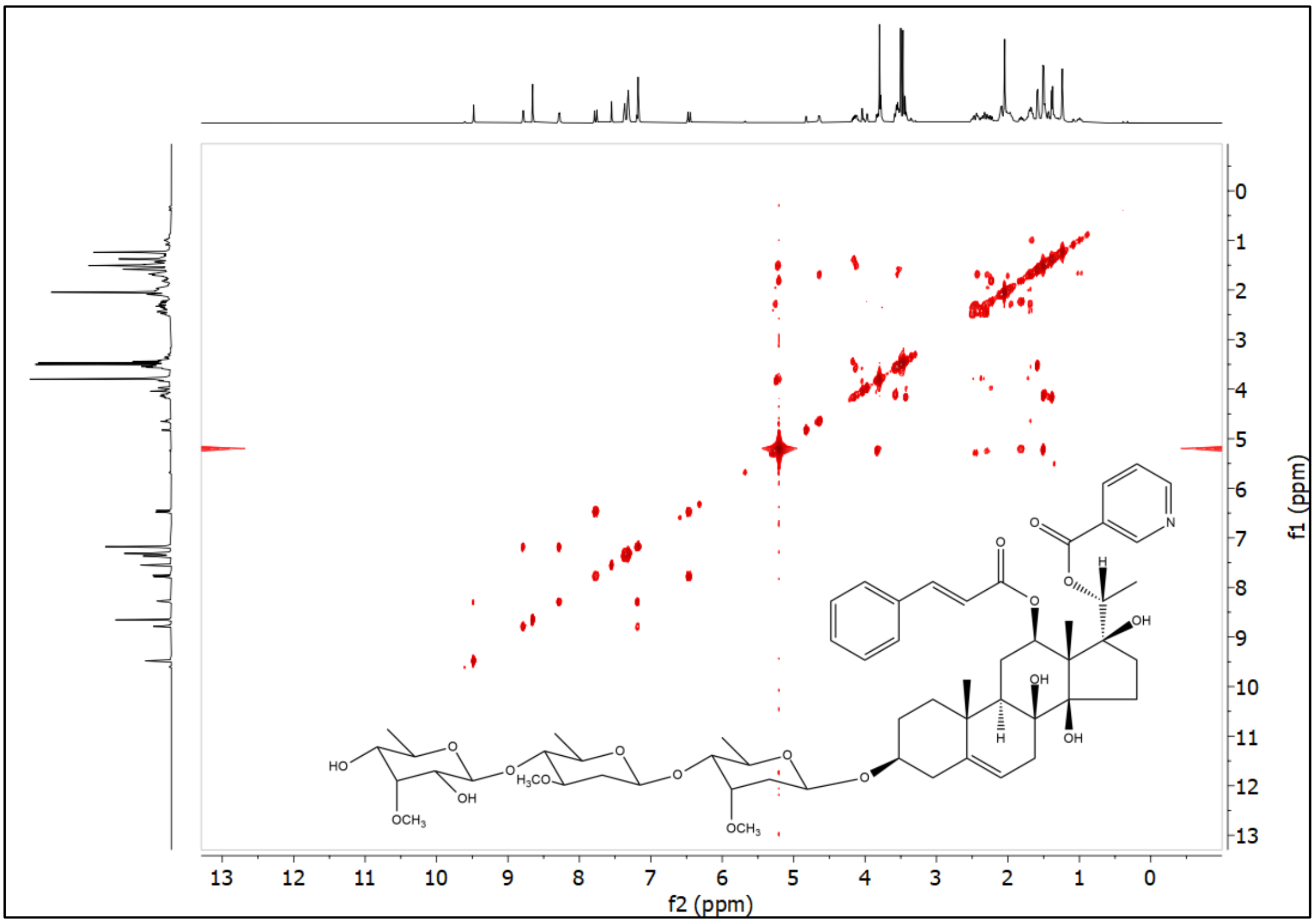

Figure S9. TOCSY spectrum (500 x 500 MHz, C_5D_5N) of compound 1.

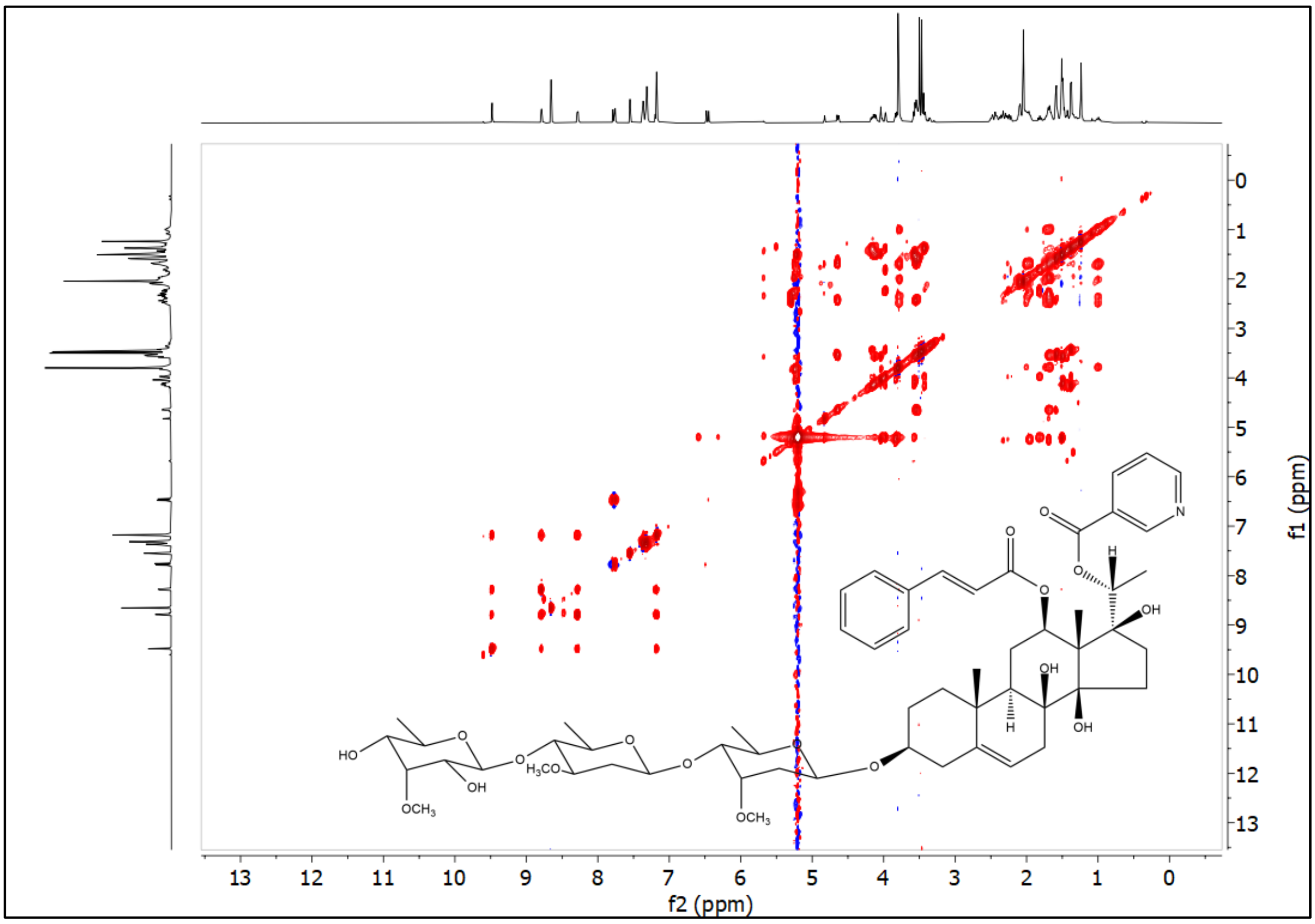

Figure S10. NOESY spectrum (500 x 500 MHz, C_5D_5N) of compound 1.

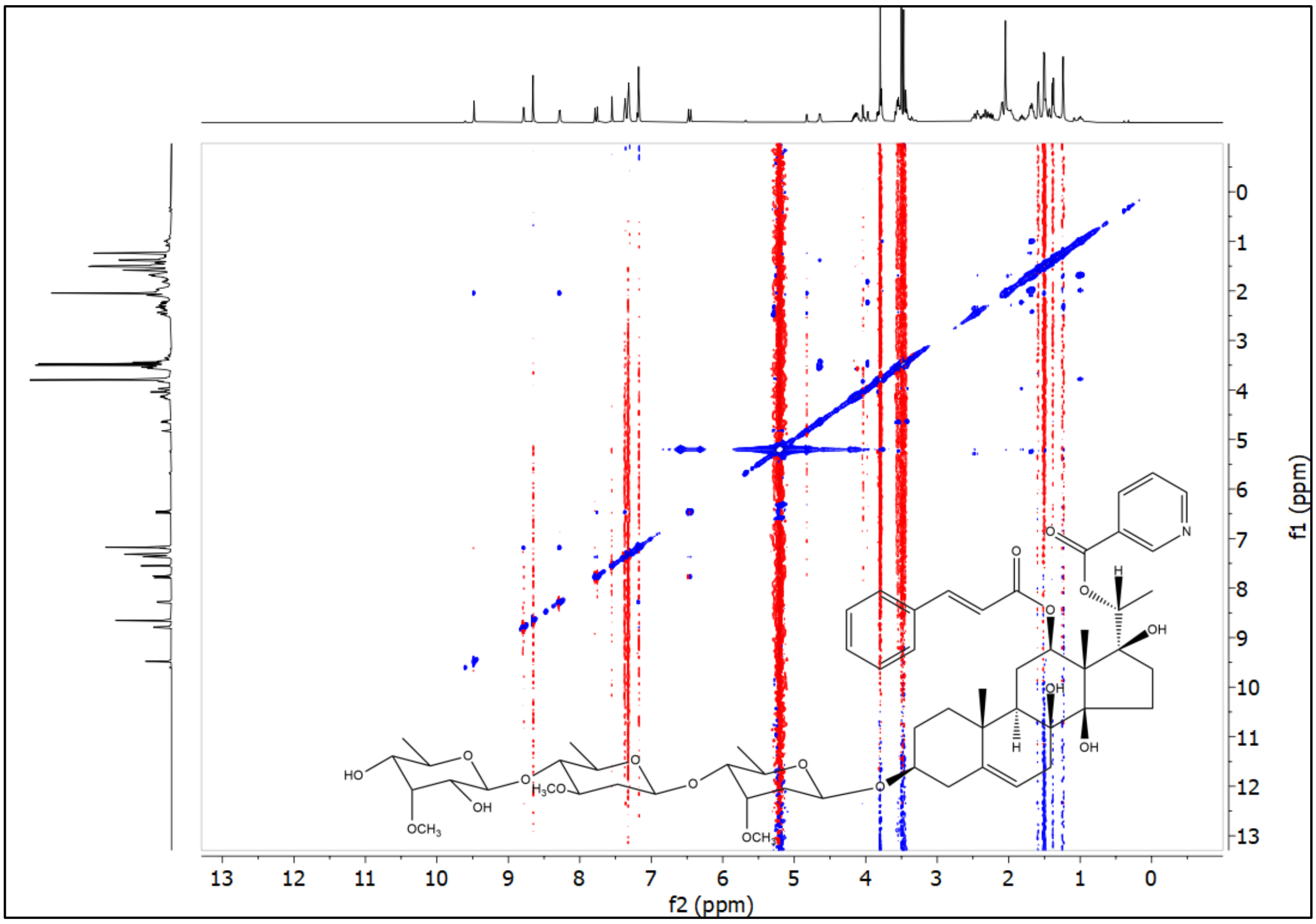

Figure S11. ATR-Infrared spectrum of compound 2.

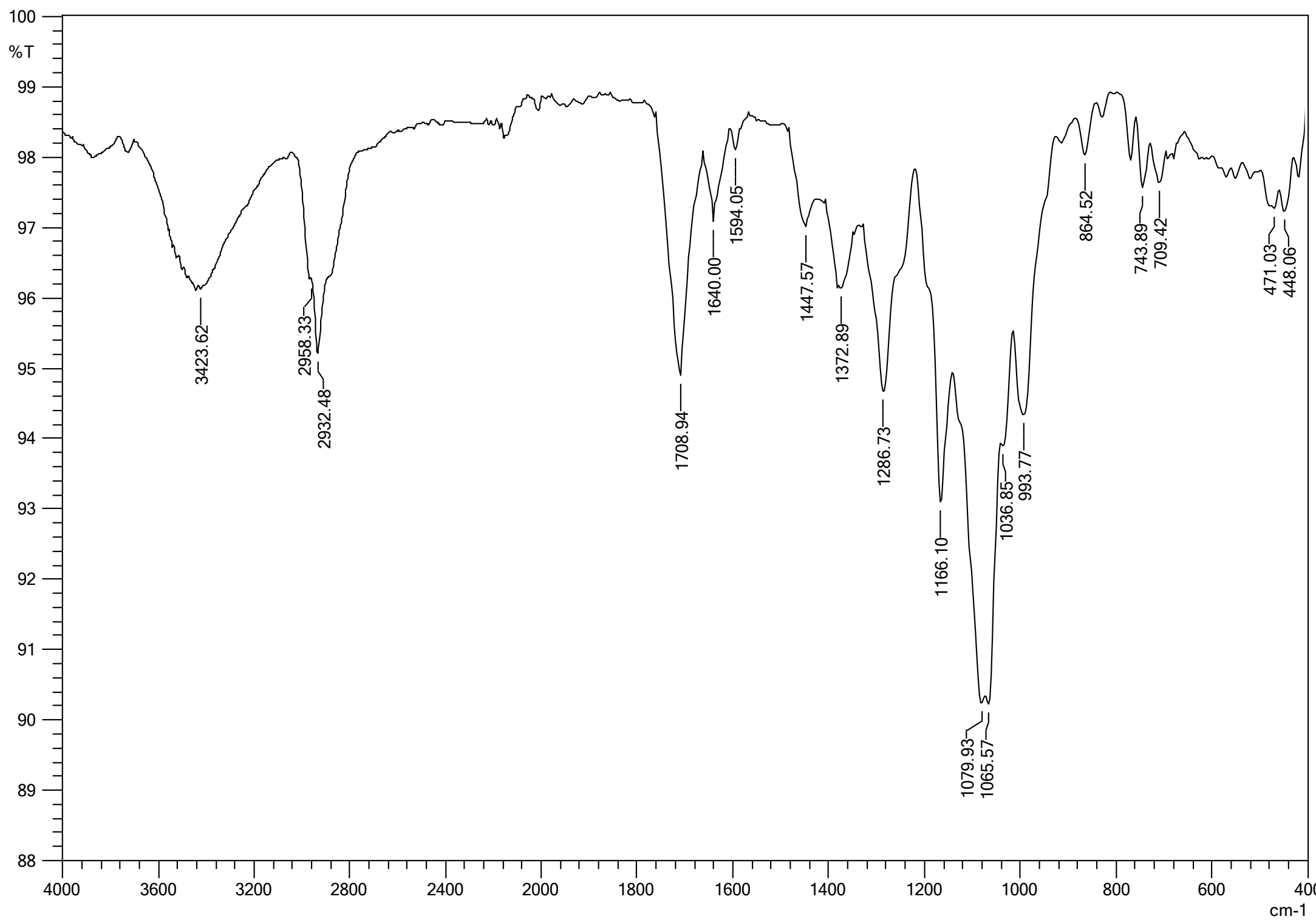

Figure S12. HR-ESI-MS mass spectrum of compound 2.

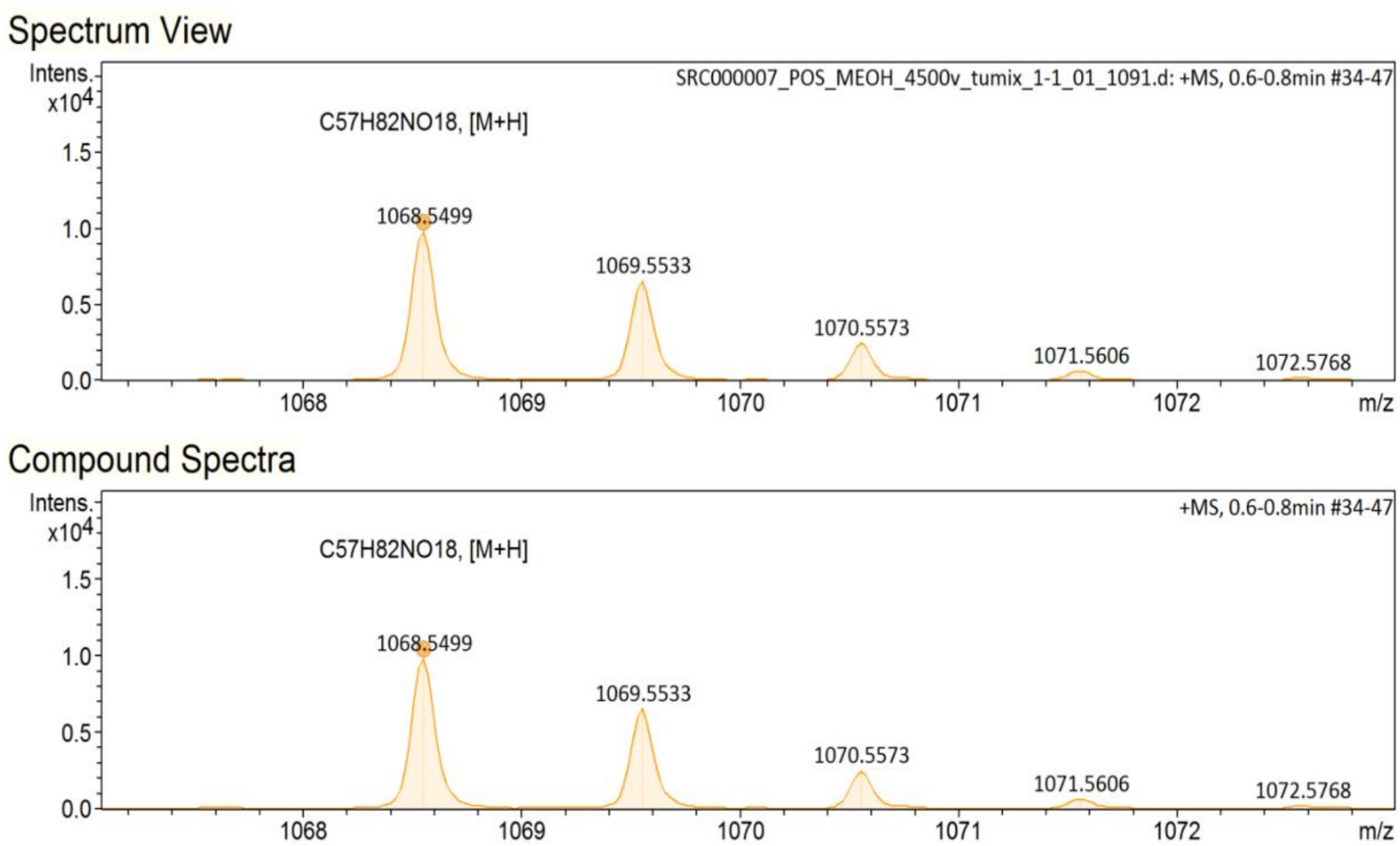

Meas. m/z	#	Ion Formula	m/z	err [ppm]	mSigma	# mSigma	Score	rdb	e⁻ Conf	N-Rule
1068.5499	1	C57H82NO18	1068.5526	2.6	15.9	1	100.00	17.5	even	ok

Figure S13. ^{1}H NMR spectrum (500 MHz, C_5D_5N) of compound 2

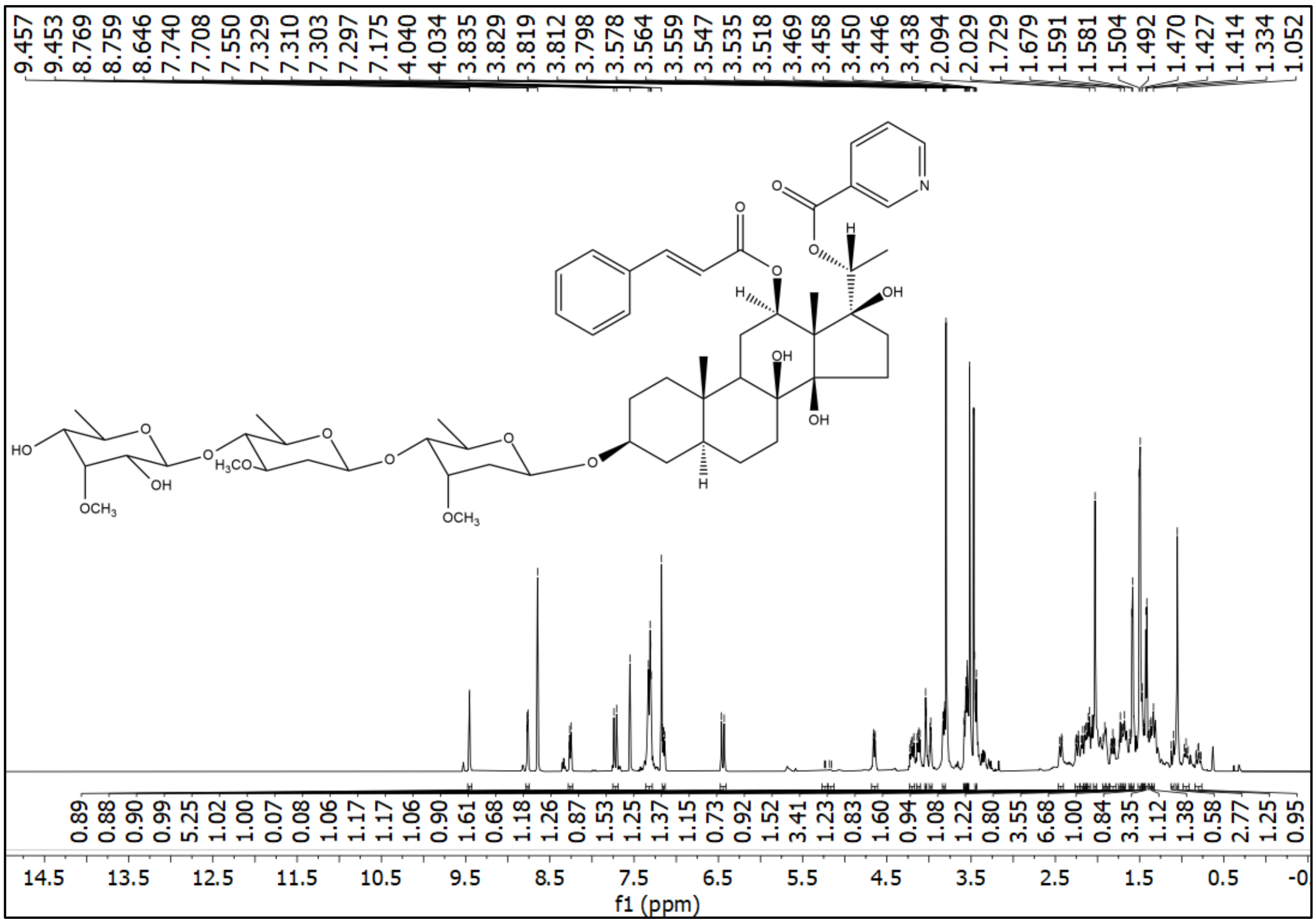

Figure S14. ^{13}C-BB NMR spectrum (125 MHz, C_5D_5N) of compound 2

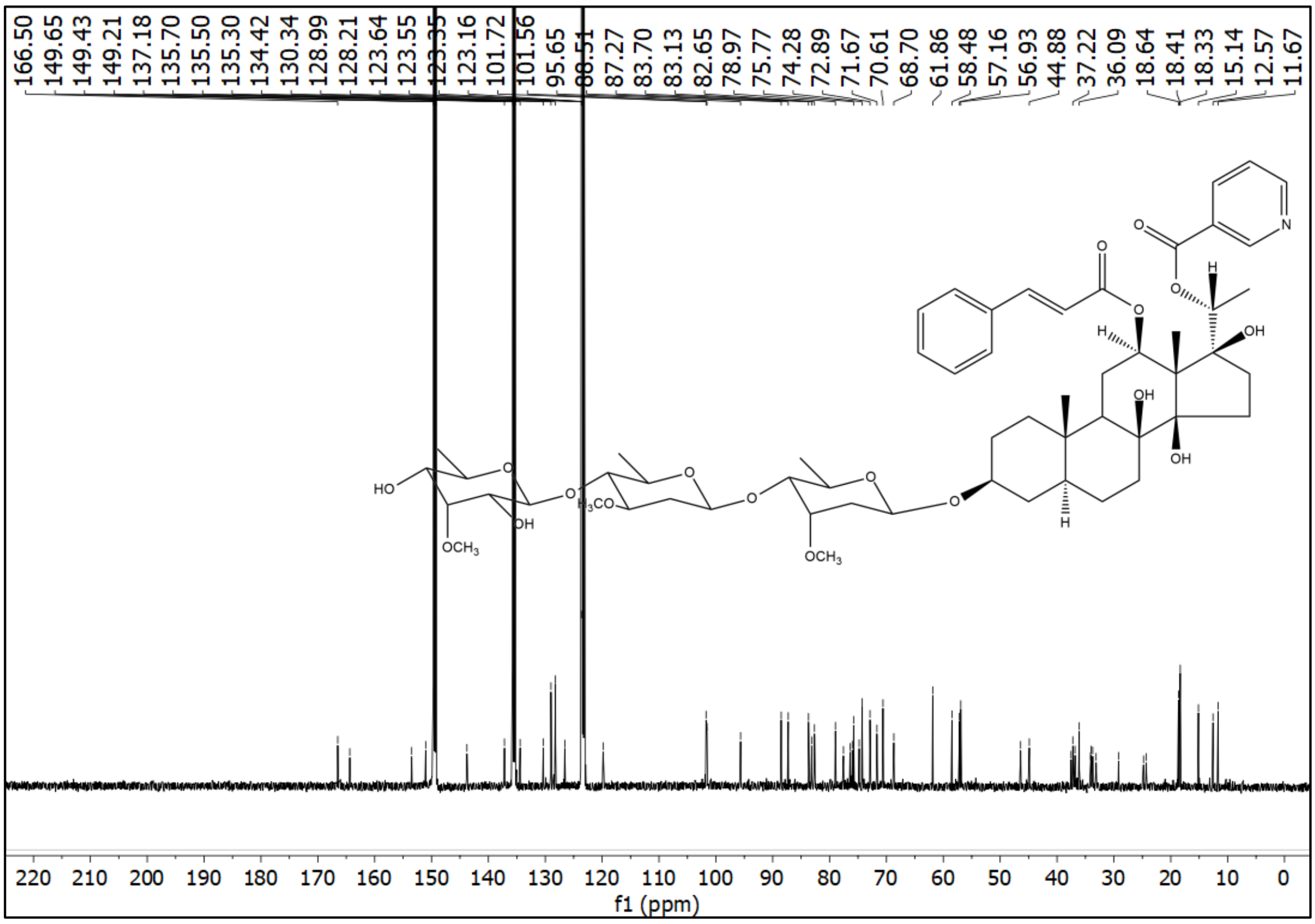

Figure S15. ^{13}C-DEPT135 NMR spectrum (125 MHz, C_5D_5N) of compound 2

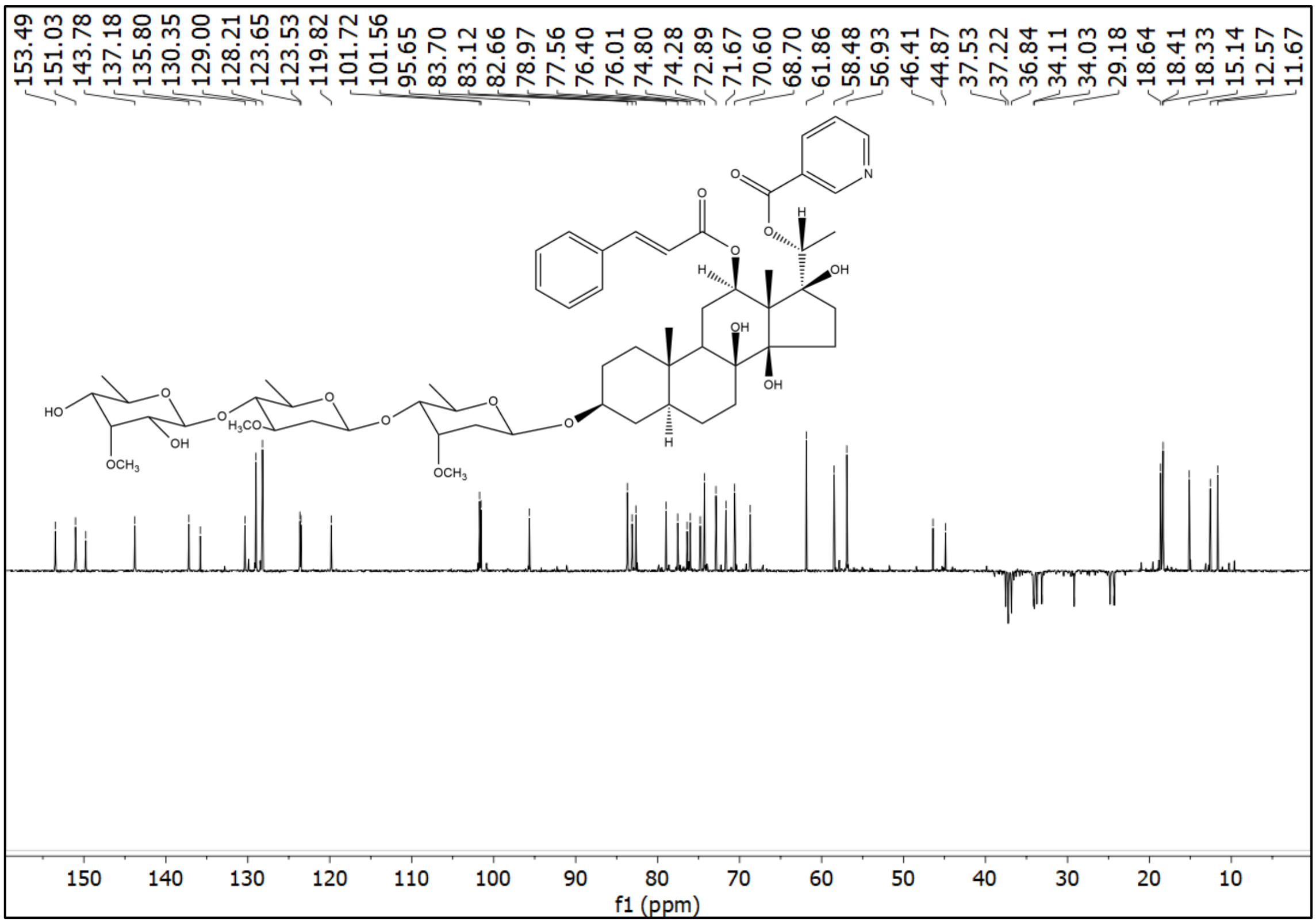

Figure S16. HSQC spectrum (500 x 125 MHz, C_5D_5N) of compound 2

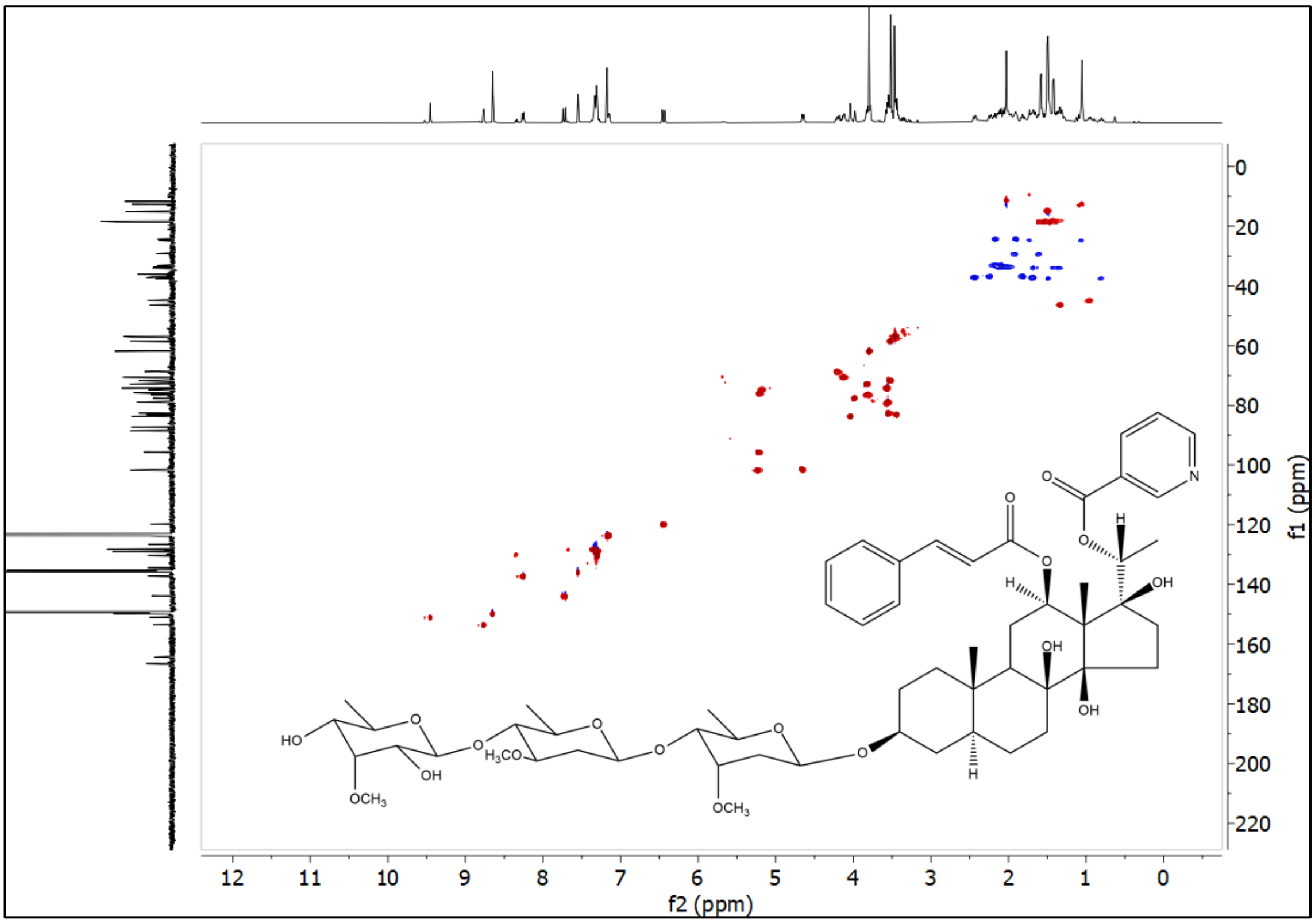

Figure S17. HMBC spectrum (500 x 125 MHz, C_5D_5N) of compound 2

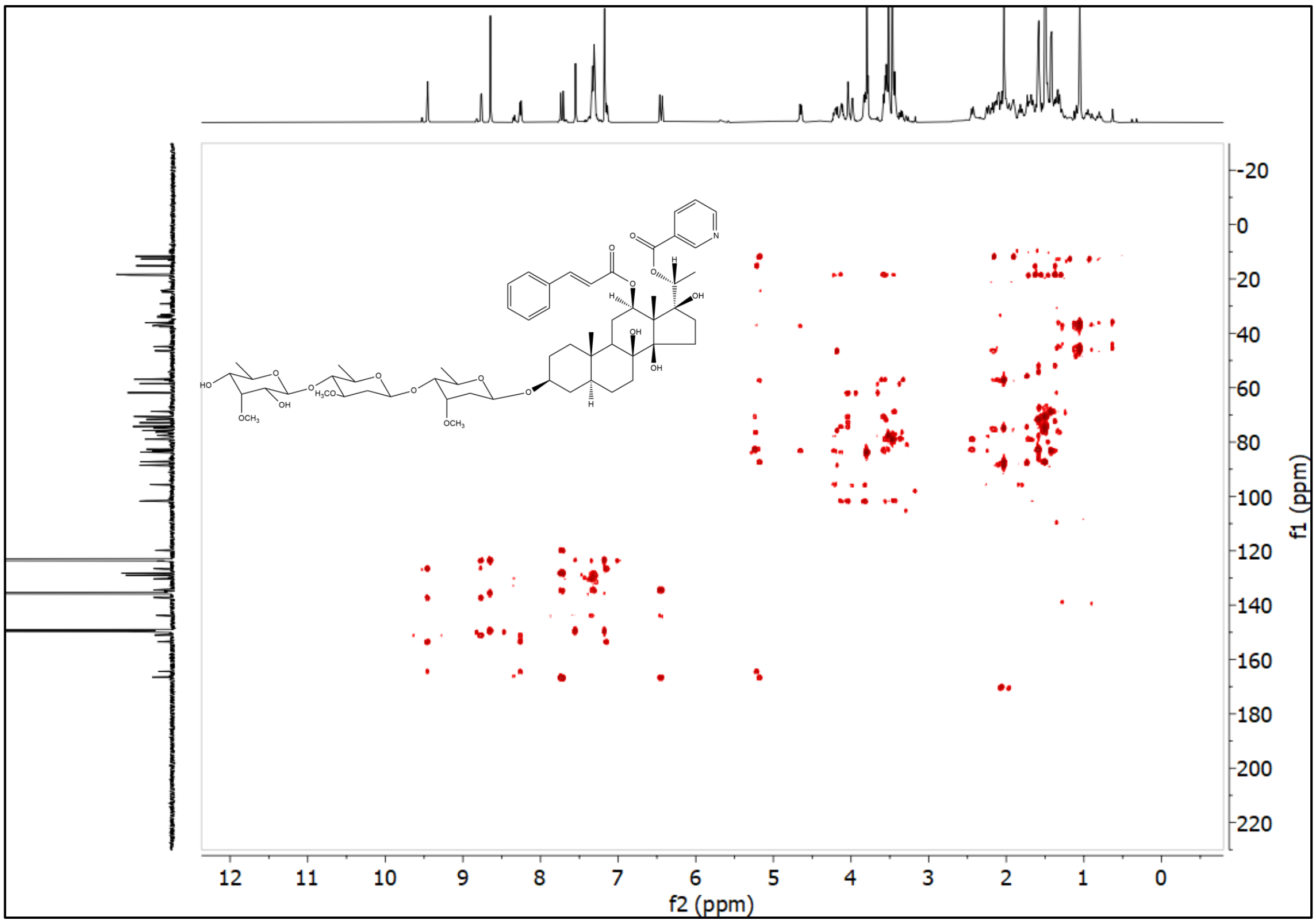

Figure S18. COSY spectrum (500 x 500 MHz, C_5D_5N) of compound 2

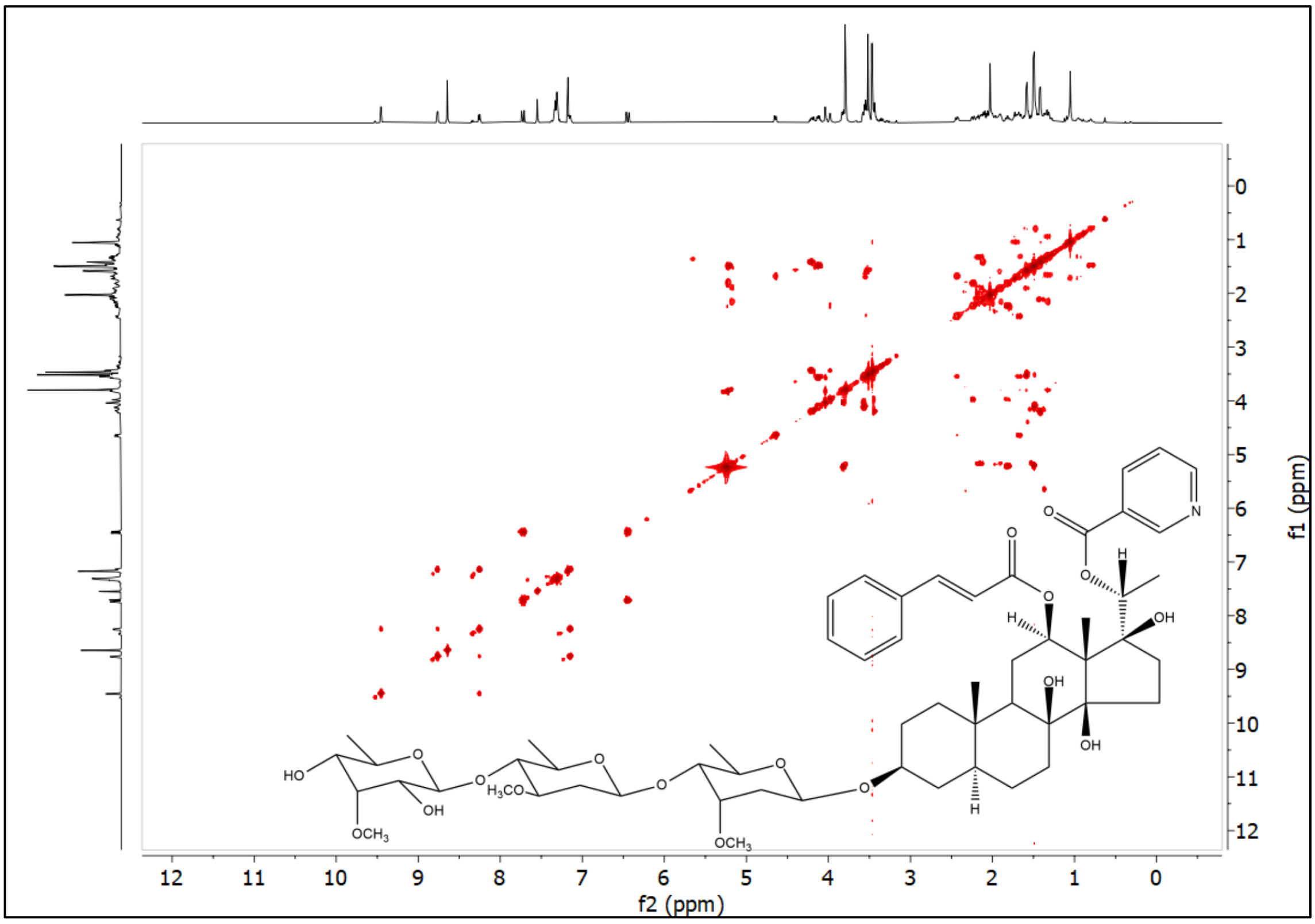

Figure S19. TOCSY spectrum (500 x 500 MHz, C_5D_5N) of compound 2

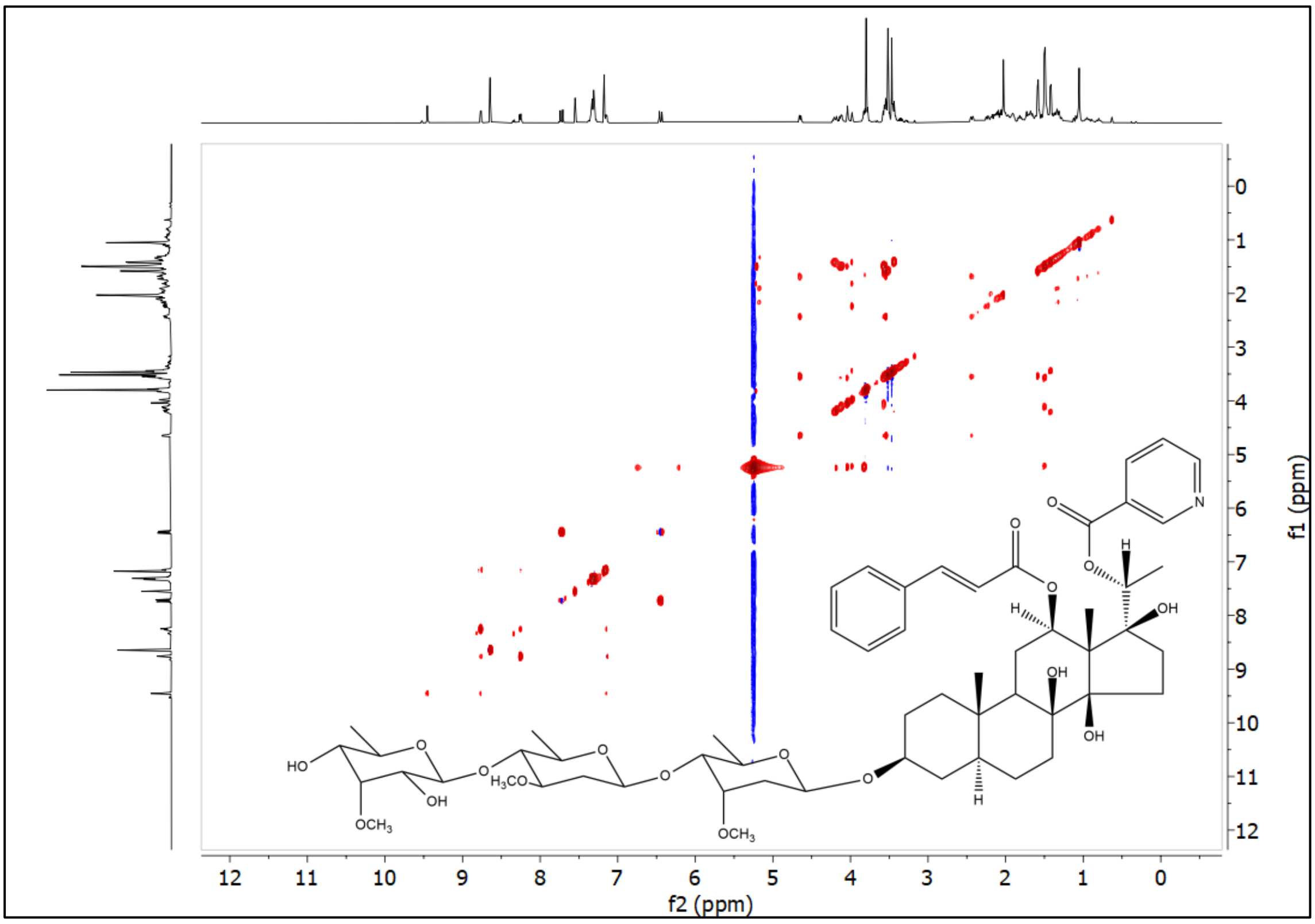

Figure S20. NOESY spectrum (500 x 500 MHz, C_5D_5N) of compound 2

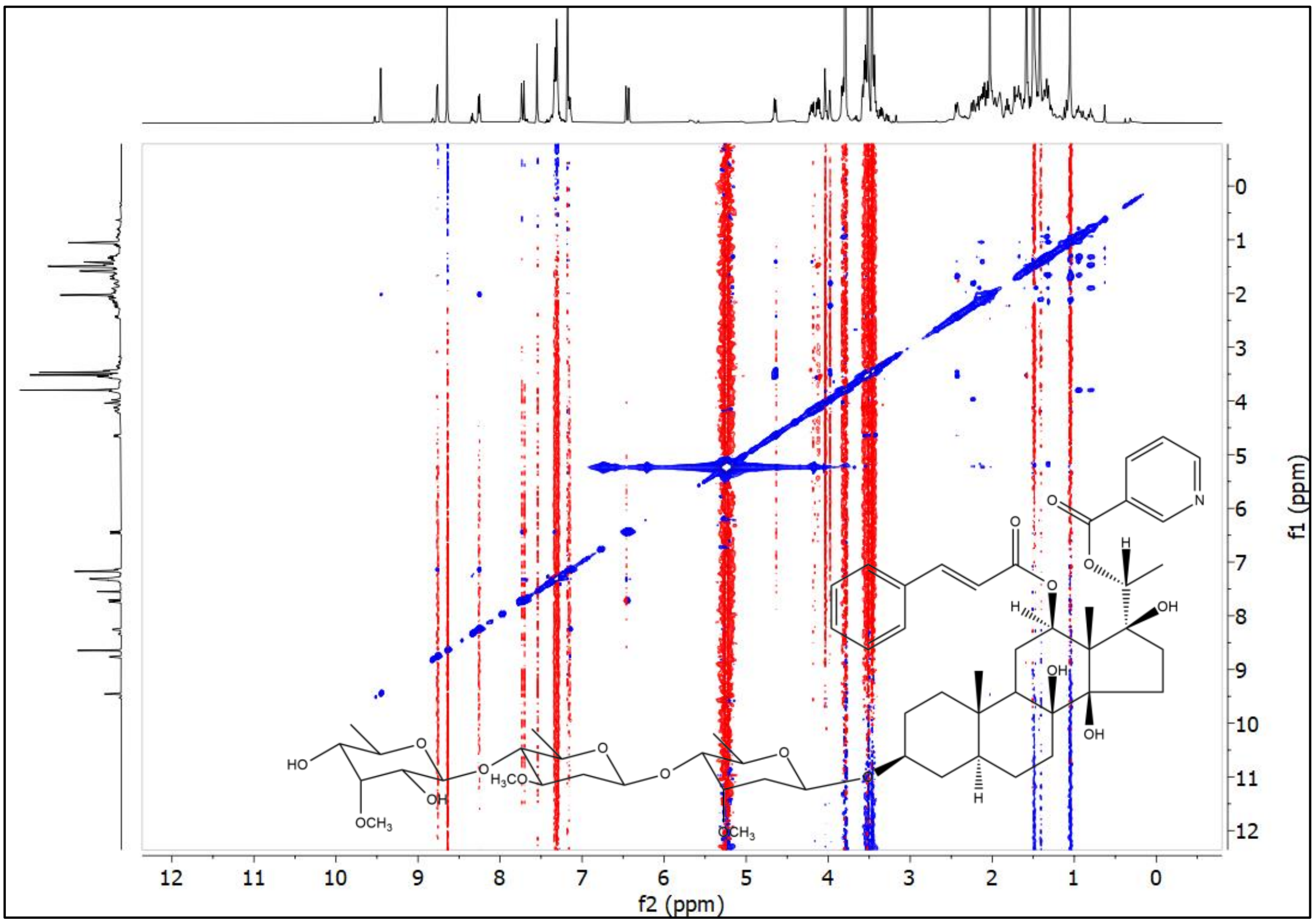

Figure S21. ATR-Infrared spectrum of compound 3.

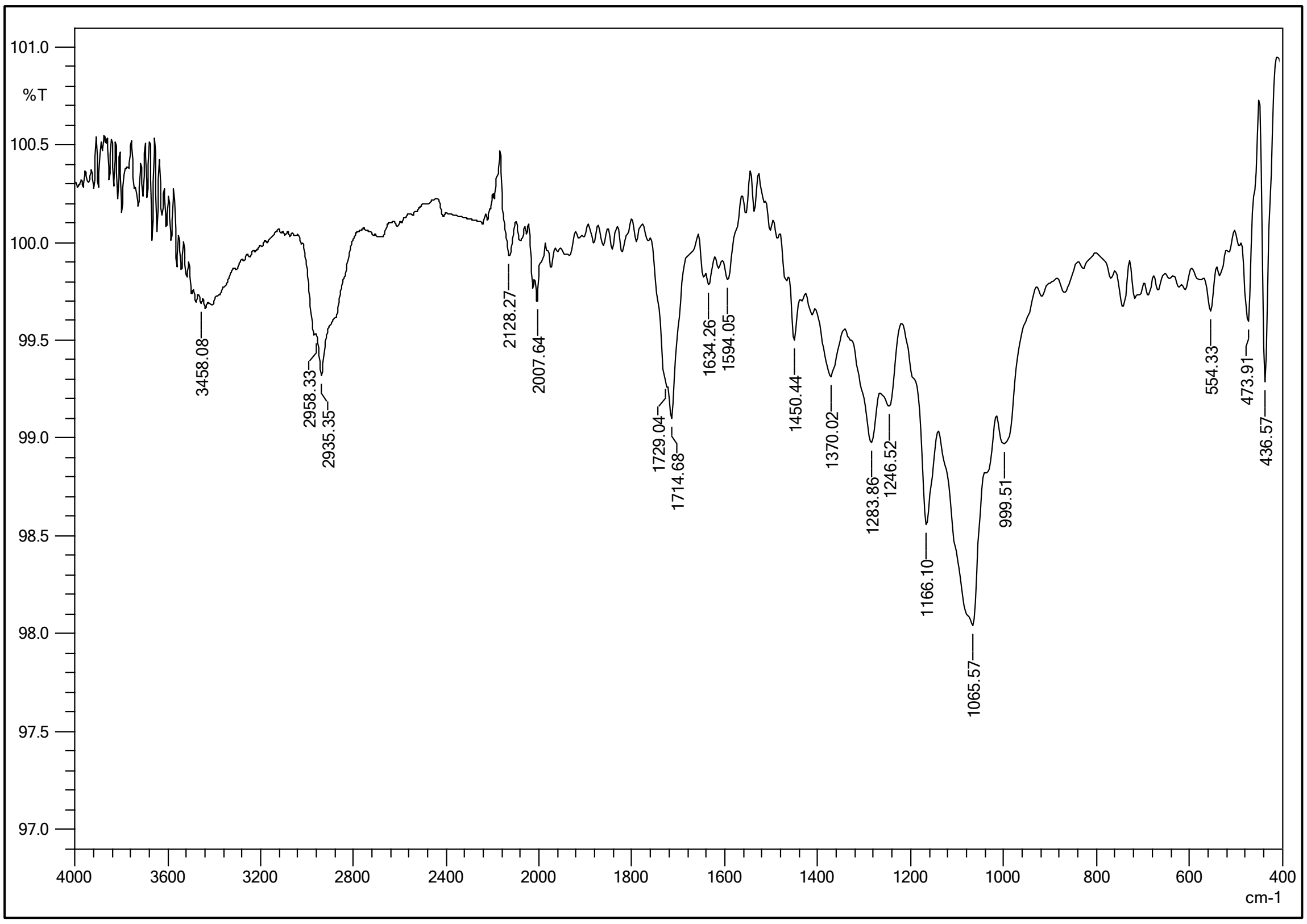

Figure S22. HR-ESI-MS mass spectrum of compound 3

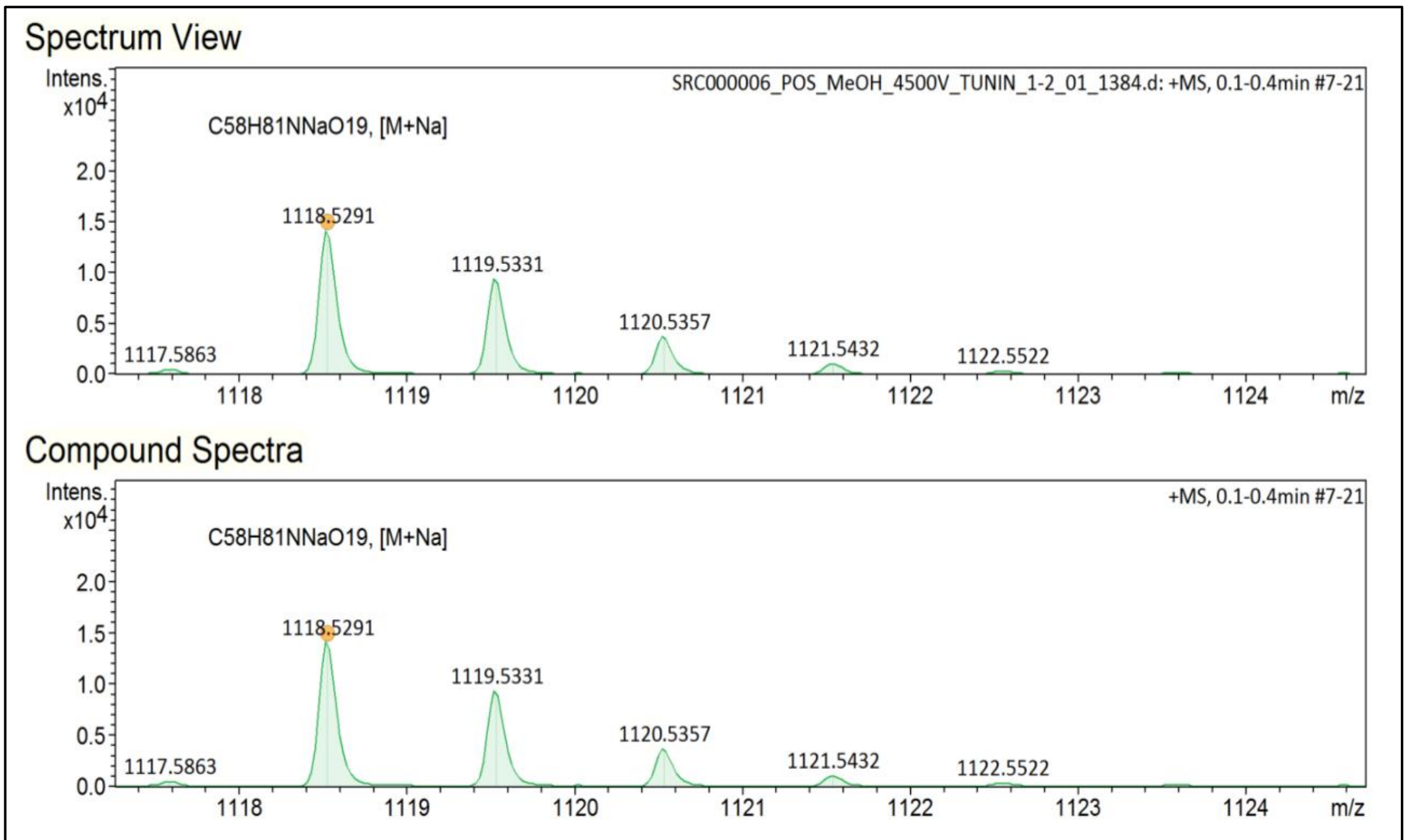

Meas. m/z	#	Ion Formula	m/z	err [ppm]	mSigma	# mSigma	Score	rdb	e⁻ Conf	N-Rule
1118.5291	1	C58H81NNaO19	1118.5295	0.3	12.8	1	100.00	18.5	even	ok

Figure S23. ^{1}H NMR spectrum (400 MHz, C_5D_5N) of compound 3

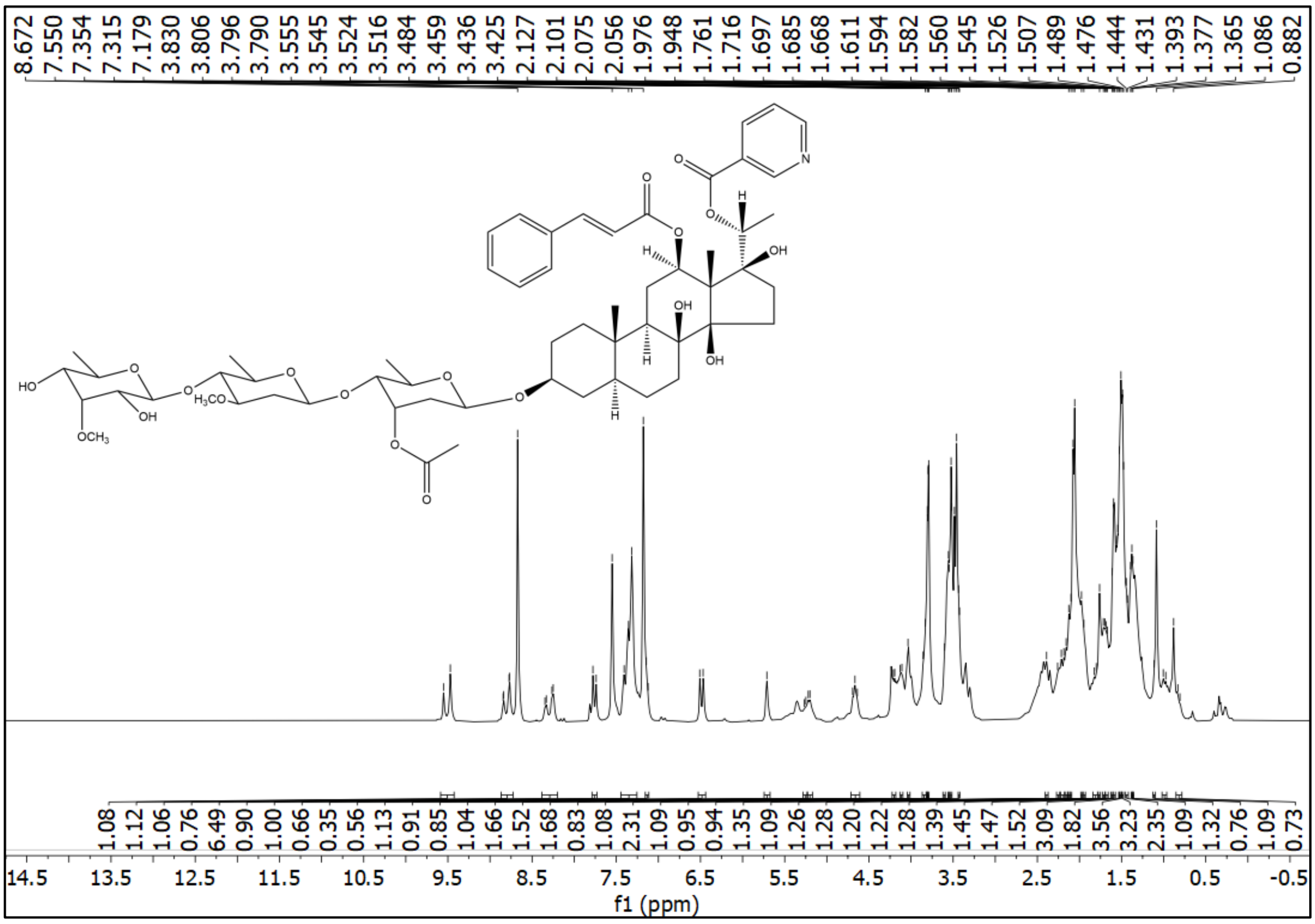

Figure S24. ^{13}C-BB NMR spectrum (100 MHz, C_5D_5N) of compound 3

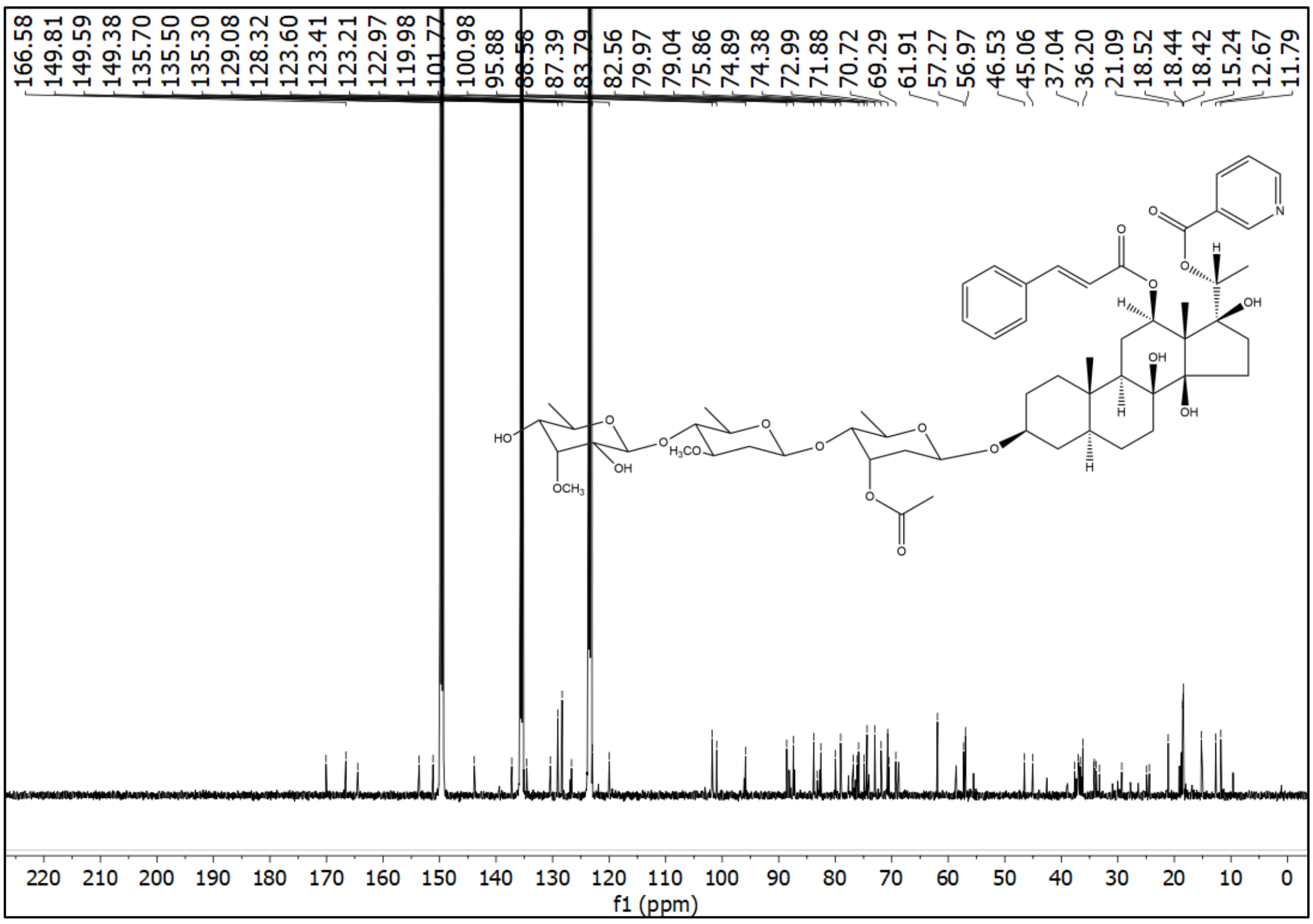

Figure S25. ^{13}C-DEPT135 NMR spectrum (100 MHz, C_5D_5N) of compound 3

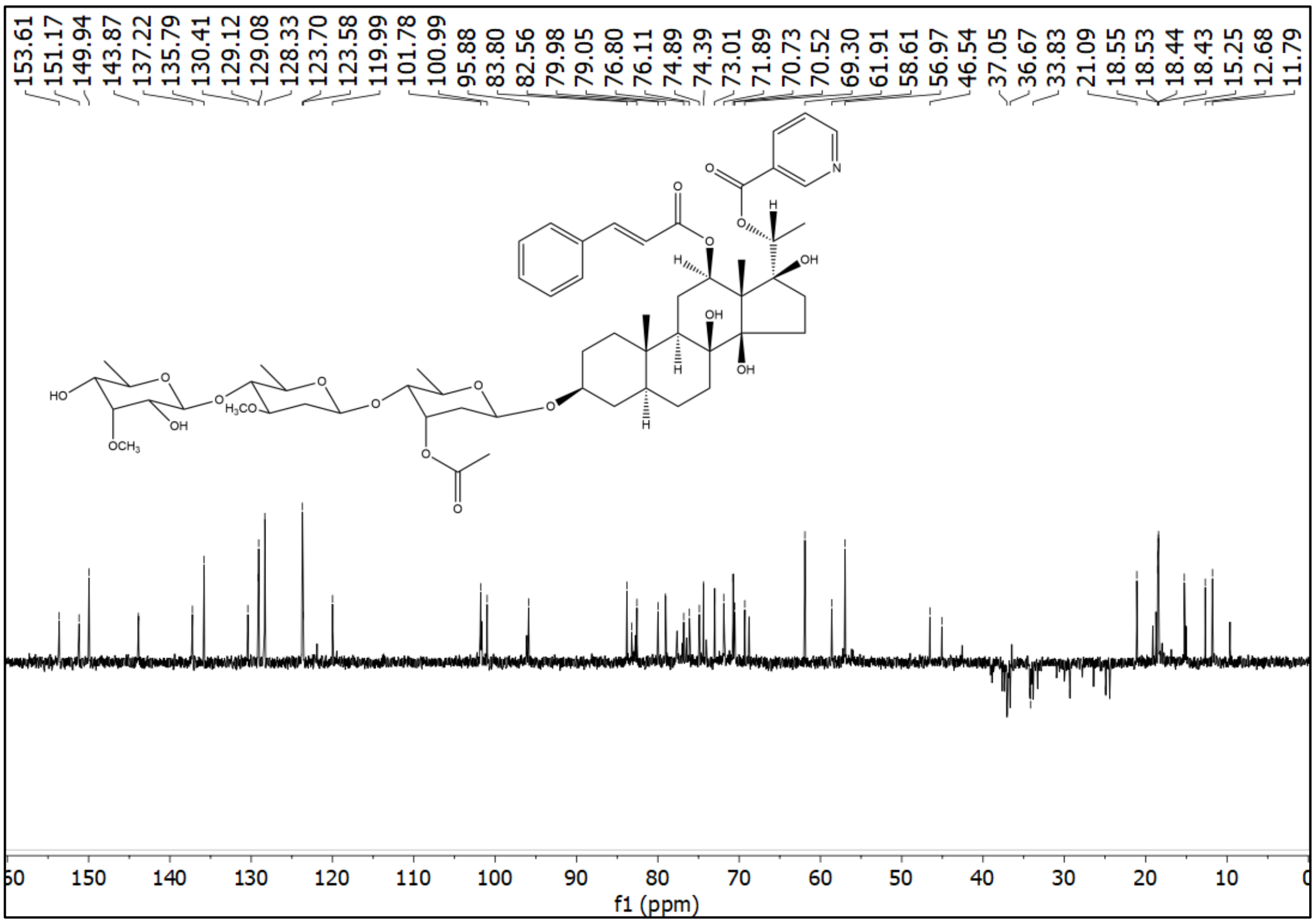

Figure S26. HSQC spectrum (400 x 100 MHz, C_5D_5N) of compound 3

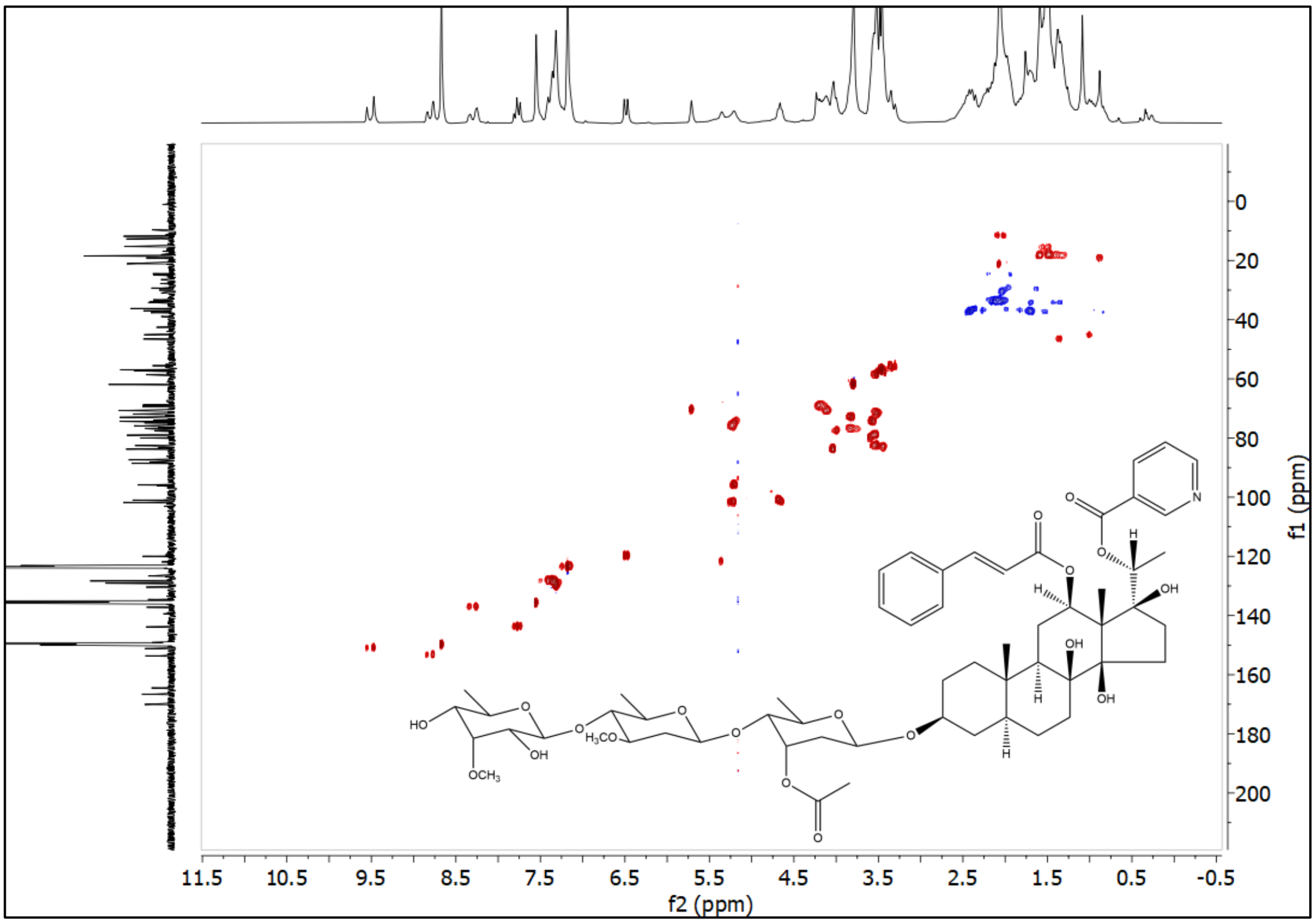

Figure S27. HMBC spectrum (400 x 100 MHz, C_5D_5N) of compound 3

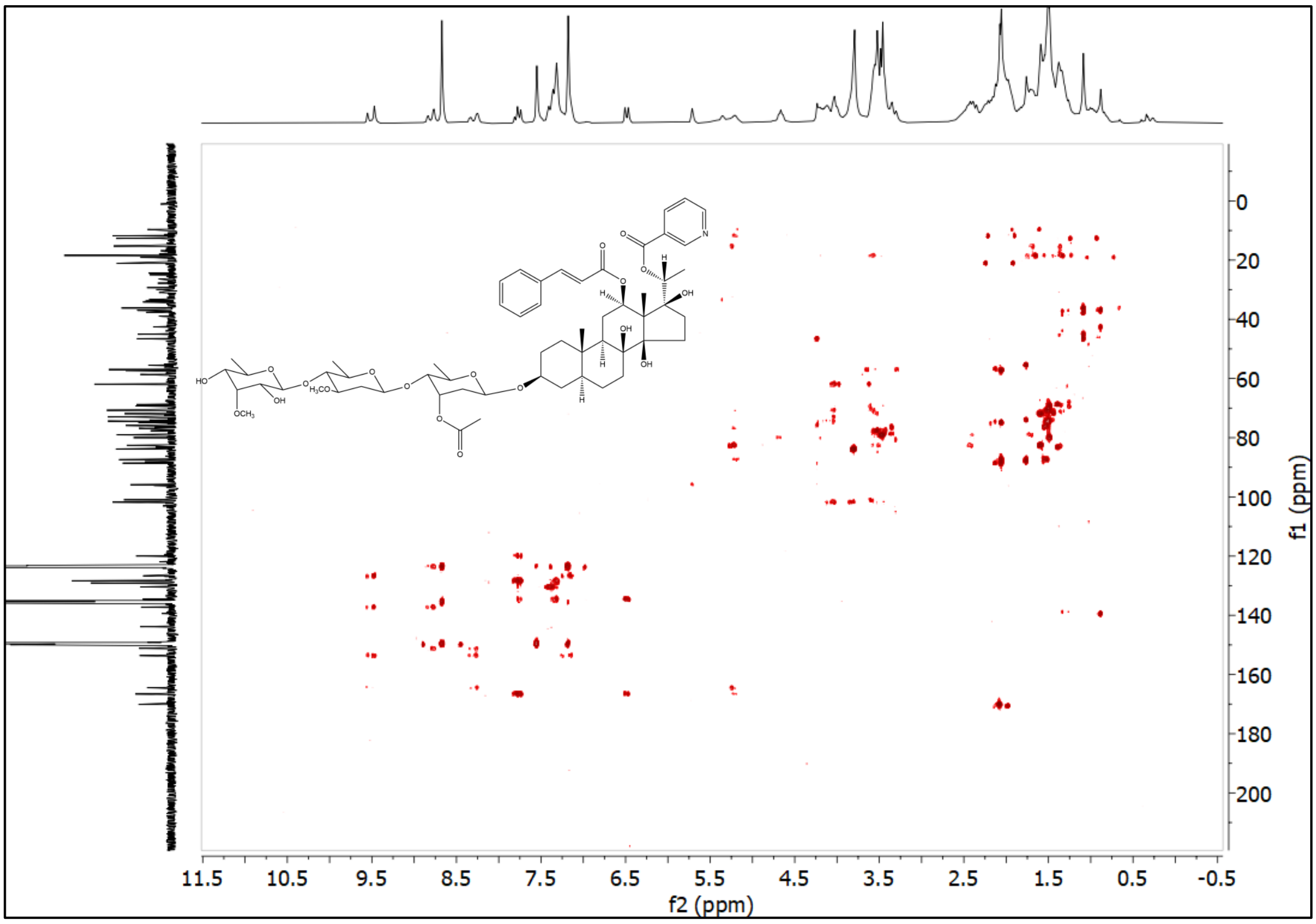

Figure S28. COSY spectrum (400 x 400 MHz, C_5D_5N) of compound 3.

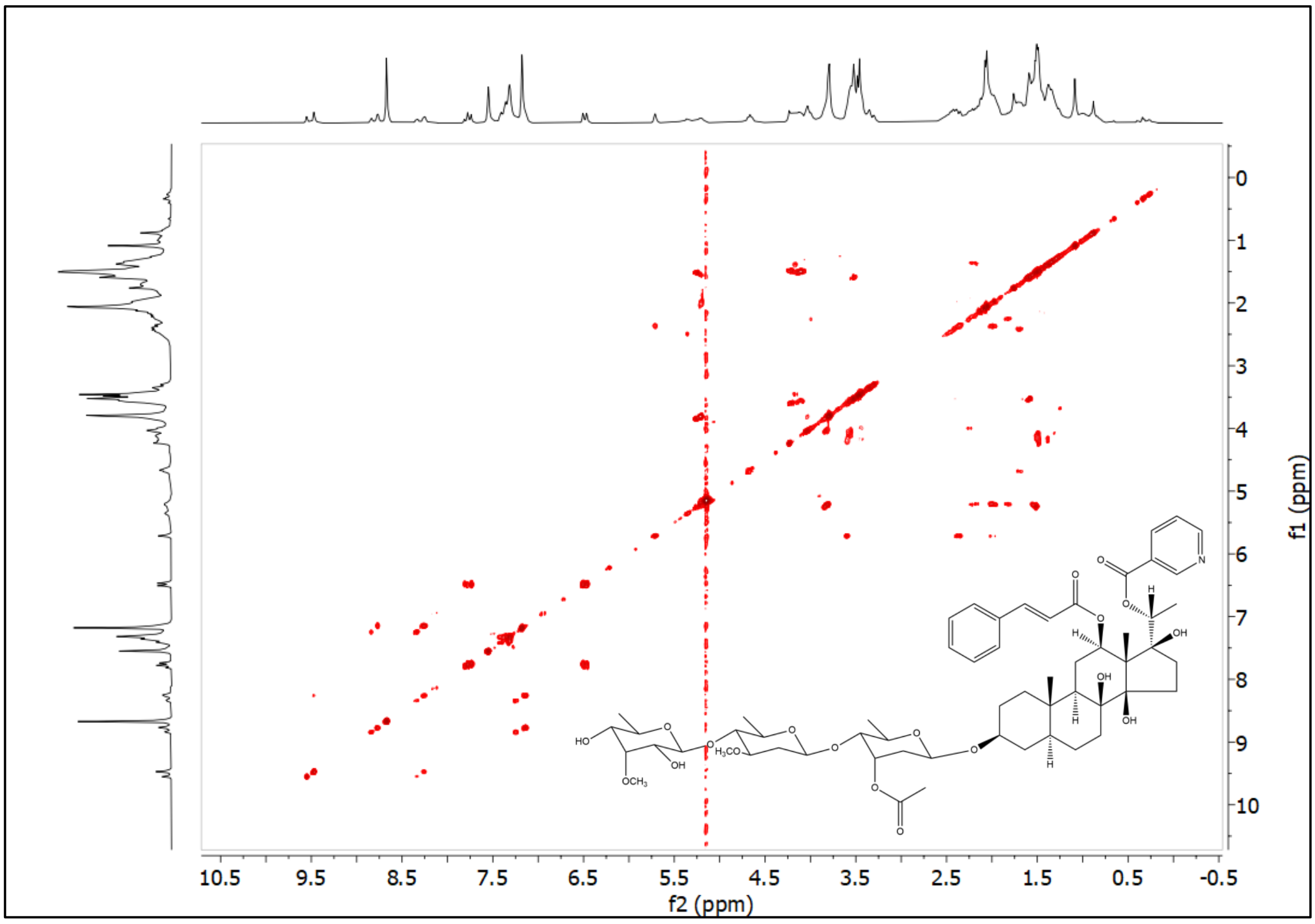

Figure S29. TOCSY spectrum (400 x 400 MHz, C_5D_5N) of compound 3.

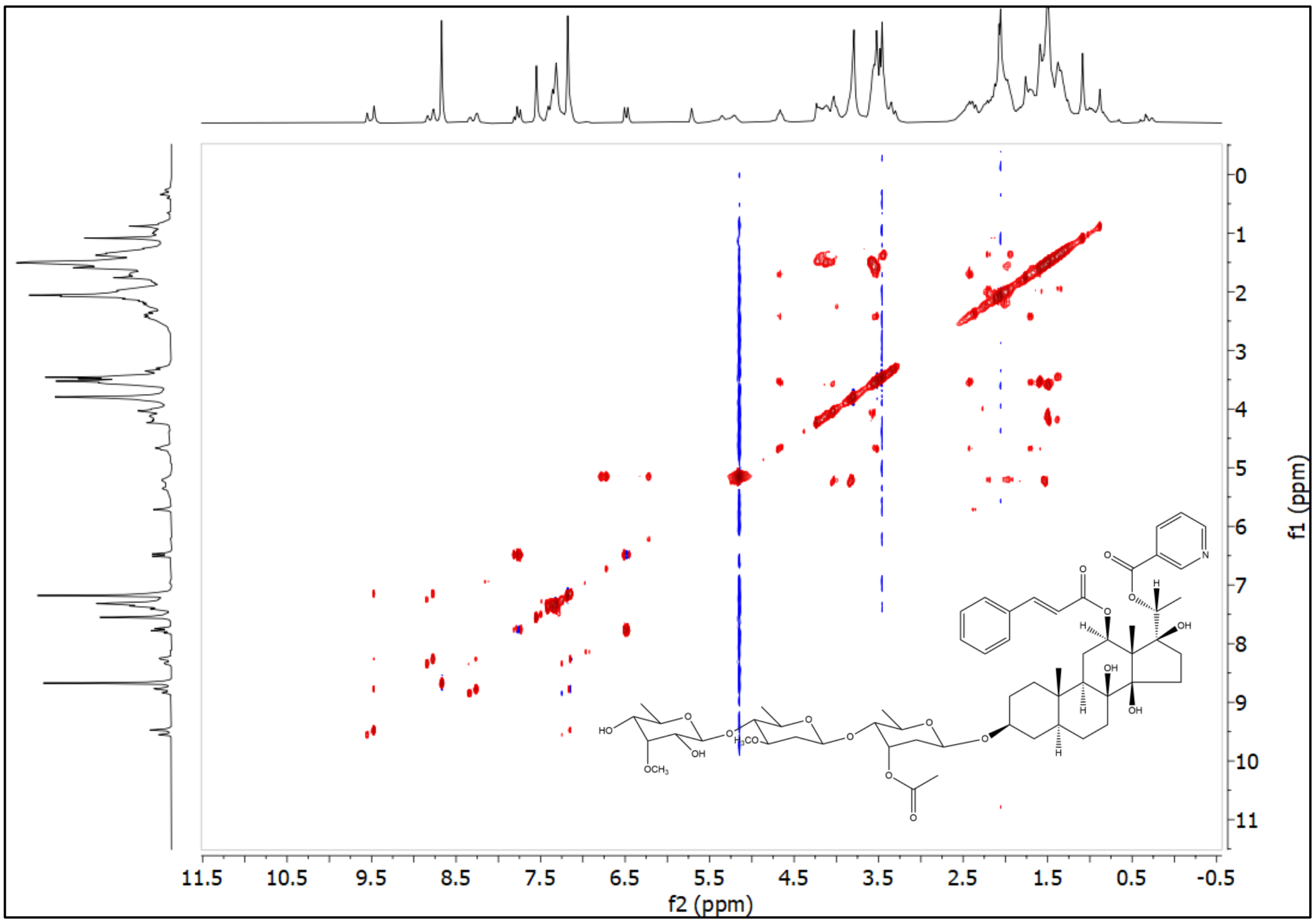

Figure S30. NOESY spectrum (400 x 400 MHz, C_5D_5N) of compound 3

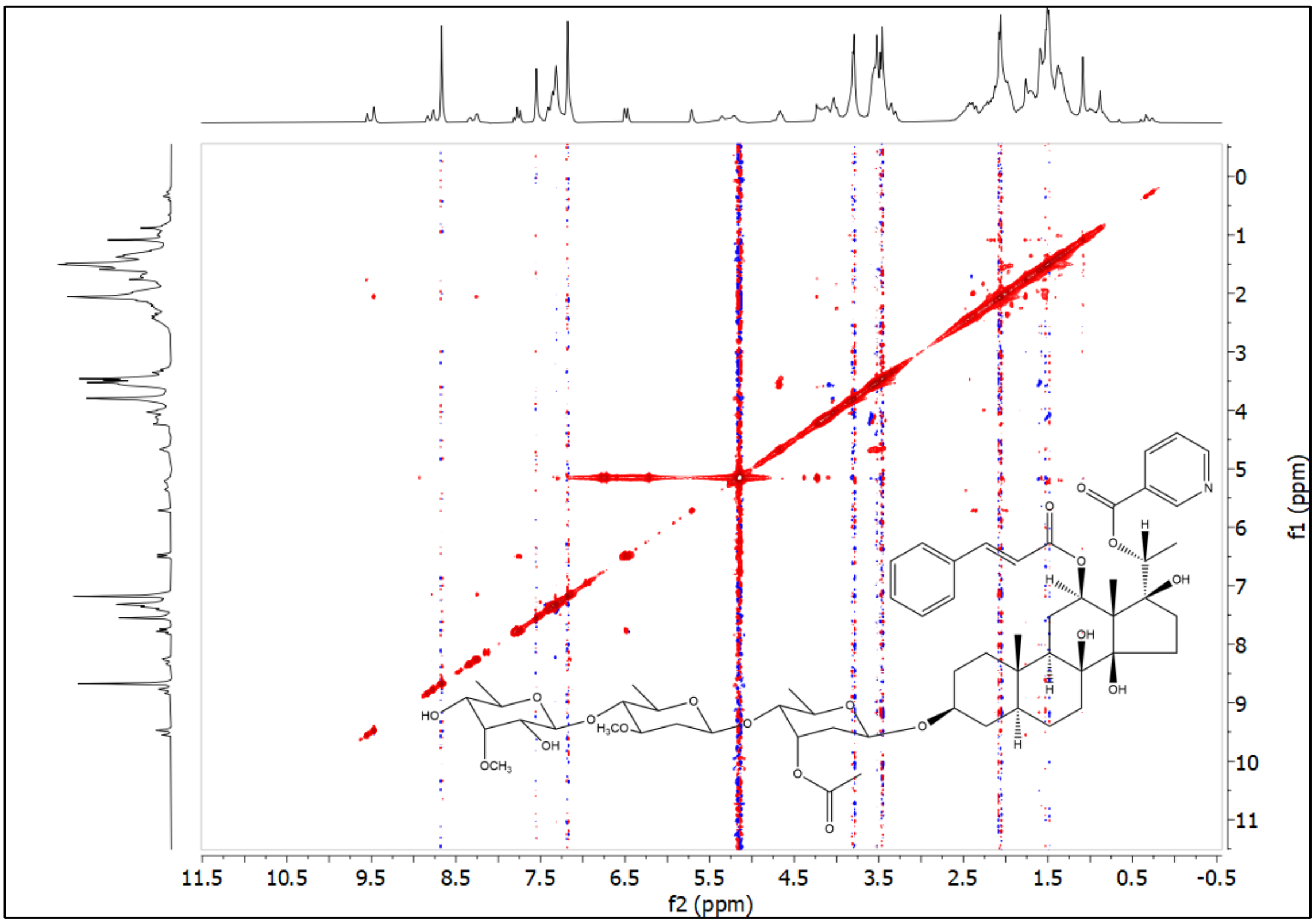

Figure S31. ATR-Infrared spectrum of compound 4.

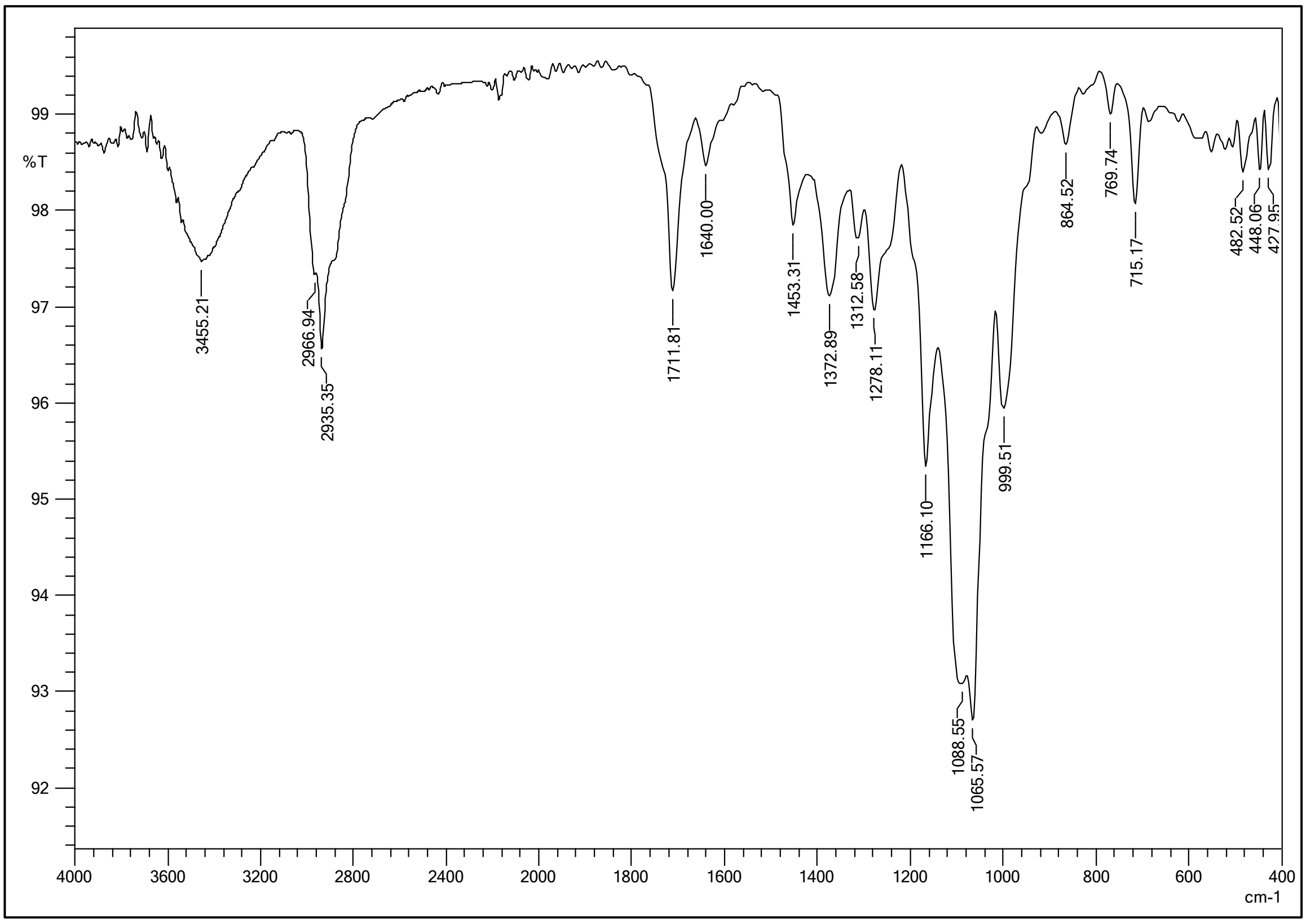

Figure S32. HR-ESI-MS mass spectrum of compound 4.

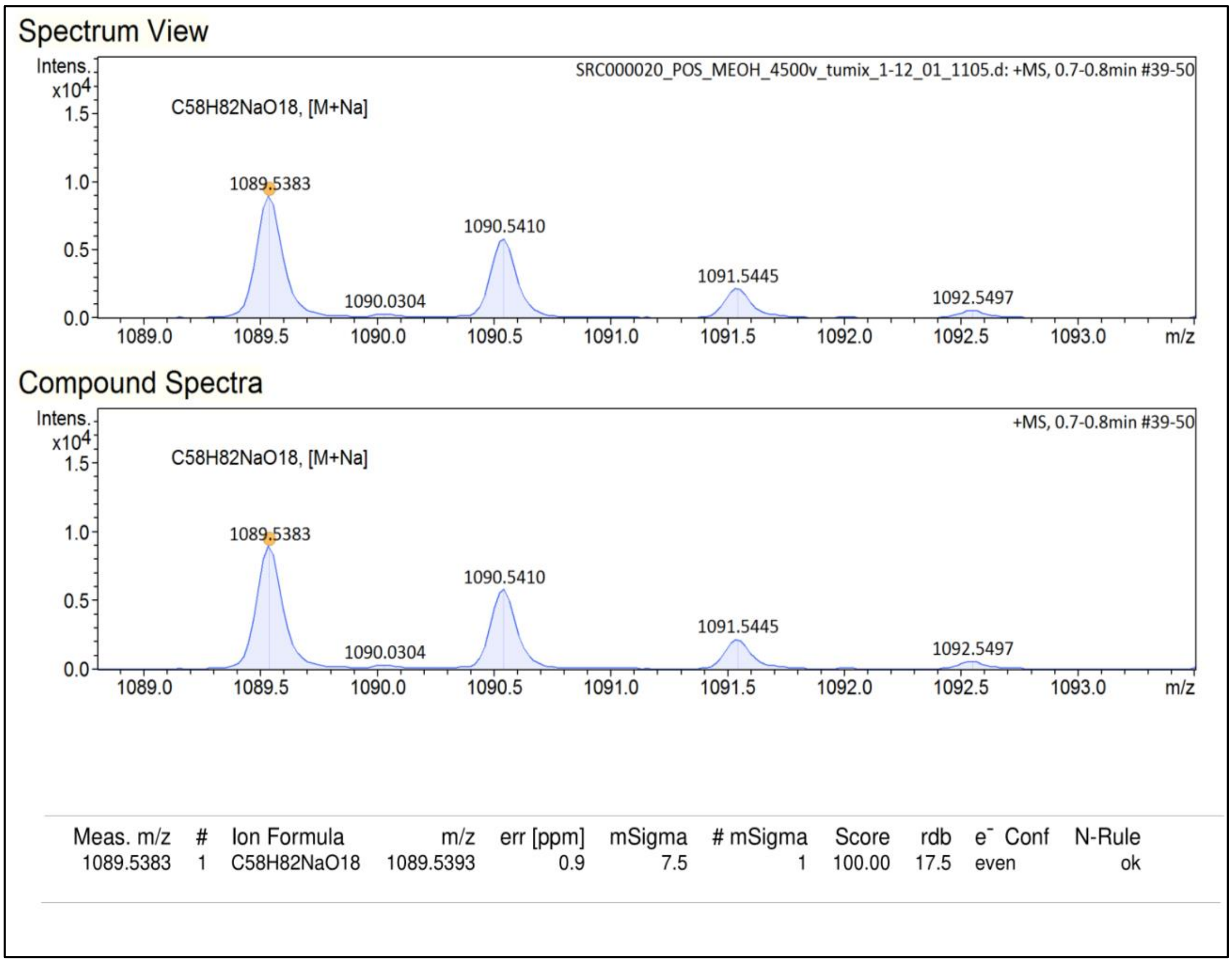

Figure S33. ^{1}H NMR spectrum (500 MHz, C_5D_5N) of compound 4

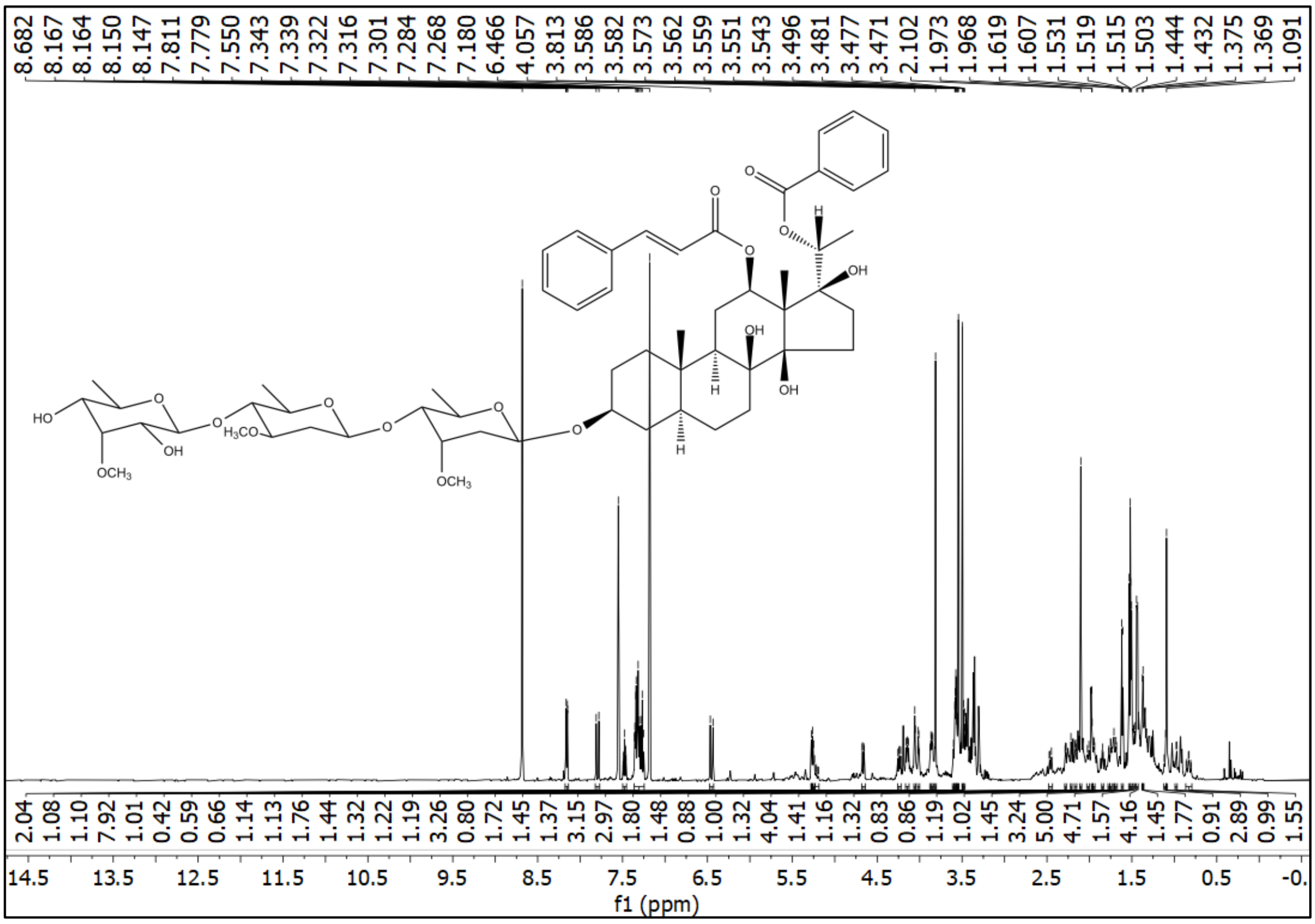

Figure S34. ^{13}C-BB NMR spectrum (125 MHz, C_5D_5N) of compound 4

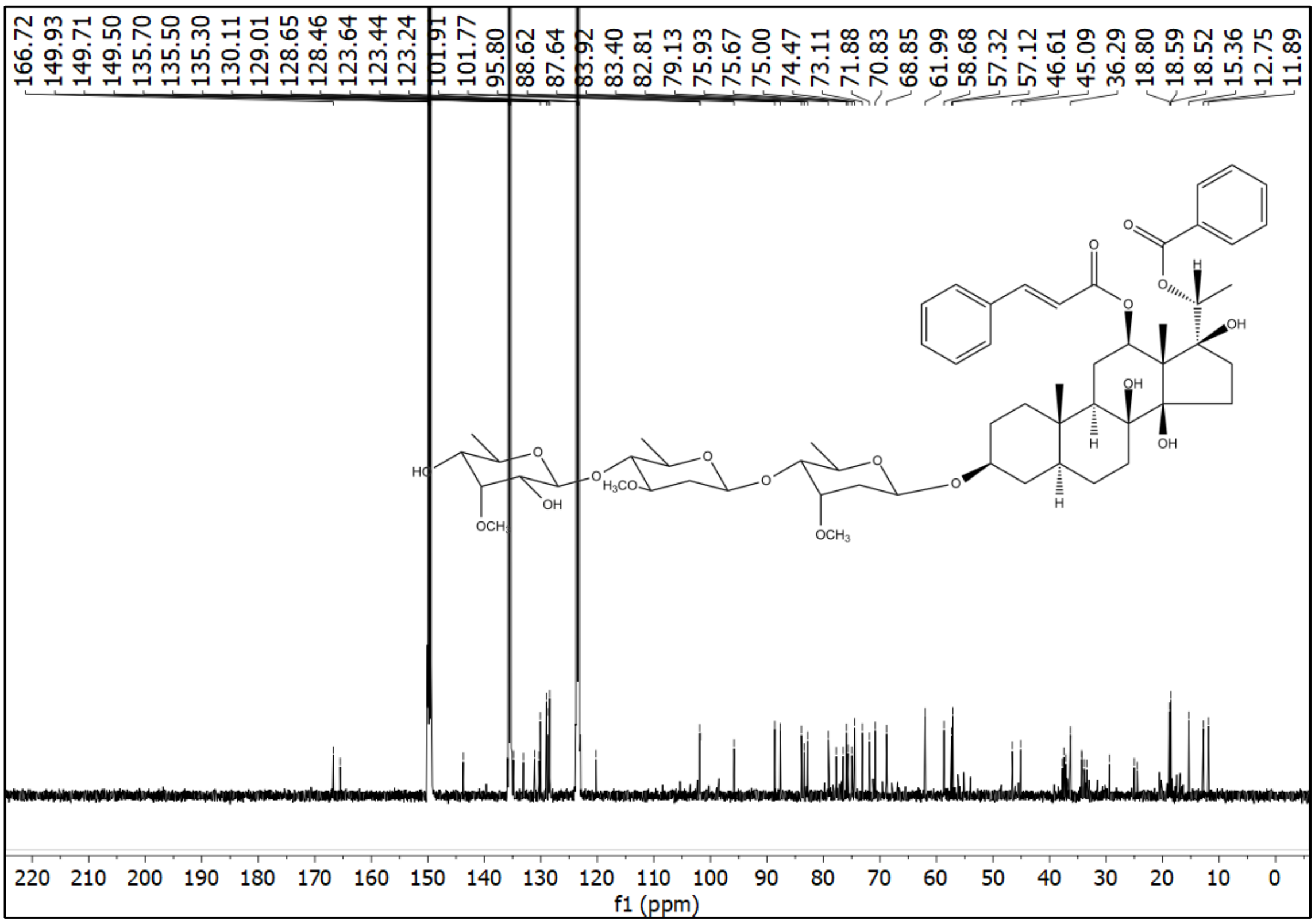

Figure S35. ^{13}C-DEPT135 NMR spectrum (125 MHz, C_5D_5N) of compound 4

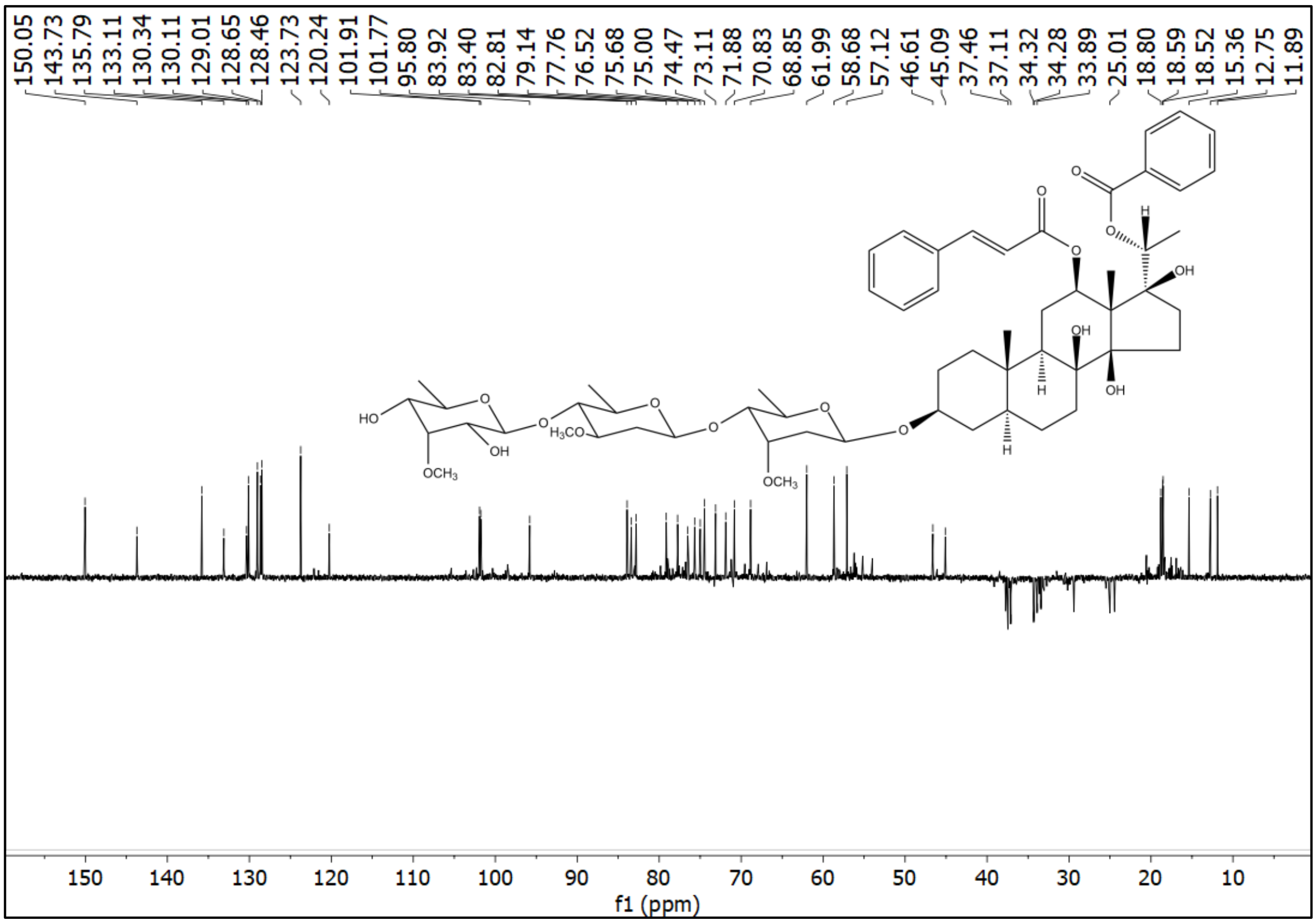

Figure S36. HSQC spectrum (500 x 125 MHz, C_5D_5N) of compound 4

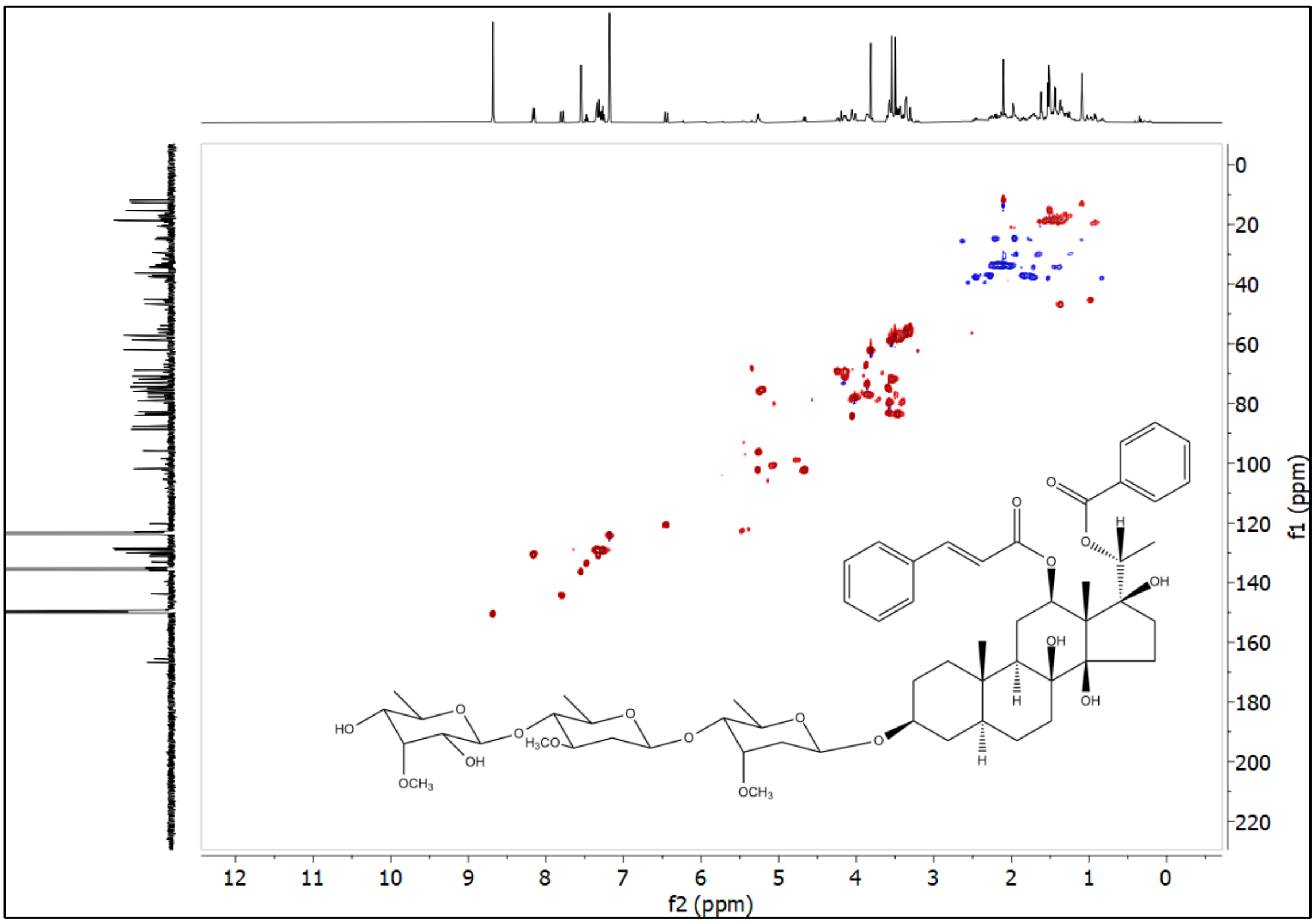

Figure S37. HMBC spectrum (500 x 125 MHz, C_5D_5N) of compound 4

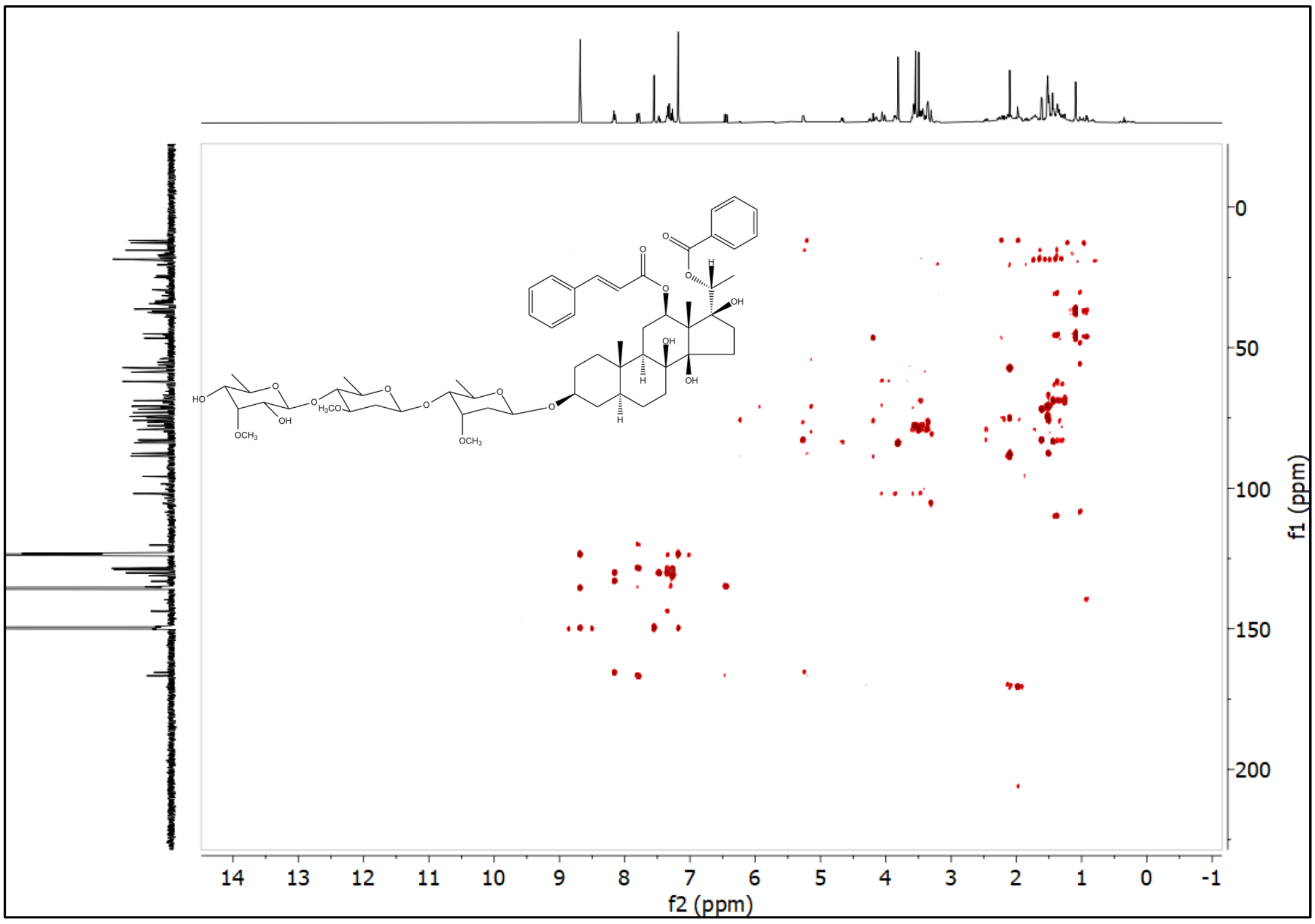

Figure S38. COSY spectrum (500 x 500 MHz, C_5D_5N) of compound 4

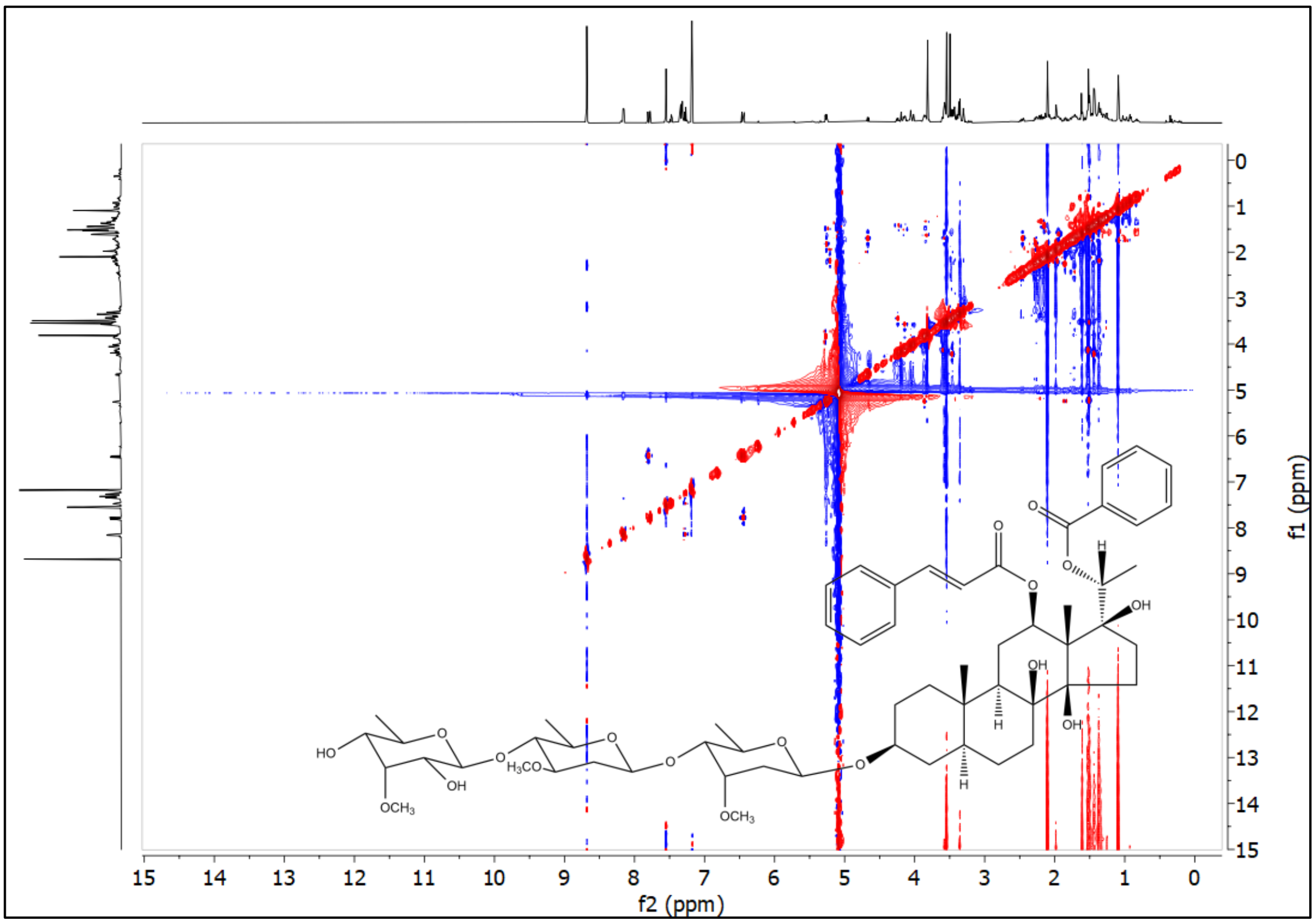

Figure S39. TOCSY spectrum (500 x 500 MHz, C_5D_5N) of compound 4

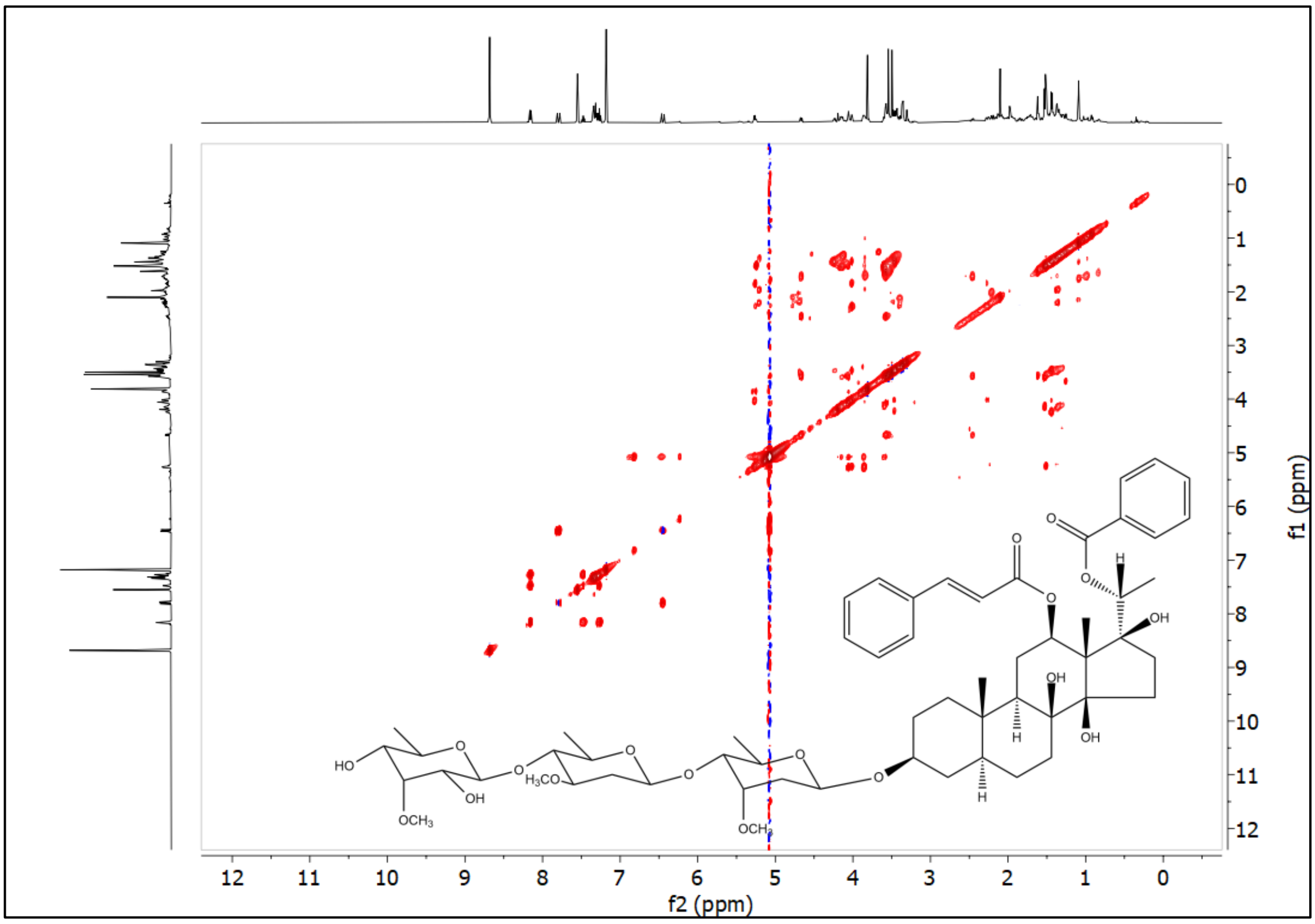

Figure S40. NOESY spectrum (500 x 500 MHz, C_5D_5N) of compound 4

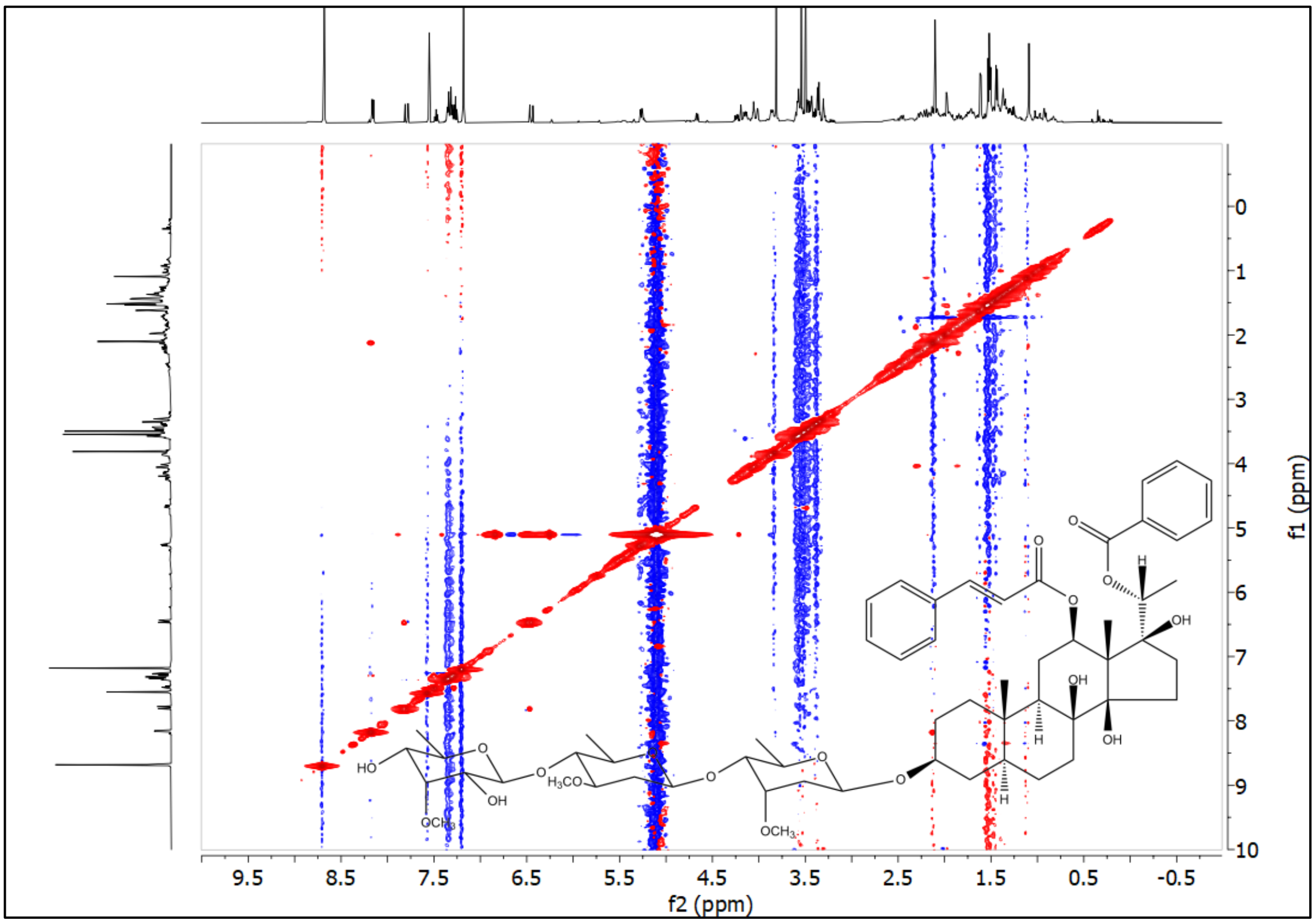

Figure S41. ATR-Infrared spectrum of compound 5

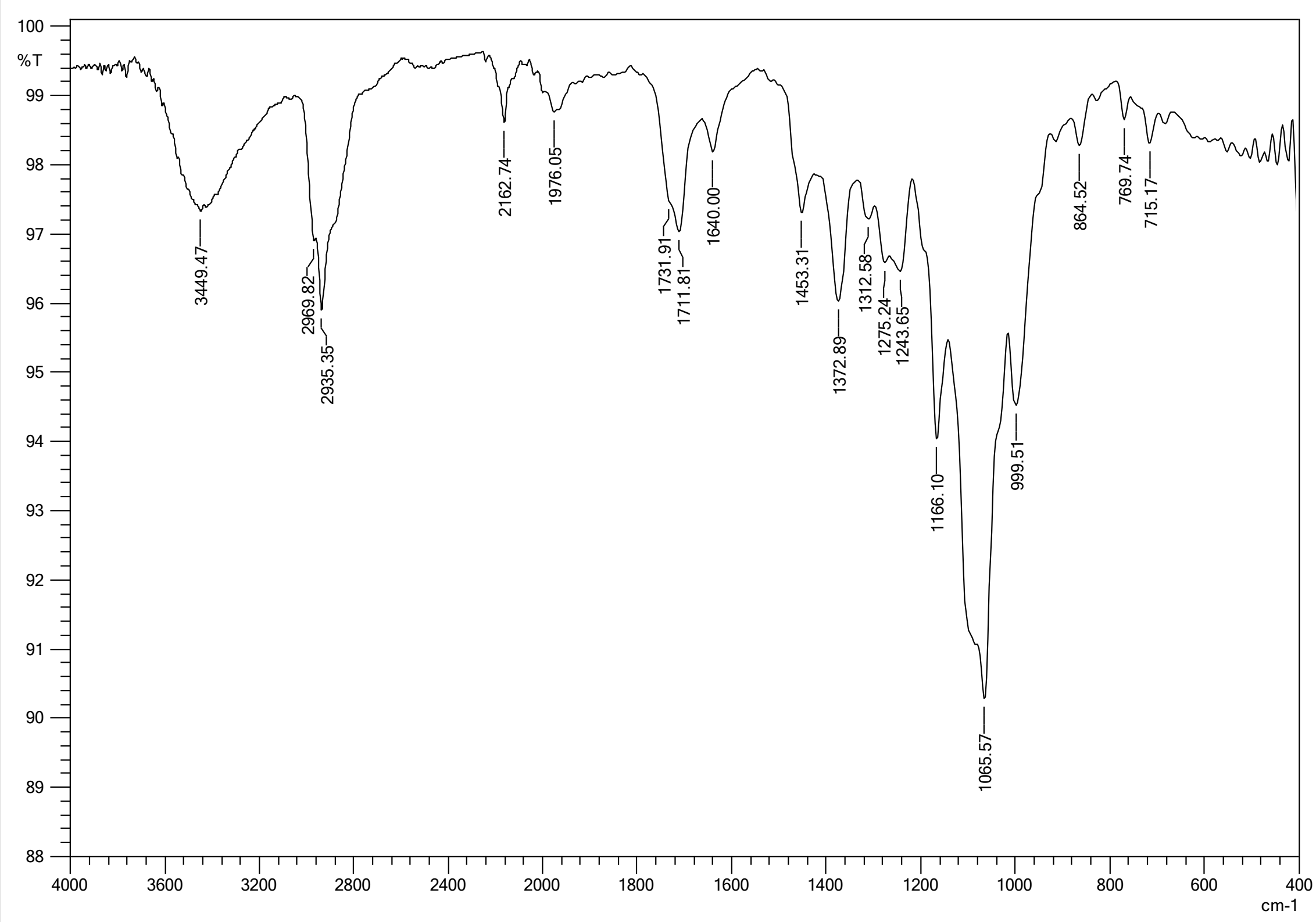

Figure S42. HR-ESI-MS mass spectrum of compound 5.

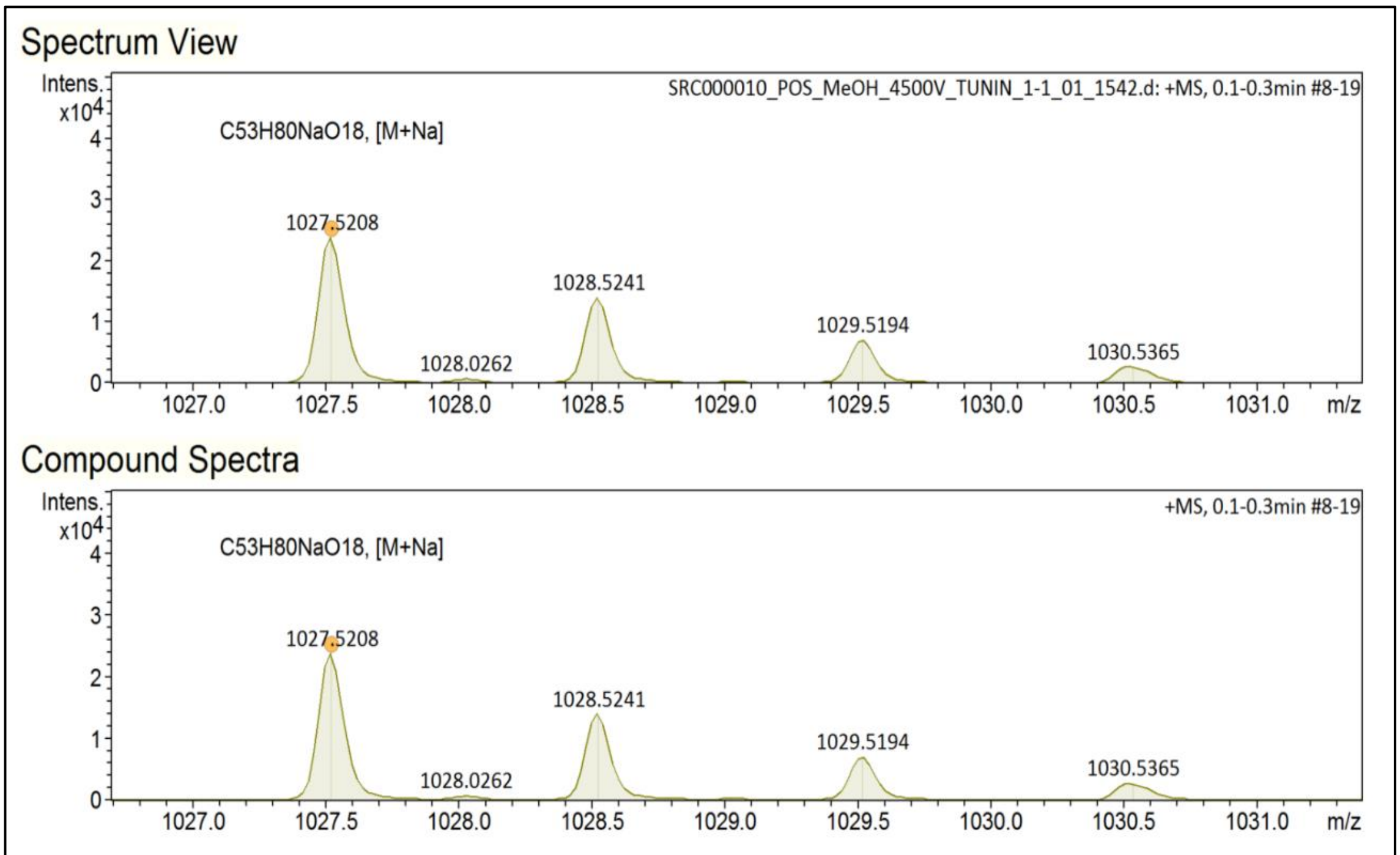

Meas. m/z	#	Ion Formula	m/z	err [ppm]	mSigma	# mSigma	Score	rdb	e⁻ Conf	N-Rule
1027.5208	1	C53H80NaO18	1027.5237	2.8	47.0	1	100.00	13.5	even	ok

Figure S43. ^{1}H NMR spectrum (400 MHz, C_5D_5N) of compound 5

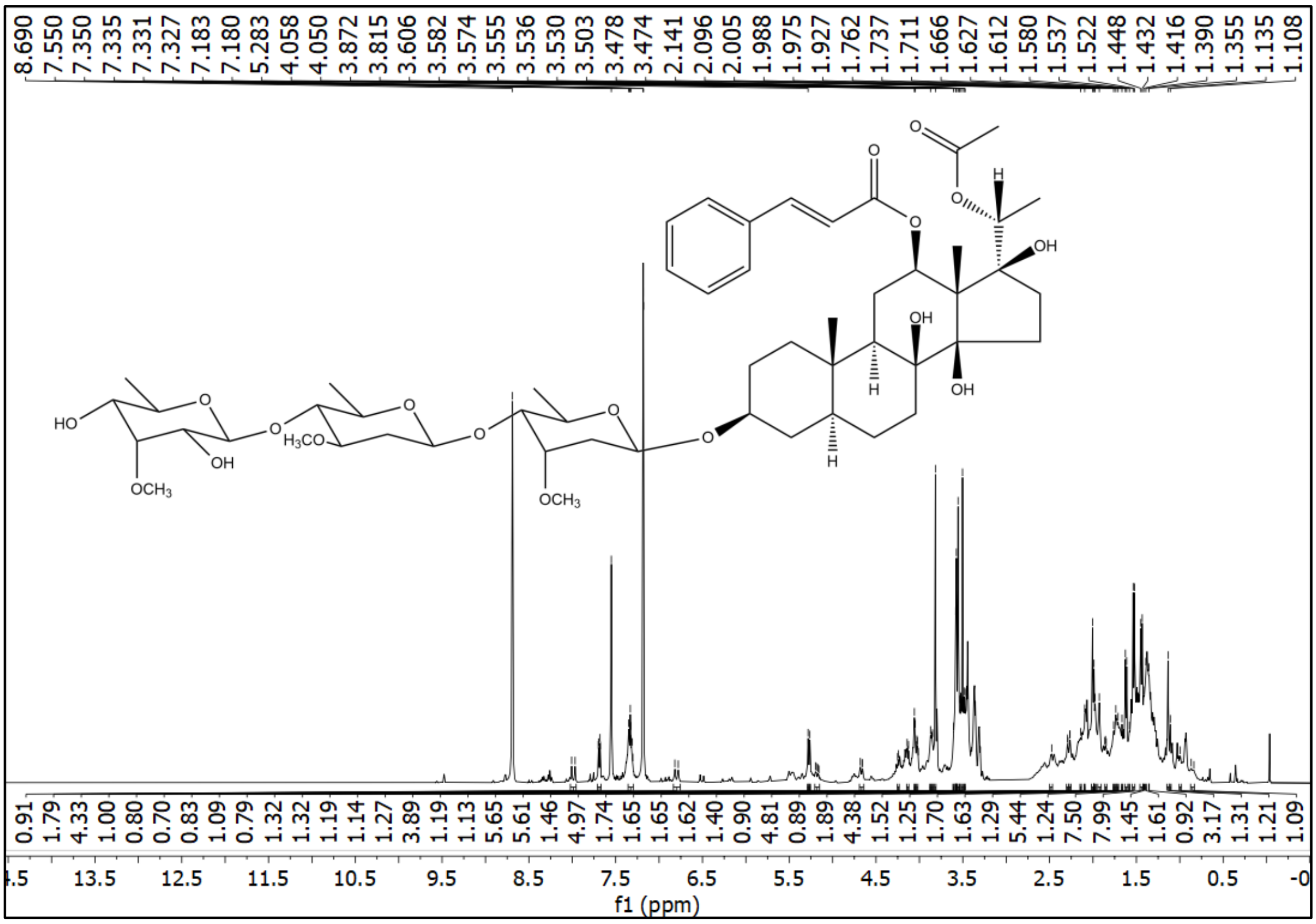

Figure S44. ^{13}C-BB NMR spectrum (100 MHz, C_5D_5N) of compound 5

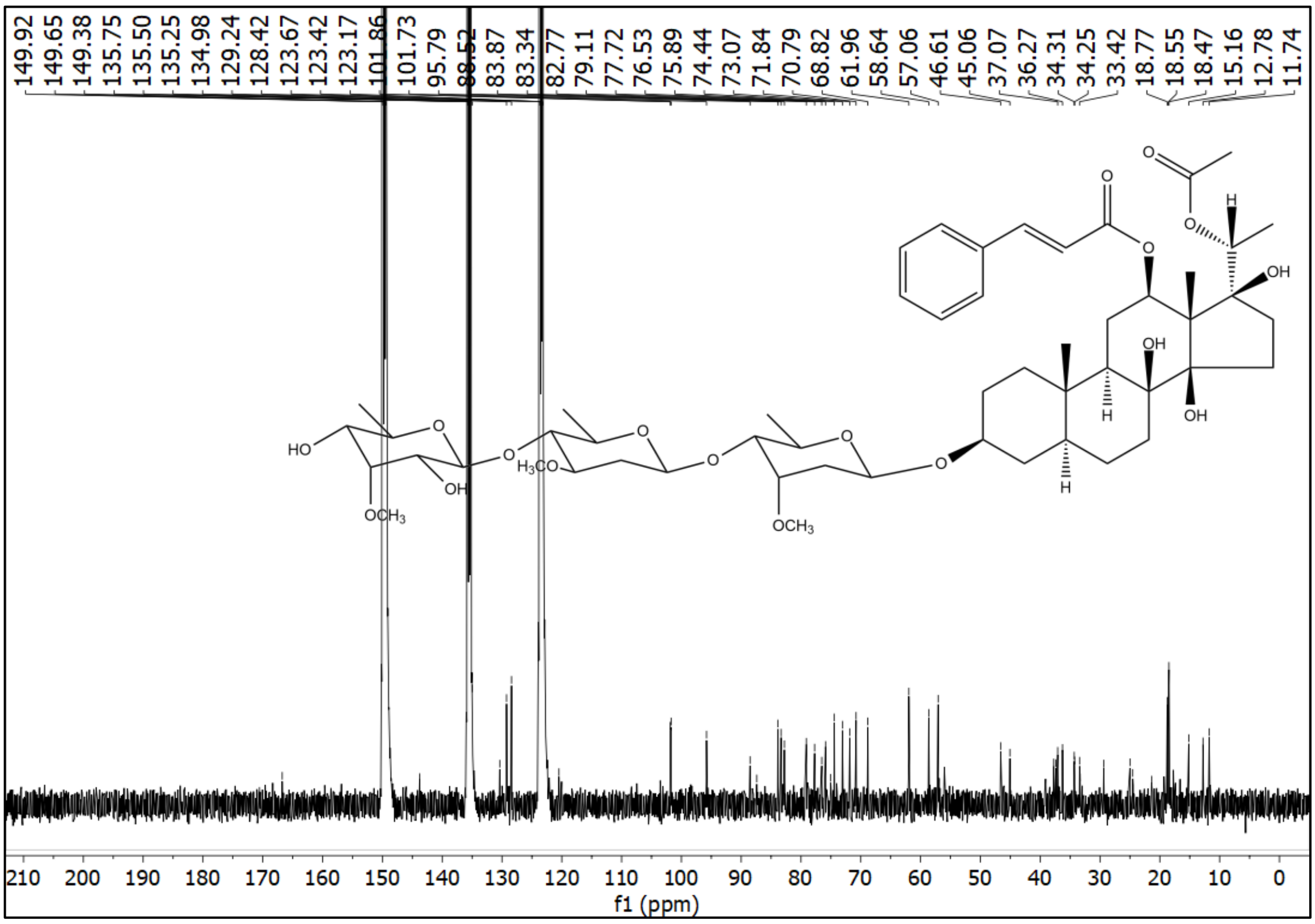

Figure S45. ^{13}C-DEPT135 NMR spectrum (100 MHz, C_5D_5N) of compound 5

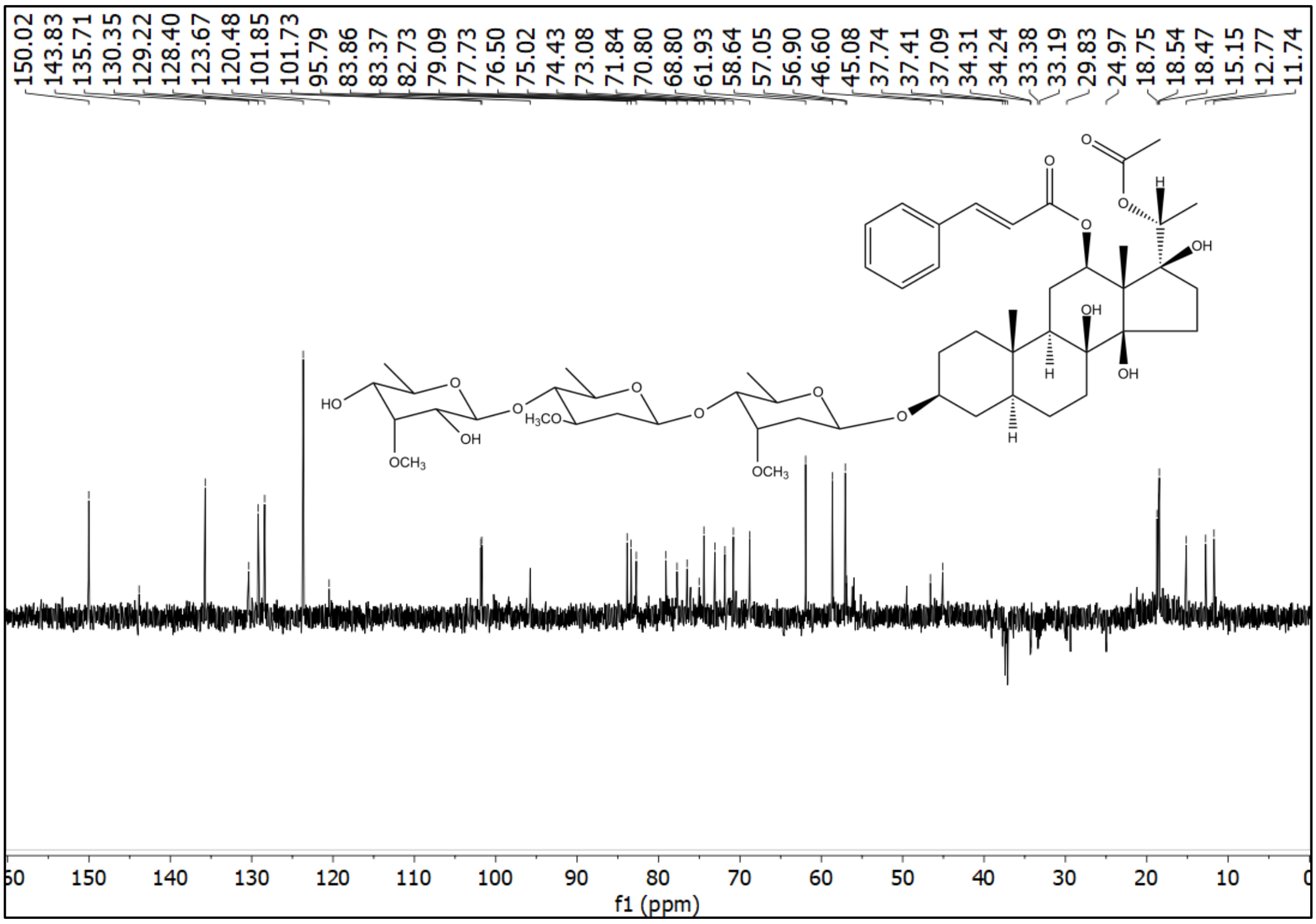

Figure S46. HSQC spectrum (400 x 100 MHz, C_5D_5N) of compound 5

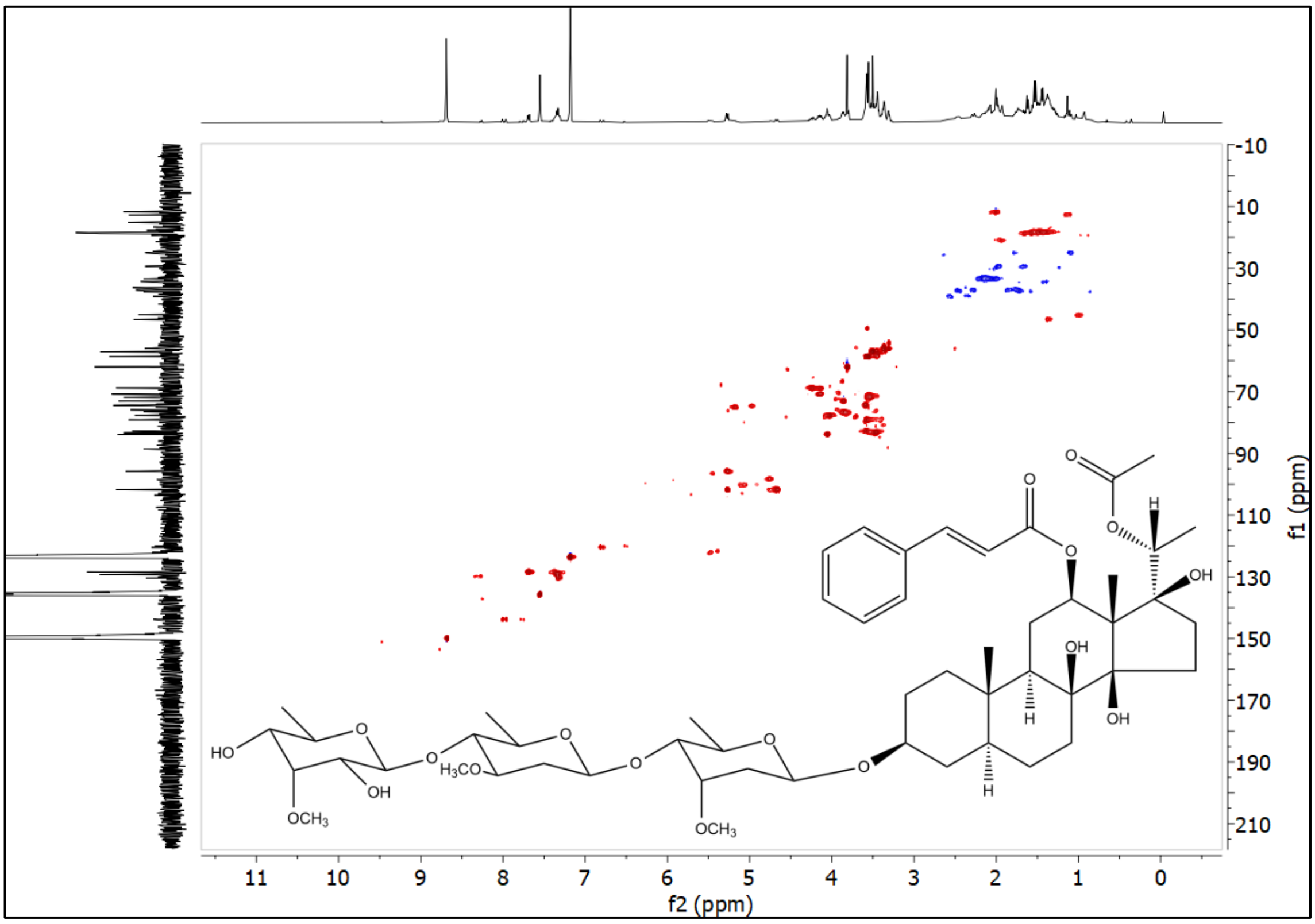

Figure S47. HMBC spectrum (400 x 100 MHz, C_5D_5N) of compound 5

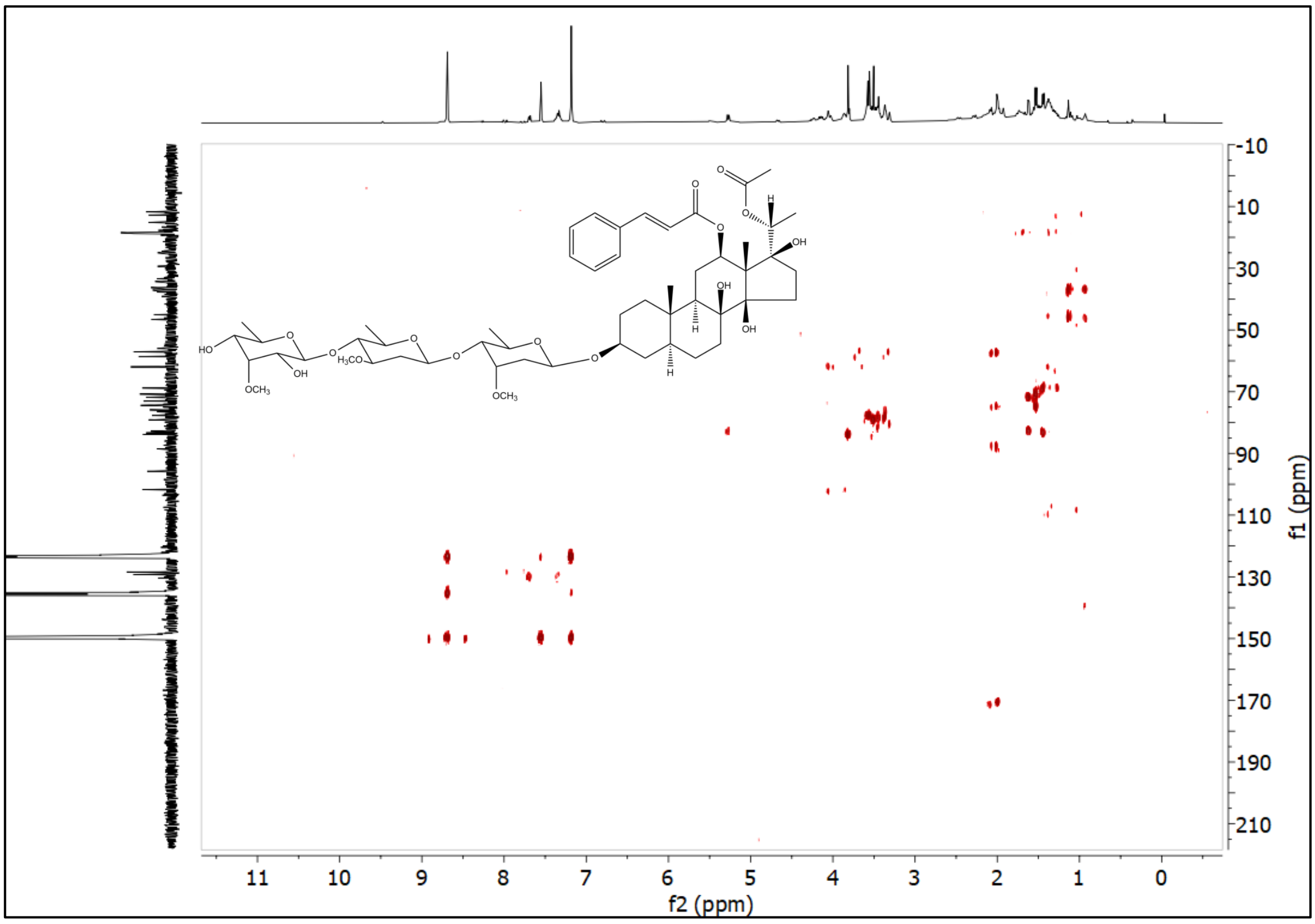

Figure S48. COSY spectrum (400 x 400 MHz, C_5D_5N) of compound 5.

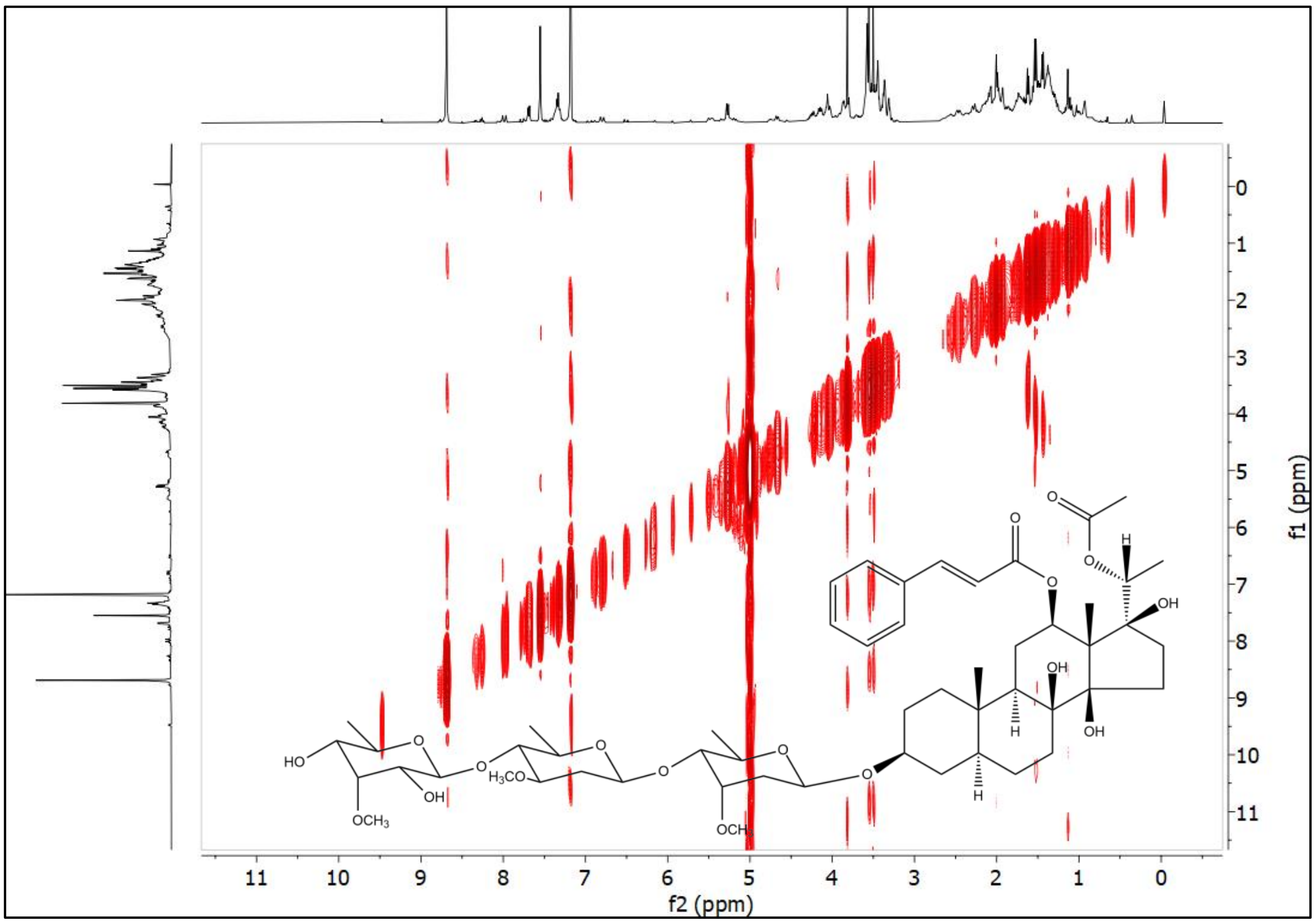

Figure S49. TOCSY spectrum (400 x 400 MHz, C_5D_5N) of compound 5.

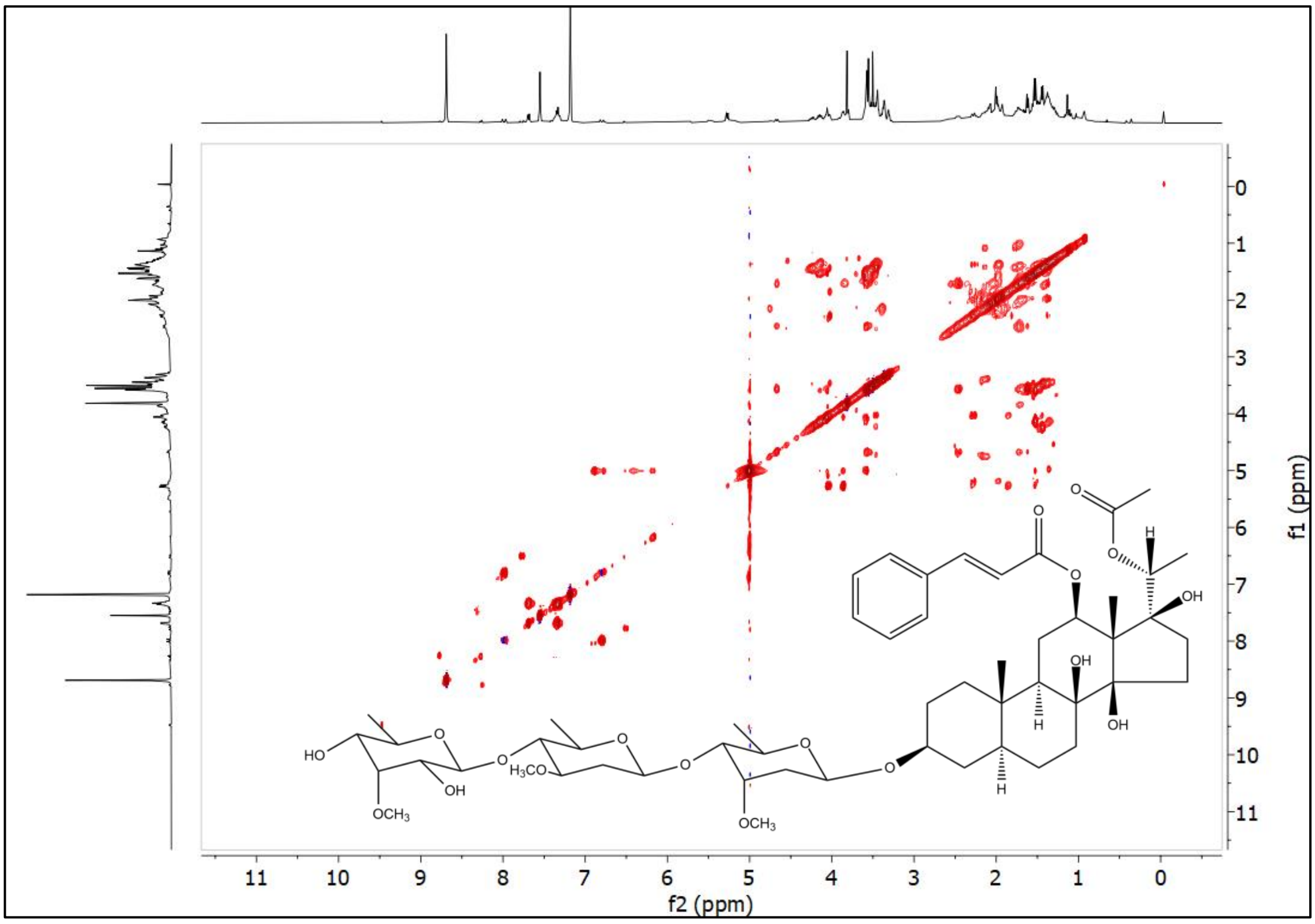

Figure S50. NOESY spectrum (400 x 400 MHz, C_5D_5N) of compound 5.

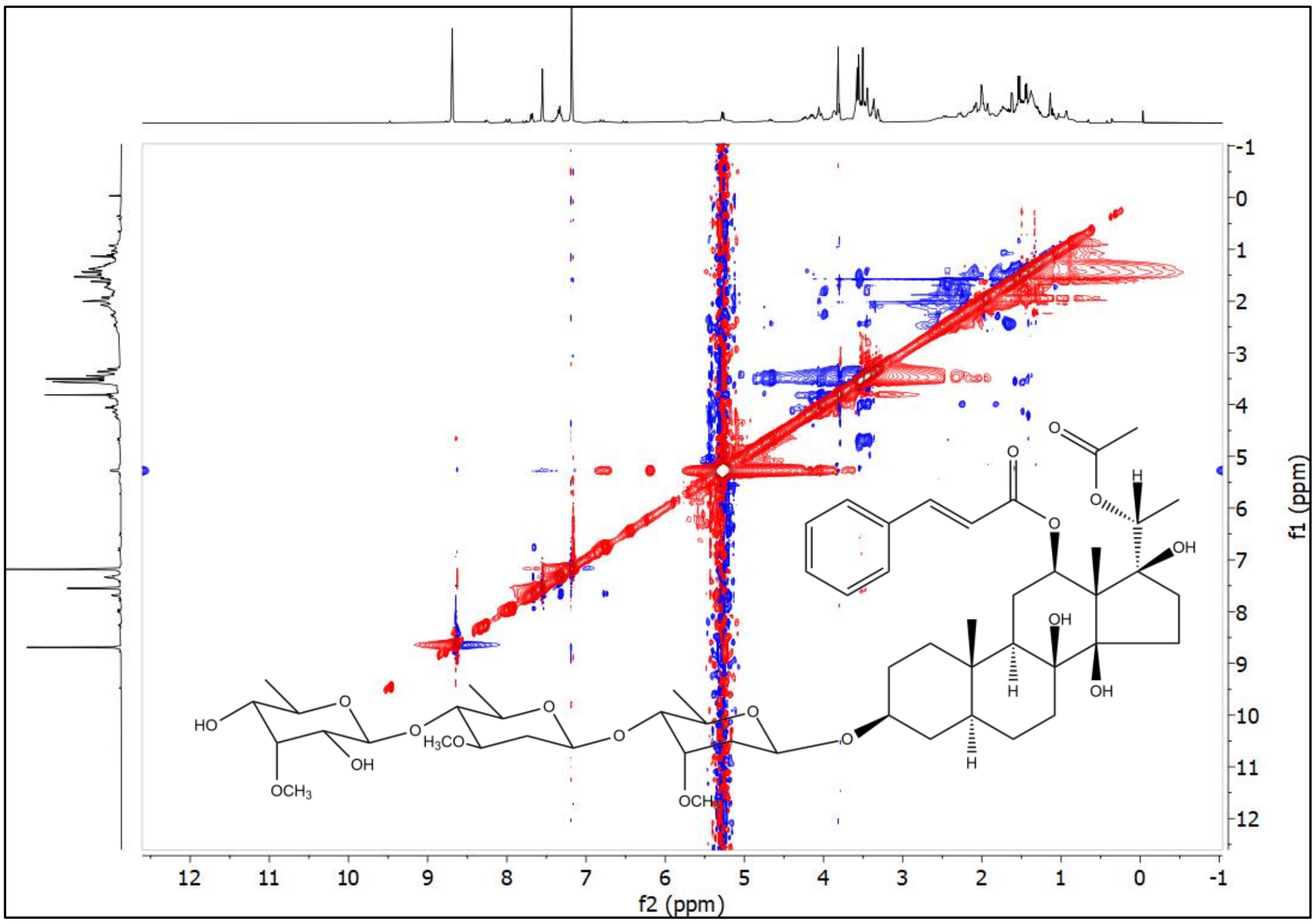

Figure S51. ATR-Infrared spectrum of compound 6.

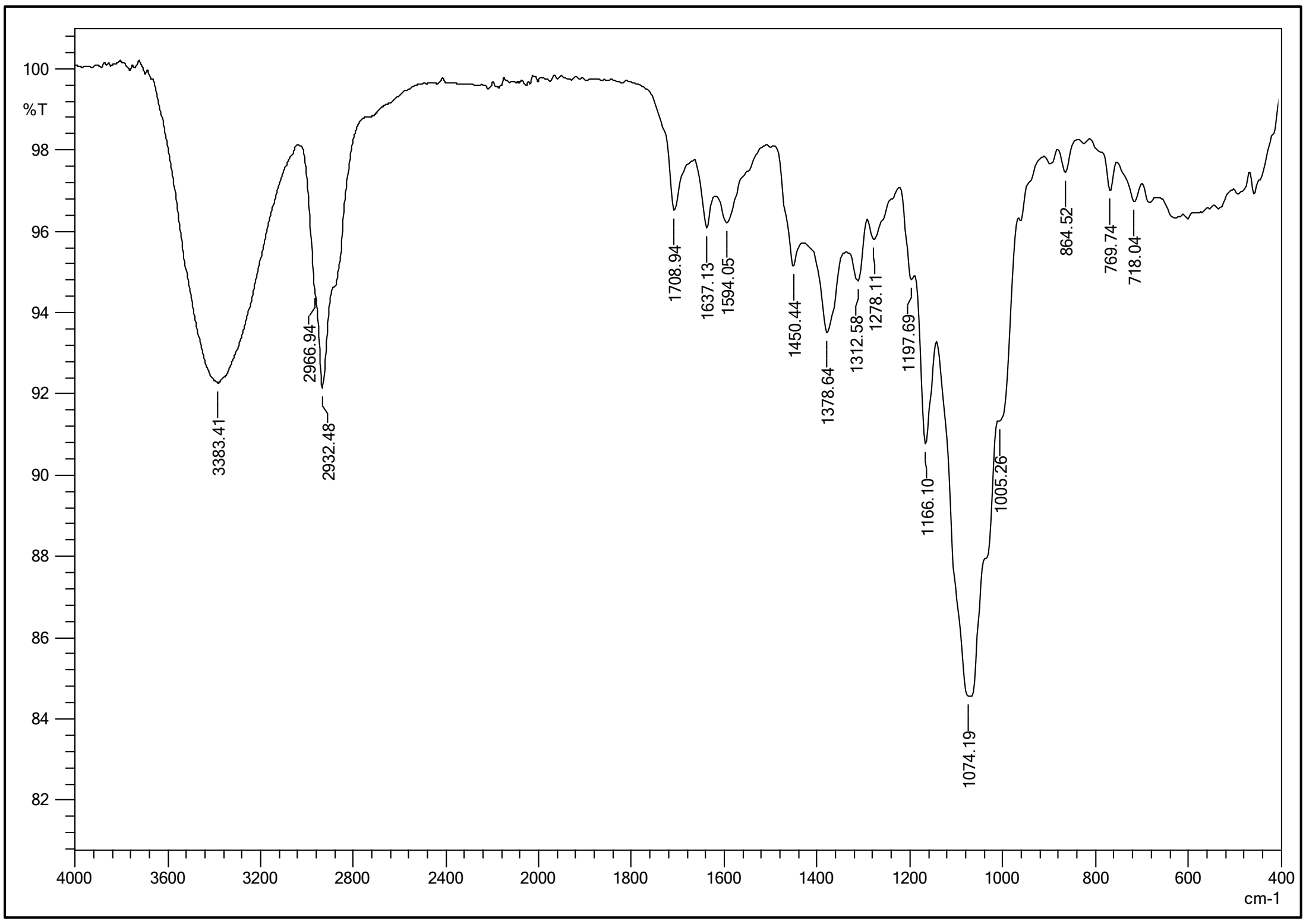

Figure S52. HR-ESI-MS mass spectrum of compound 6

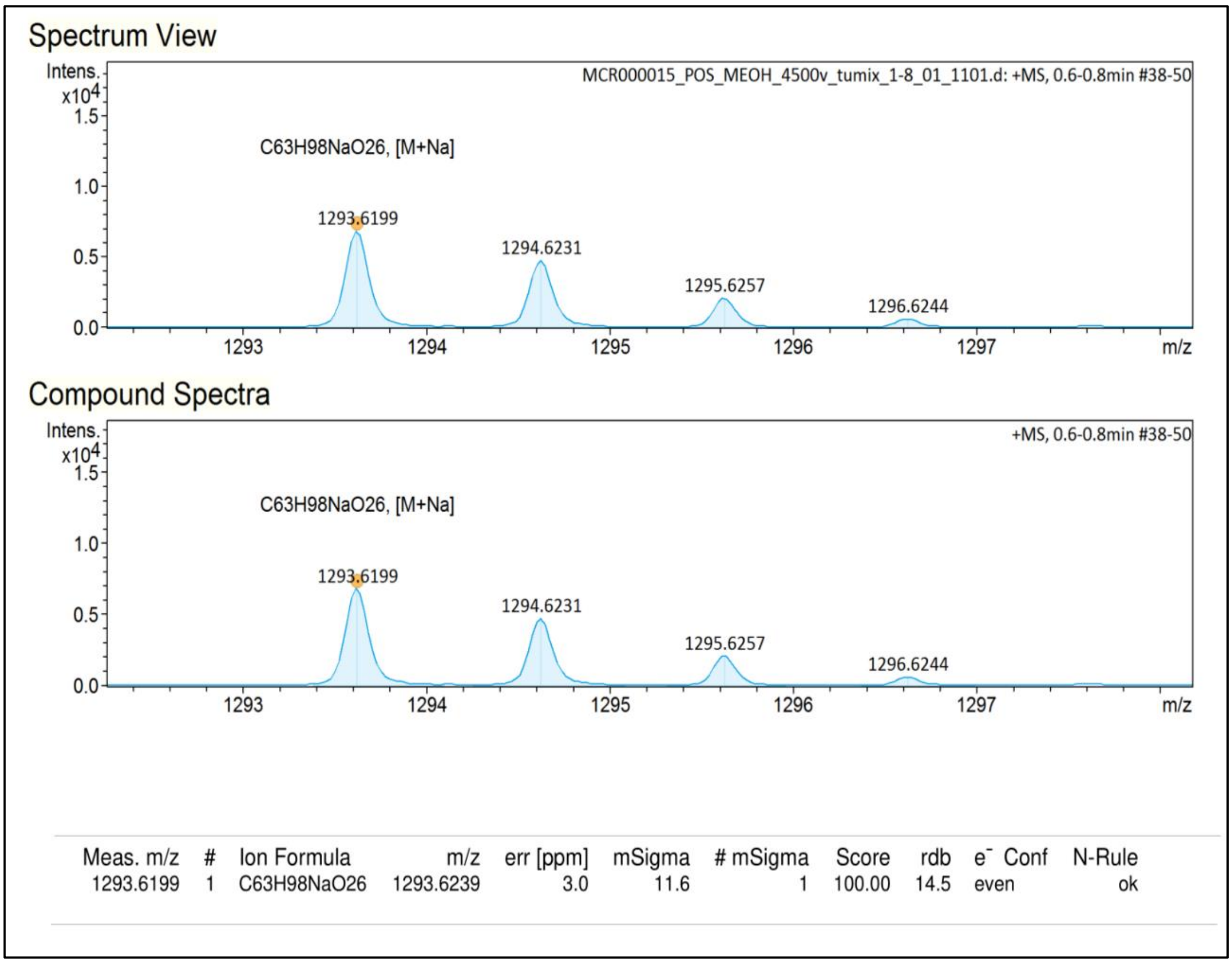

Figure S53. ^{1}H NMR spectrum (400 MHz, C_5D_5N) of compound 6

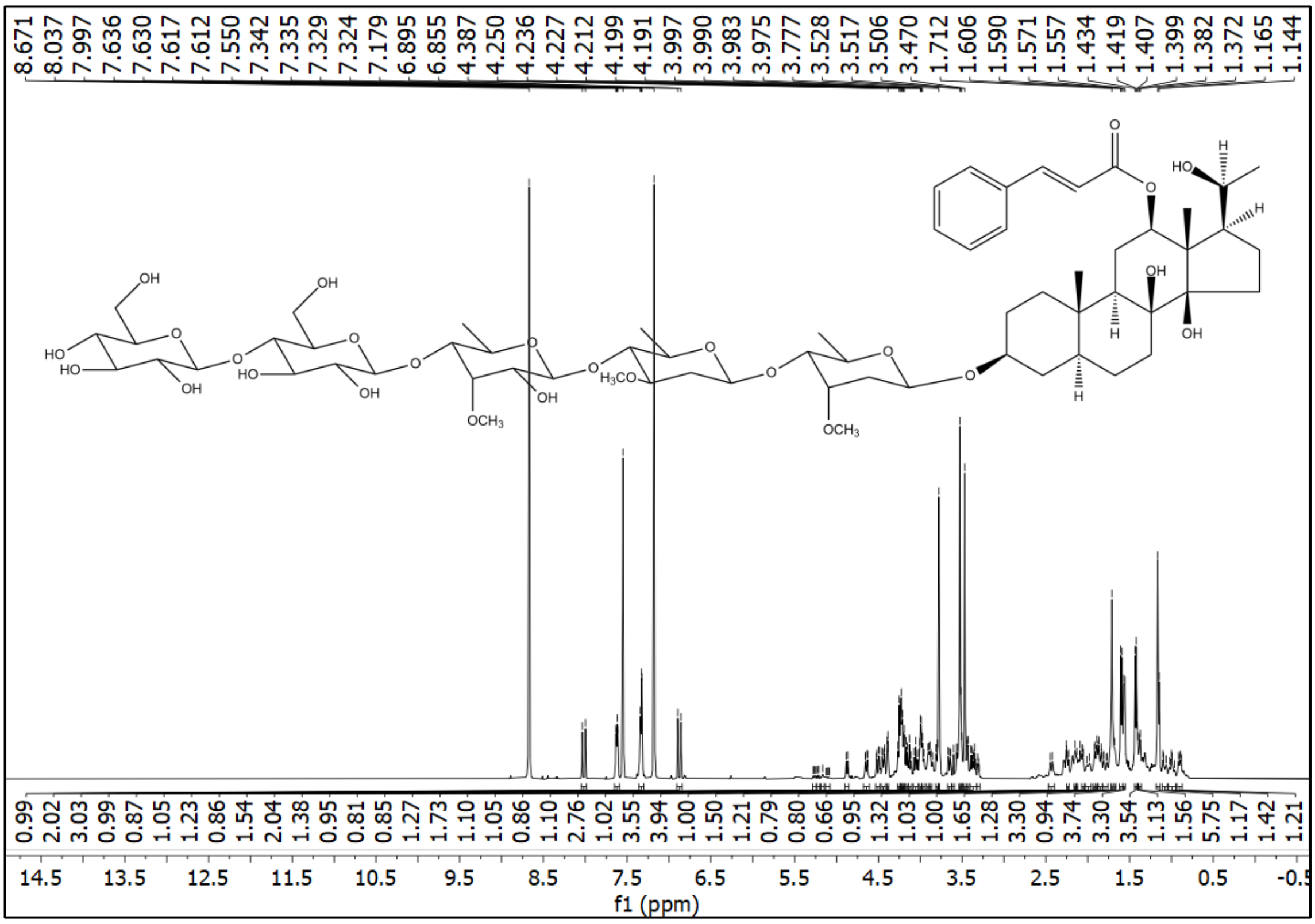

Figure S54. ^{13}C-BB NMR spectrum (100 MHz, C_5D_5N) of compound 6

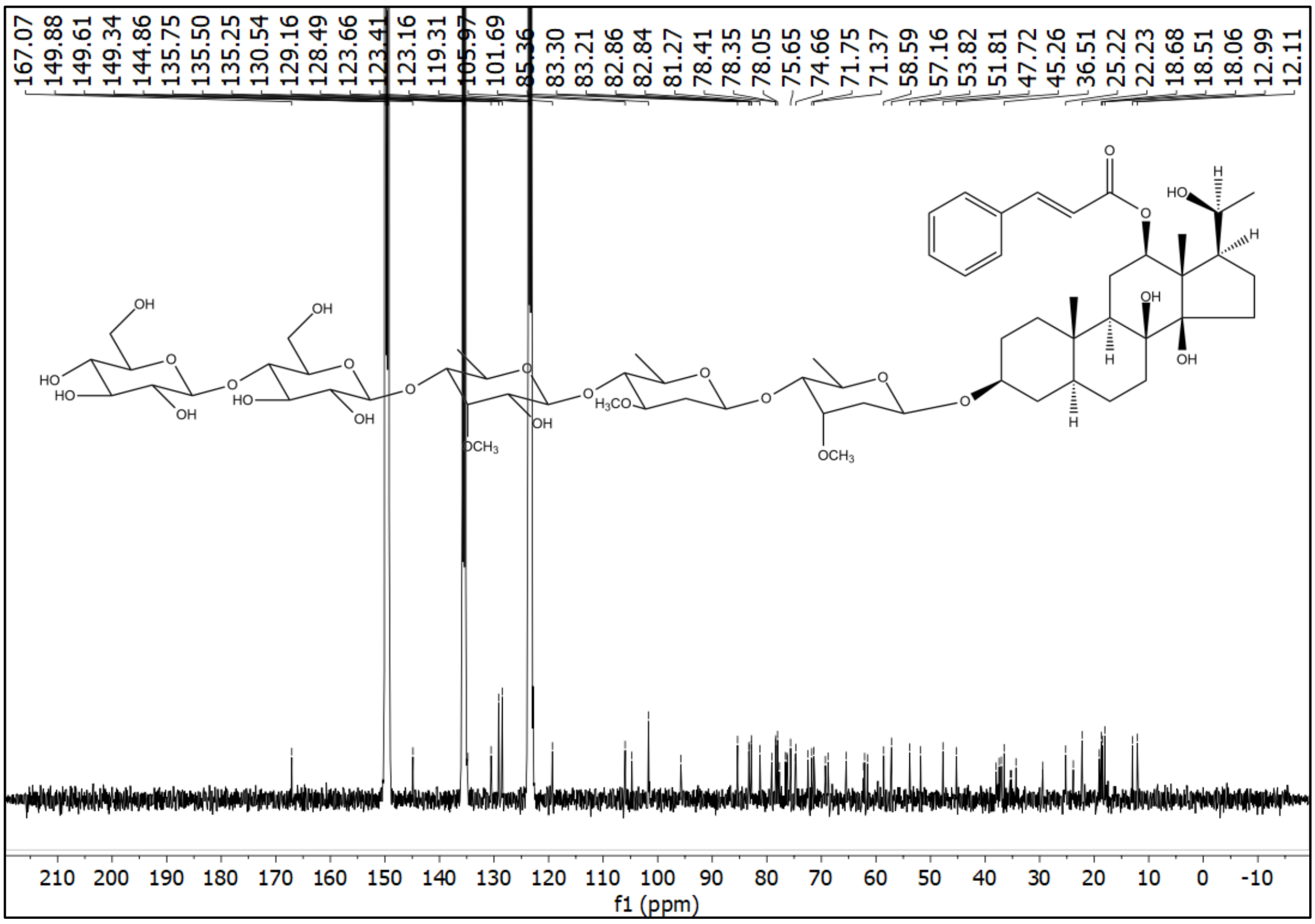

Figure S55. ^{13}C-DEPT135 NMR spectrum (100 MHz, C_5D_5N) of compound 6

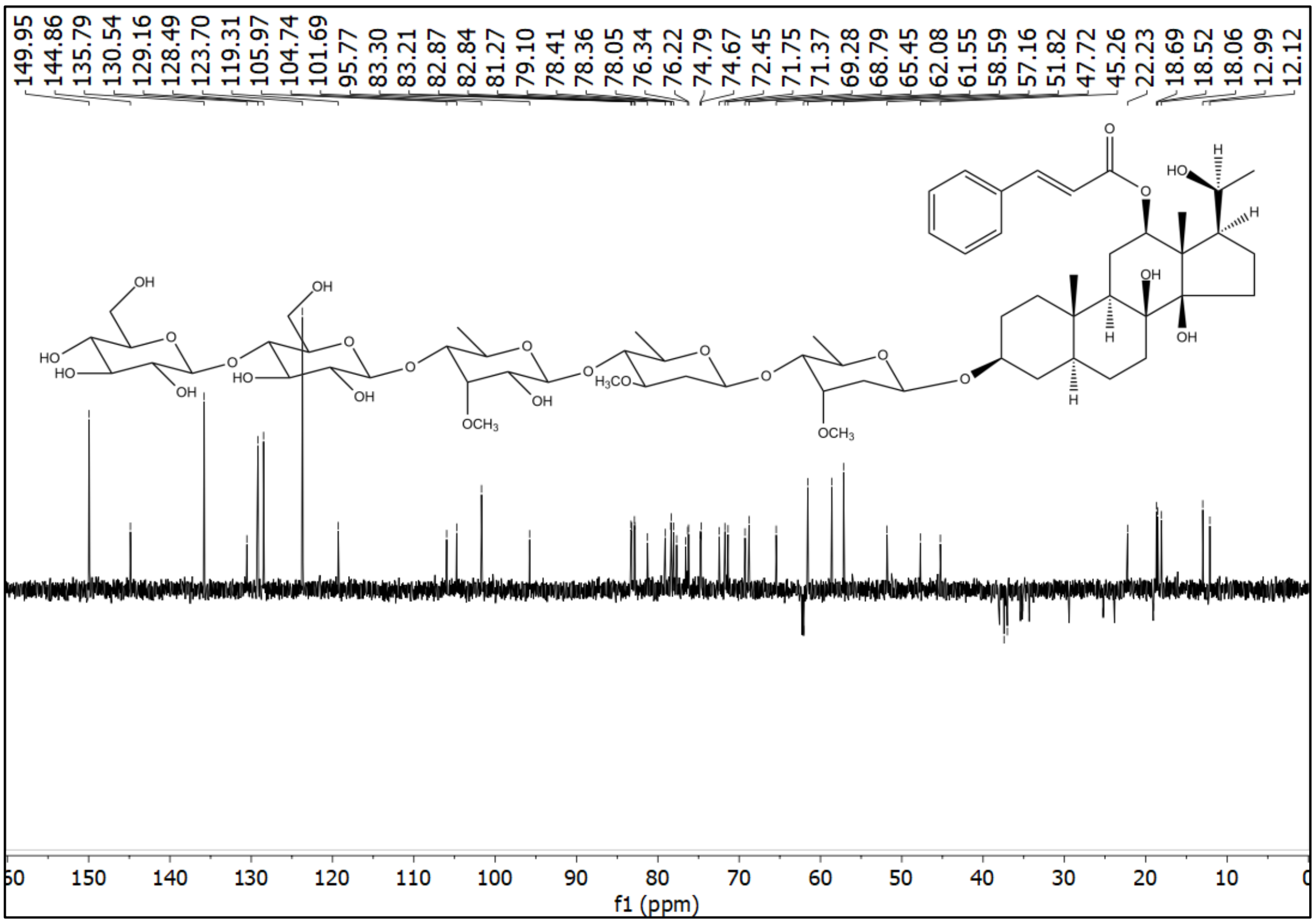

Figure S56. HSQC spectrum (400 x 100 MHz, C_5D_5N) of compound 6

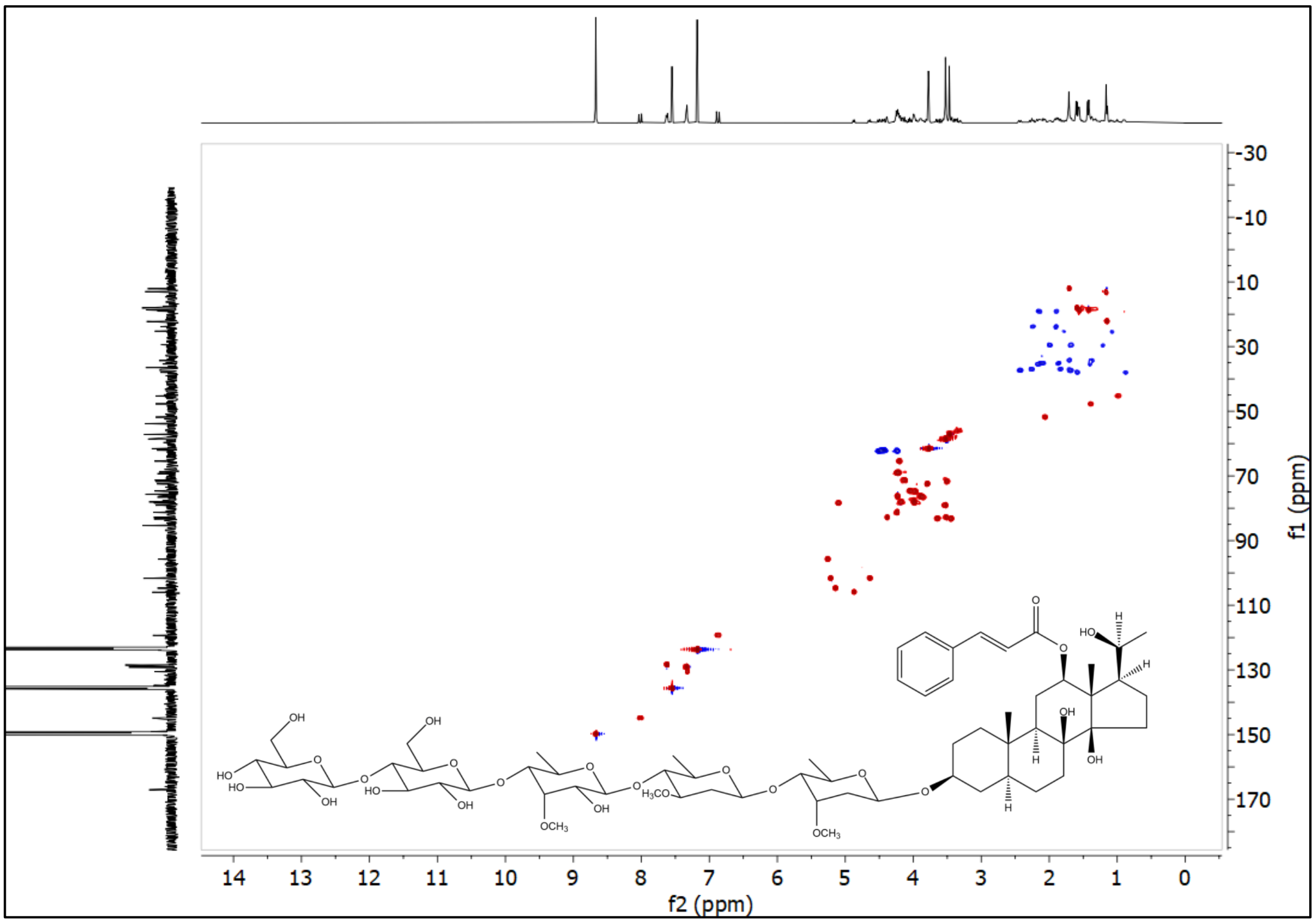

Figure S57. HMBC spectrum (400 x 100 MHz, C_5D_5N) of compound 6

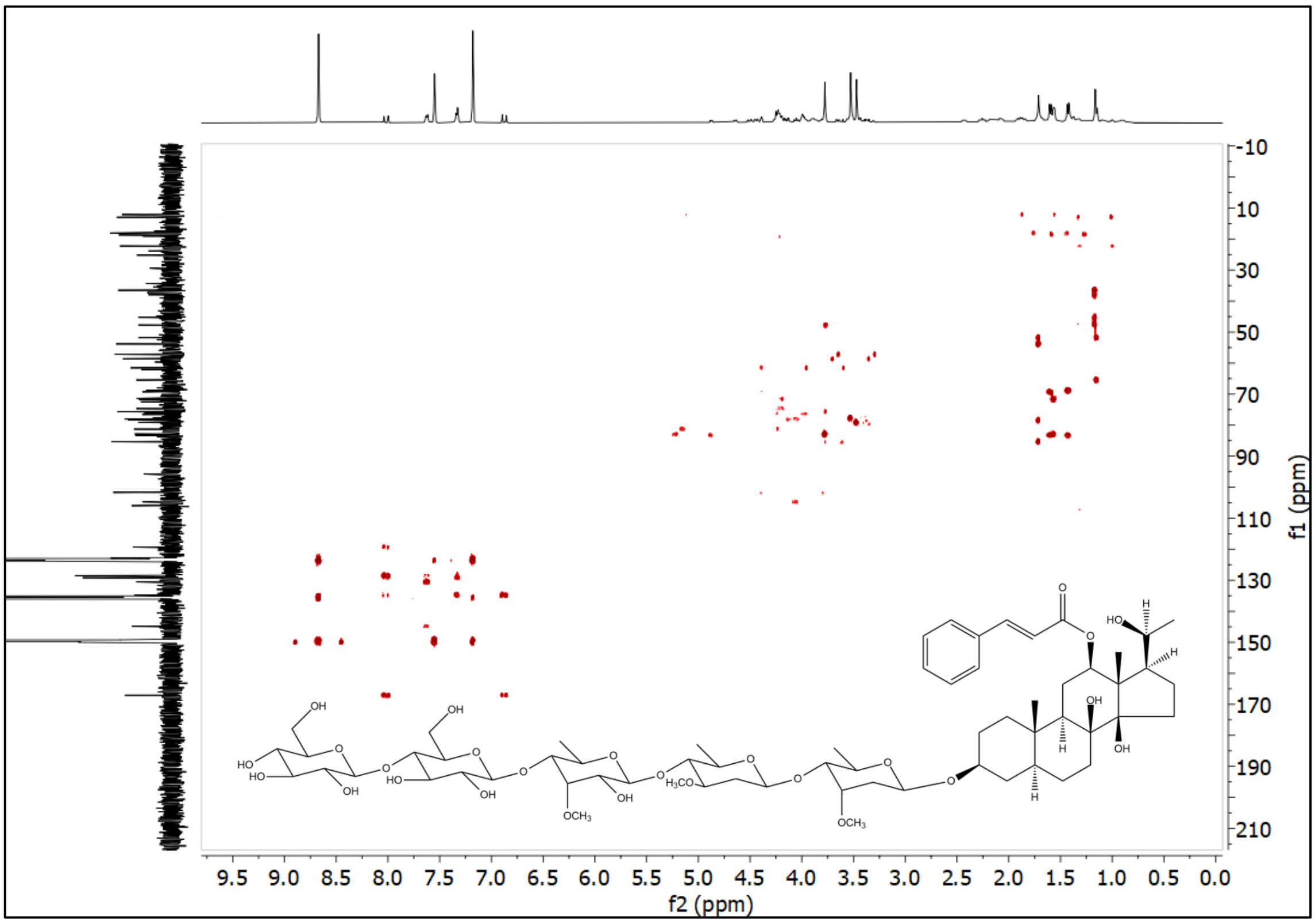

Figure S58. COSY spectrum (400 x 400 MHz, C_5D_5N) of compound 6

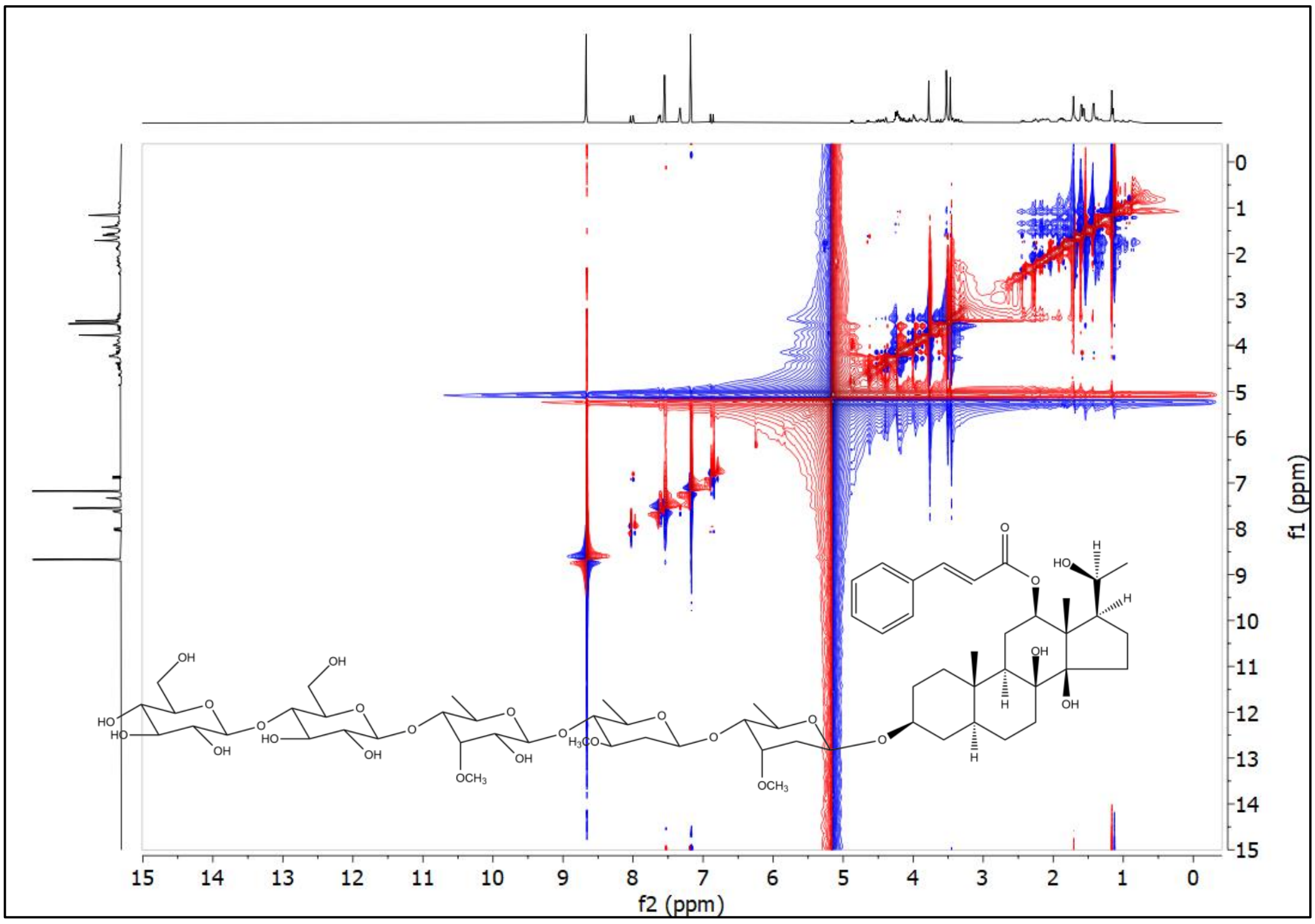

Figure S59. TOCSY spectrum (400 x 400 MHz, C_5D_5N) of compound 6

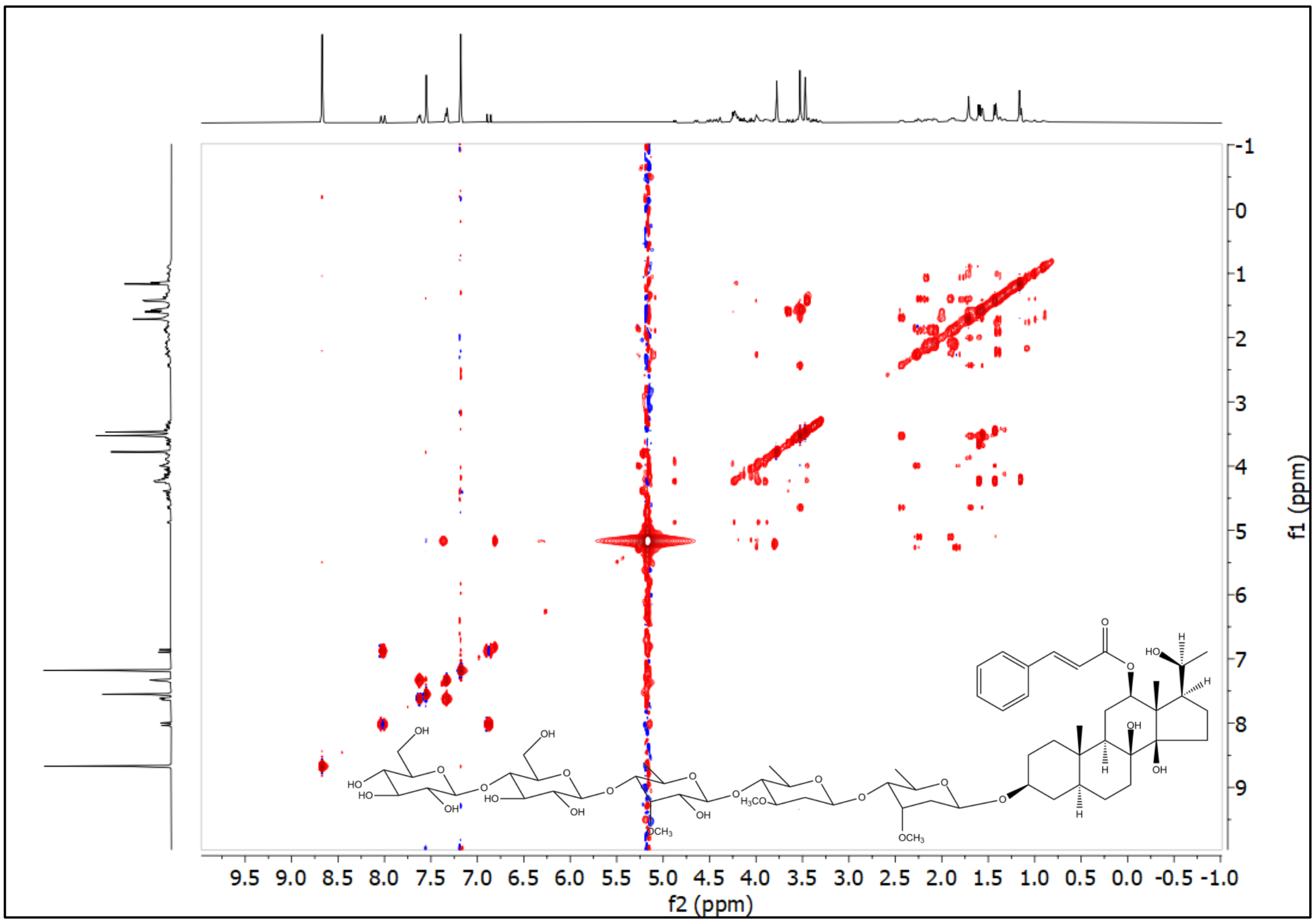

Figure S60. ROESY spectrum (400 x 400 MHz, C_5D_5N) of compound 6

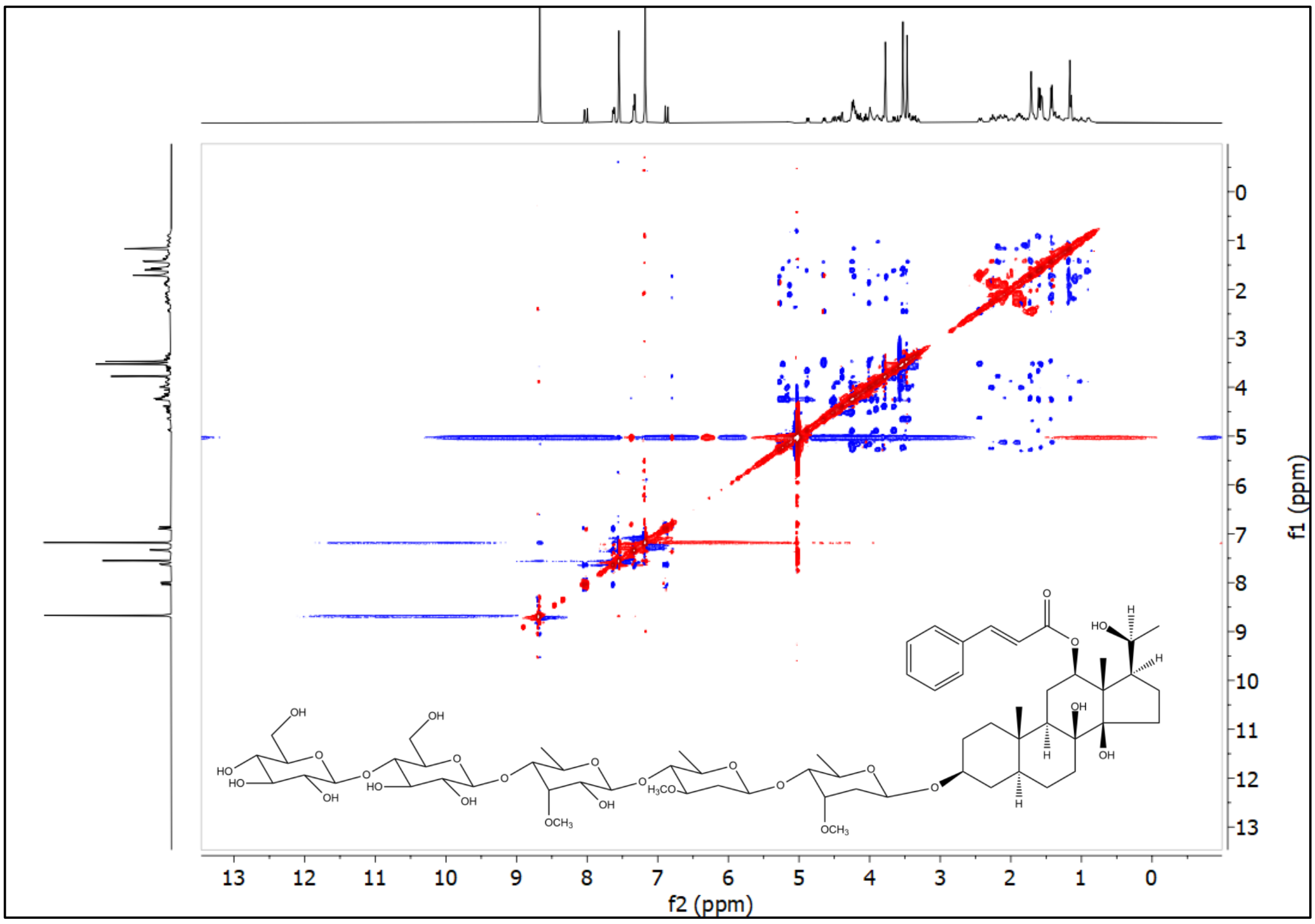

Figure S61. ATR-Infrared spectrum of compound 7.

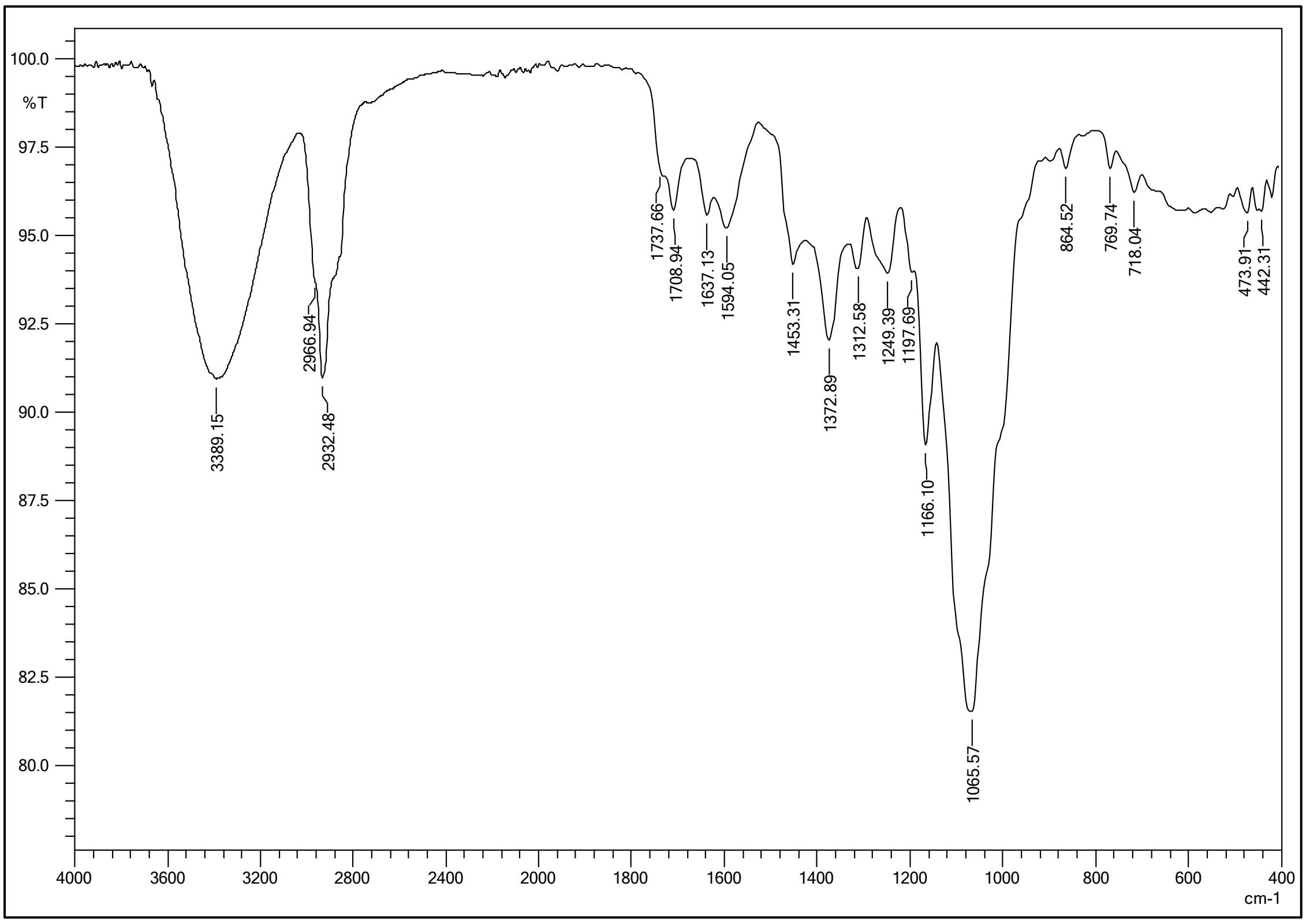

Figure S62. HR-ESI-MS mass spectrum of compound 7.

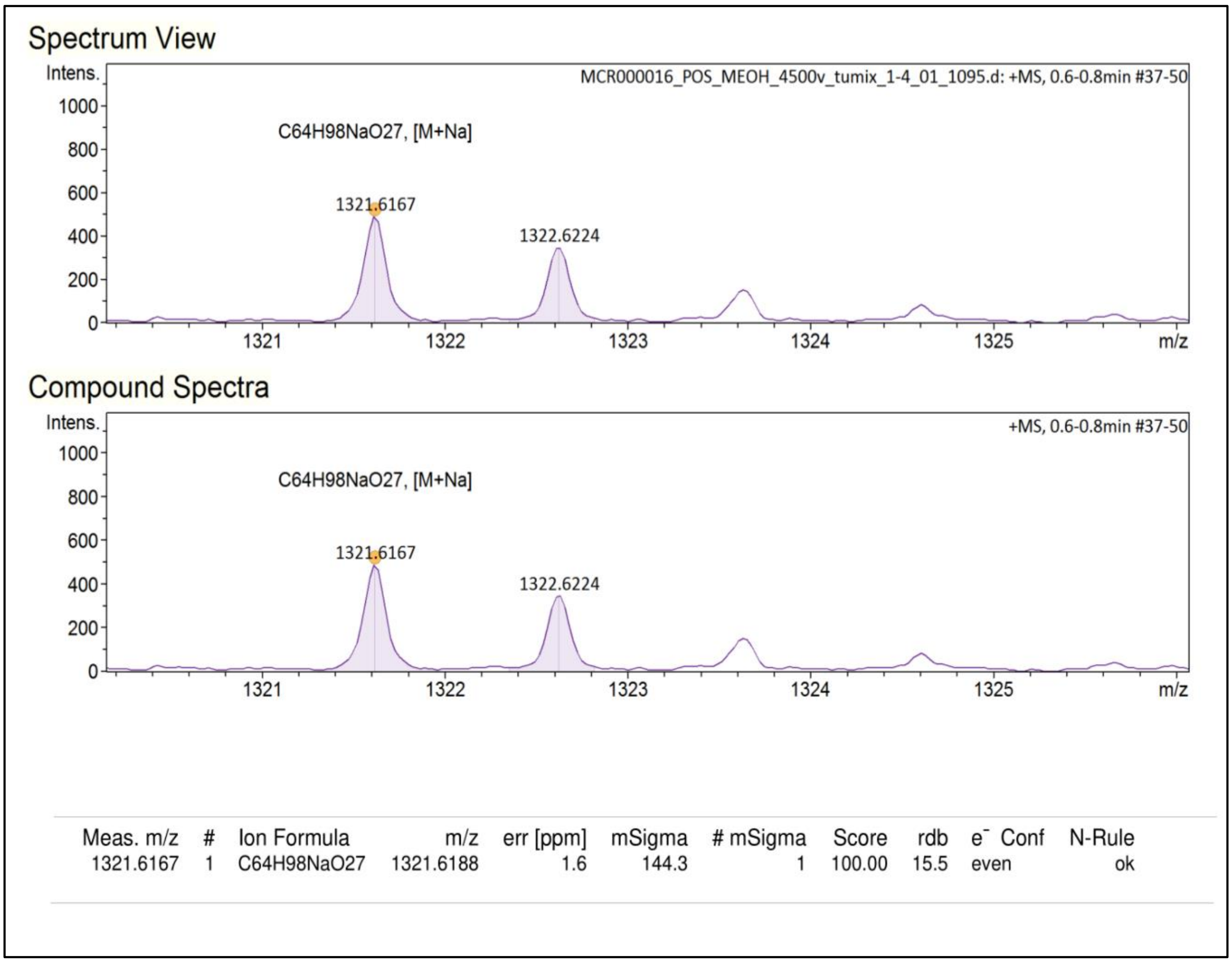

Figure S63. ^{1}H NMR spectrum (400 MHz, C_5D_5N) of compound 7

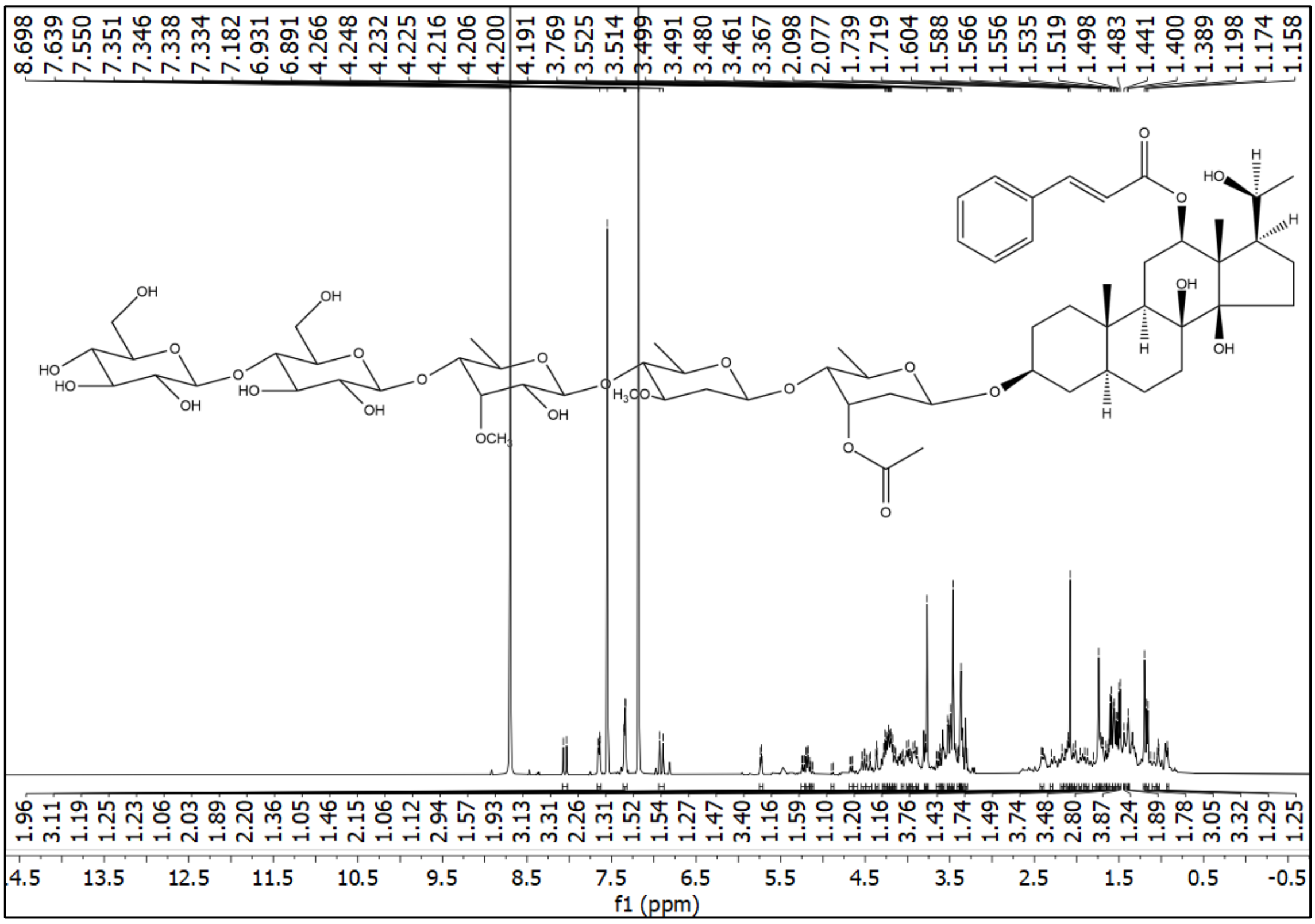

Figure S64. ^{13}C-BB NMR spectrum (100 MHz, C_5D_5N) of compound 7

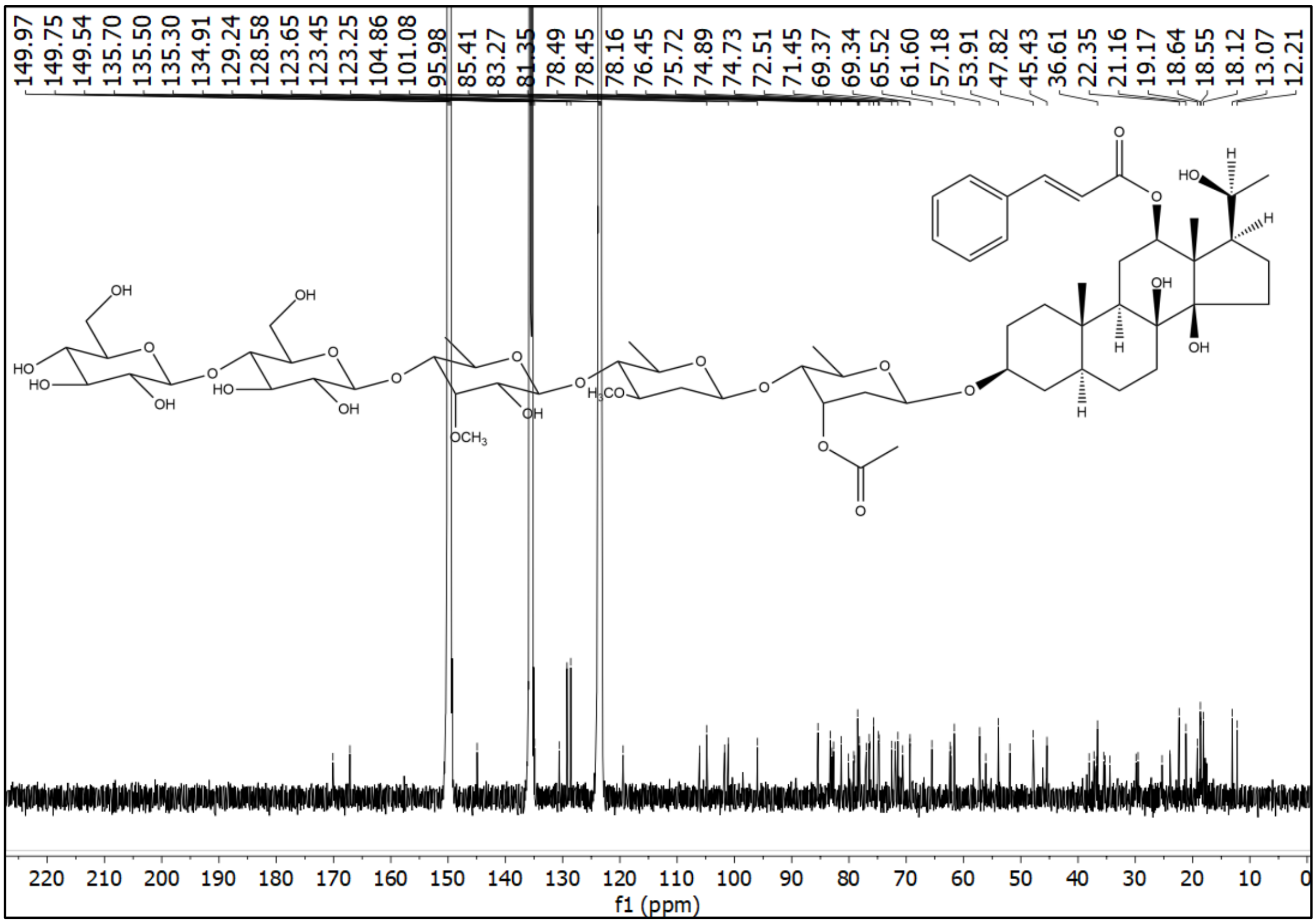

Figure S65. ^{13}C-DEPT135 NMR spectrum (100 MHz, C_5D_5N) of compound 7

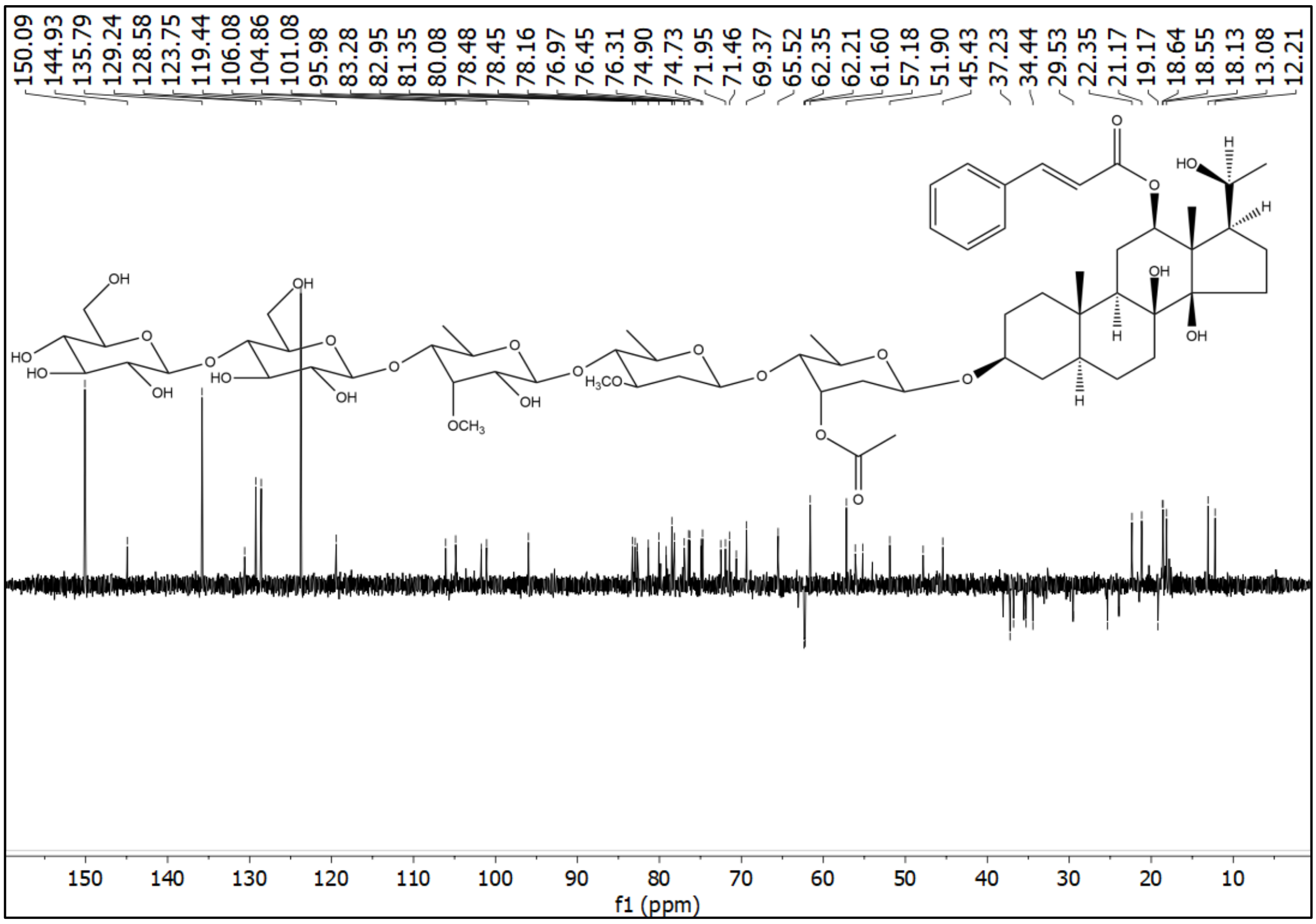

Figure S66. HSQC spectrum (400 x 100 MHz, C_5D_5N) of compound 7

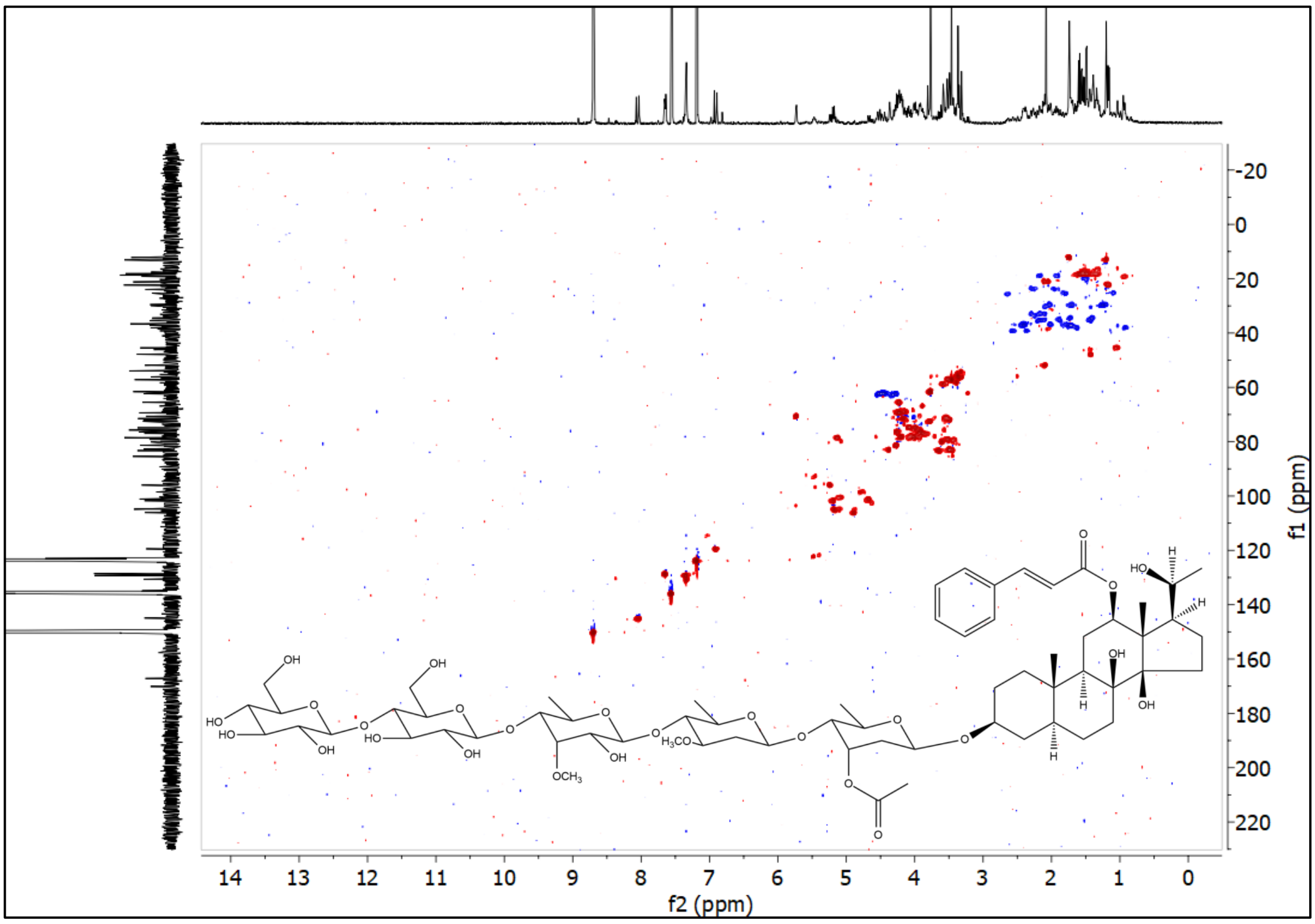

Figure S67. HMBC spectrum (400 x 100 MHz, C_5D_5N) of compound 7

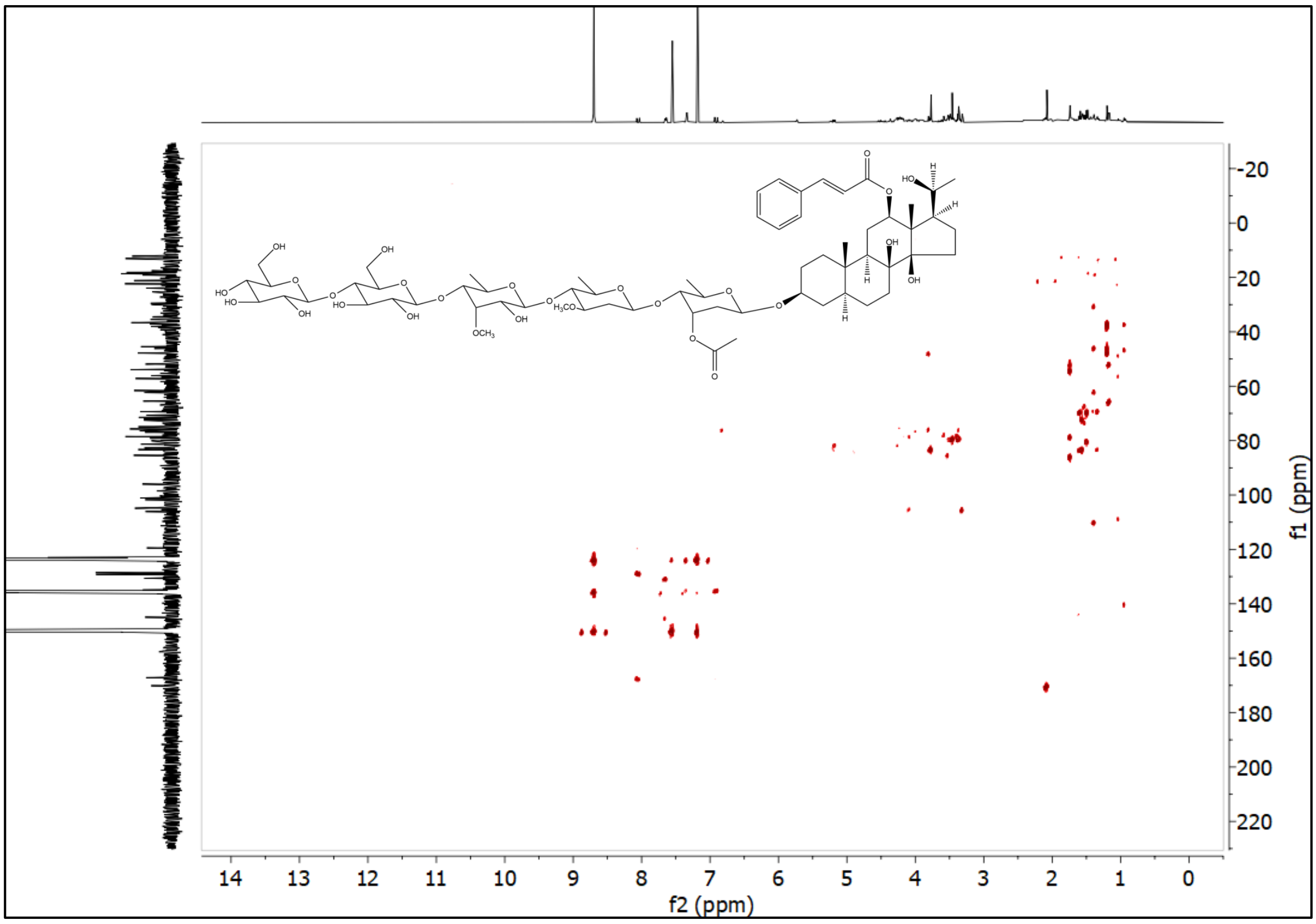

Figure S68. COSY spectrum (400 x 400 MHz, C_5D_5N) of compound 7.

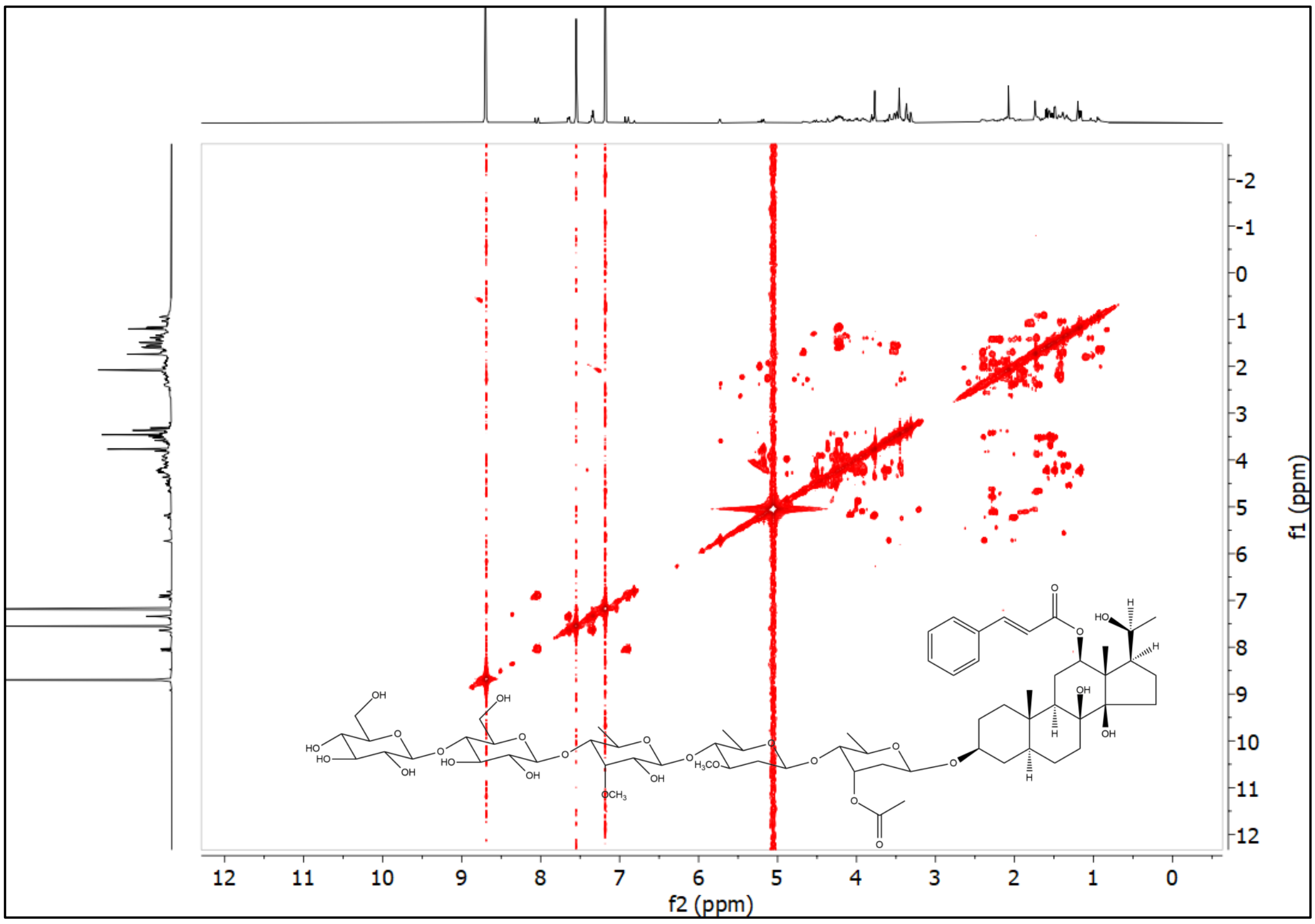

Figure S69. TOCSY spectrum (400 x 400 MHz, C_5D_5N) of compound 7.

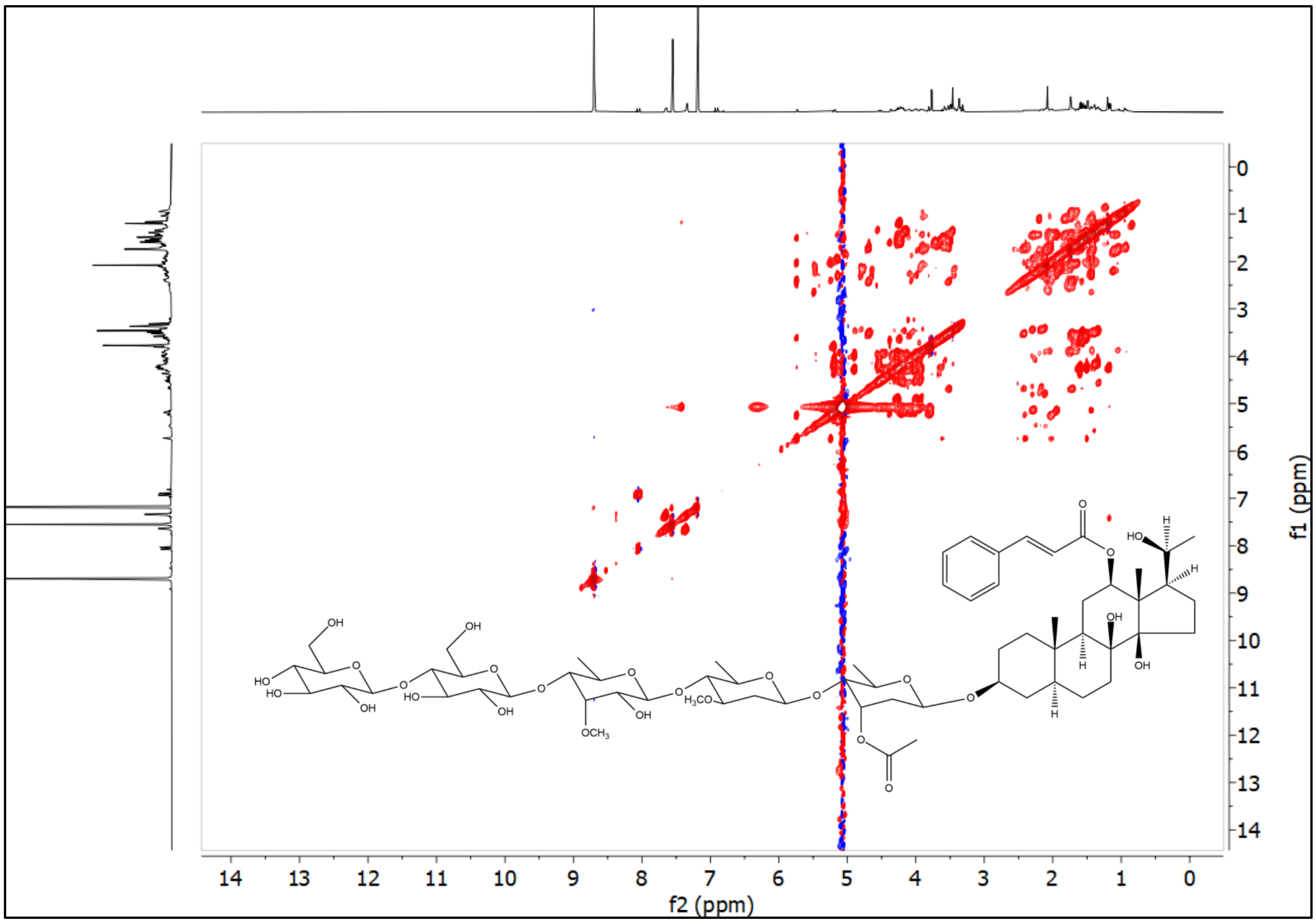

Figure S70. NOESY spectrum (400 x 400 MHz, C_5D_5N) of compound 7.

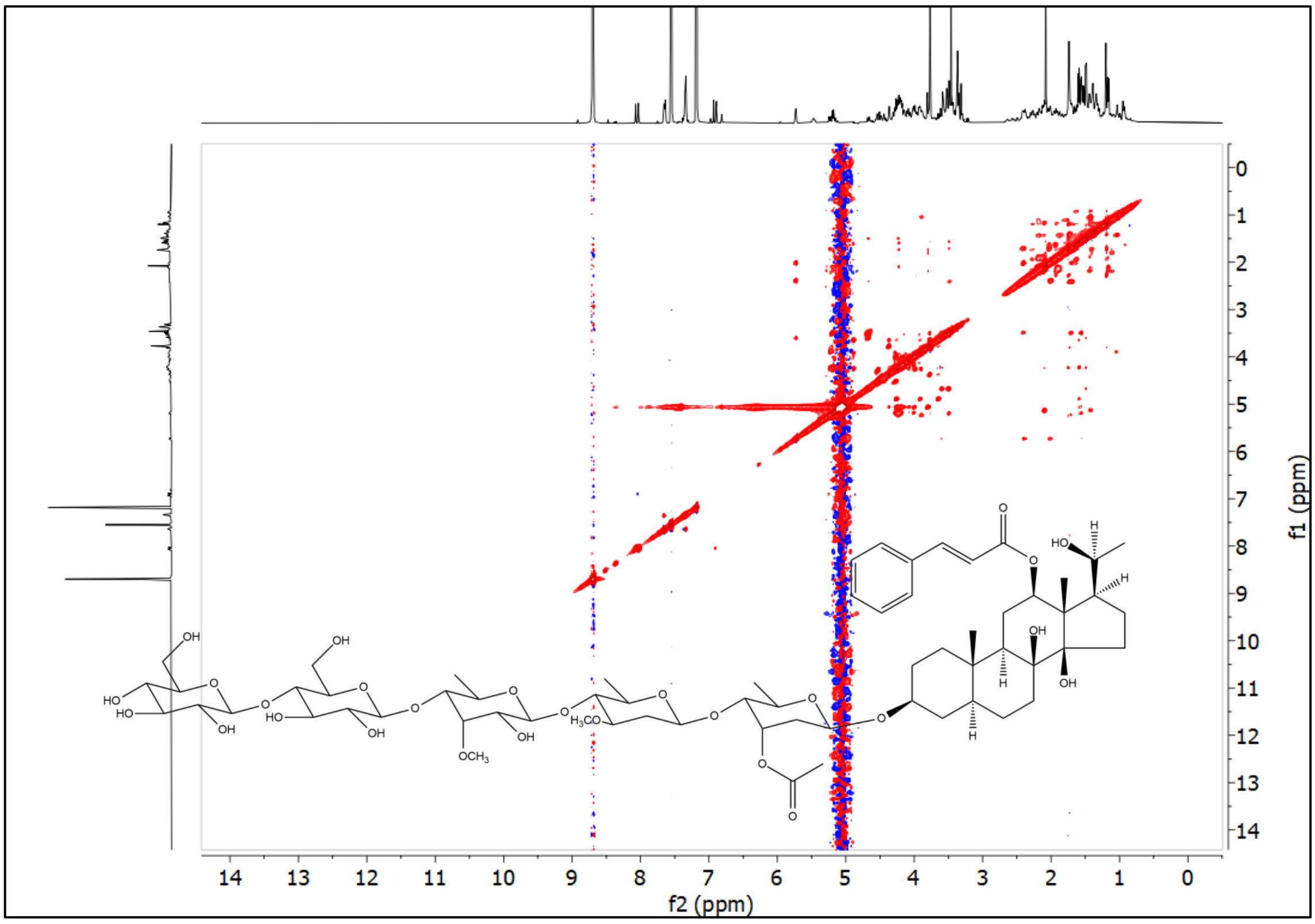

Figure S71. ATR-Infrared spectrum of compound 8.

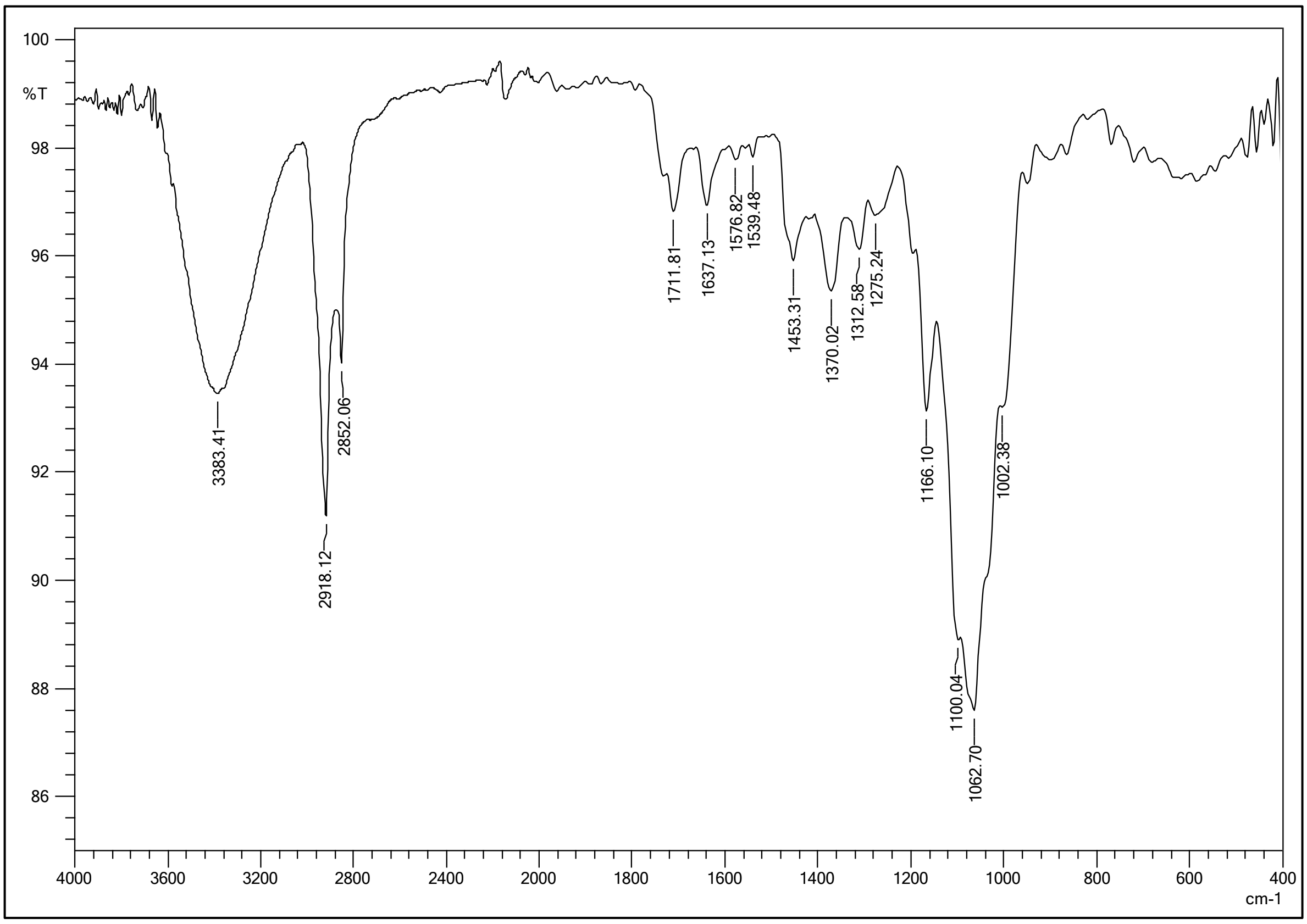

Figure S72. HR-ESI-MS mass spectrum of compound 8.

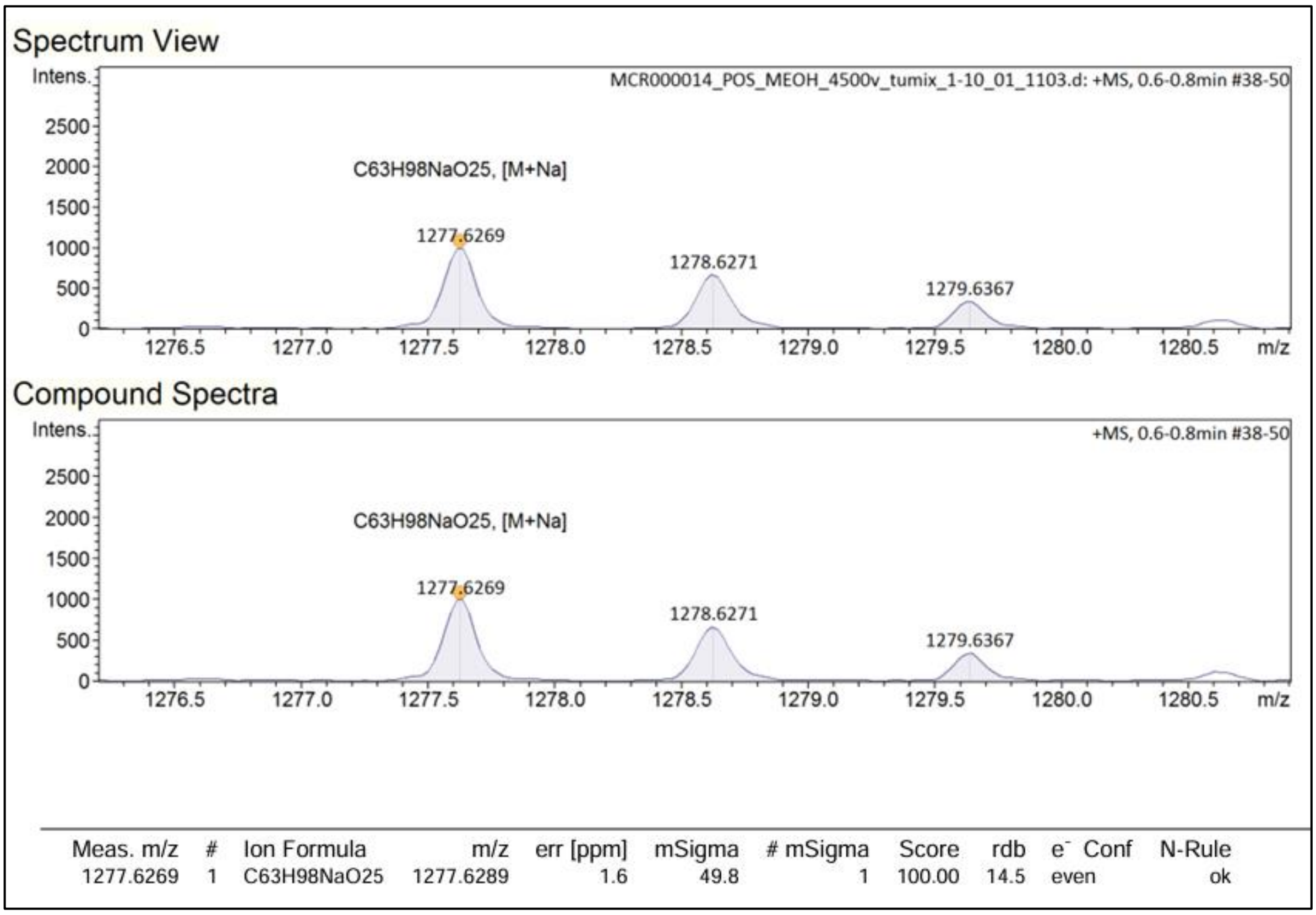

Figure S73. ^{1}H NMR spectrum (400 MHz, C_5D_5N) of compound 8

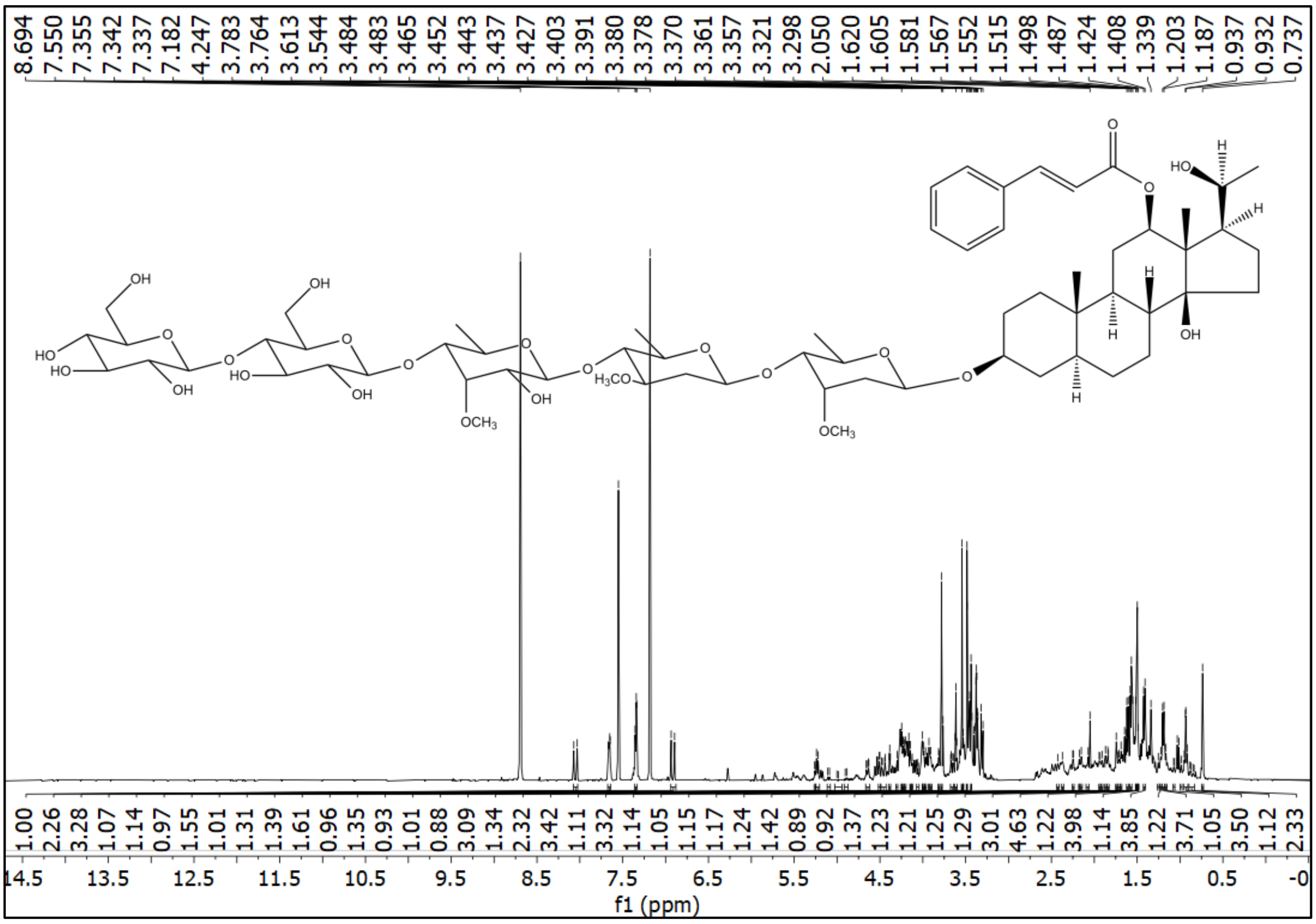

Figure S74. ^{13}C-BB NMR spectrum (100 MHz, C_5D_5N) of compound 8

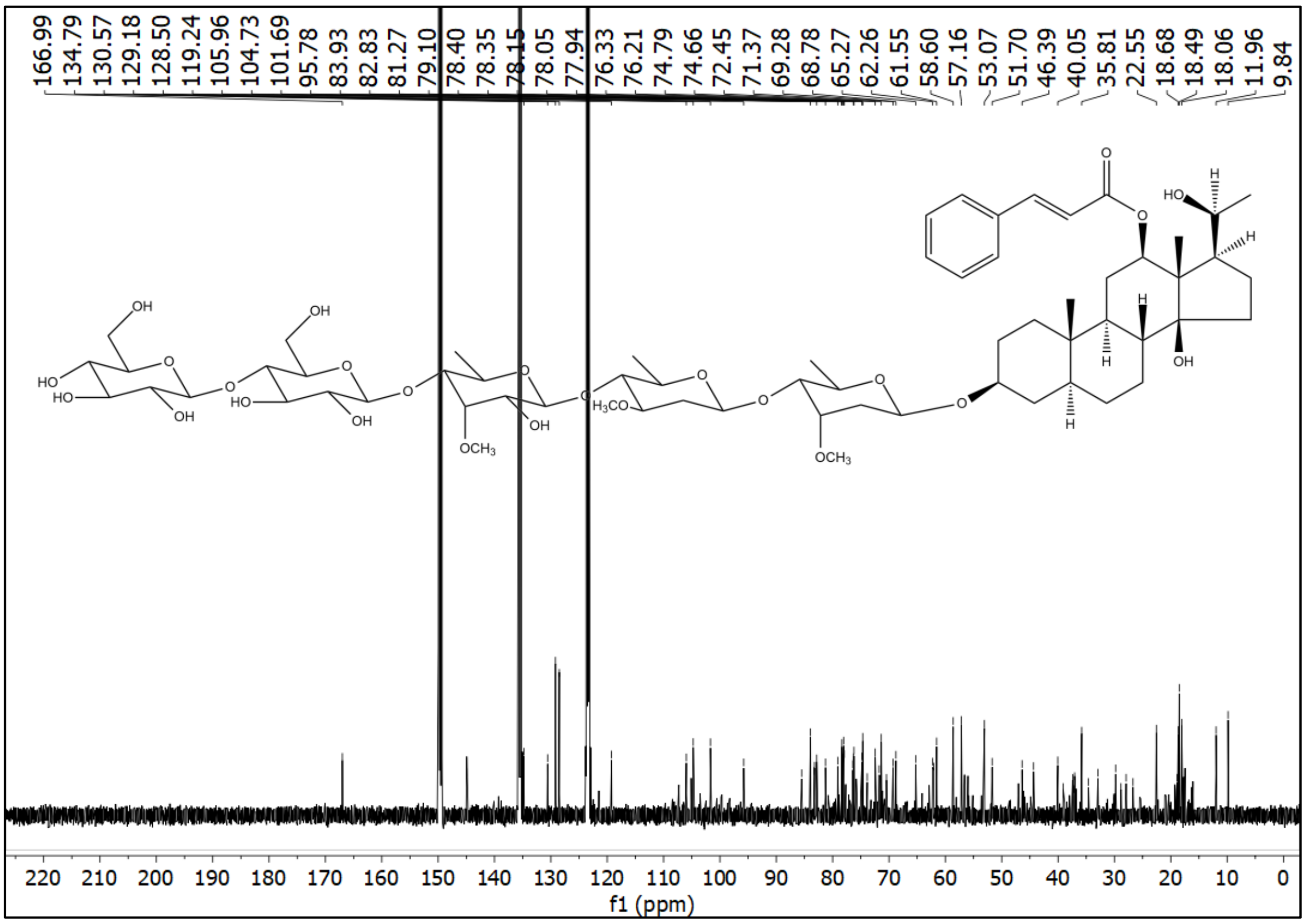

Figure S75. ^{13}C-DEPT135 NMR spectrum (100 MHz, C_5D_5N) of compound 8

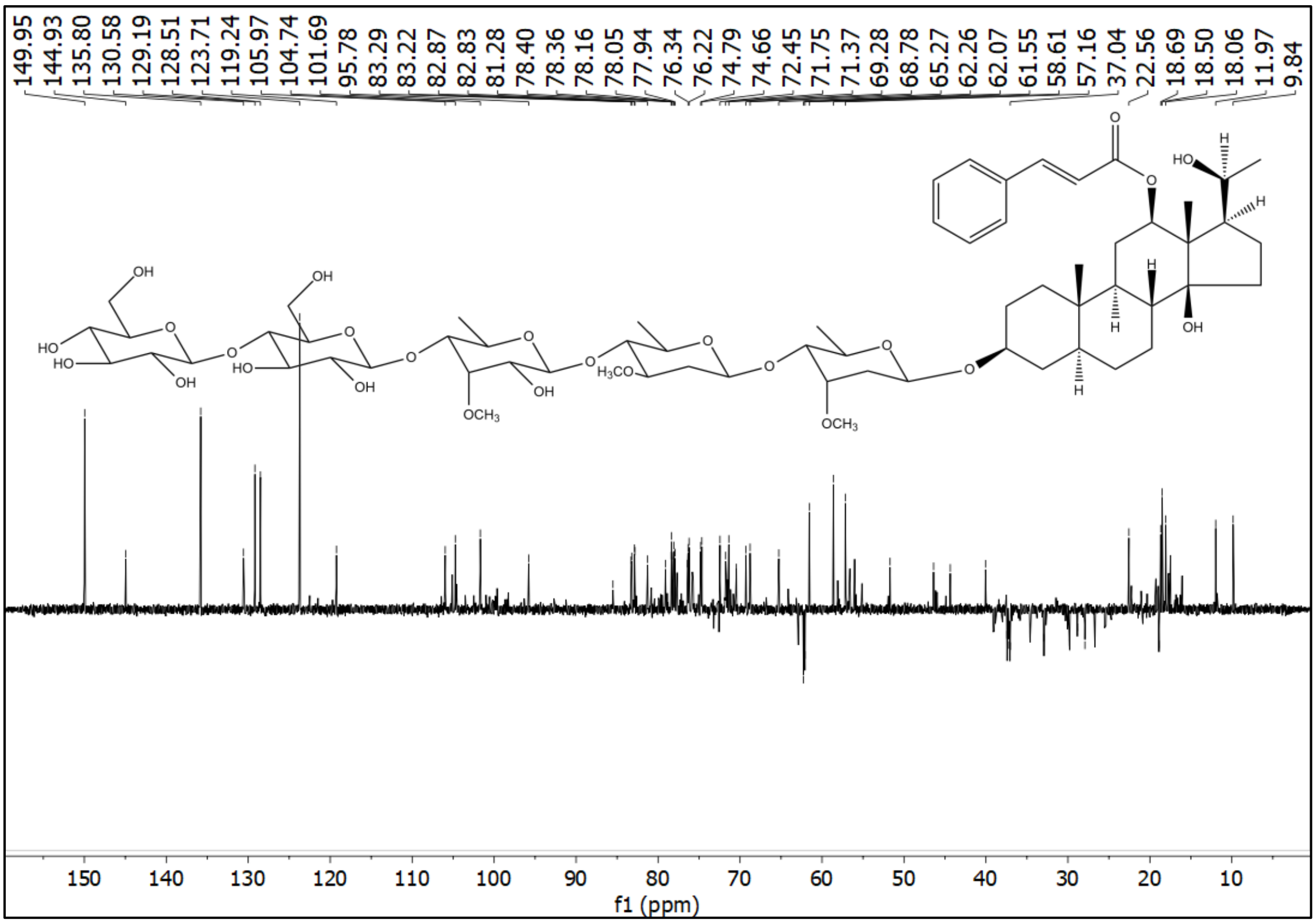

Figure S76. HSQC spectrum (400 x 100 MHz, C_5D_5N) of compound 8

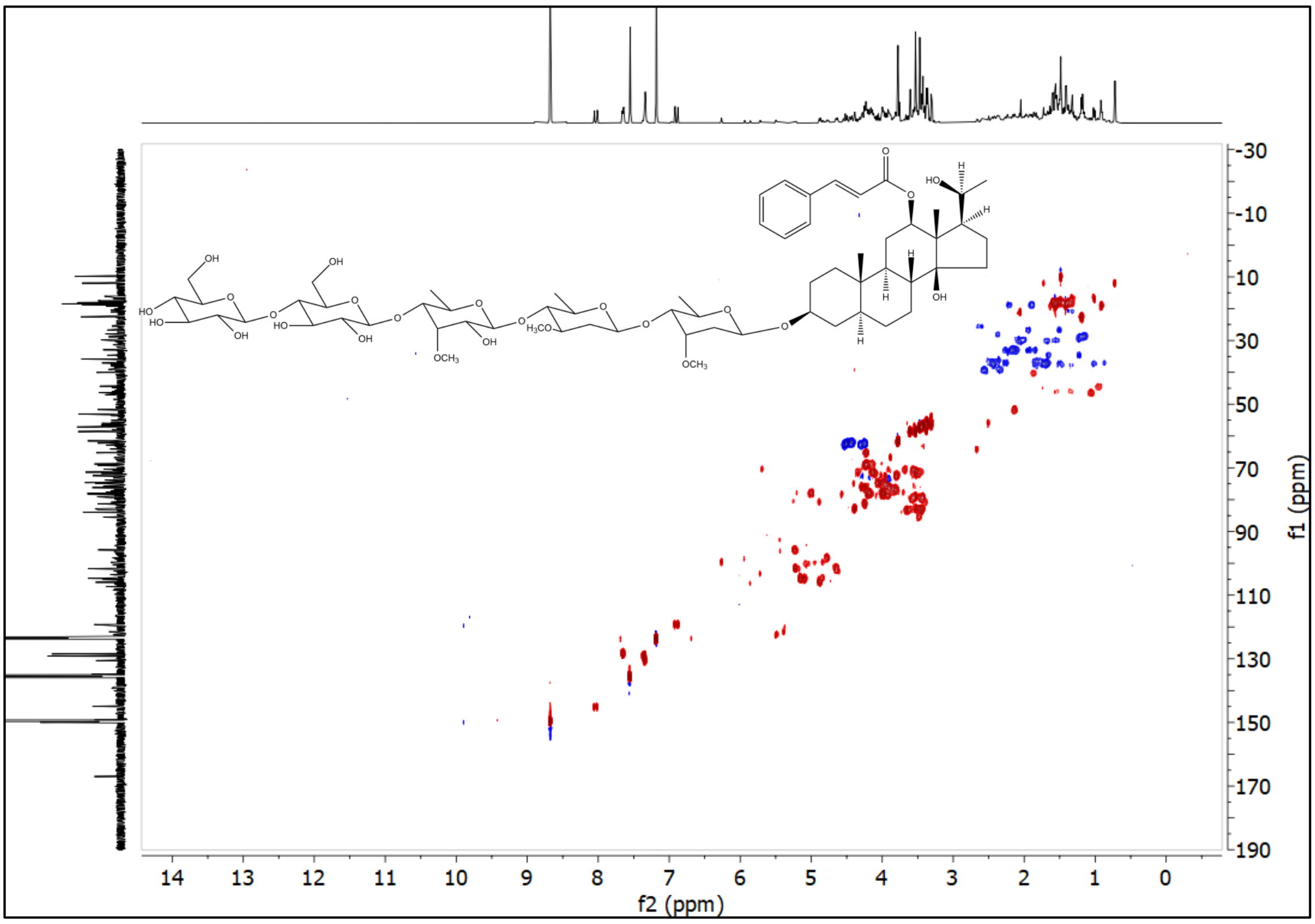

Figure S77. HMBC spectrum (400 x 100 MHz, C_5D_5N) of compound 8

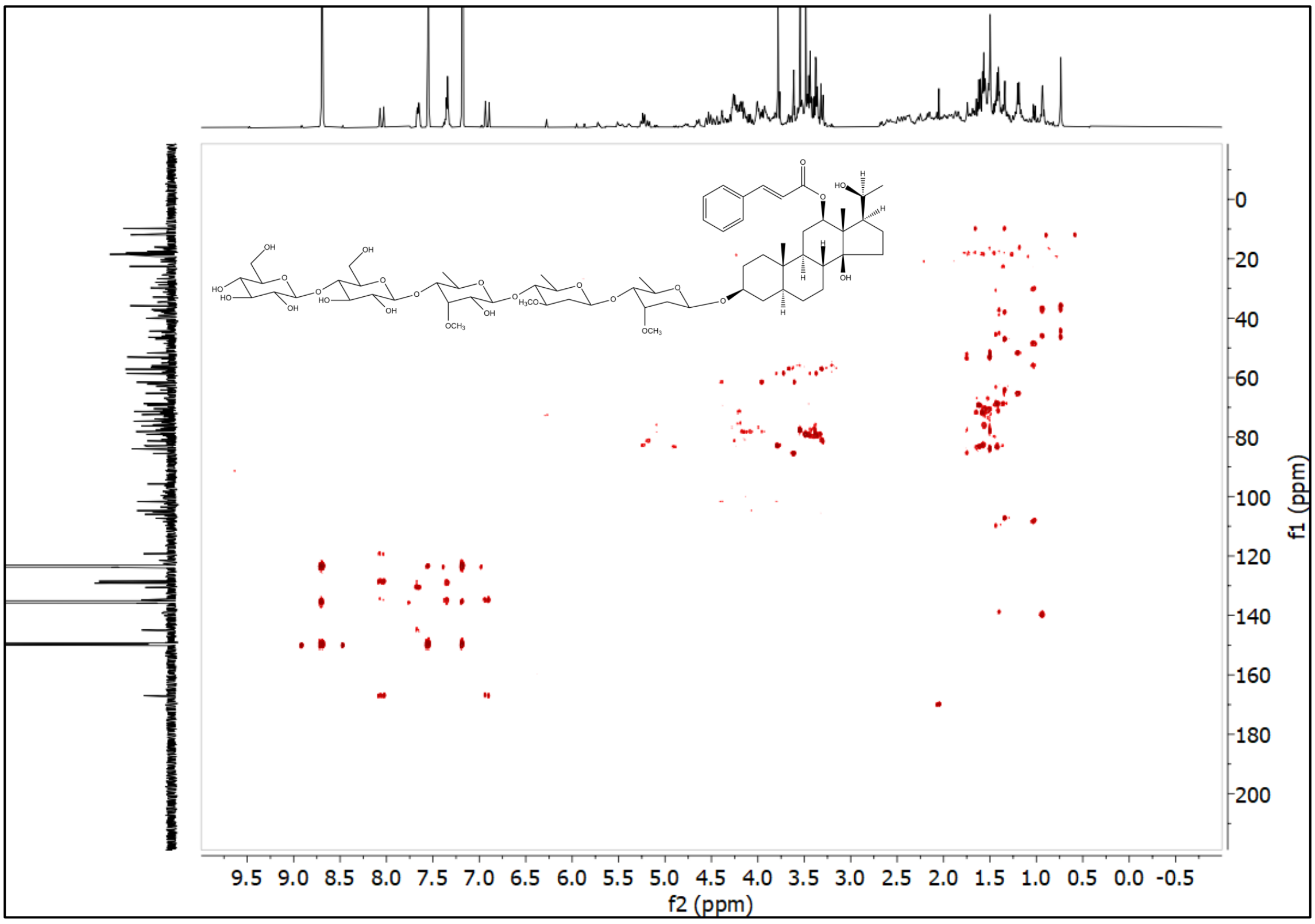

Figure S78. COSY spectrum (400 x 400 MHz, C_5D_5N) of compound 8

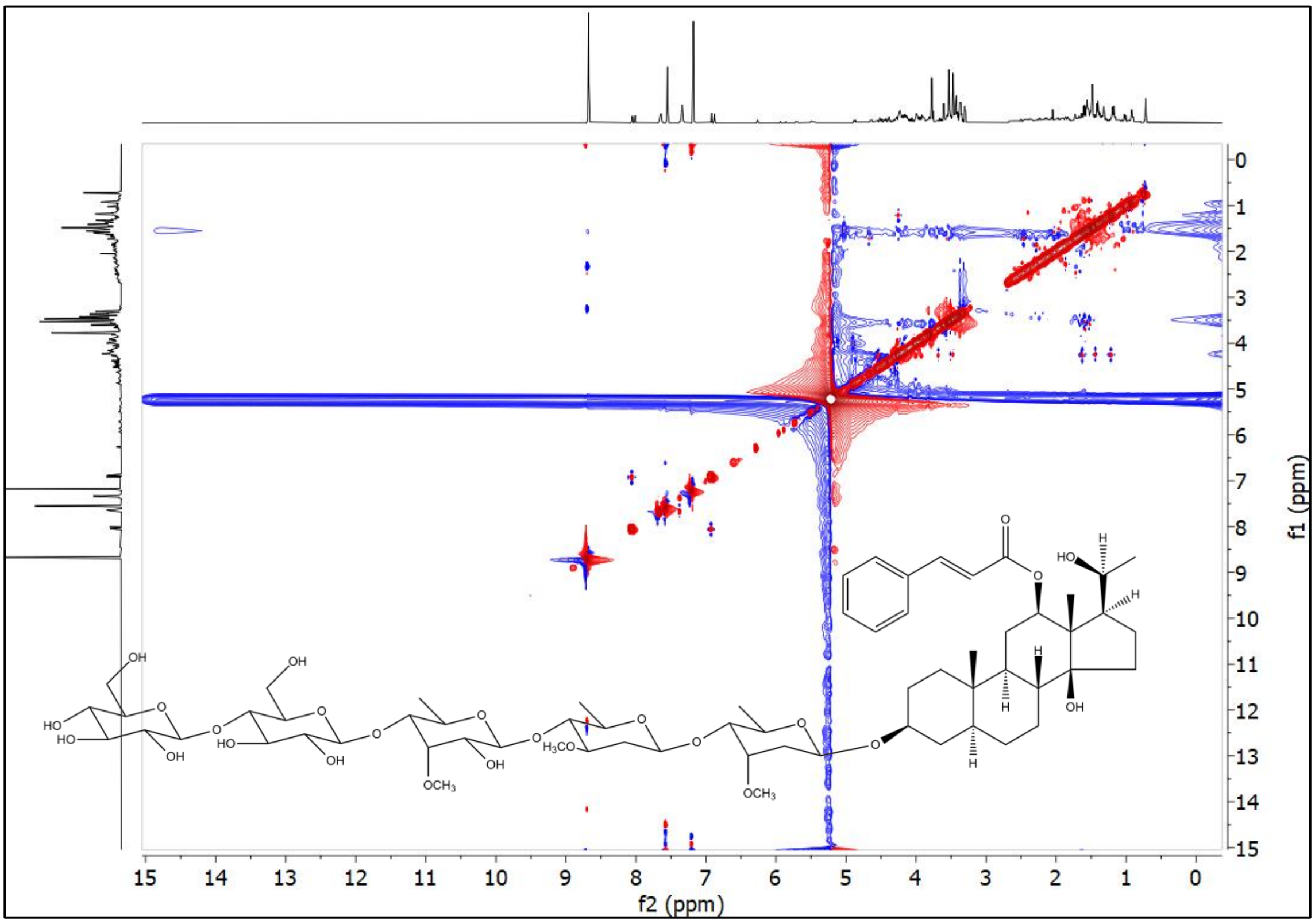

Figure S79. TOCSY spectrum (400 x 400 MHz, C_5D_5N) of compound 8

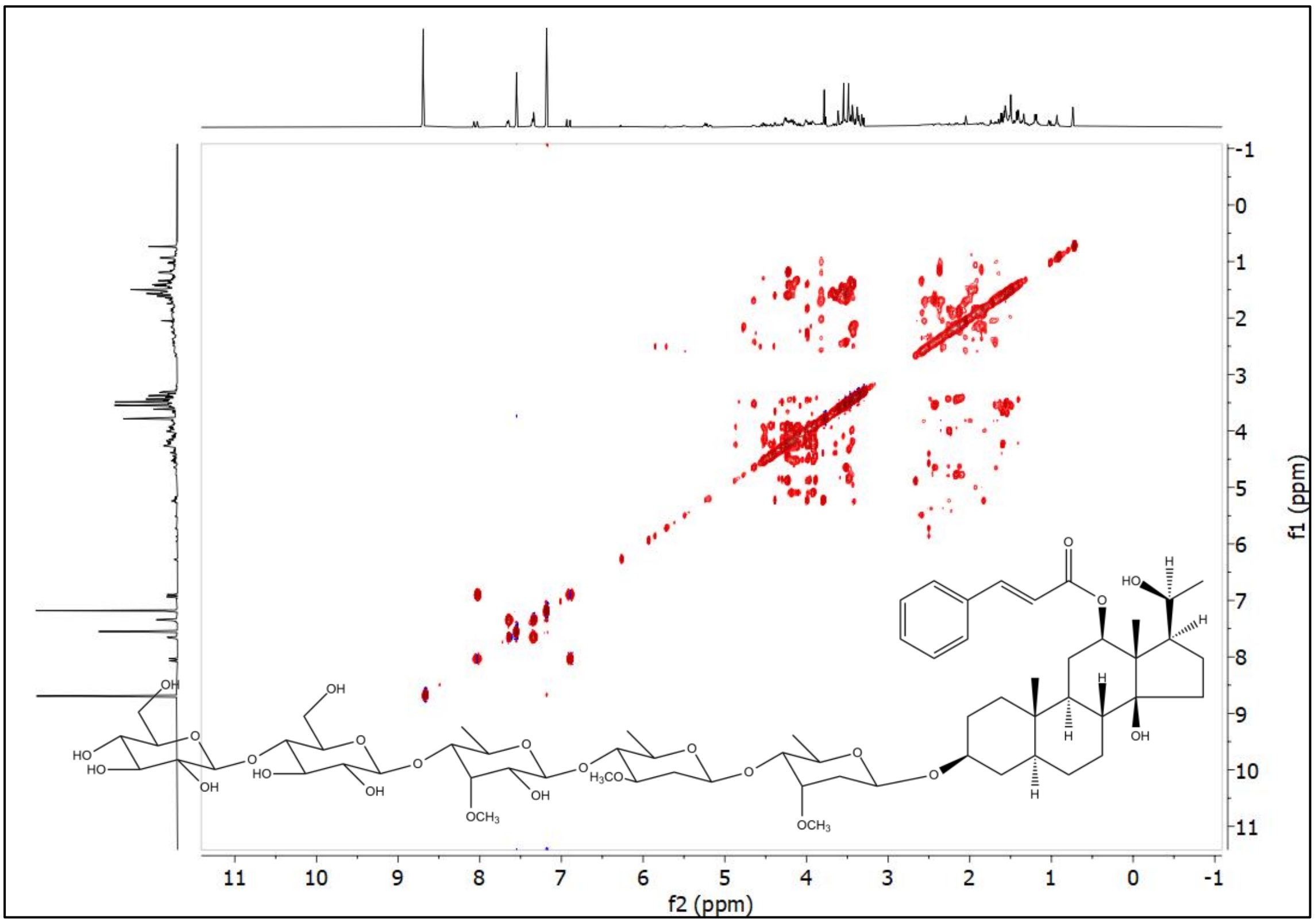

Figure S80. NOESY spectrum (400 x 400 MHz, C_5D_5N) of compound 8

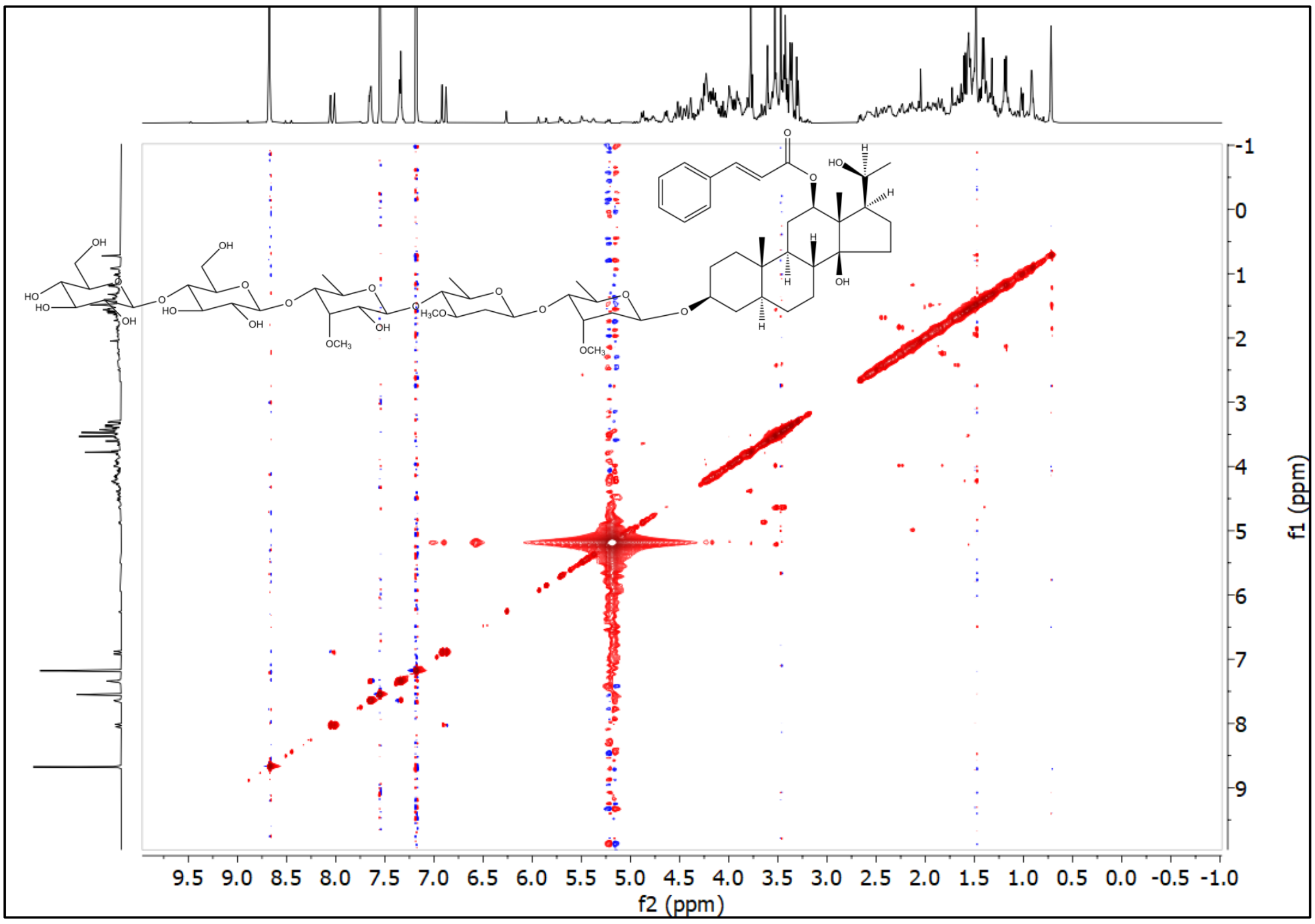

Figure S81. ATR-Infrared spectrum of compound 9.

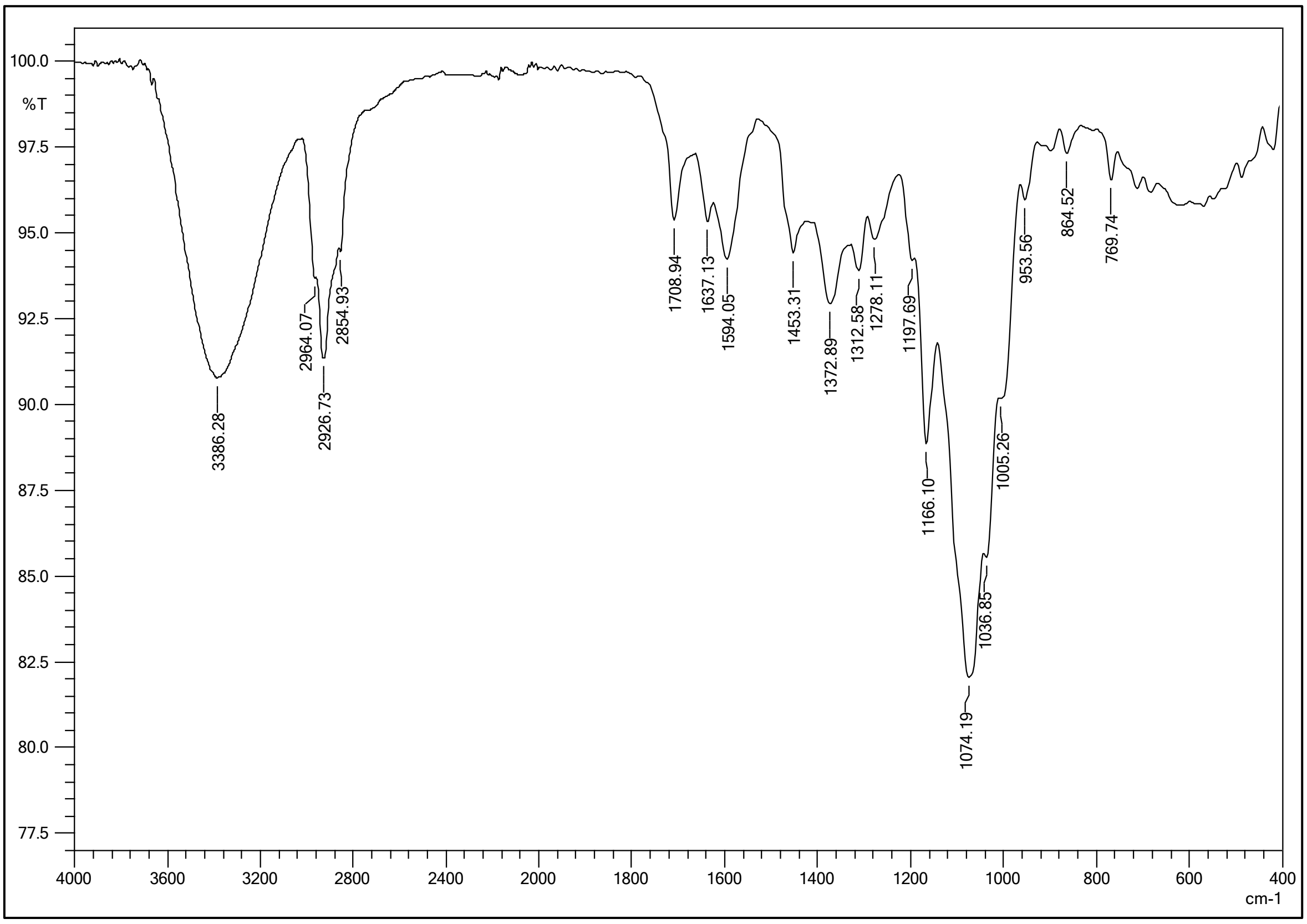

Figure S82. HR-ESI-MS mass spectrum of compound 9.

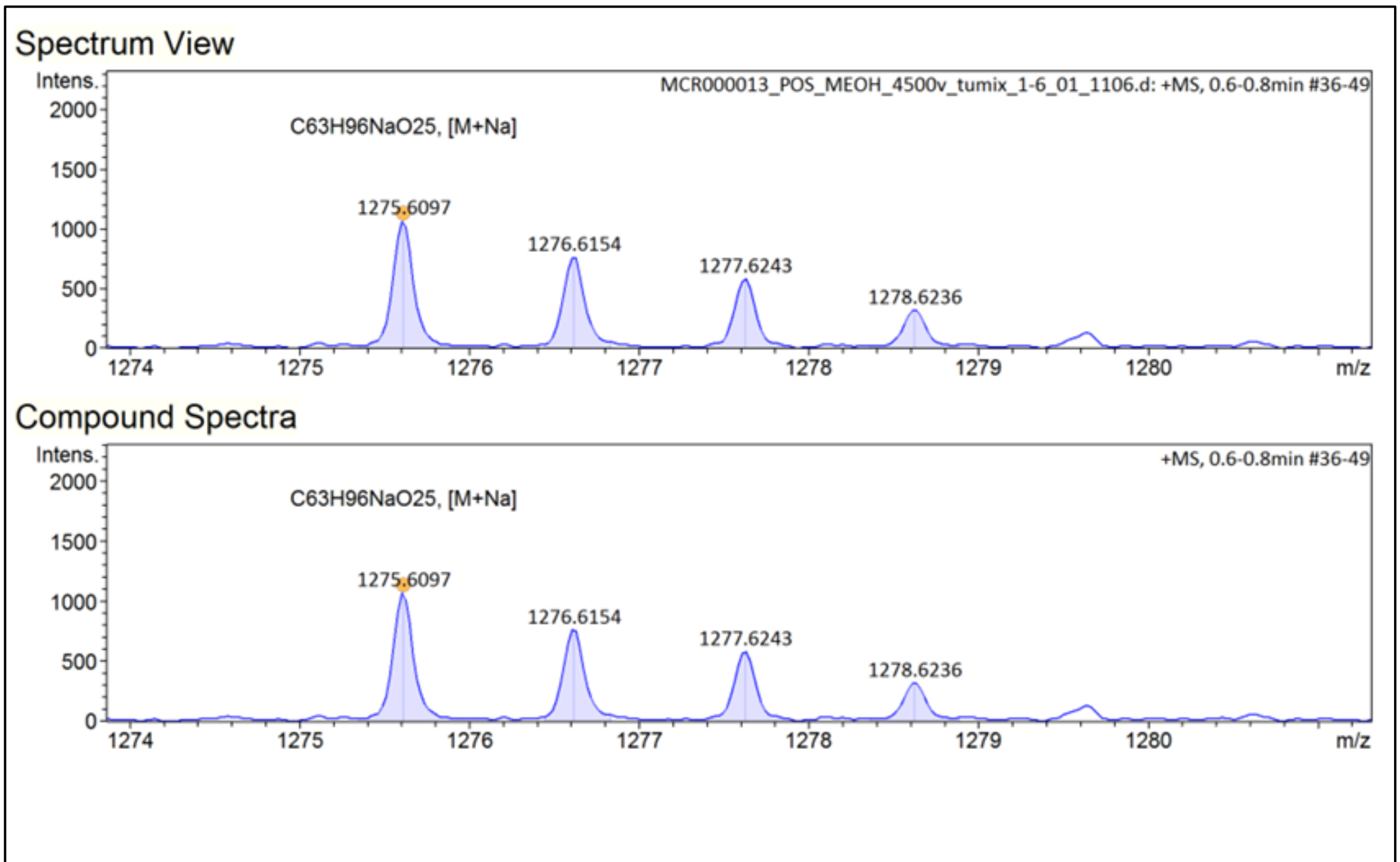

Meas. m/z	#	Ion Formula	m/z	err [ppm]	mSigma	# mSigma	Score	rdb	e⁻ Conf	N-Rule
1275.6097	1	C63H96NaO25	1275.6133	2.8	144.4	1	100.00	15.5	even	ok

Figure S83. ^{1}H NMR spectrum (400 MHz, C_5D_5N) of compound 9

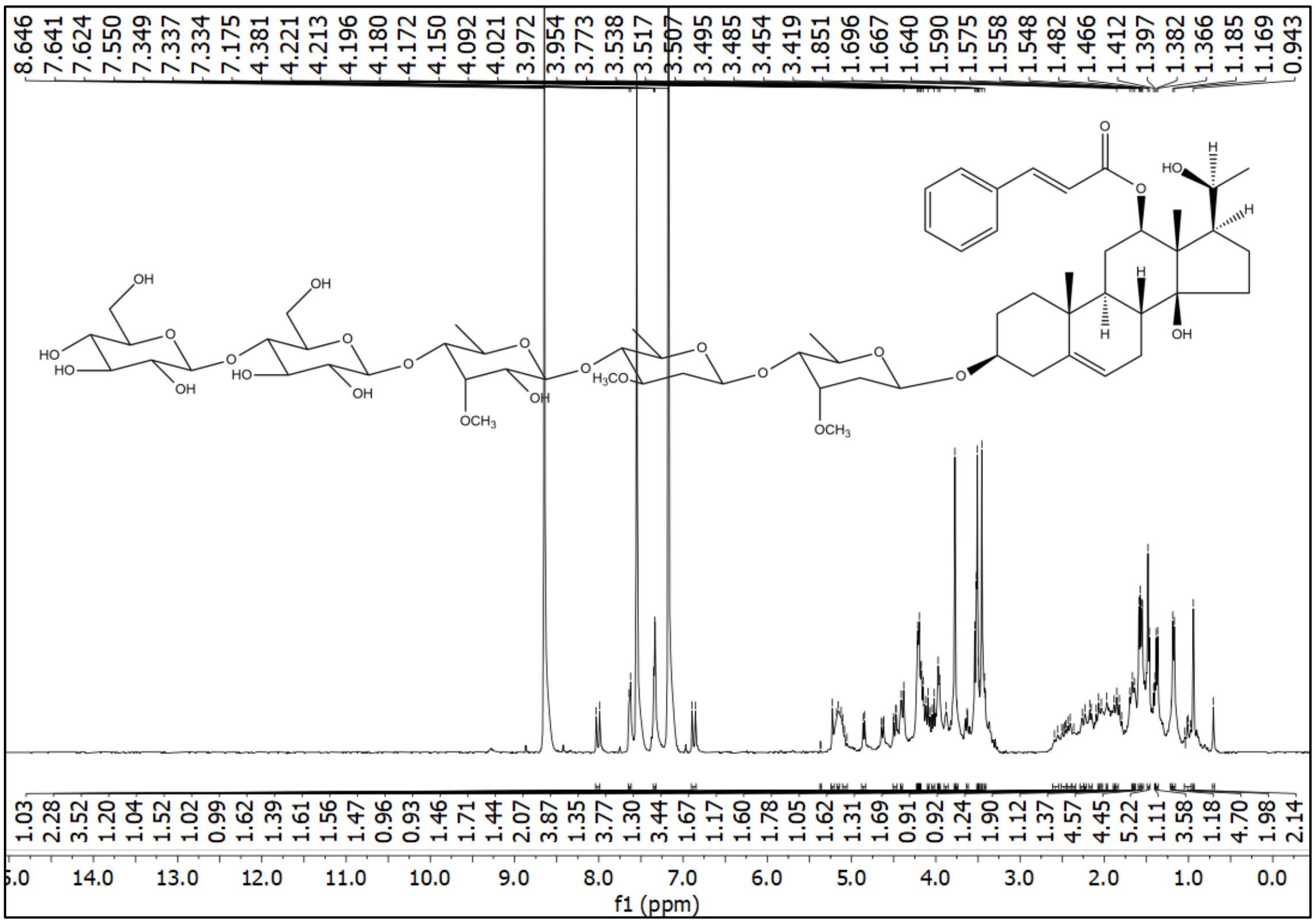

Figure S84. ^{13}C-BB NMR spectrum (100 MHz, C_5D_5N) of compound 9

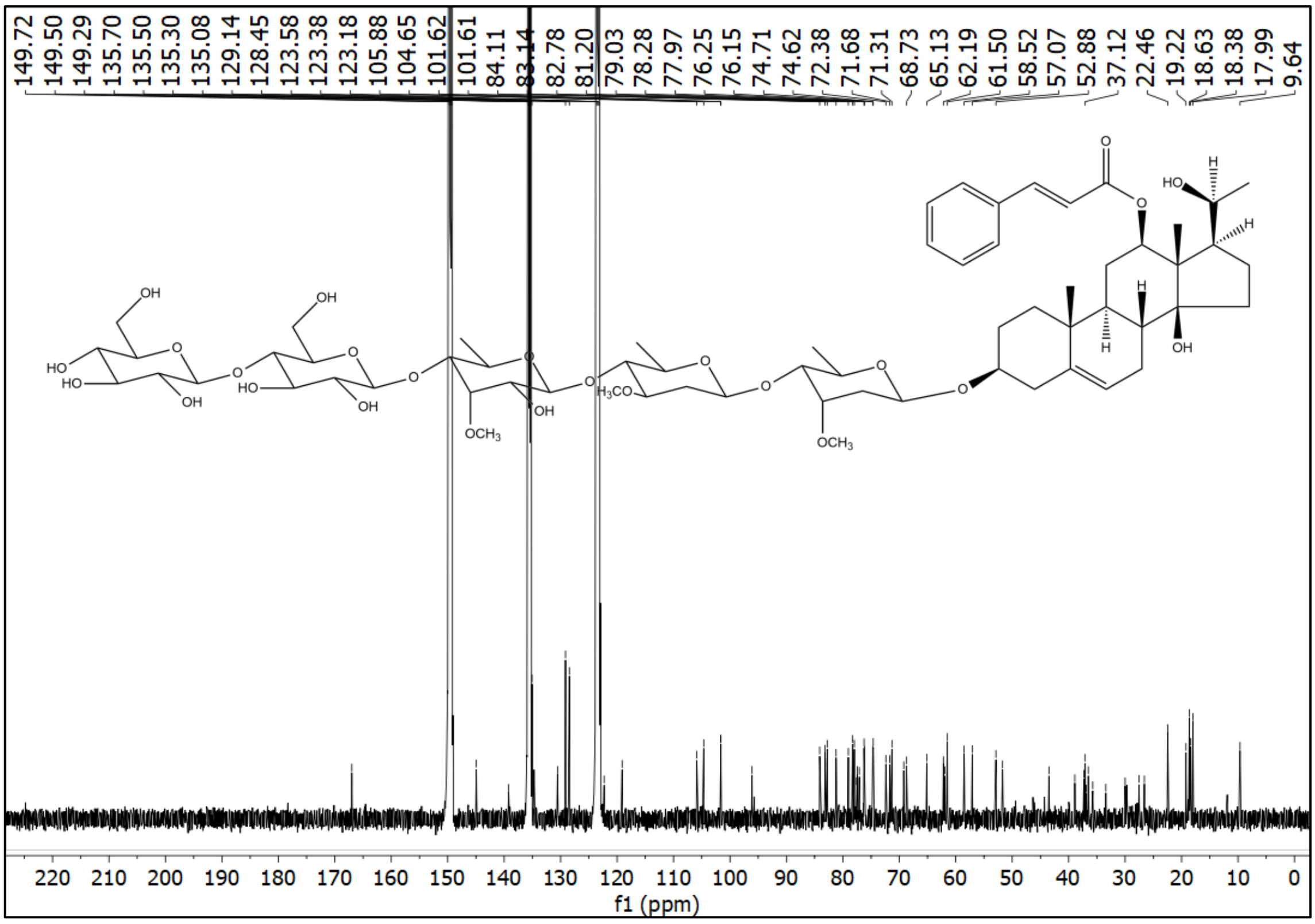

Figure S85. ^{13}C-DEPT135 NMR spectrum (100 MHz, C_5D_5N) of compound 9

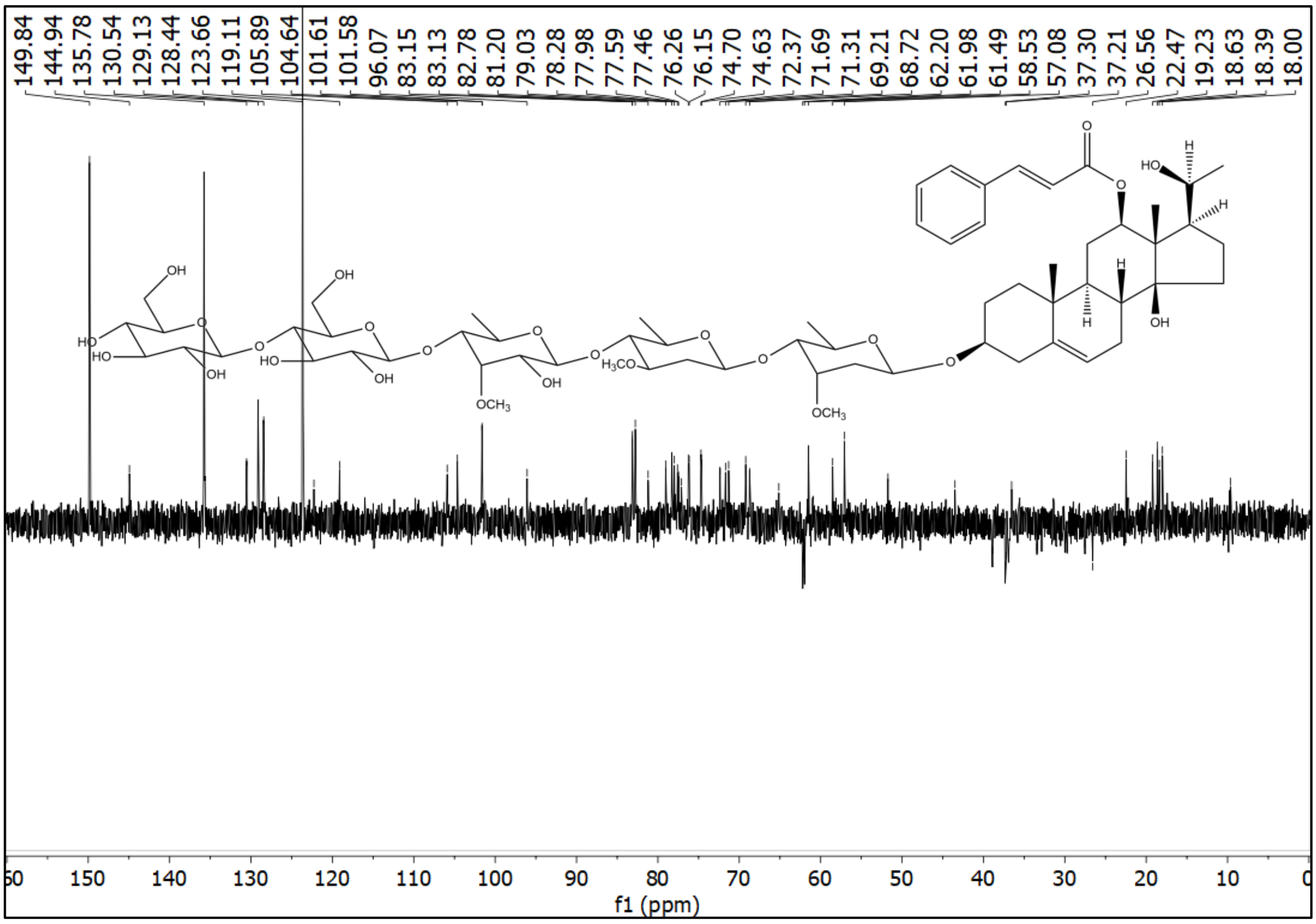

Figure S86. HSQC spectrum (400 x 100 MHz, C_5D_5N) of compound 9

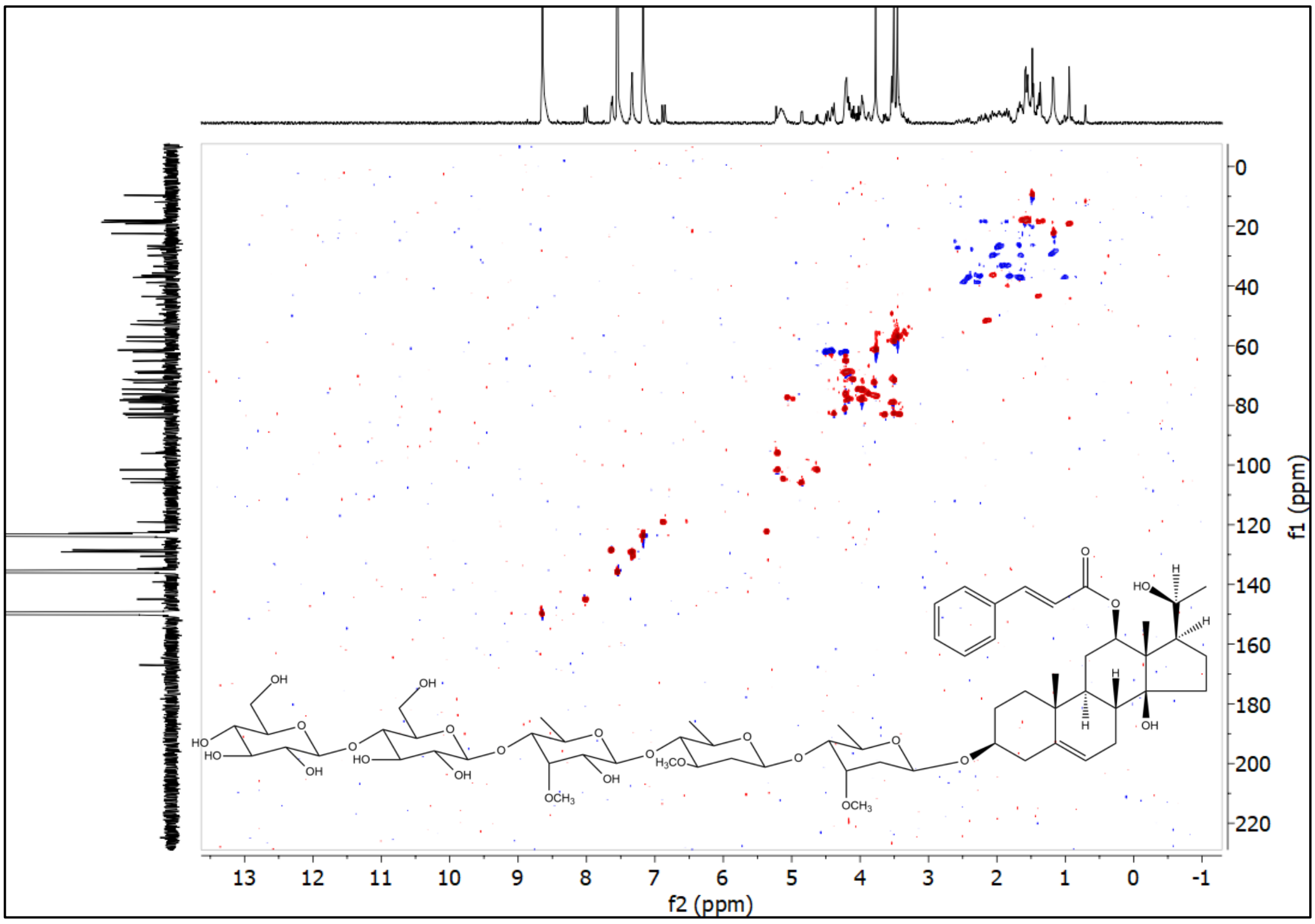

Figure S87. HMBC spectrum (400 x 100 MHz, C_5D_5N) of compound 9

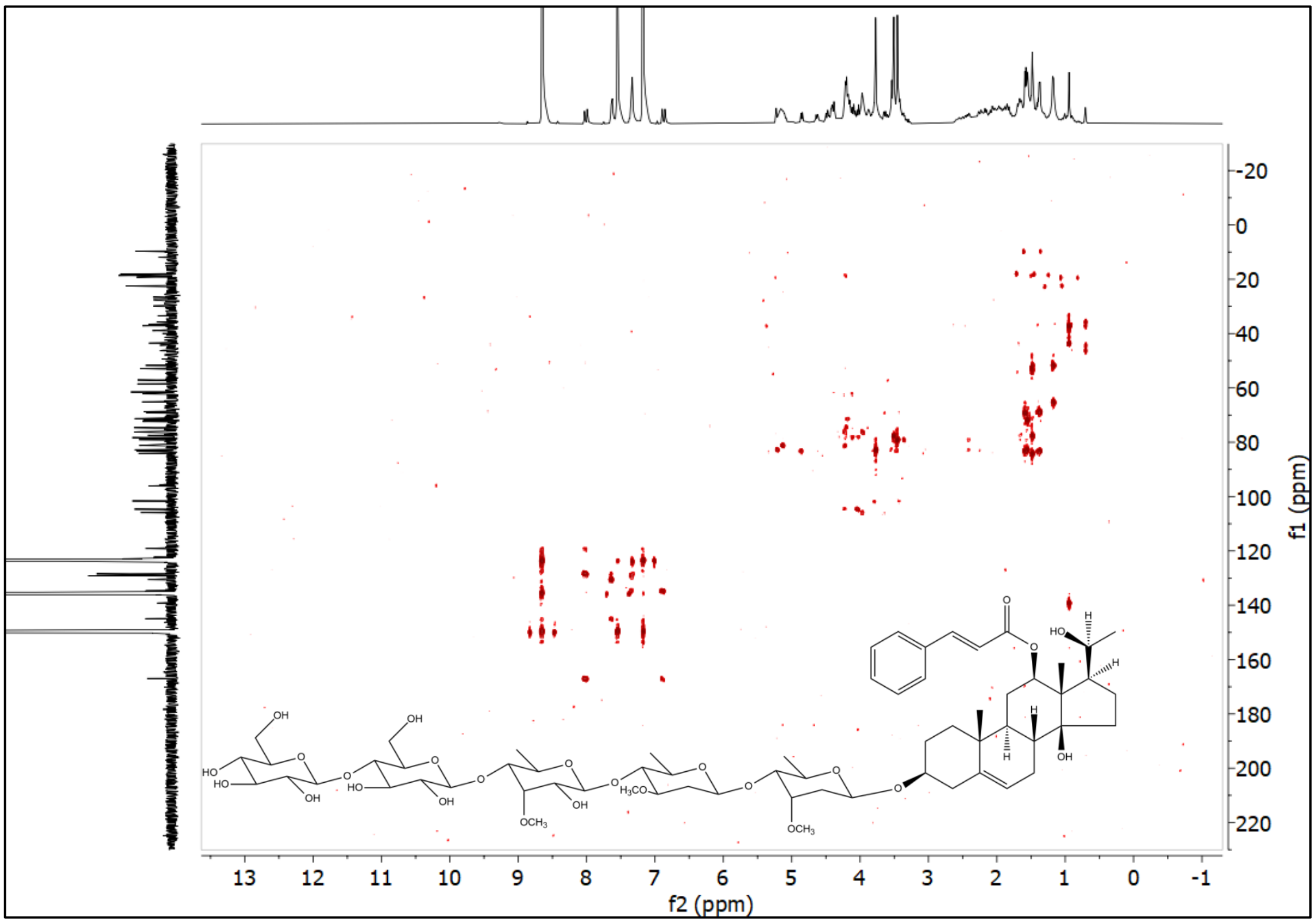

Figure S88. COSY spectrum (400 x 400 MHz, C_5D_5N) of compound 9.

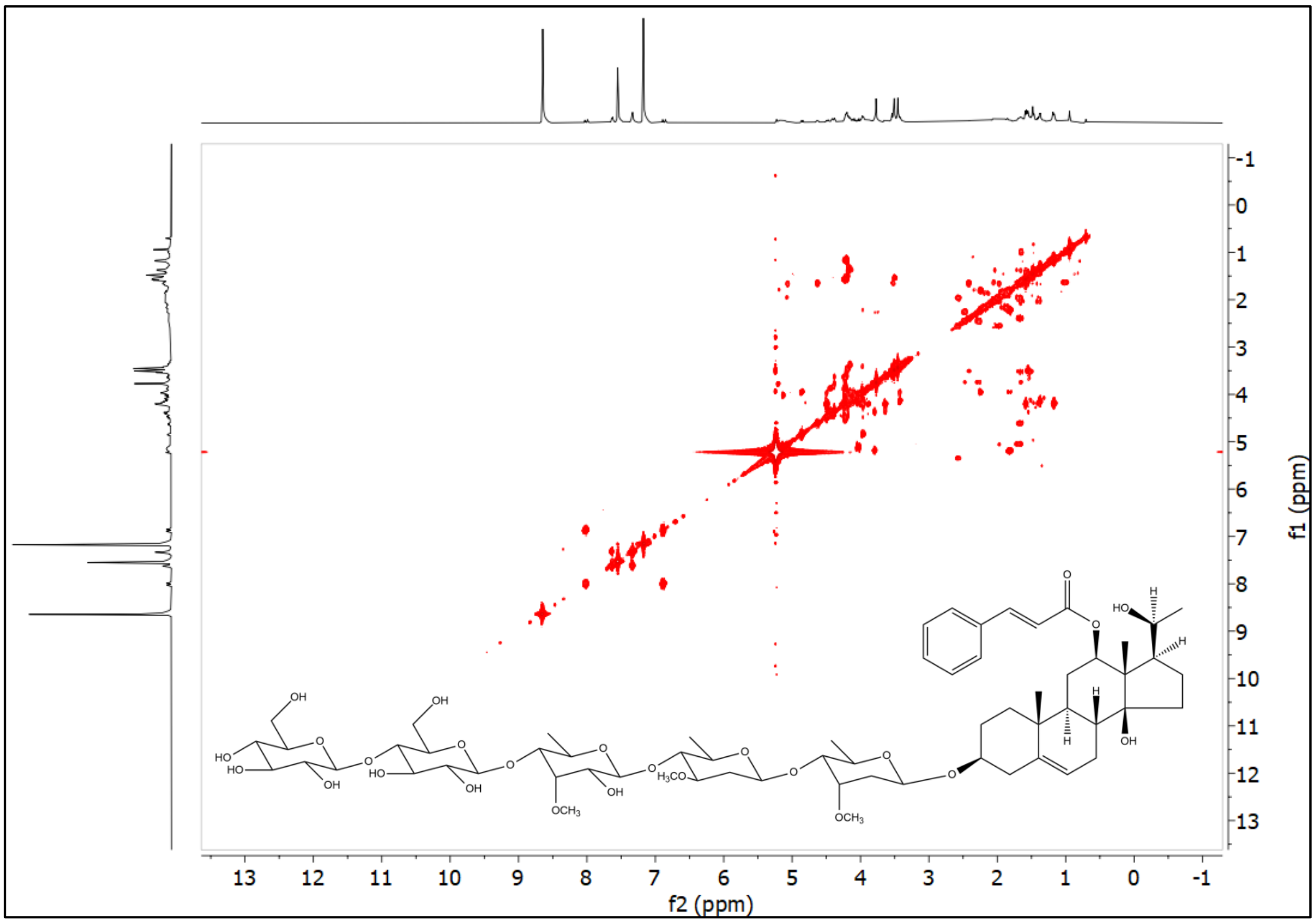

Figure S89. TOCSY spectrum (400 x 400 MHz, C_5D_5N) of compound 9.

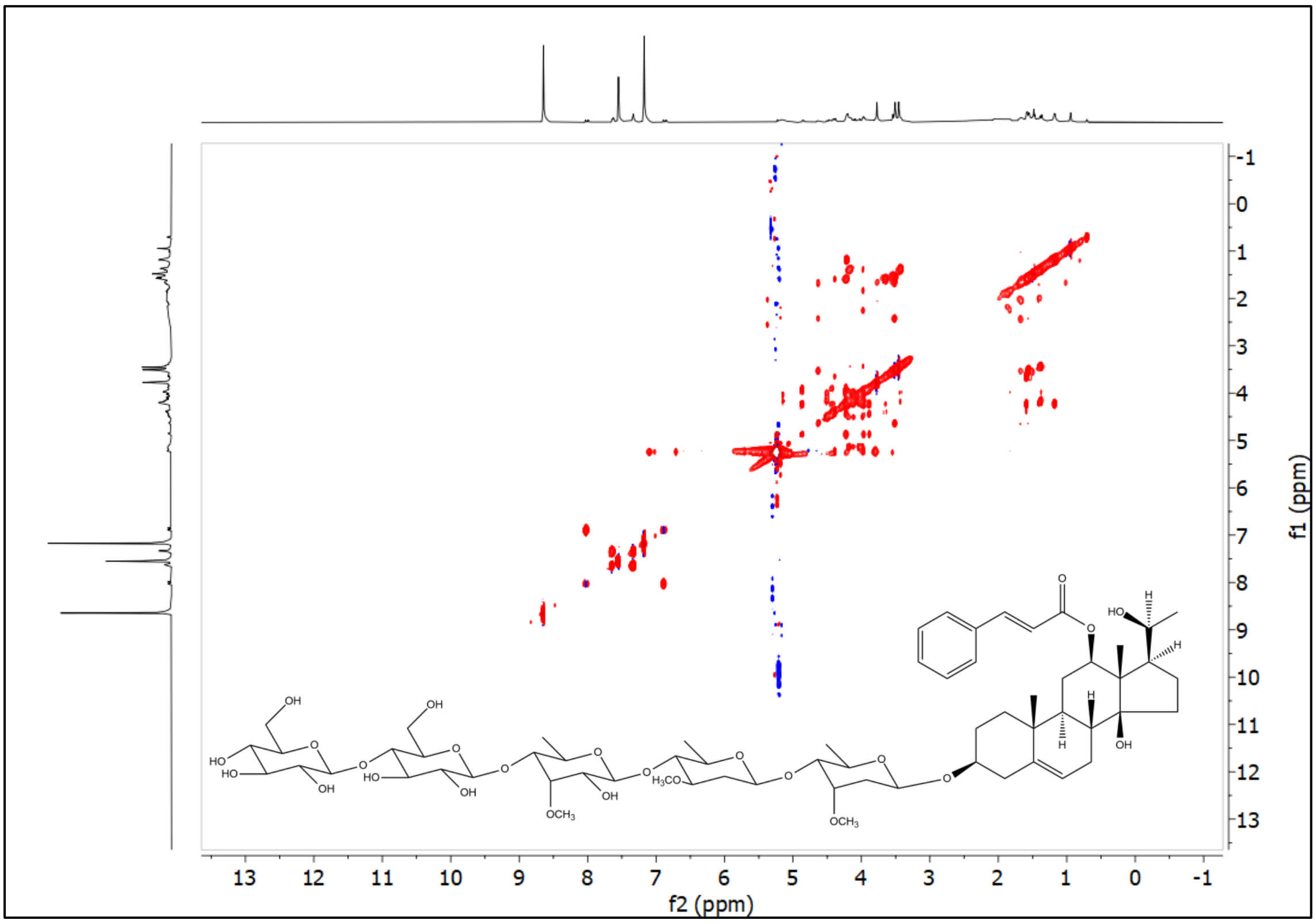

Figure S90. NOESY spectrum (400 x 400 MHz, C_5D_5N) of compound 9.

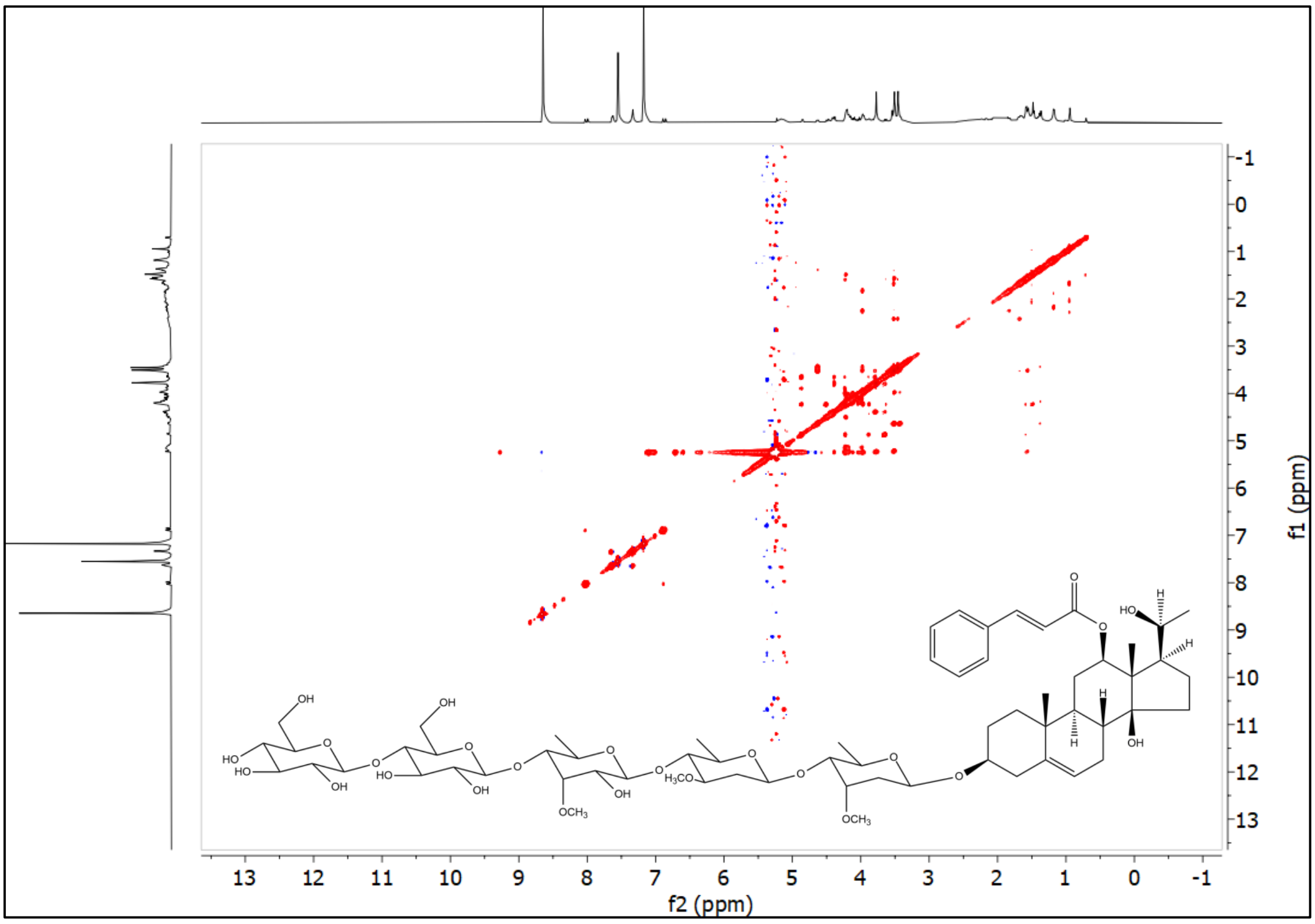

Figure S91. ATR-Infrared spectrum of compound 10.

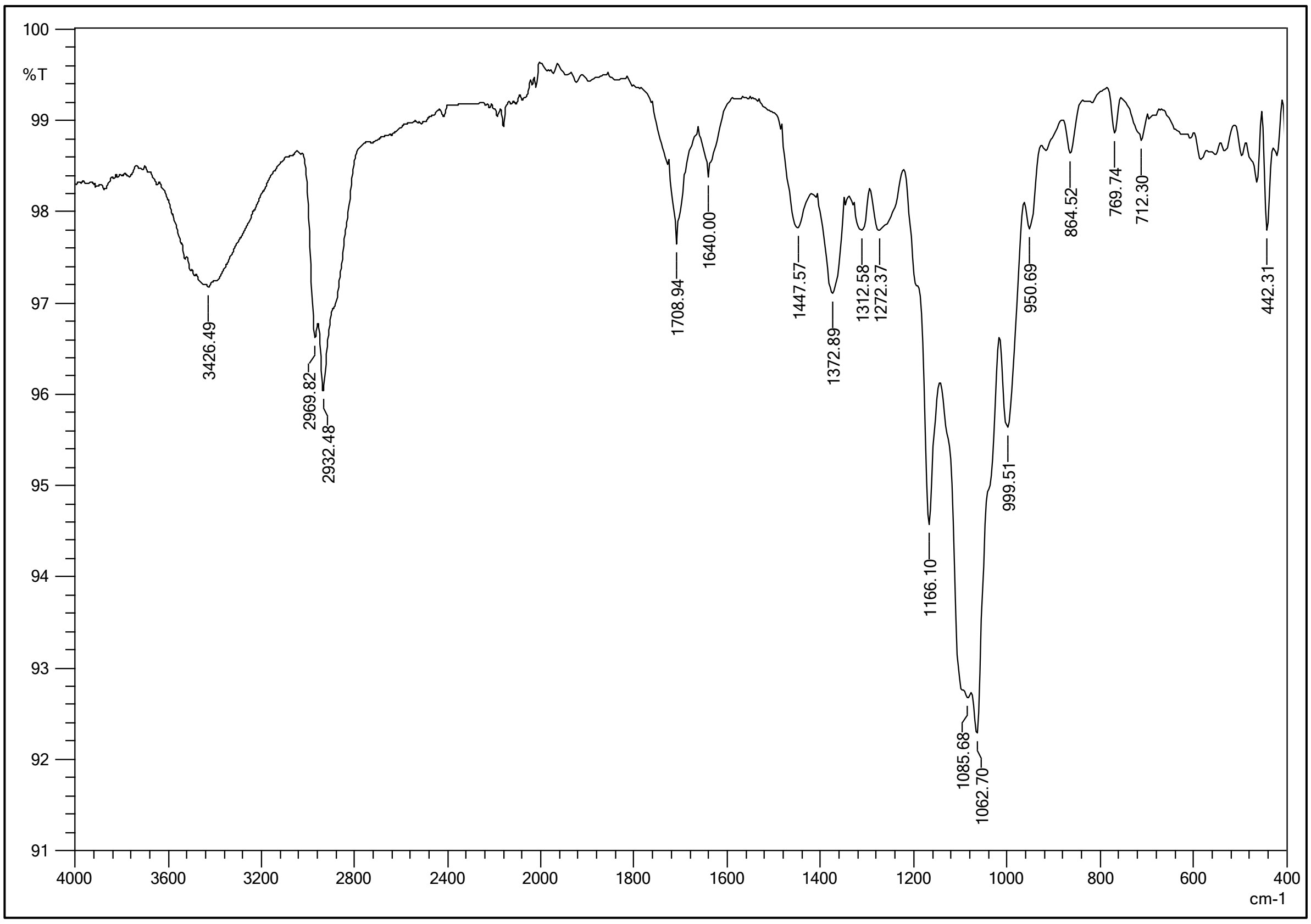

Figure S92. HR-ESI-MS mass spectrum of compound 10.

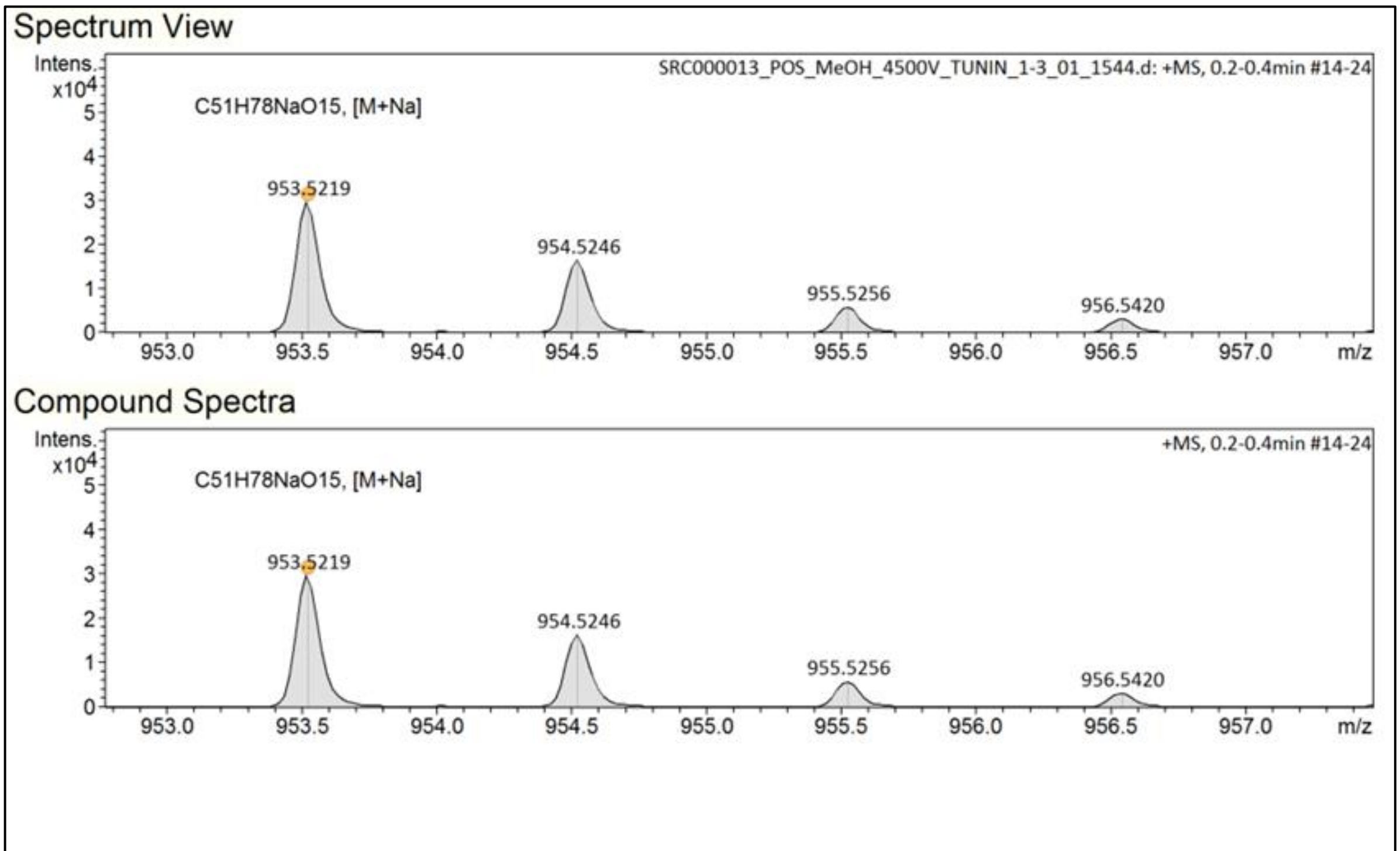

Meas. m/z	#	Ion Formula	m/z	err [ppm]	mSigma	# mSigma	Score	rdb	e⁻ Conf	N-Rule
953.5219	1	$C_{51}H_{78}NaO_{15}$	953.5233	1.5	22.2	1	100.00	12.5	even	ok

Figure S93. ^{1}H NMR spectrum (400 MHz, C_5D_5N) of compound 10

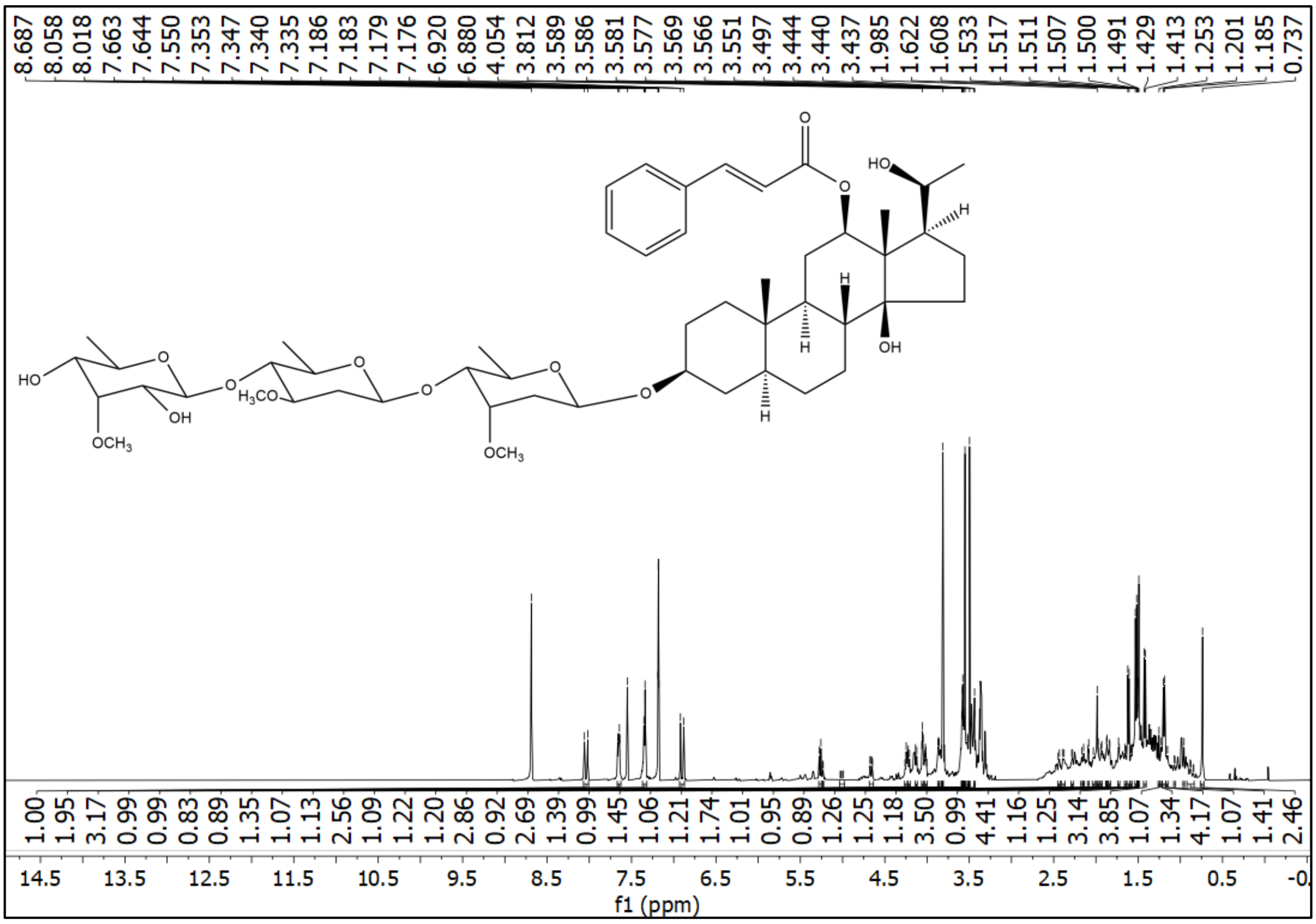

Figure S94. ^{13}C-BB NMR spectrum (100 MHz, C_5D_5N) of compound 10

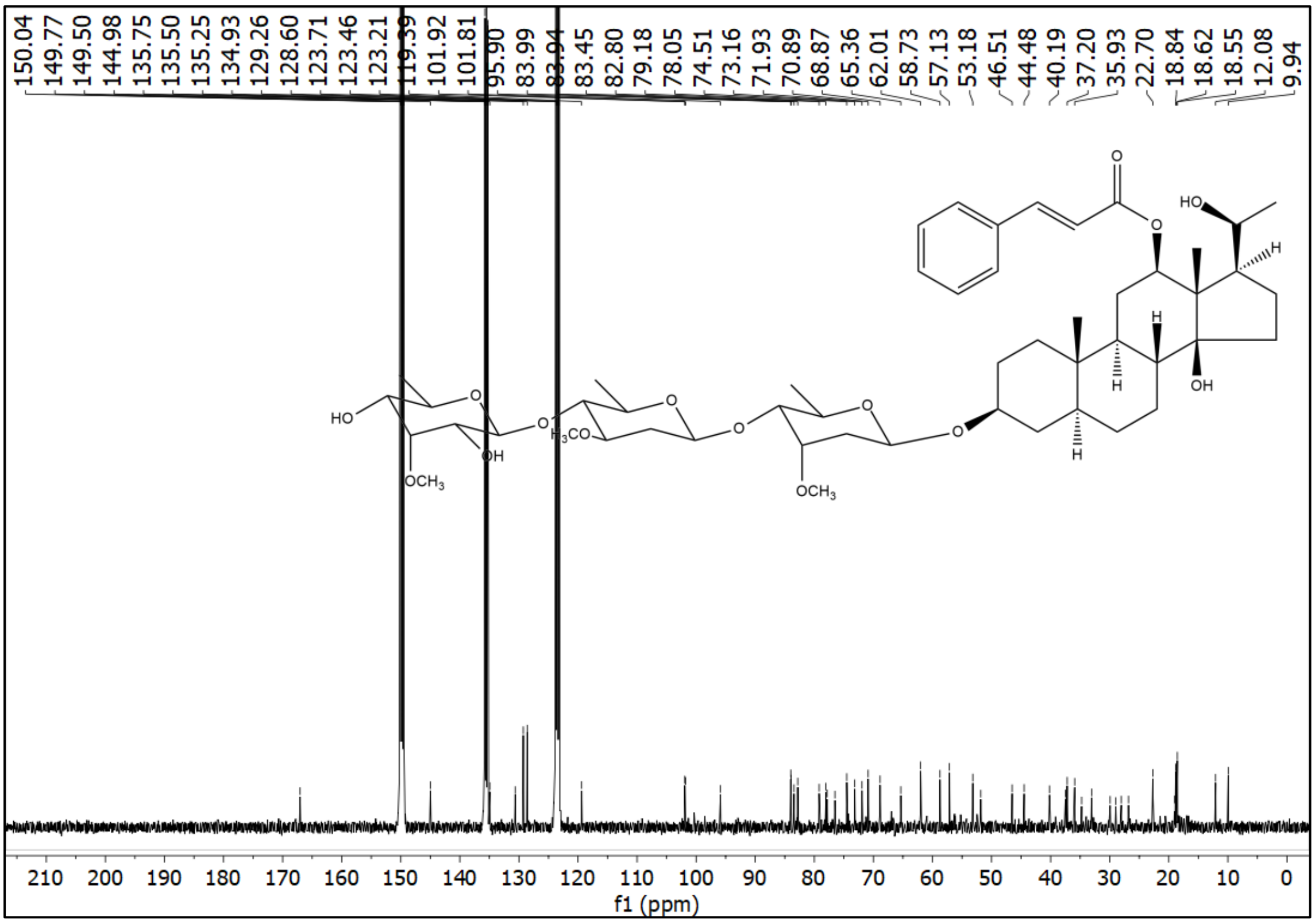

Figure S95. ^{13}C-DEPT135 NMR spectrum (100 MHz, C_5D_5N) of compound 10

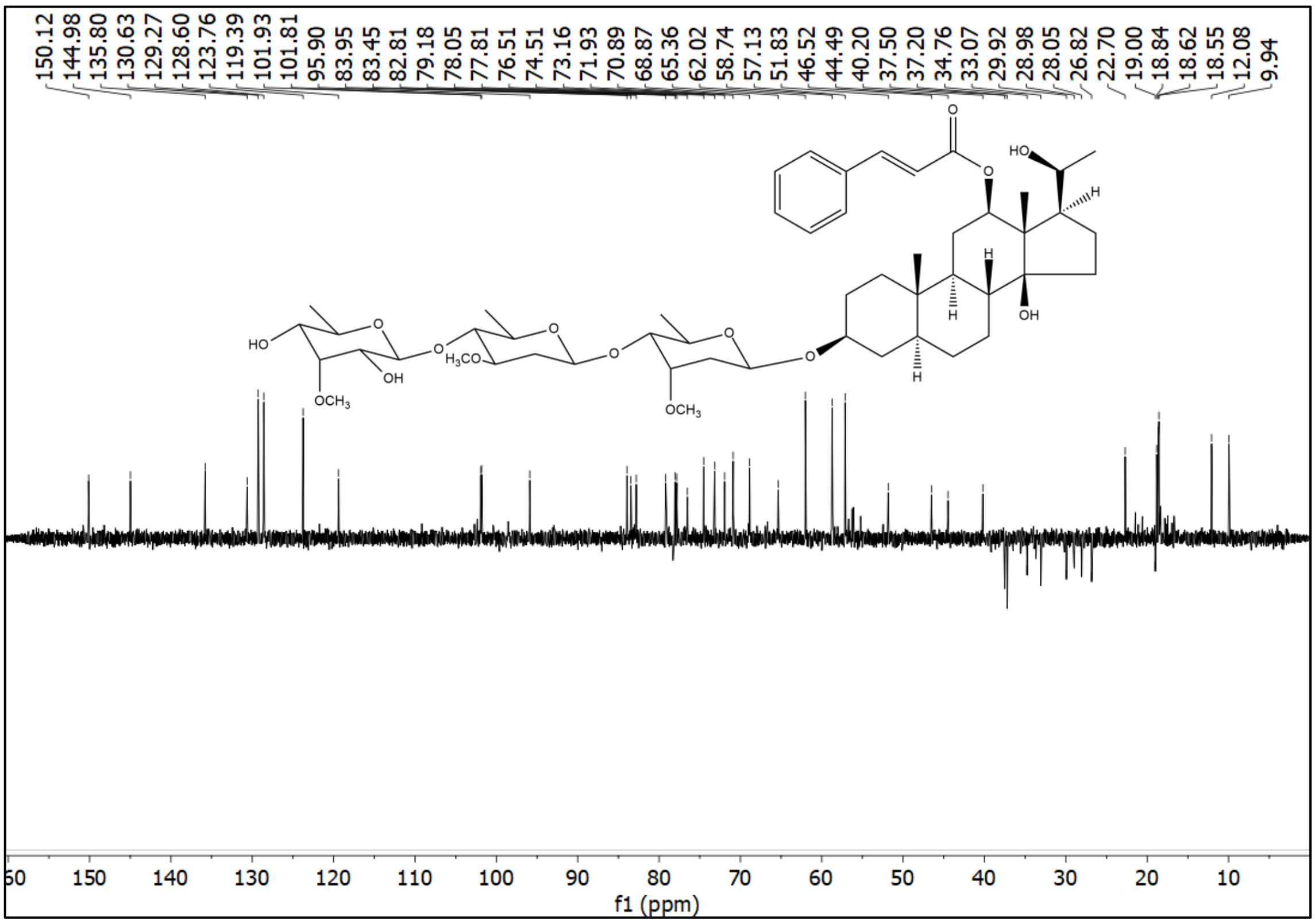

Figure S96. HSQC spectrum (400 x 100 MHz, C_5D_5N) of compound 10

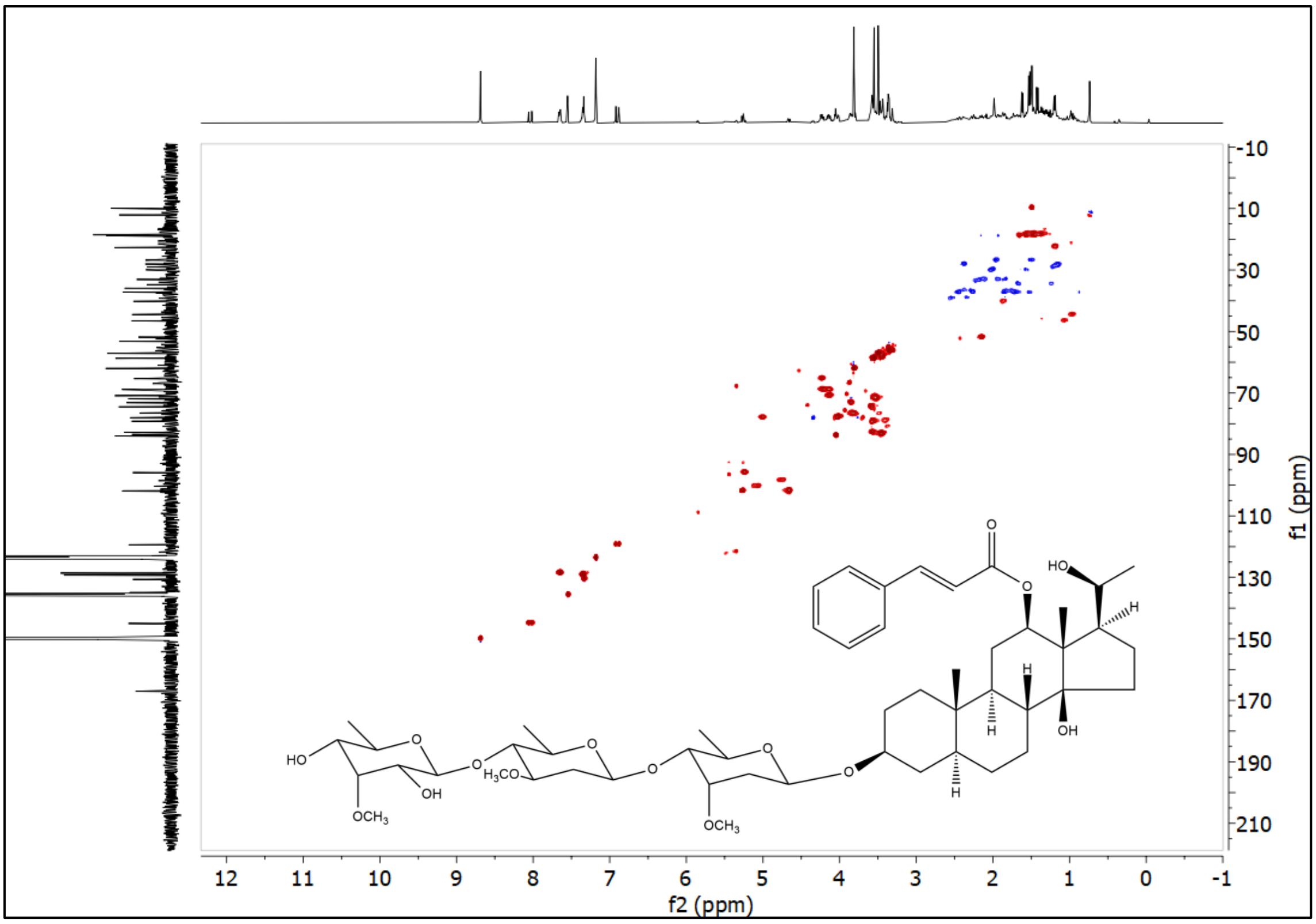

Figure S97. HMBC spectrum (400 x 100 MHz, C_5D_5N) of compound 10

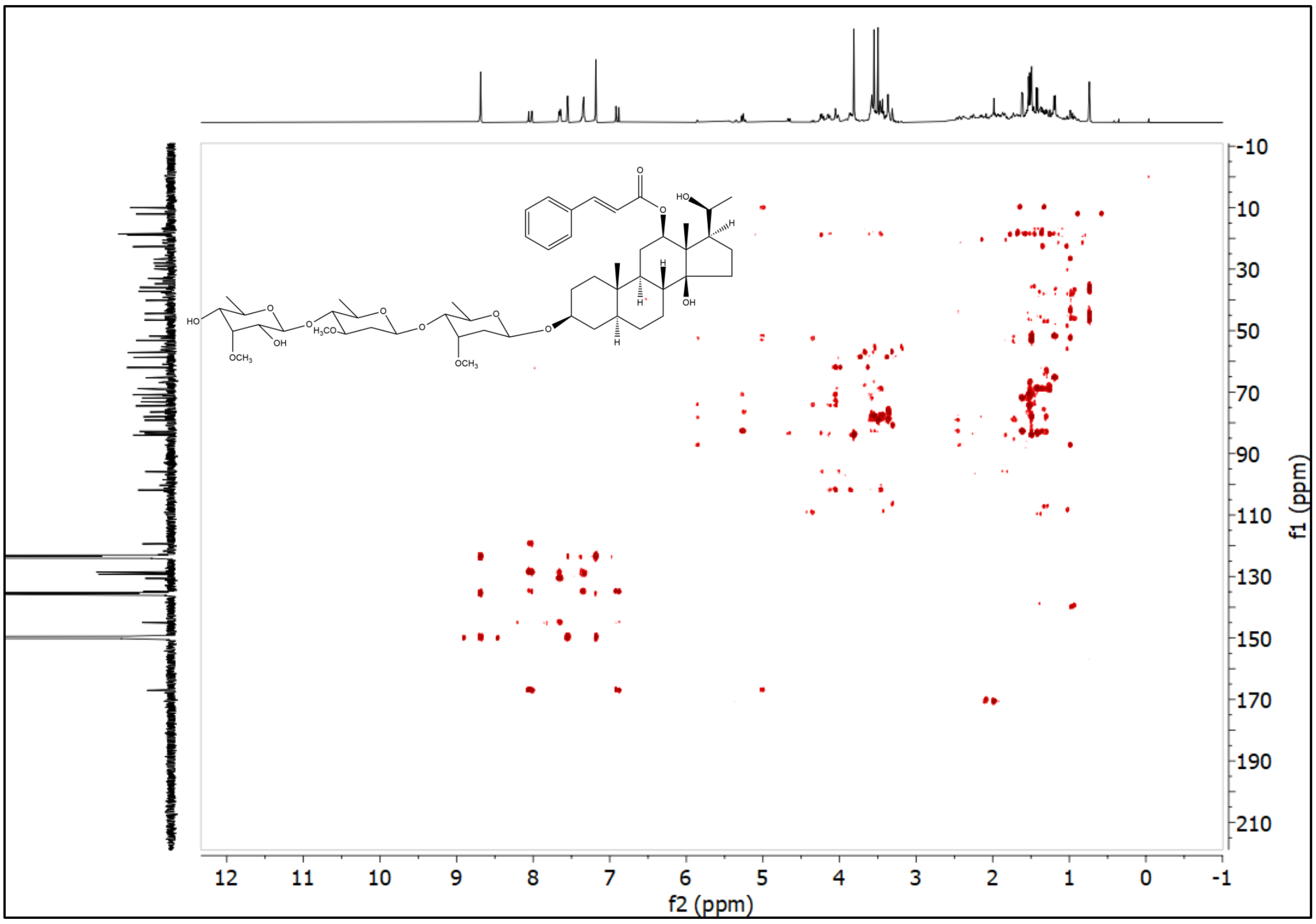

Figure S98. COSY spectrum (400 x 400 MHz, C_5D_5N) of compound 10.

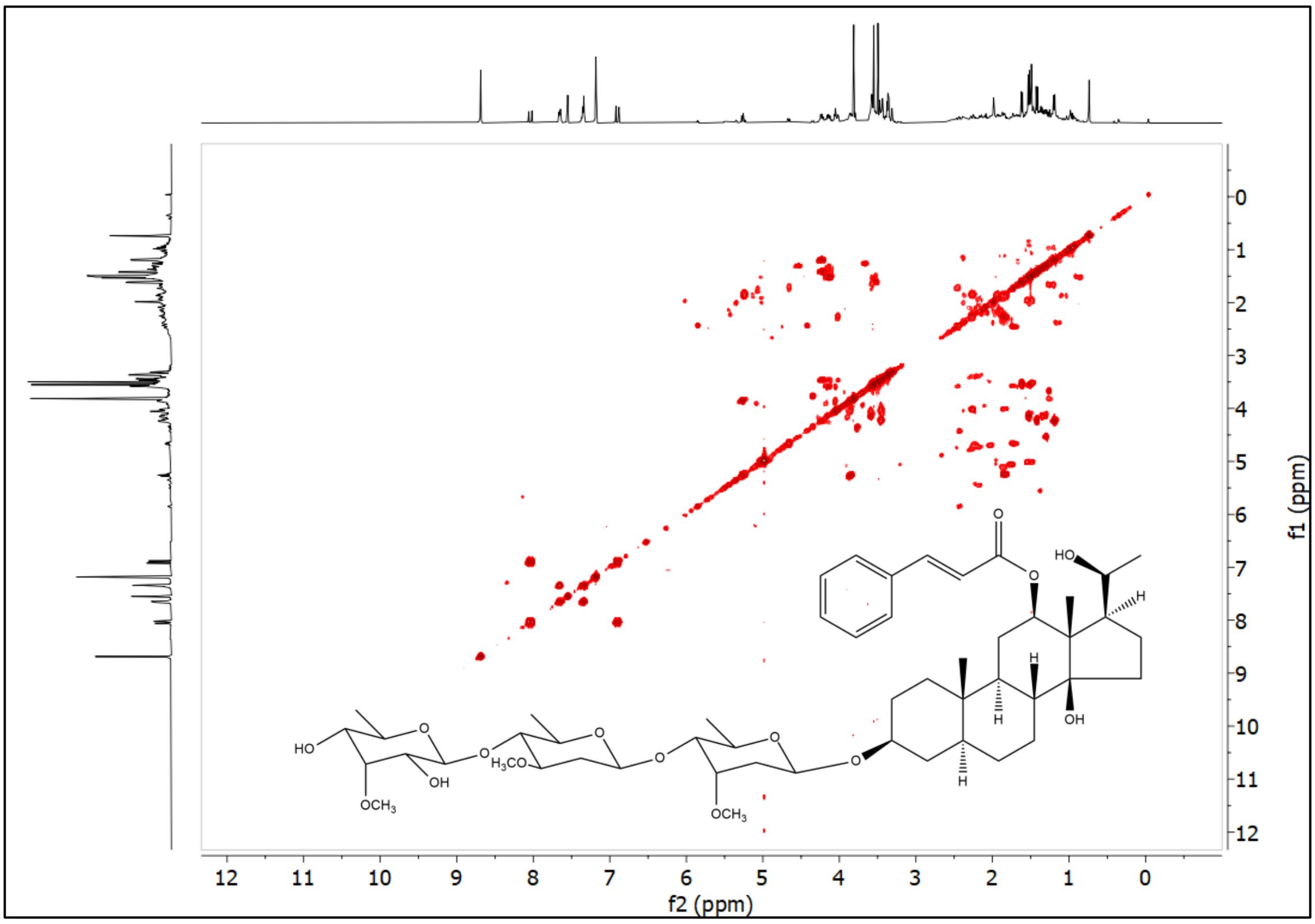

Figure S99. TOCSY spectrum (400 x 400 MHz, C_5D_5N) of compound 10.

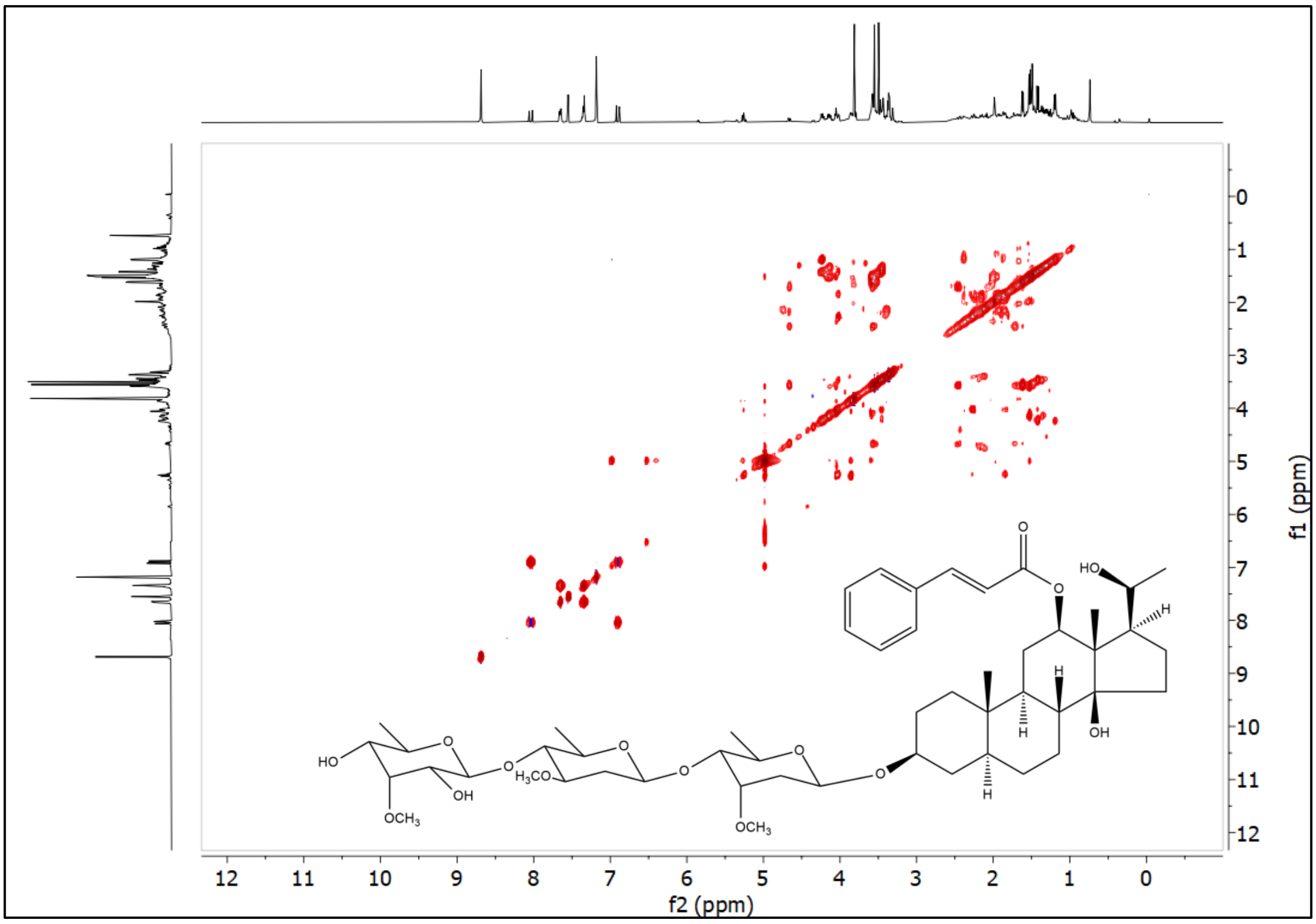

Figure S100. ROESY spectrum (400 x 400 MHz, C_5D_5N) of compound 10.

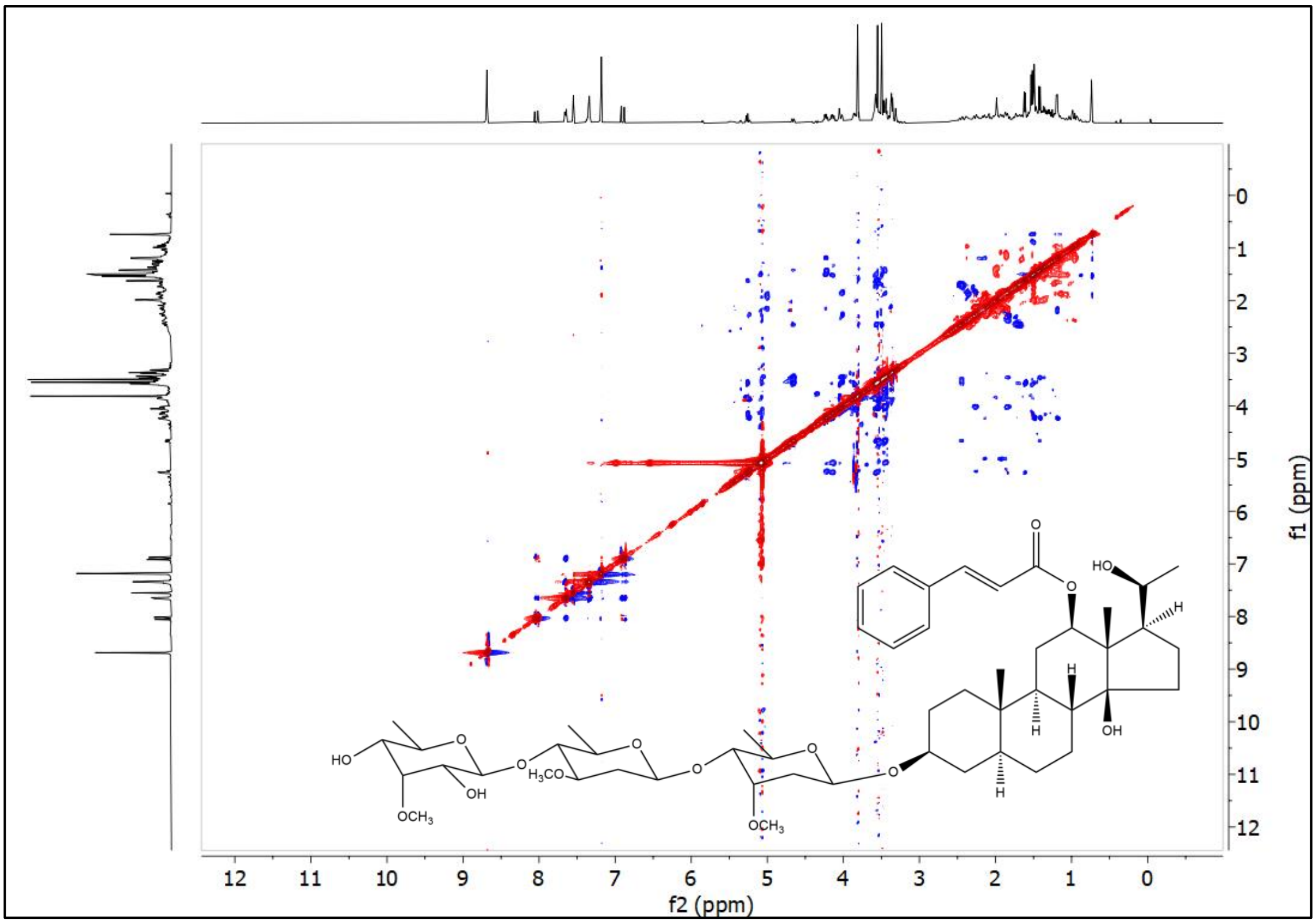

Figure S101. ATR-Infrared spectrum of compound 11.

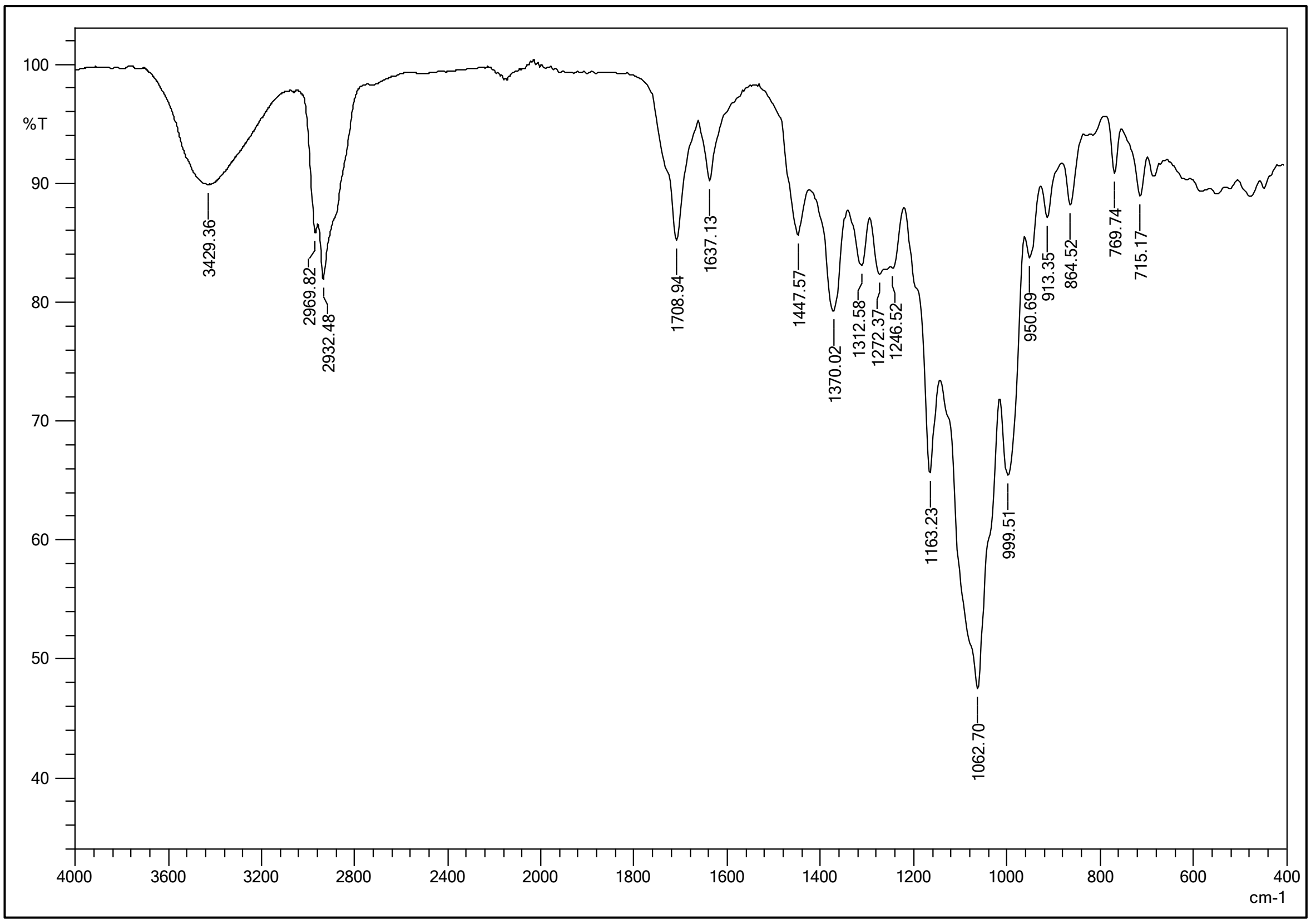

Figure S102. HR-ESI-MS mass spectrum of compound 11.

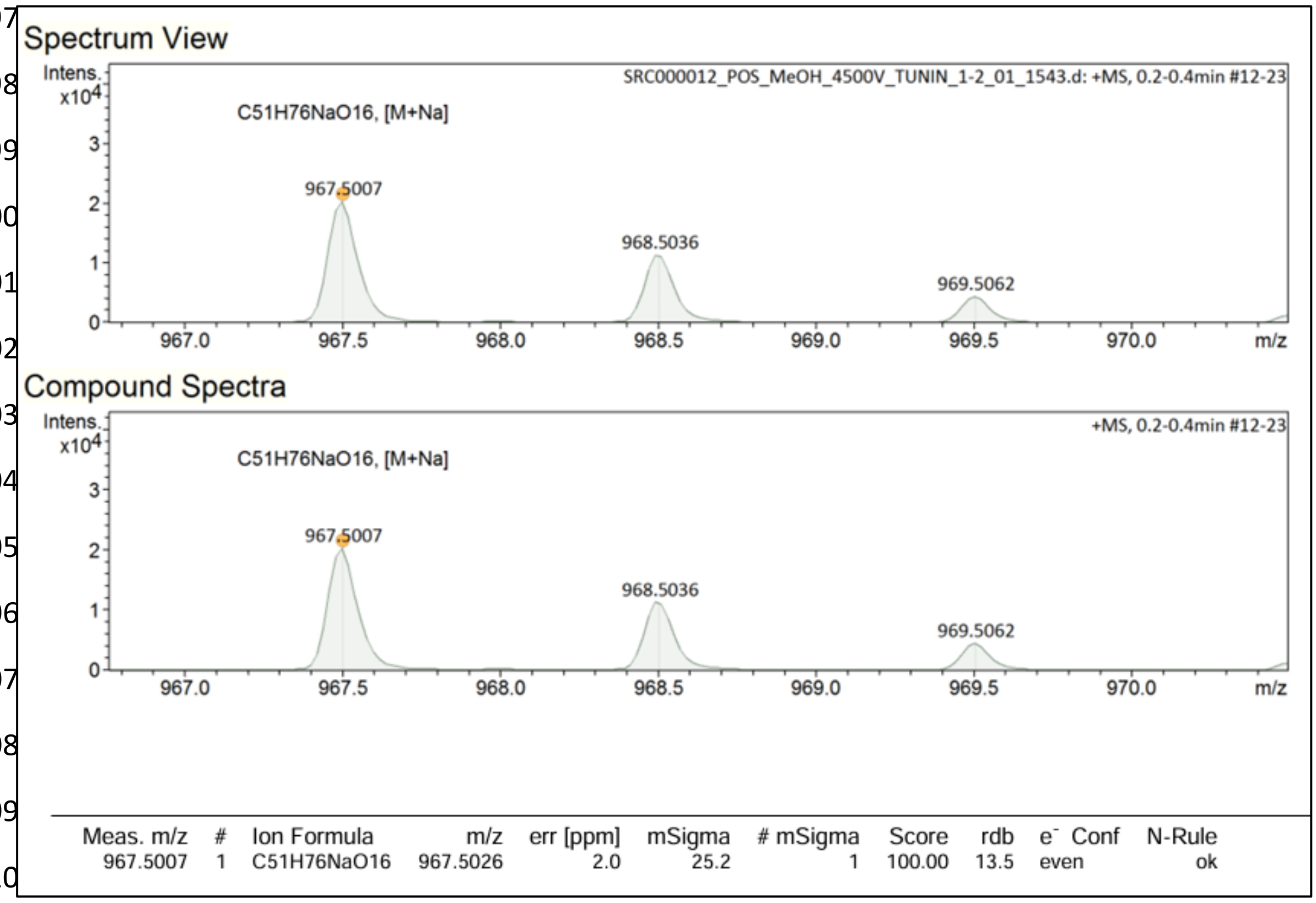

Figure S103. ^{1}H NMR spectrum (400 MHz, C_5D_5N) of compound 11

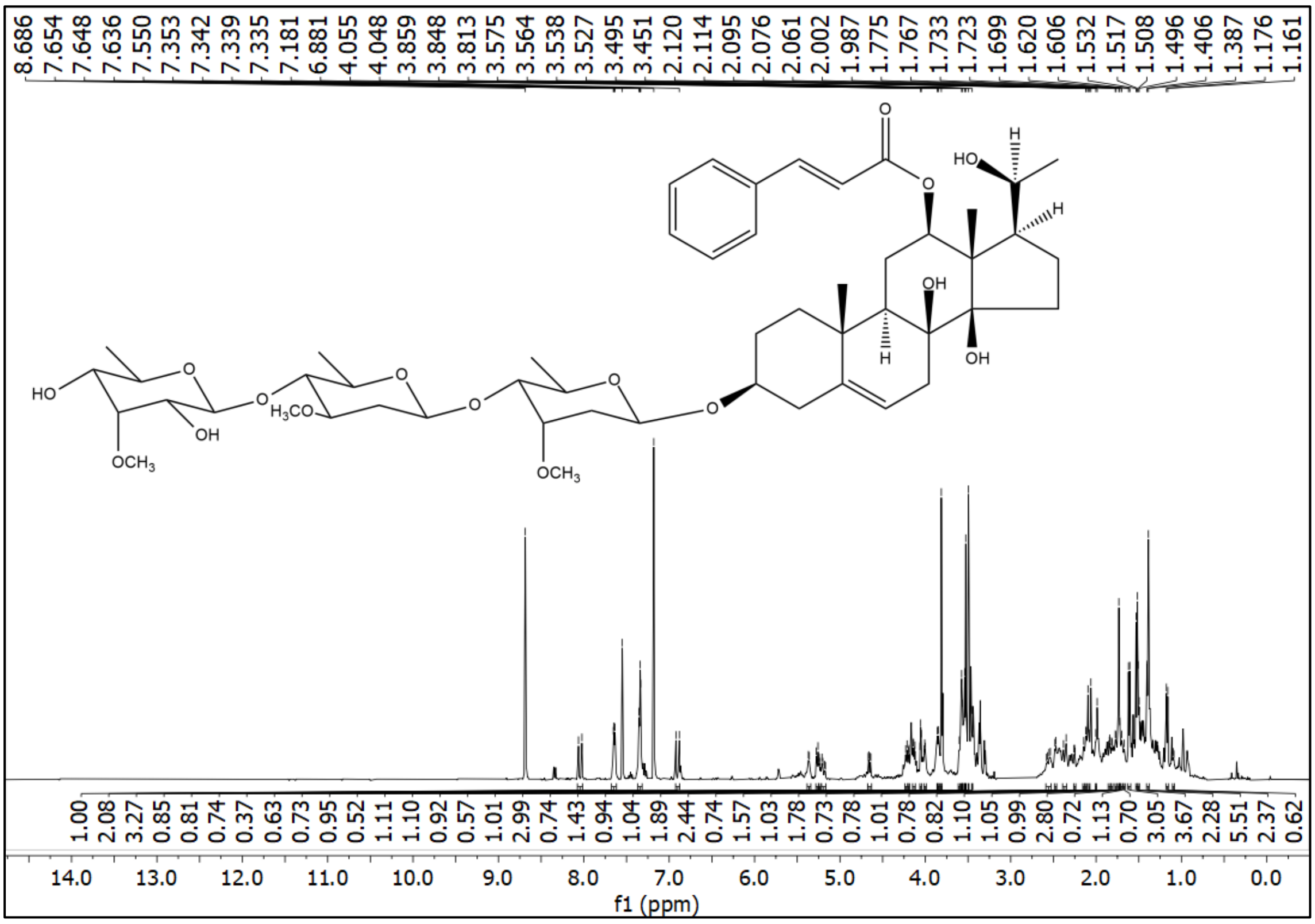

Figure S104. ^{13}C-BB NMR spectrum (100 MHz, C_5D_5N) of compound 11

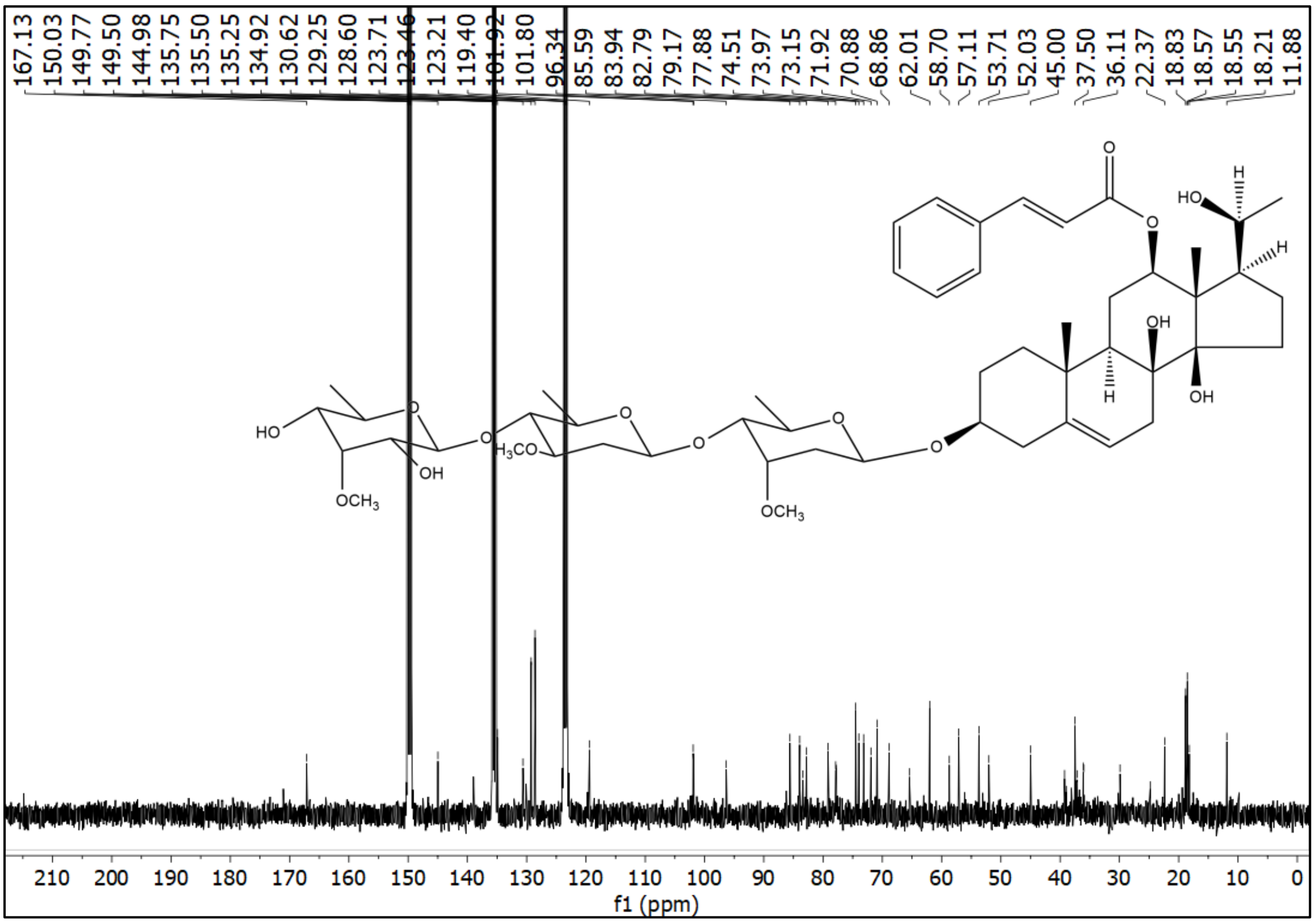

Figure S105. ^{13}C-DEPT135 NMR spectrum (100 MHz, C_5D_5N) of compound 11

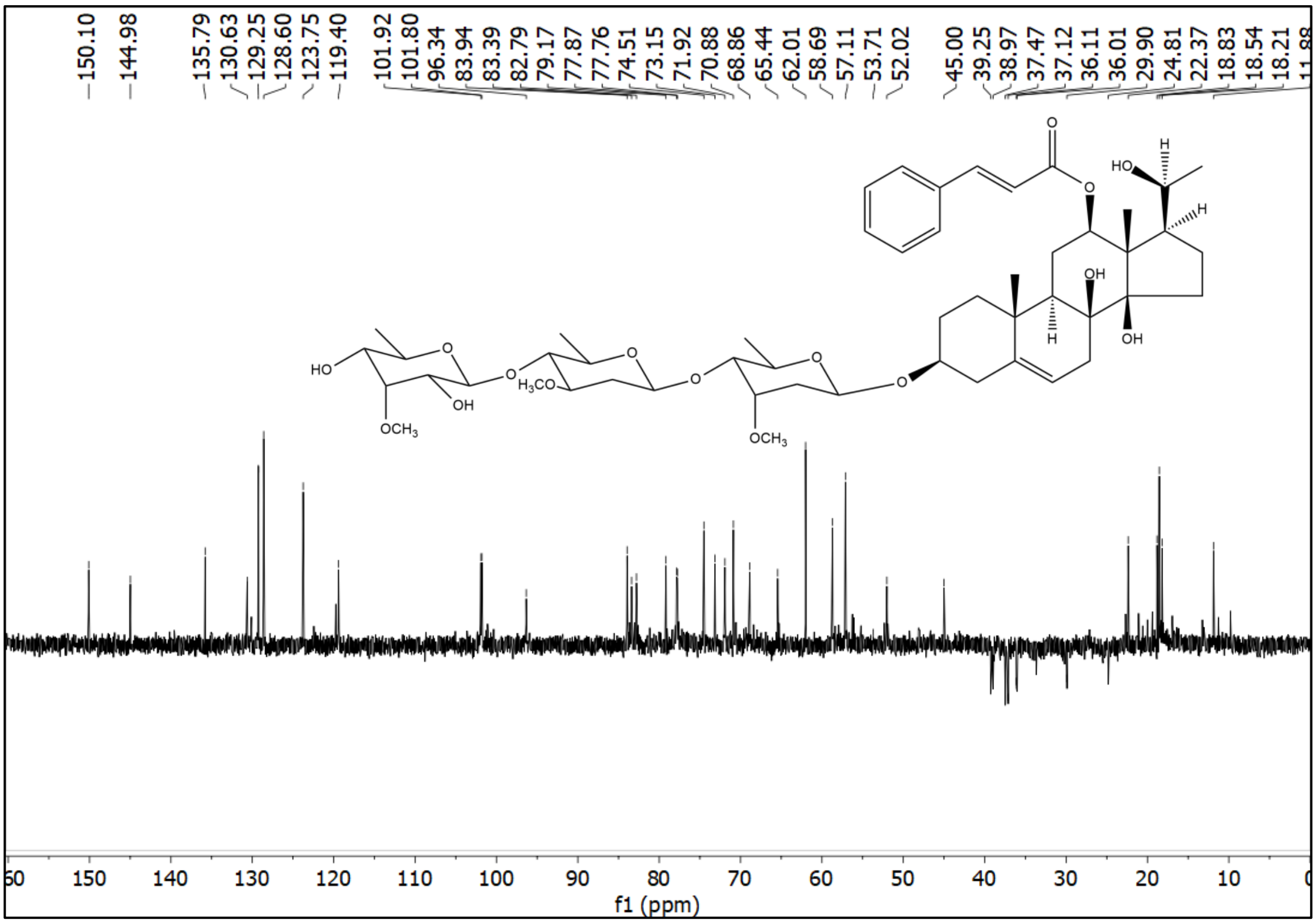

Figure S106. HSQC spectrum (400 x 100 MHz, C_5D_5N) of compound 11

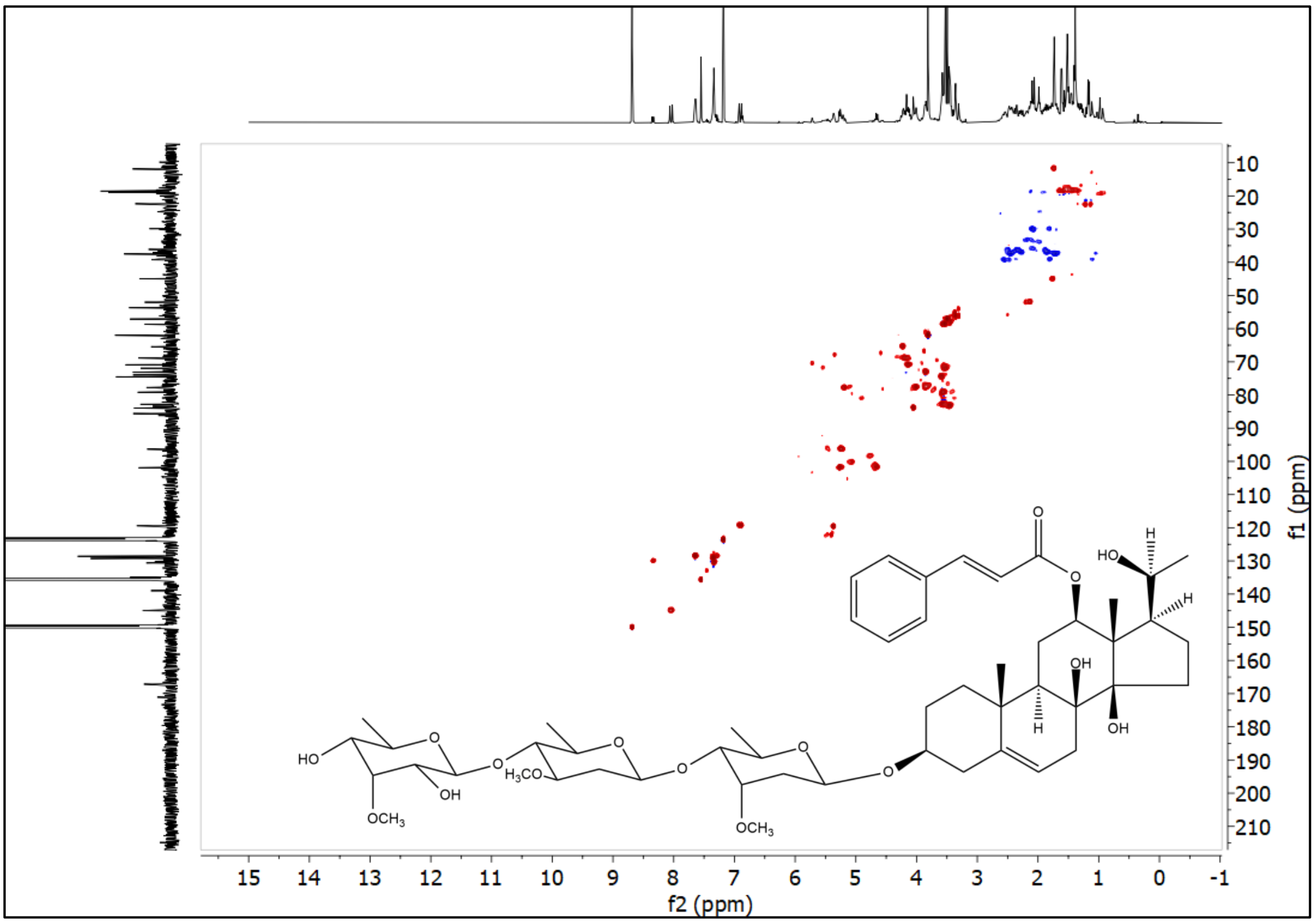

Figure S107. HMBC spectrum (400 x 100 MHz, C_5D_5N) of compound 11

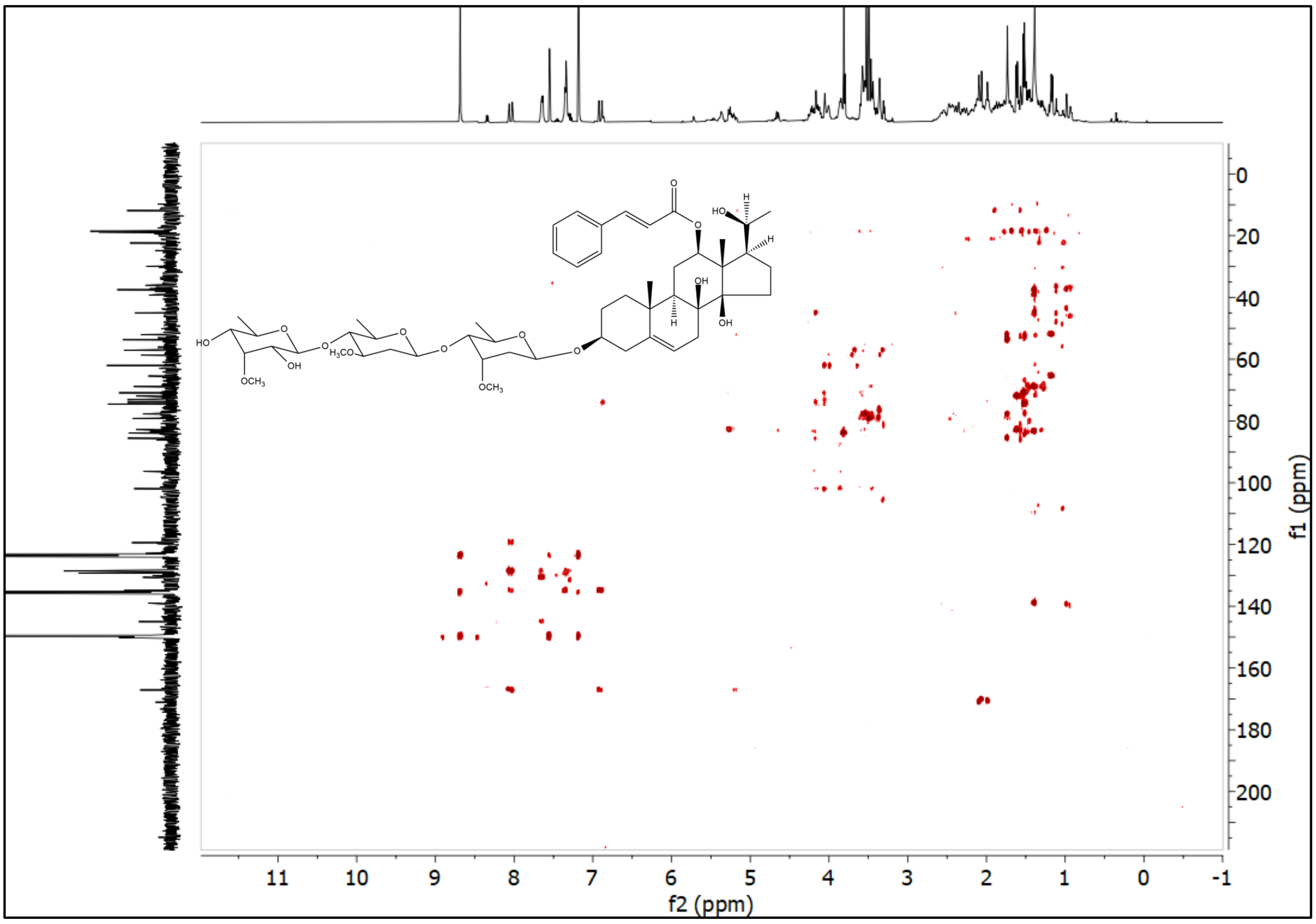

Figure S108. COSY spectrum (400 x 400 MHz, C_5D_5N) of compound 11.

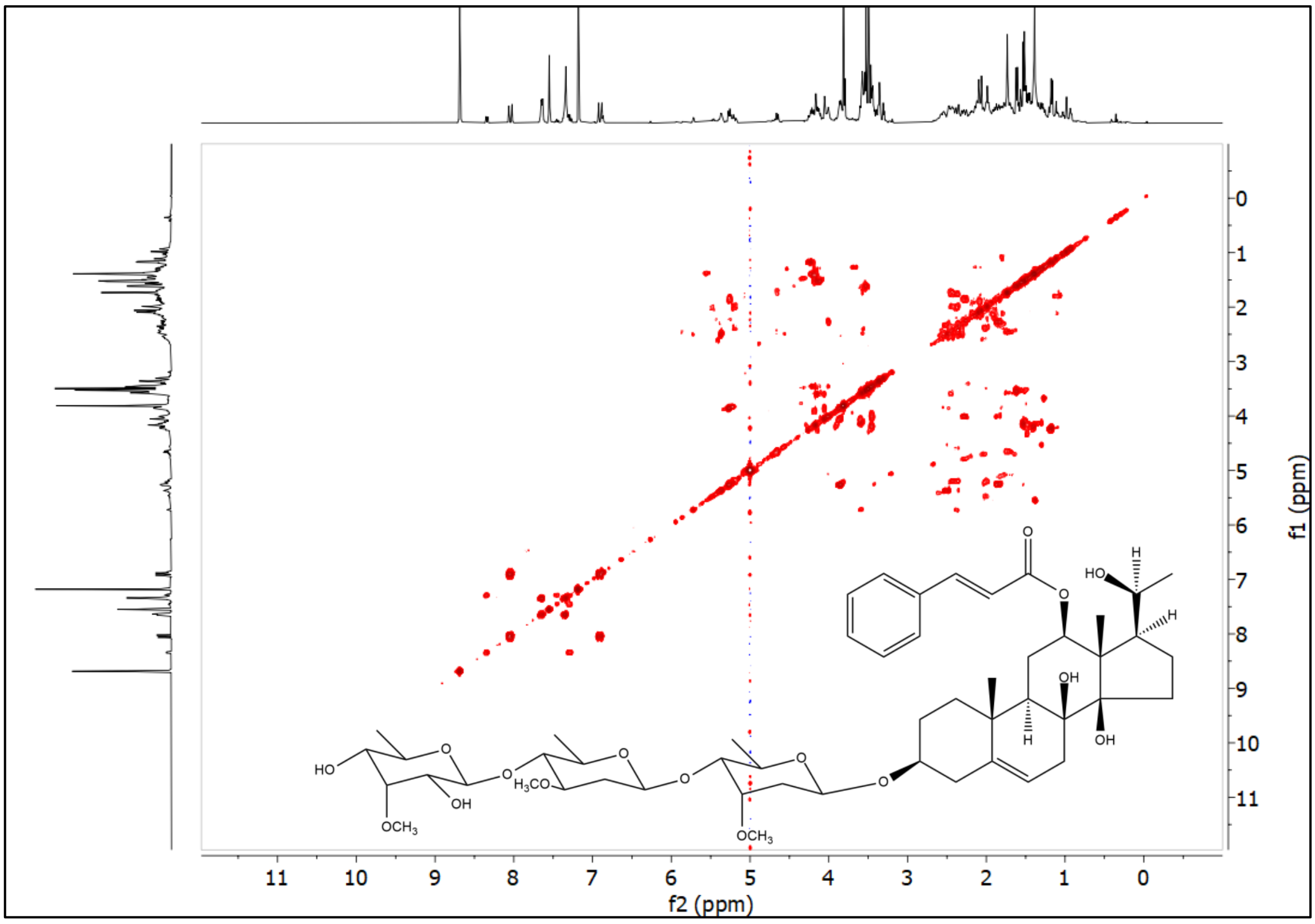

Figure S109. TOCSY spectrum (400 x 400 MHz, C_5D_5N) of compound 11.

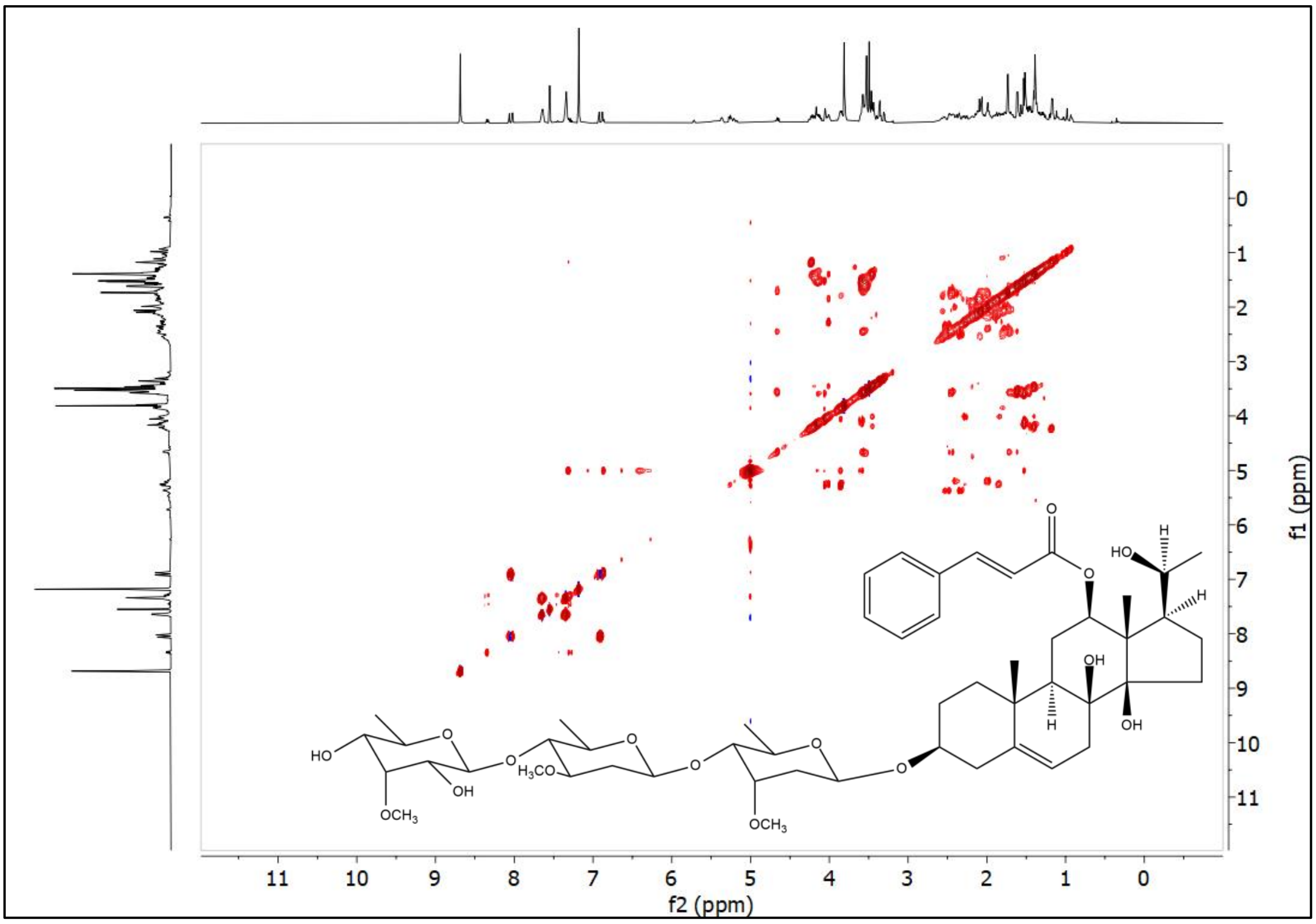

Figure S110. ROESY spectrum (400 x 400 MHz, C_5D_5N) of compound 11

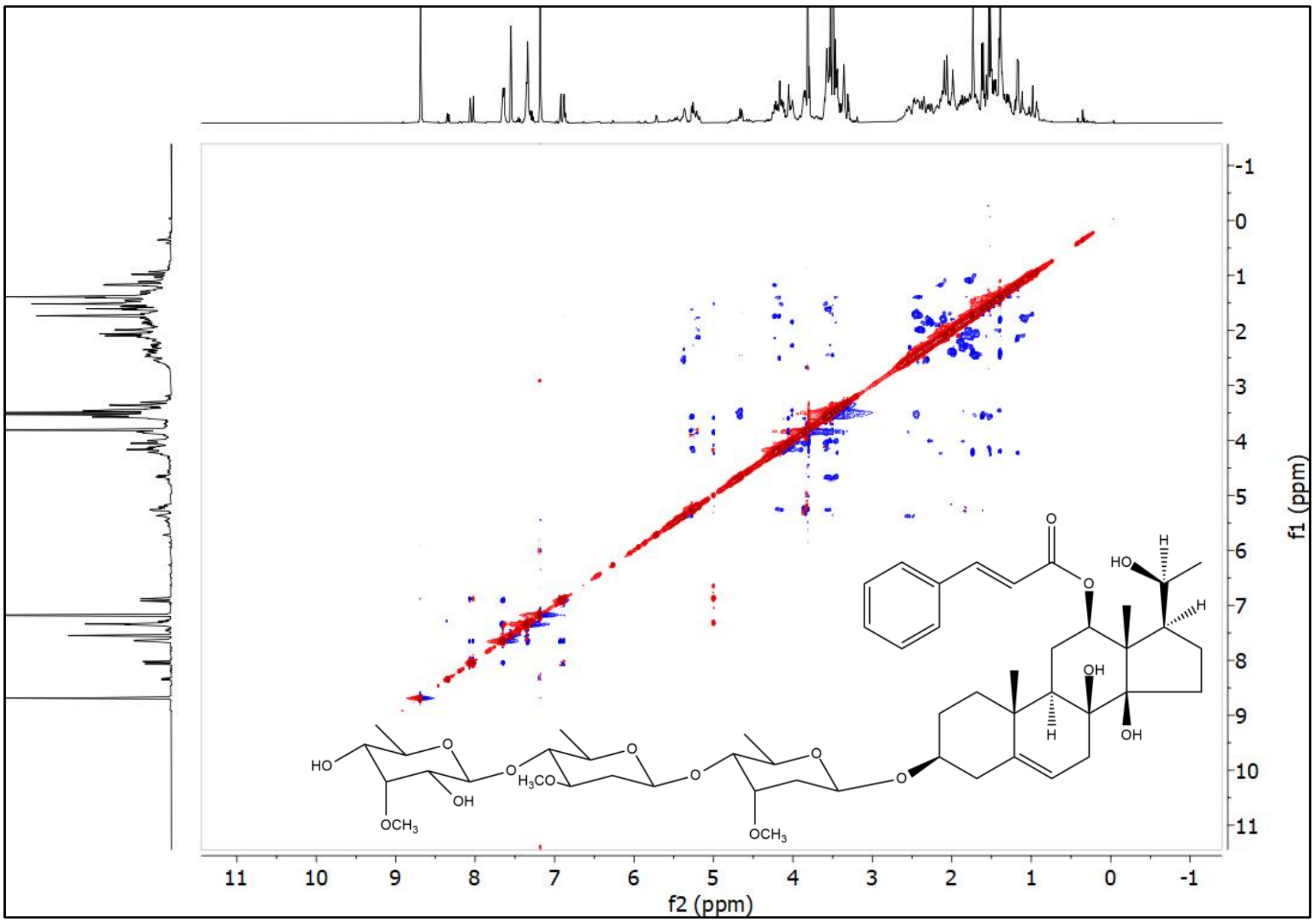

Figure S111. ATR-Infrared spectrum of compound 12.

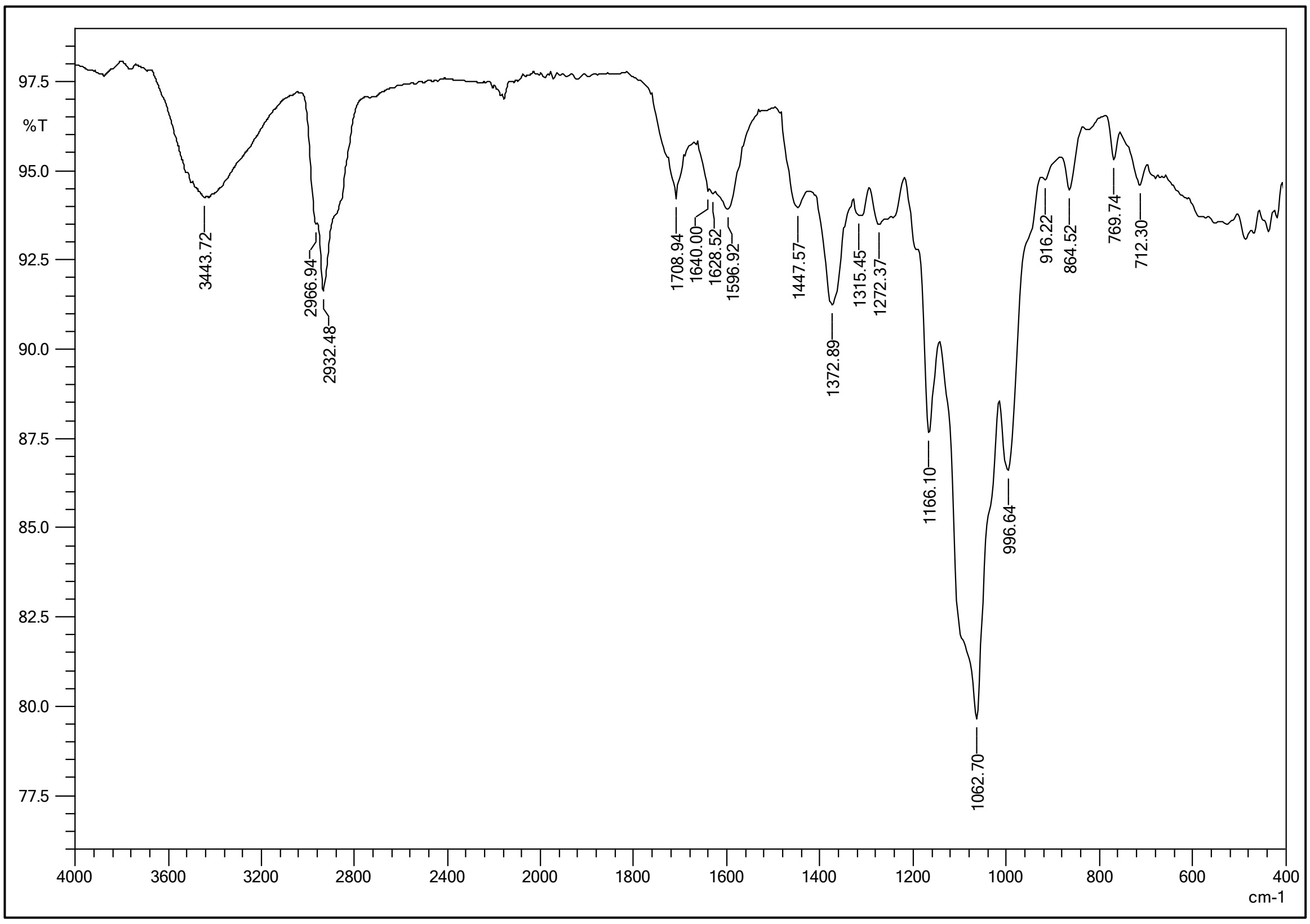

Figure S112. HR-ESI-MS mass spectrum of compound 12.

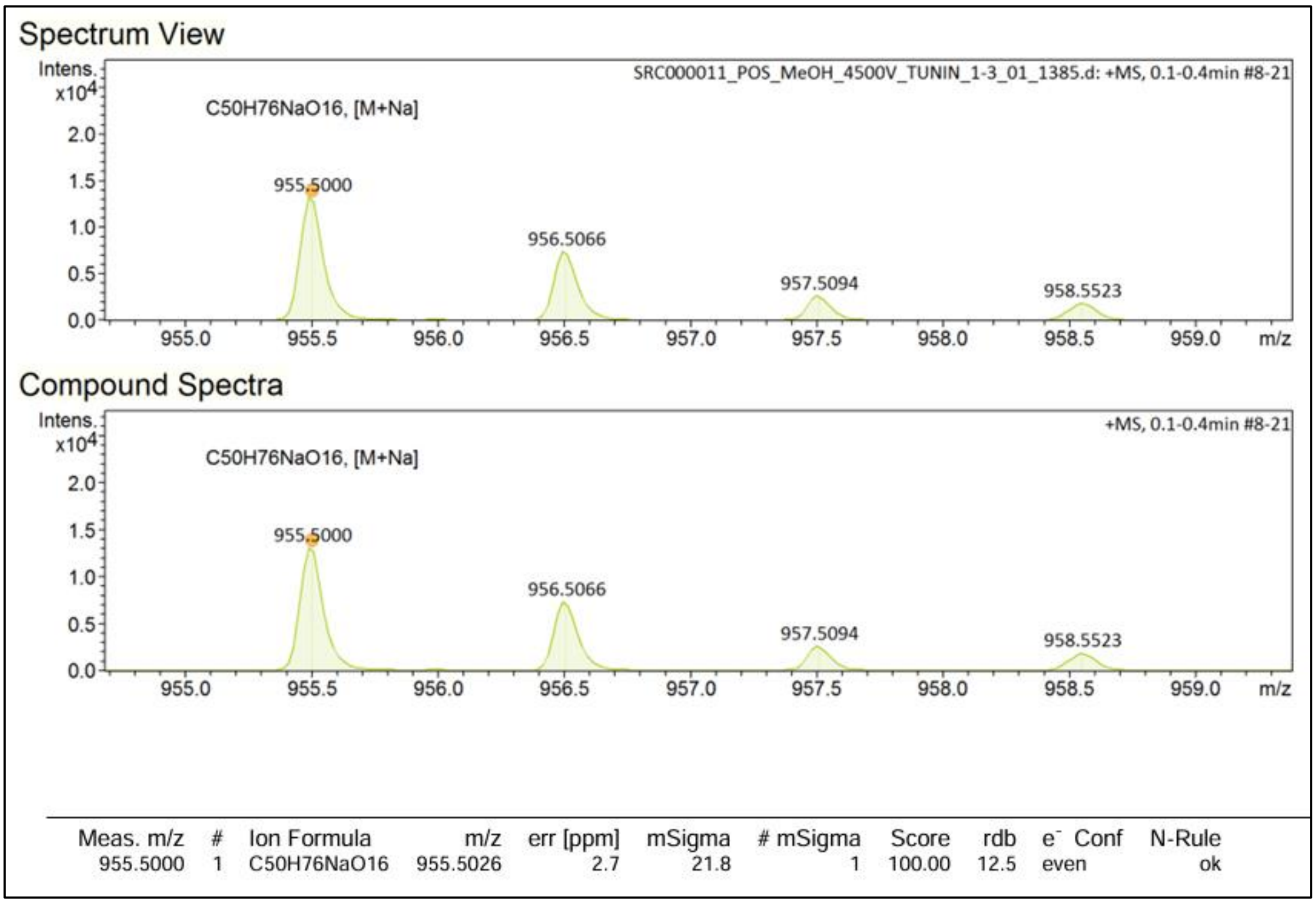

Figure S113. ^{1}H NMR spectrum (400 MHz, C_5D_5N) of compound 12

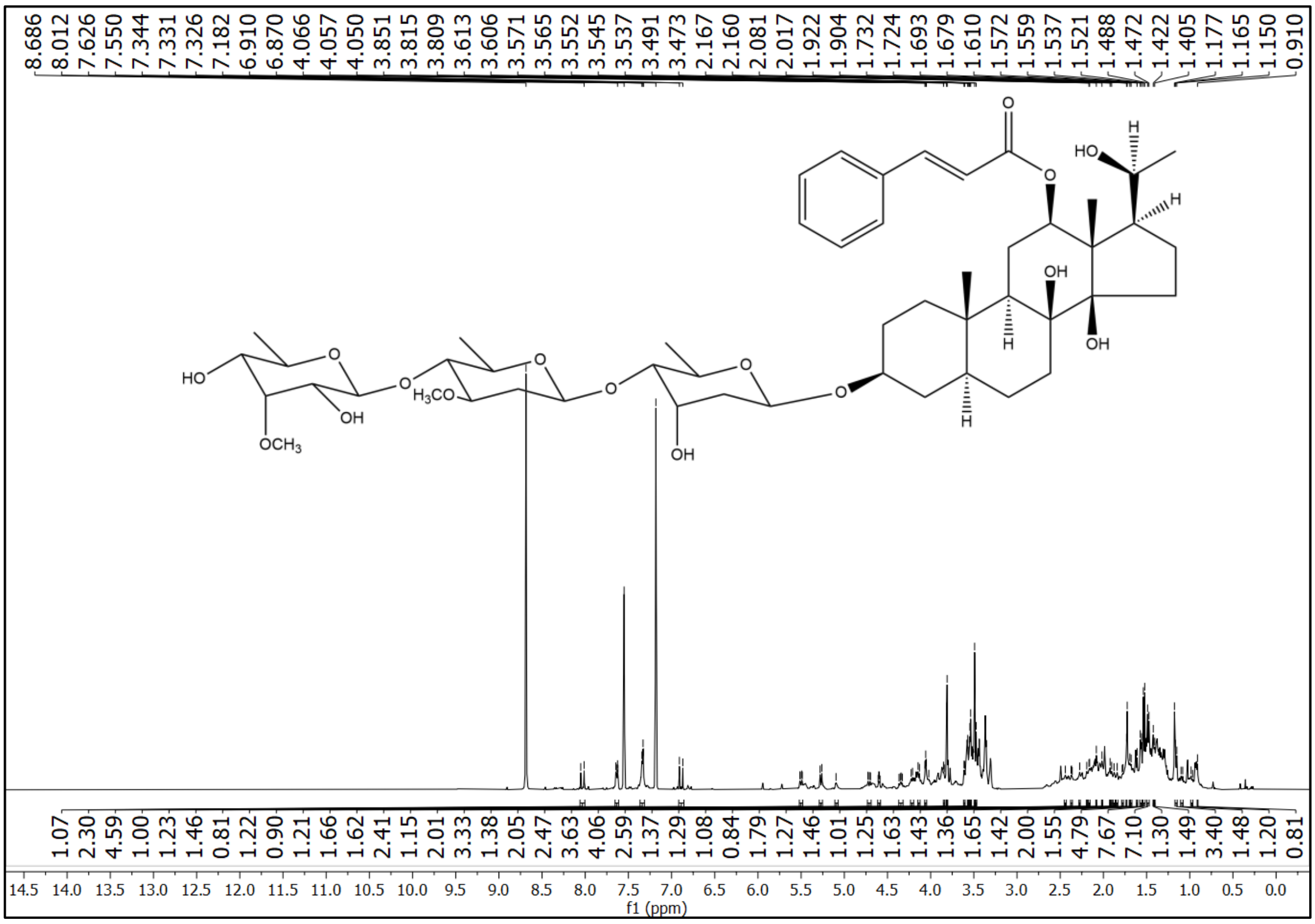

Figure S114. ^{13}C-BB NMR spectrum (100 MHz, C_5D_5N) of compound 12

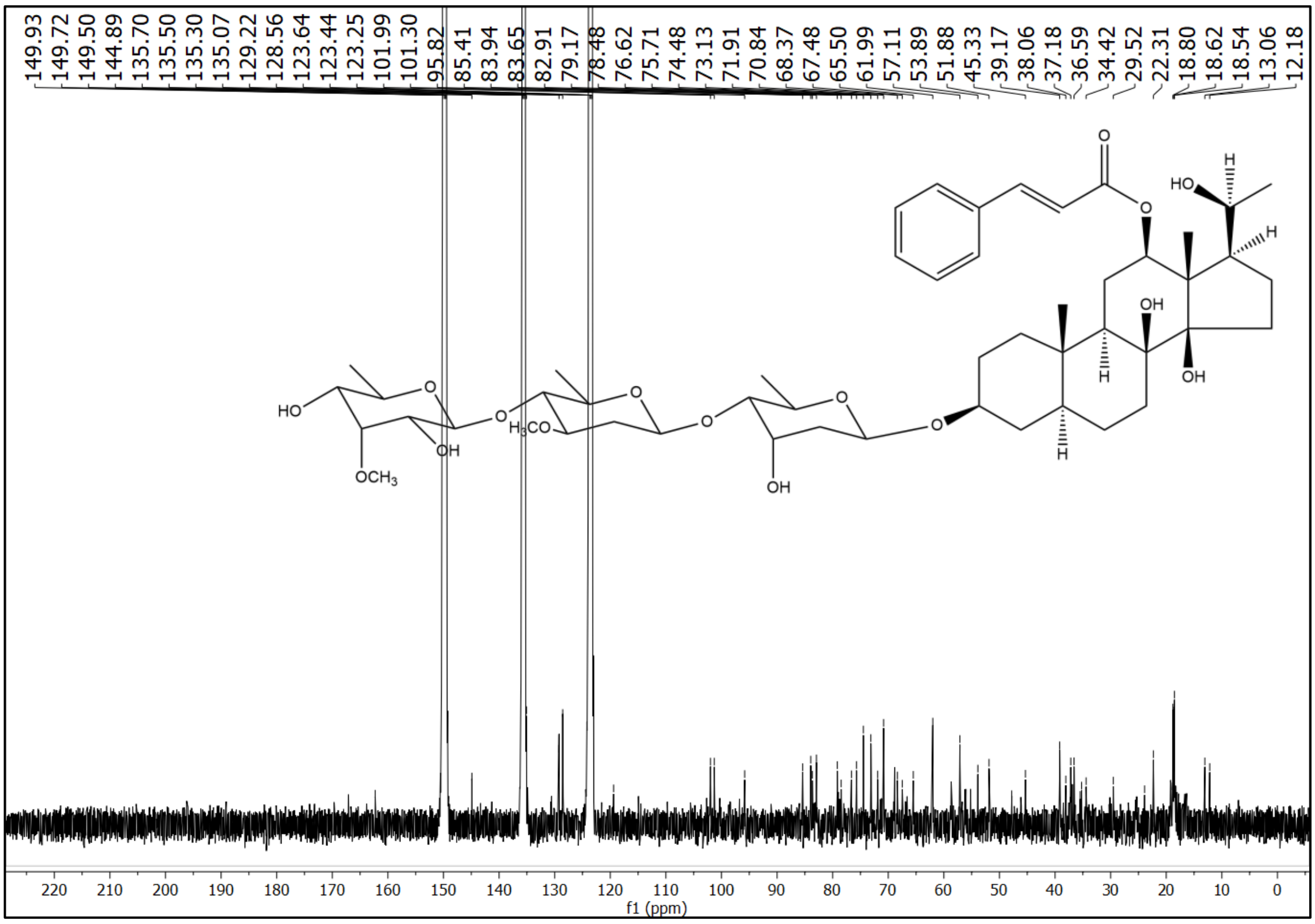

Figure S115. ^{13}C-DEPT135 NMR spectrum (100 MHz, C_5D_5N) of compound 12

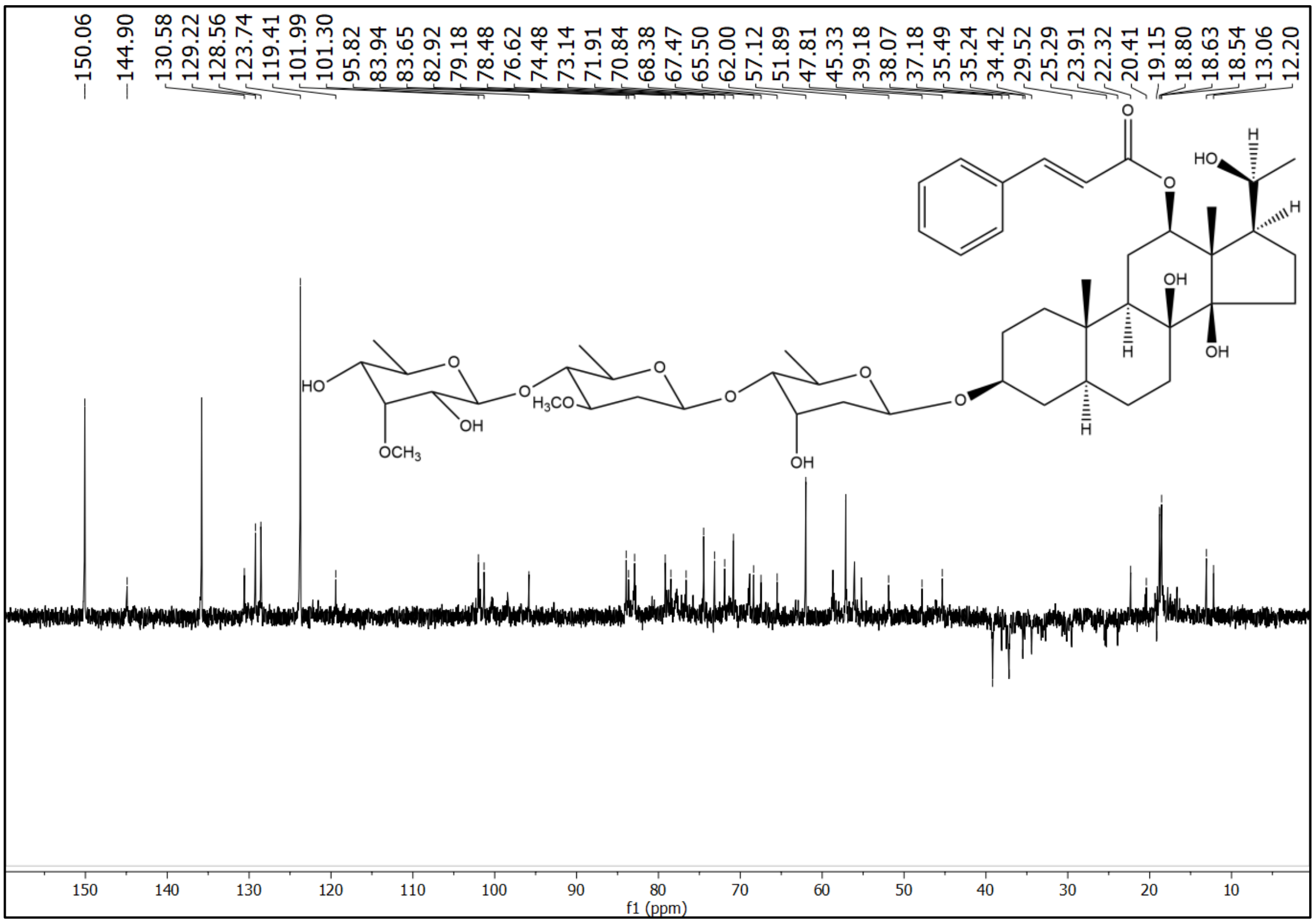

Figure S116. HSQC spectrum (400 x 100 MHz, C_5D_5N) of compound 12

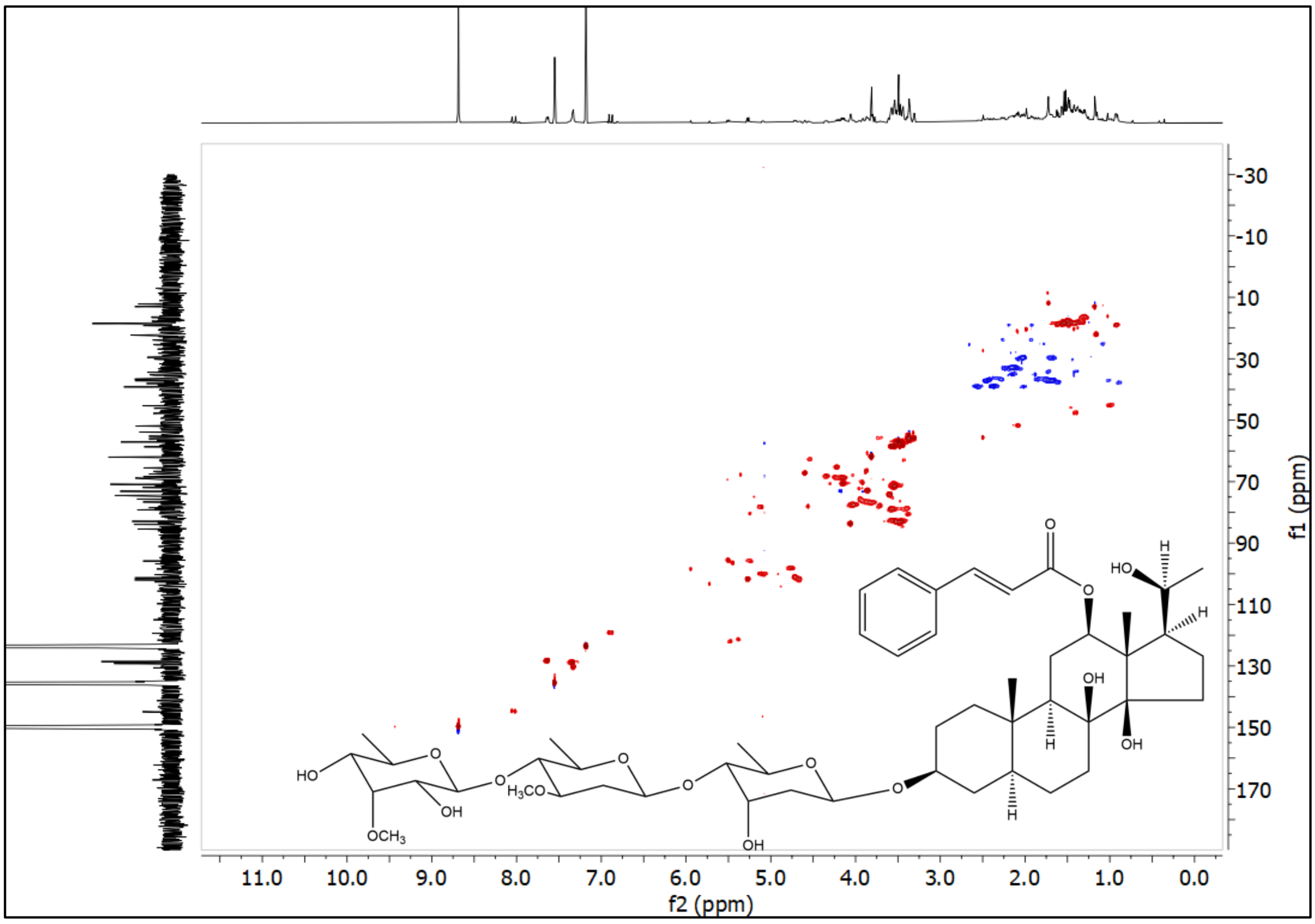

Figure S117. HMBC spectrum (400 x 100 MHz, C_5D_5N) of compound 12

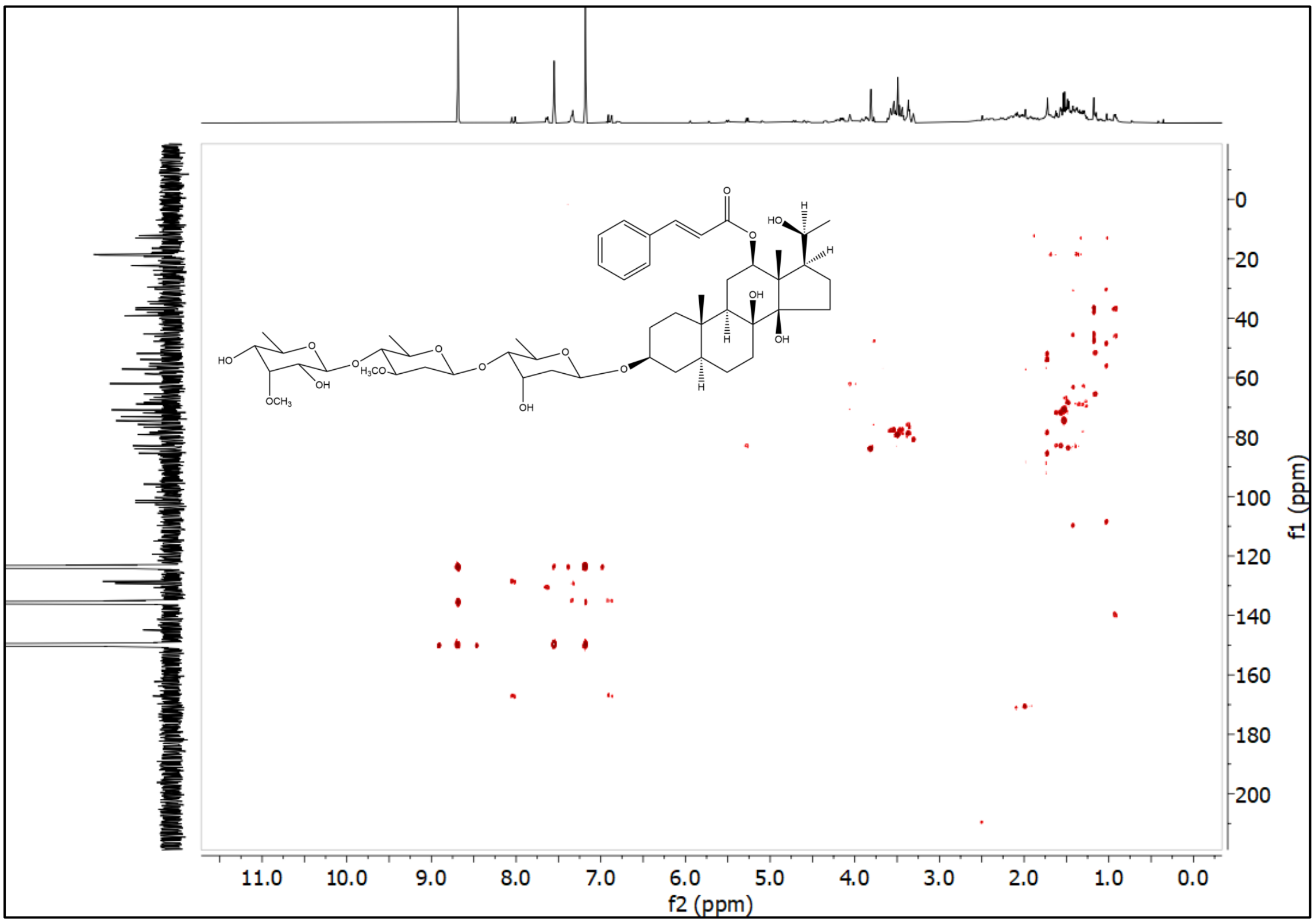

Figure S118. COSY spectrum (400 x 400 MHz, C_5D_5N) of compound 12

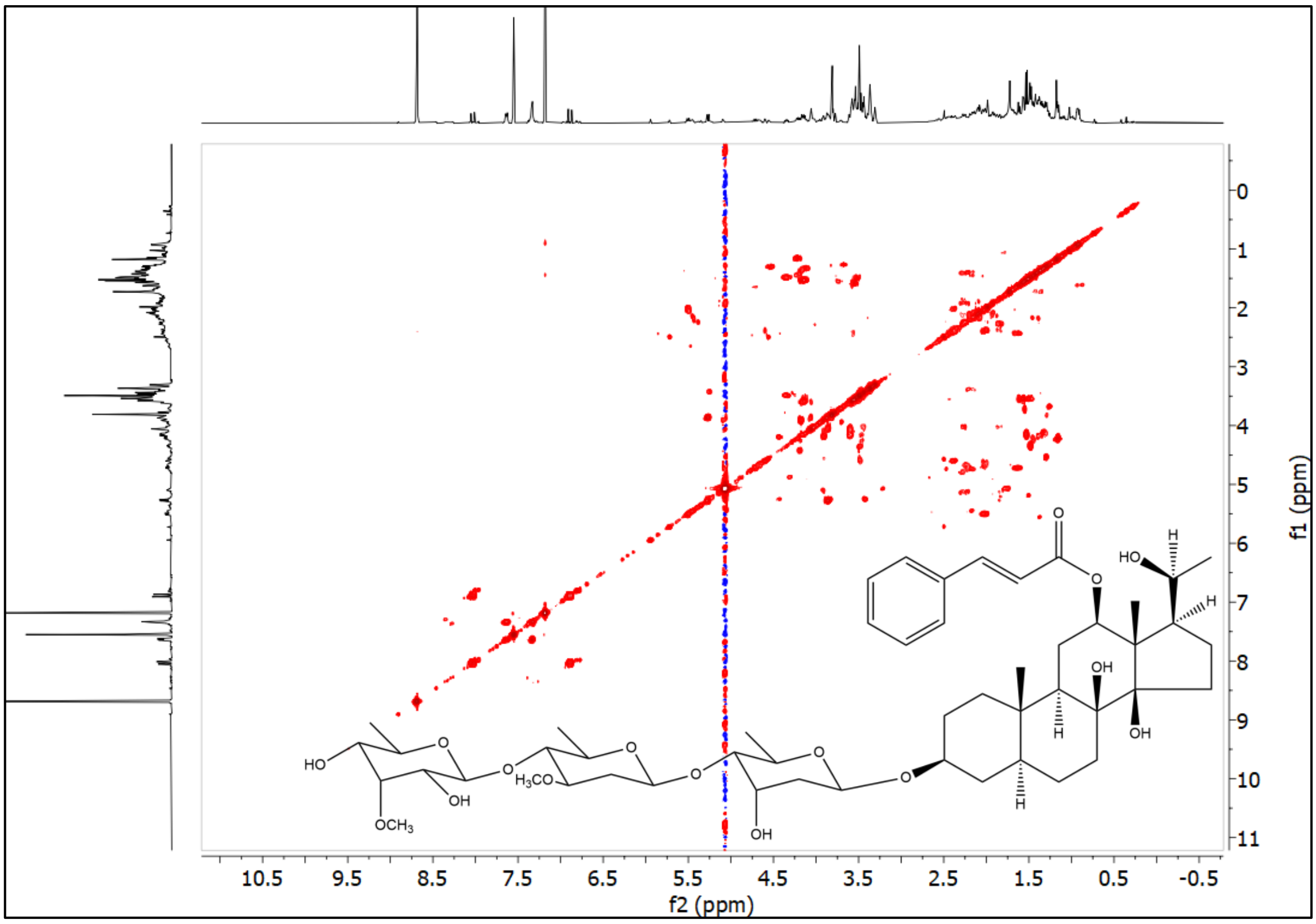

Figure S119. TOCSY spectrum (400 x 400 MHz, C_5D_5N) of compound 12.

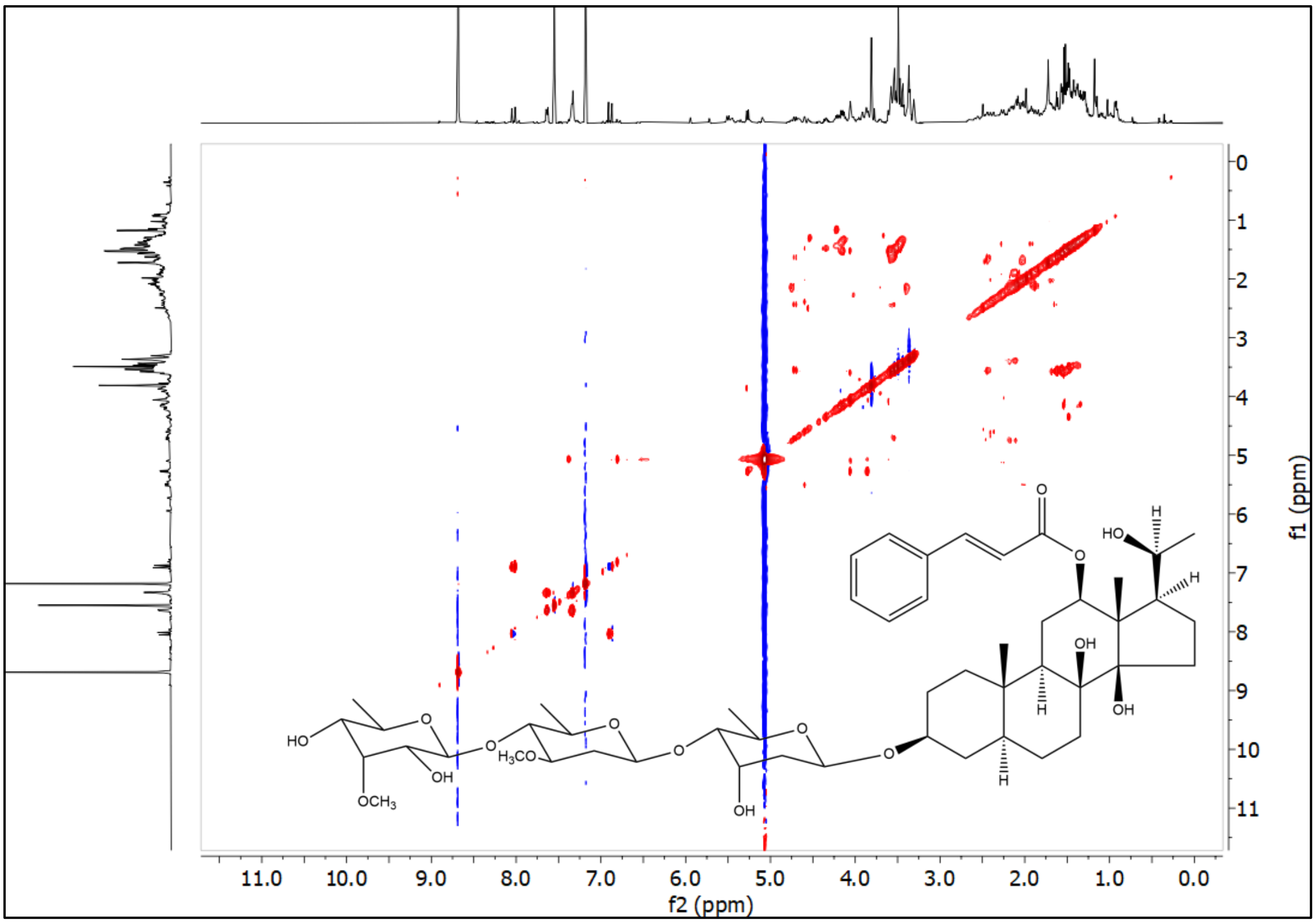

Figure S120. NOESY spectrum (400 x 400 MHz, C_5D_5N) of compound 12.

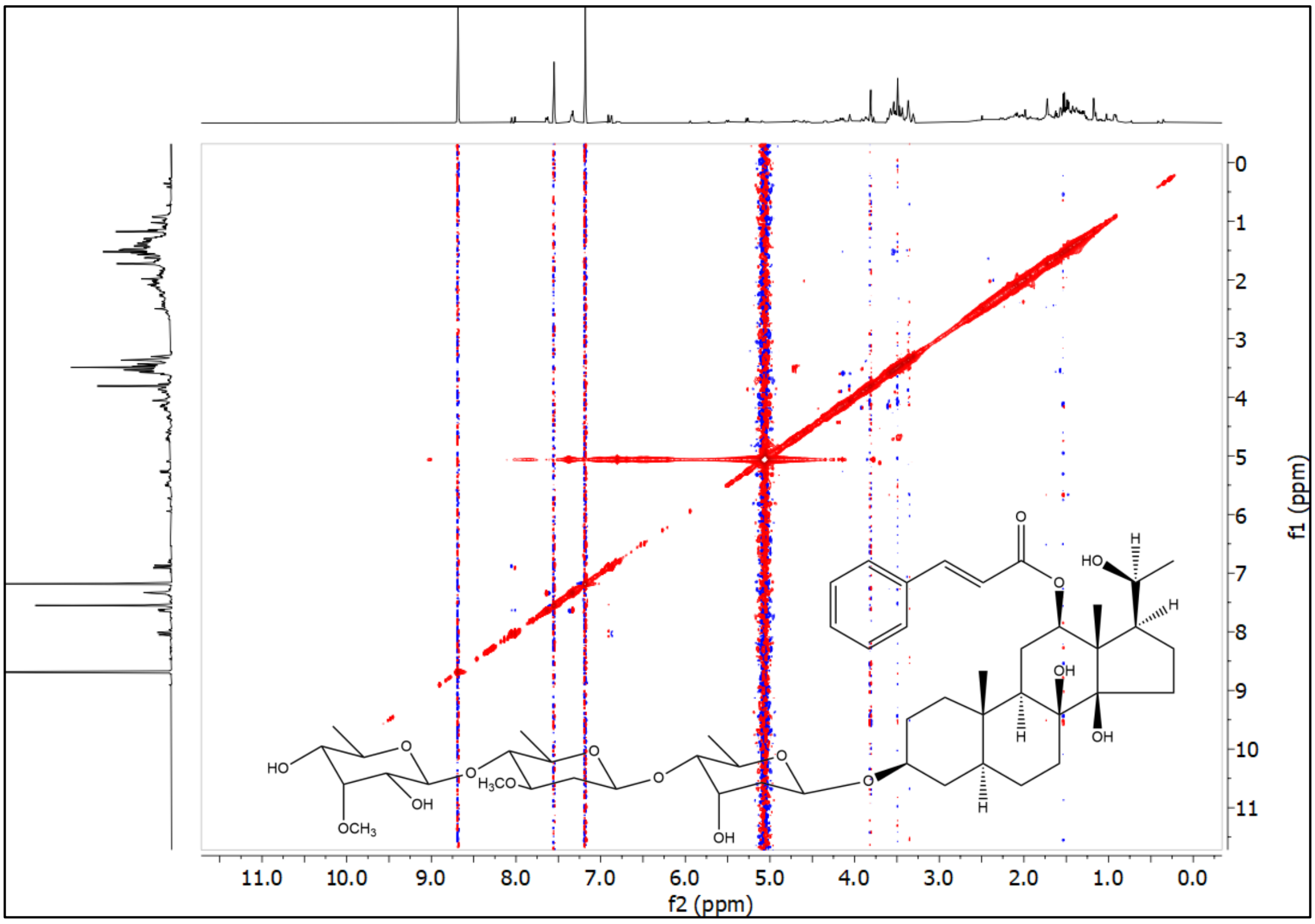

Figure S121. ATR-Infrared spectrum of compound 13.

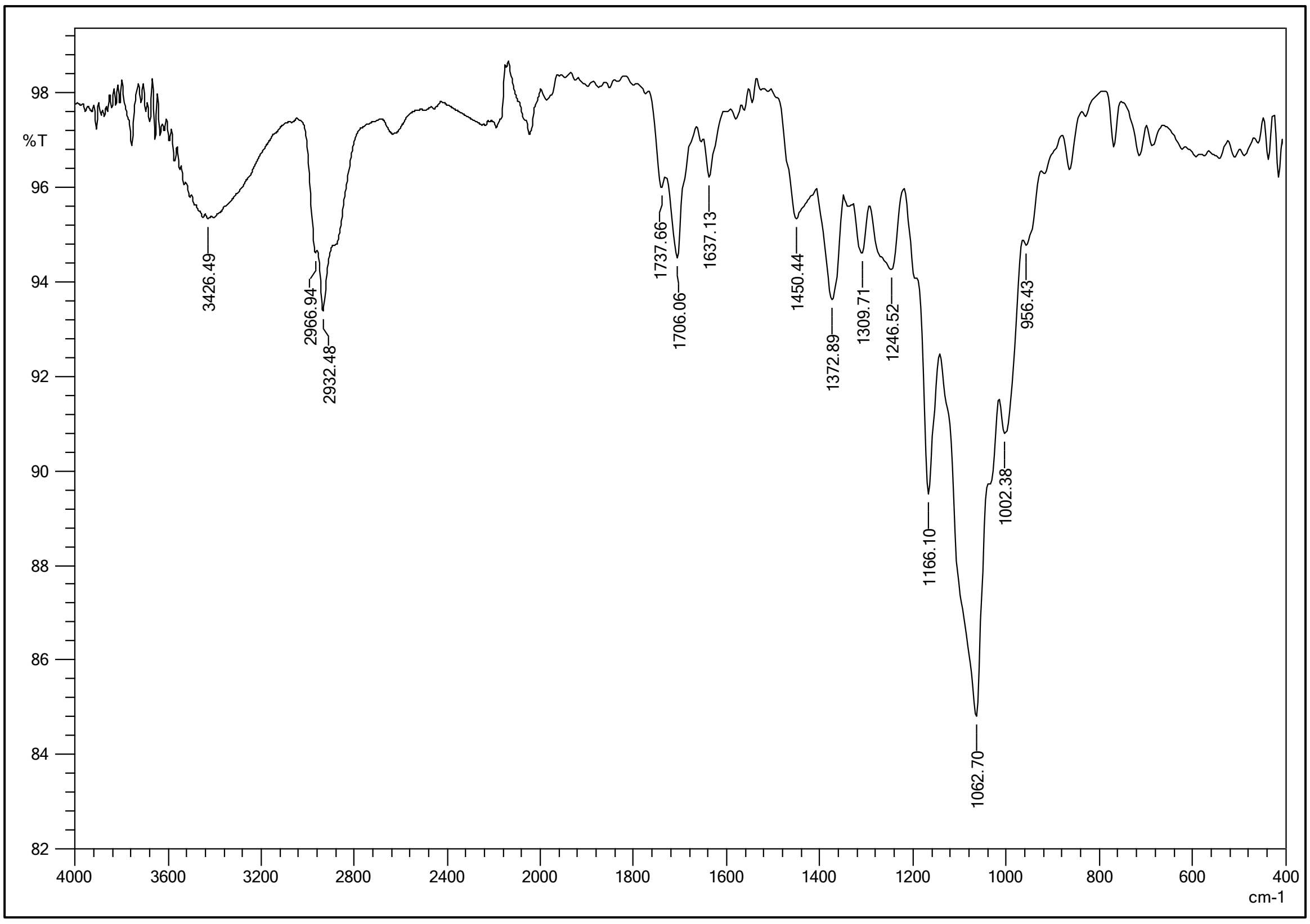

Figure S122. HR-ESI-MS mass spectrum of compound 13.

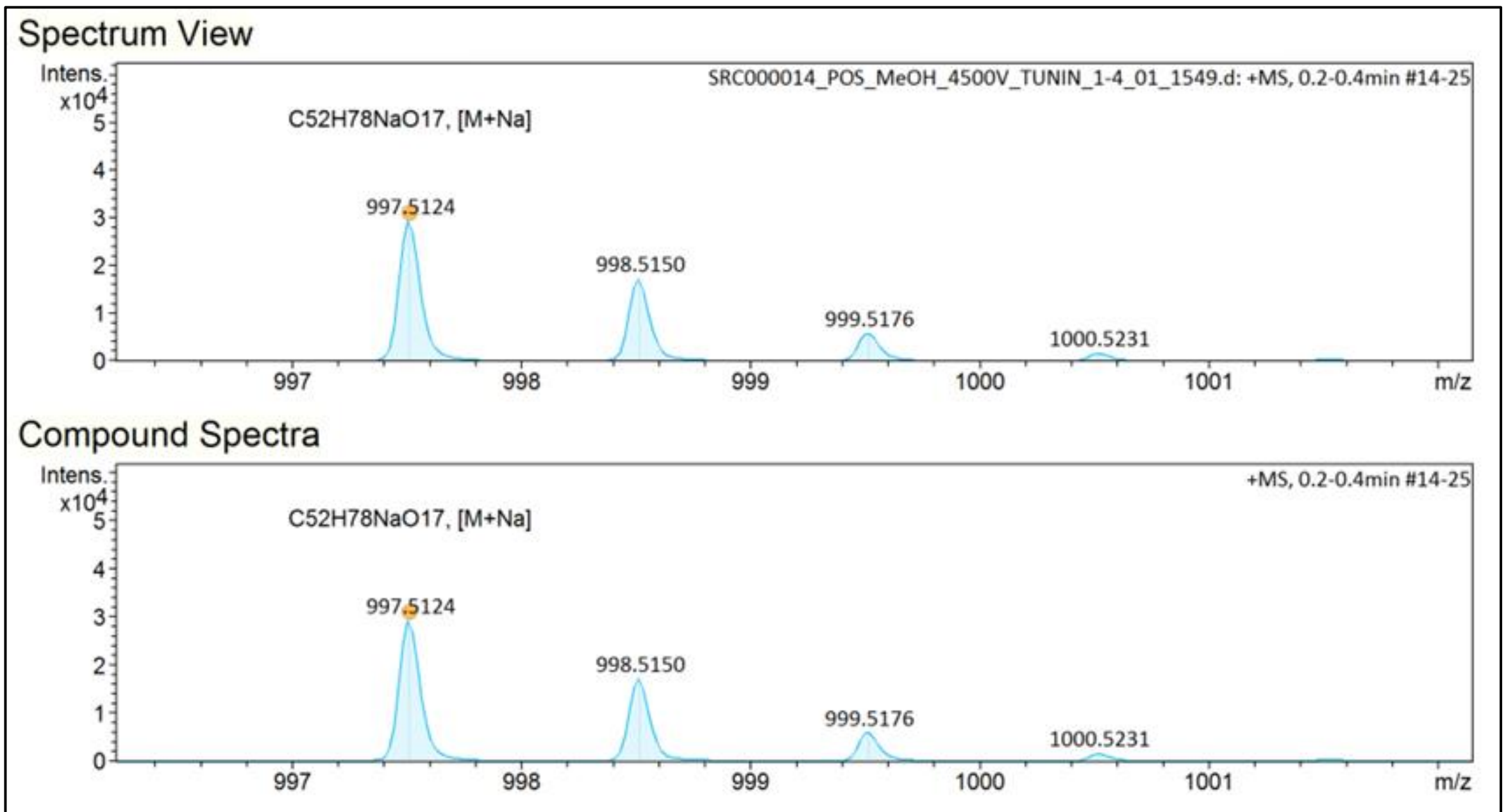

Meas. m/z	#	Ion Formula	m/z	err [ppm]	mSigma	# mSigma	Score	rdb	e⁻ Conf	N-Rule
997.5124	1	C52H78NaO17	997.5131	0.7	4.7	1	100.00	13.5	even	ok

Figure S123. ^{1}H NMR spectrum (5400 MHz, C_5D_5N) of compound 13

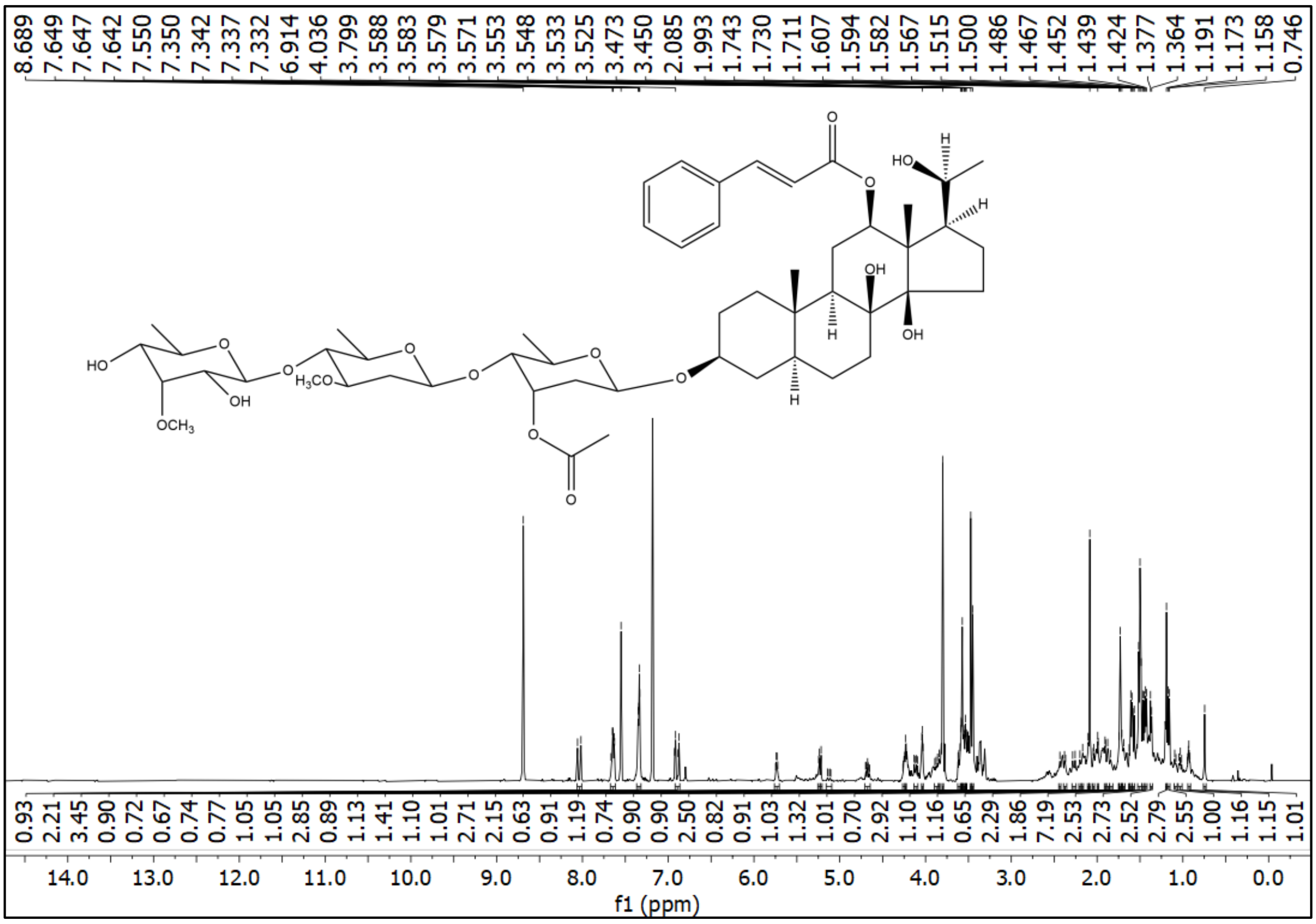

Figure S124. ^{13}C-BB NMR spectrum (100 MHz, C_5D_5N) of compound 13

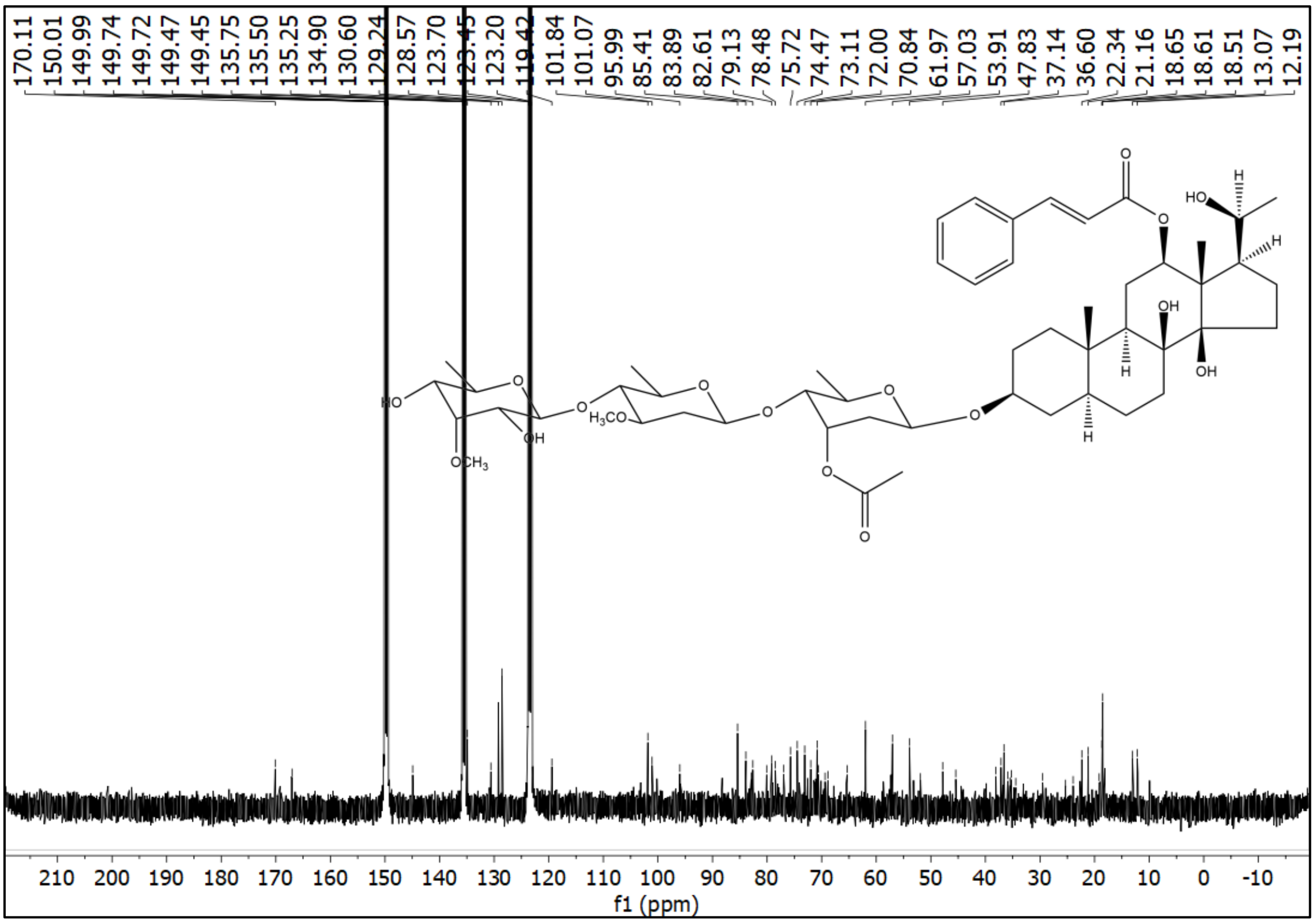

Figure S125. ^{13}C-DEPT135 NMR spectrum (100 MHz, C_5D_5N) of compound 13

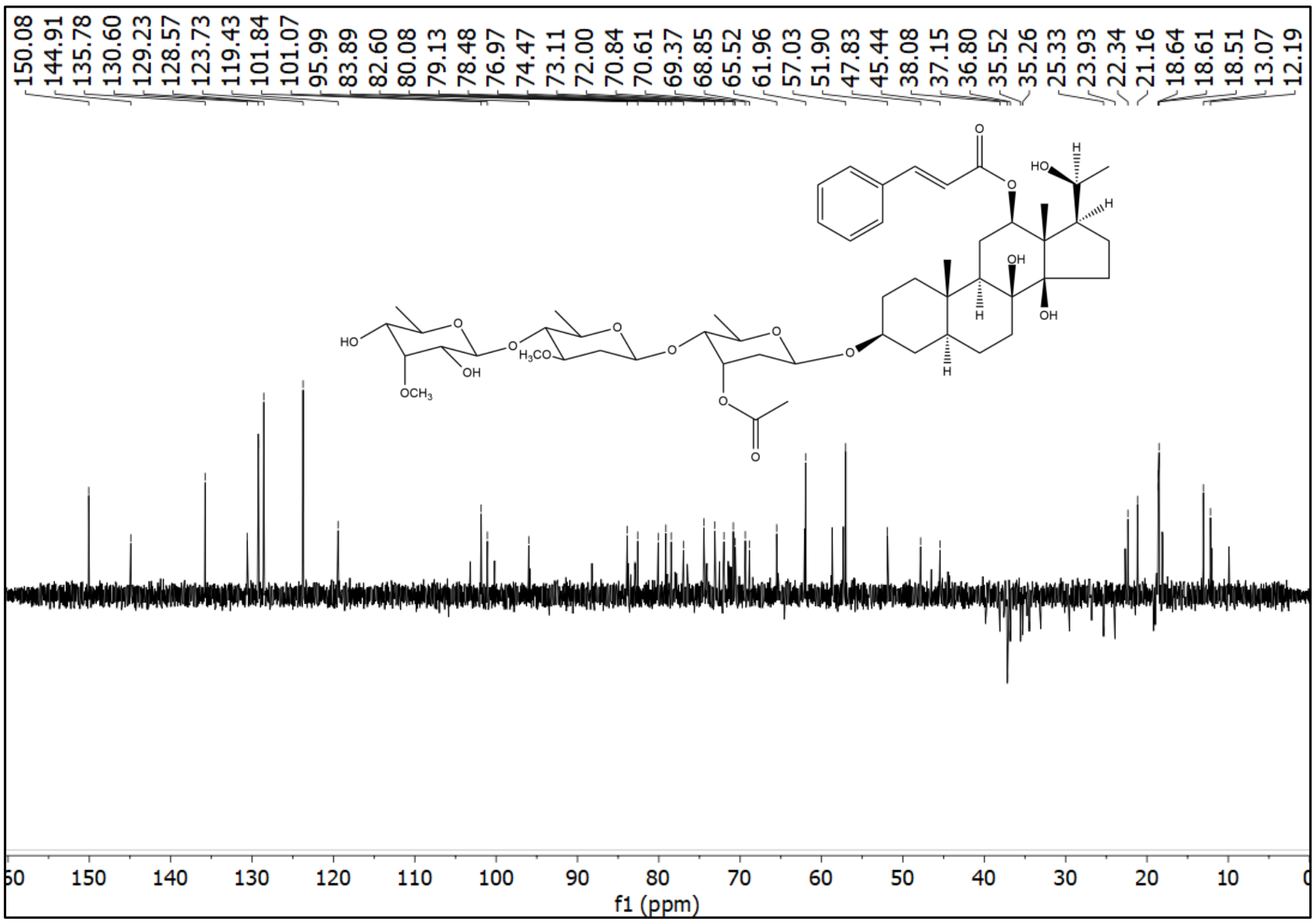

Figure S126. HSQC spectrum (400 x 100 MHz, C_5D_5N) of compound 13

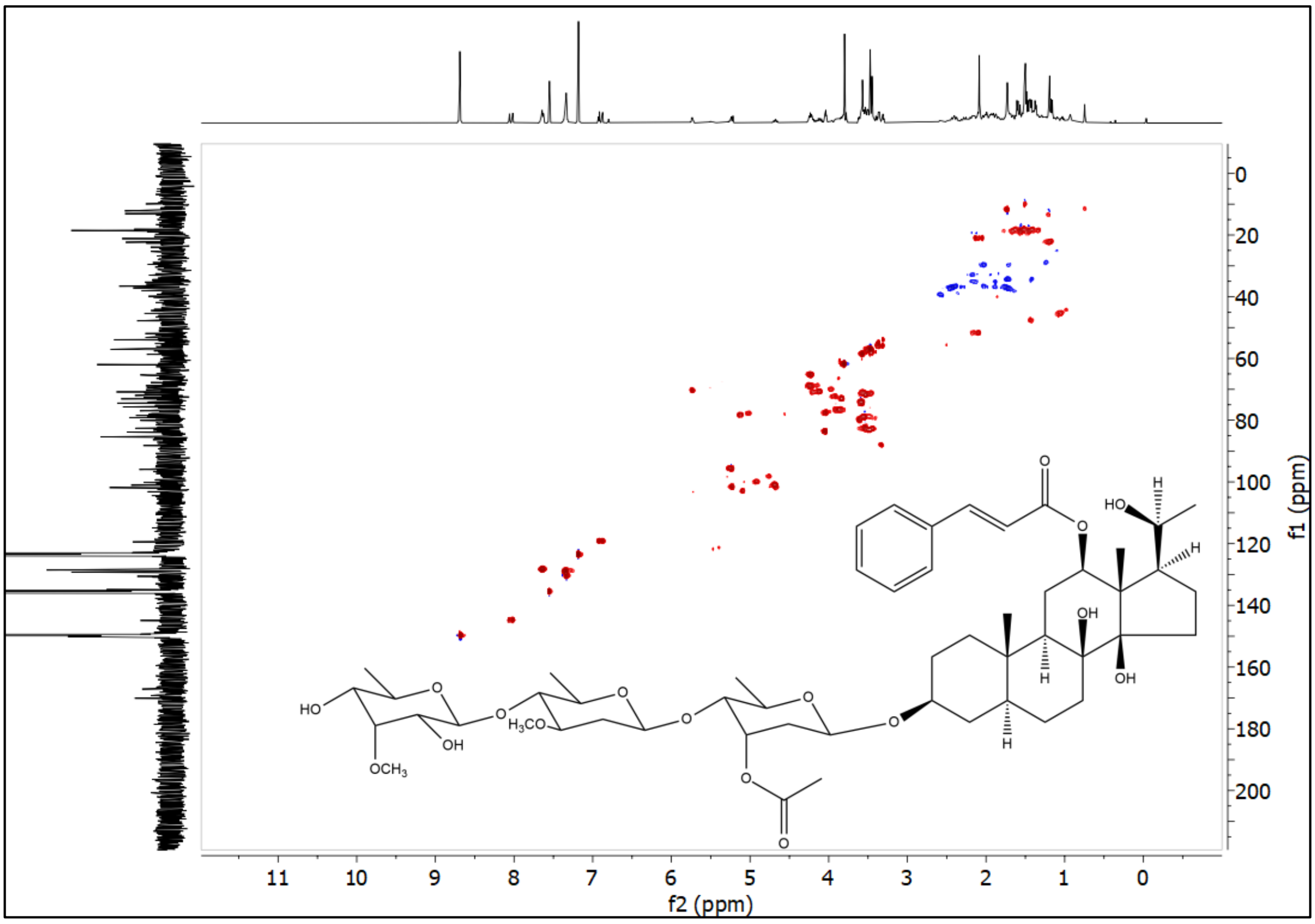

Figure S127. HMBC spectrum (400 x 100 MHz, C_5D_5N) of compound 13

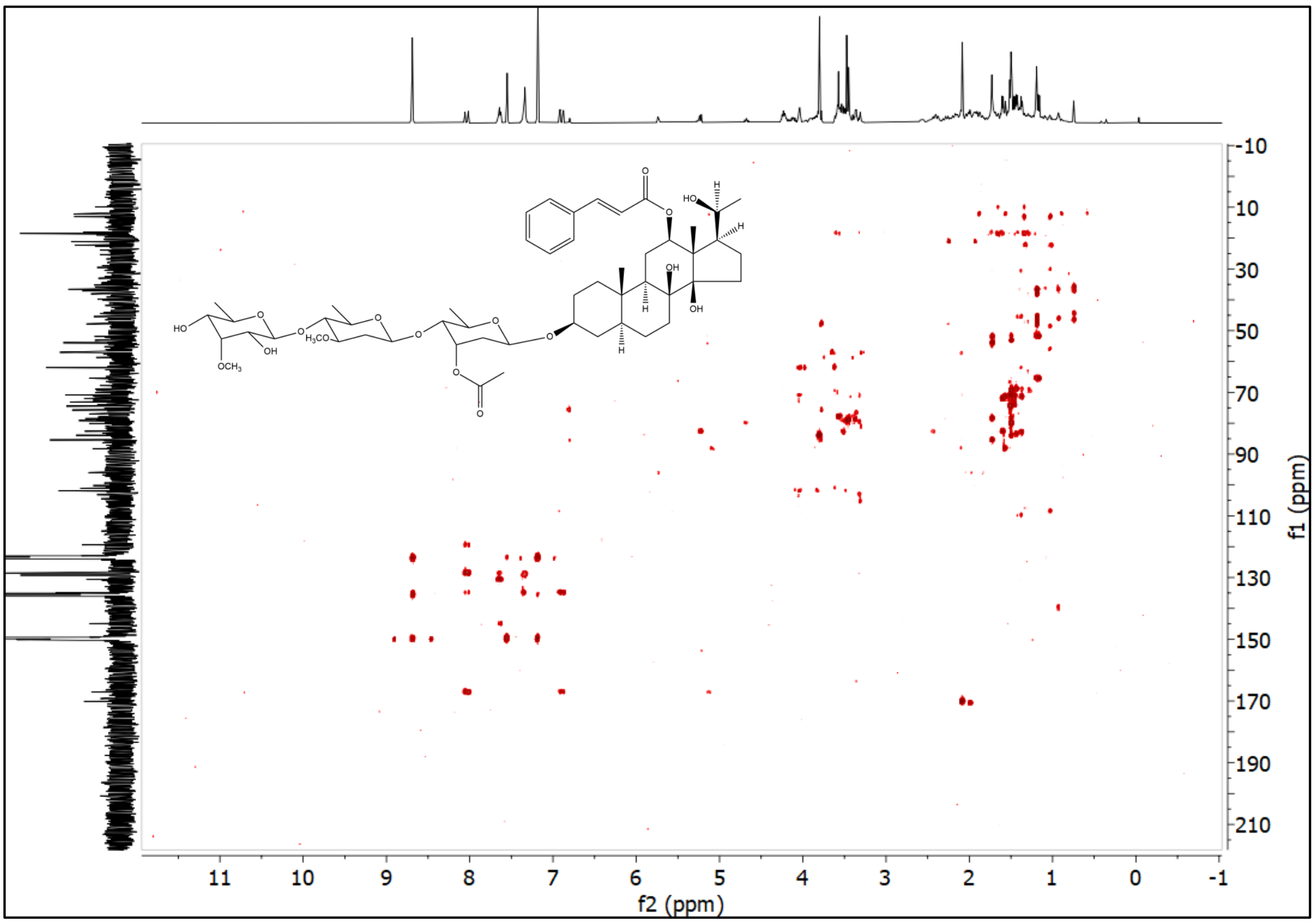

Figure S128. COSY spectrum (400 x 400 MHz, C_5D_5N) of compound 13.

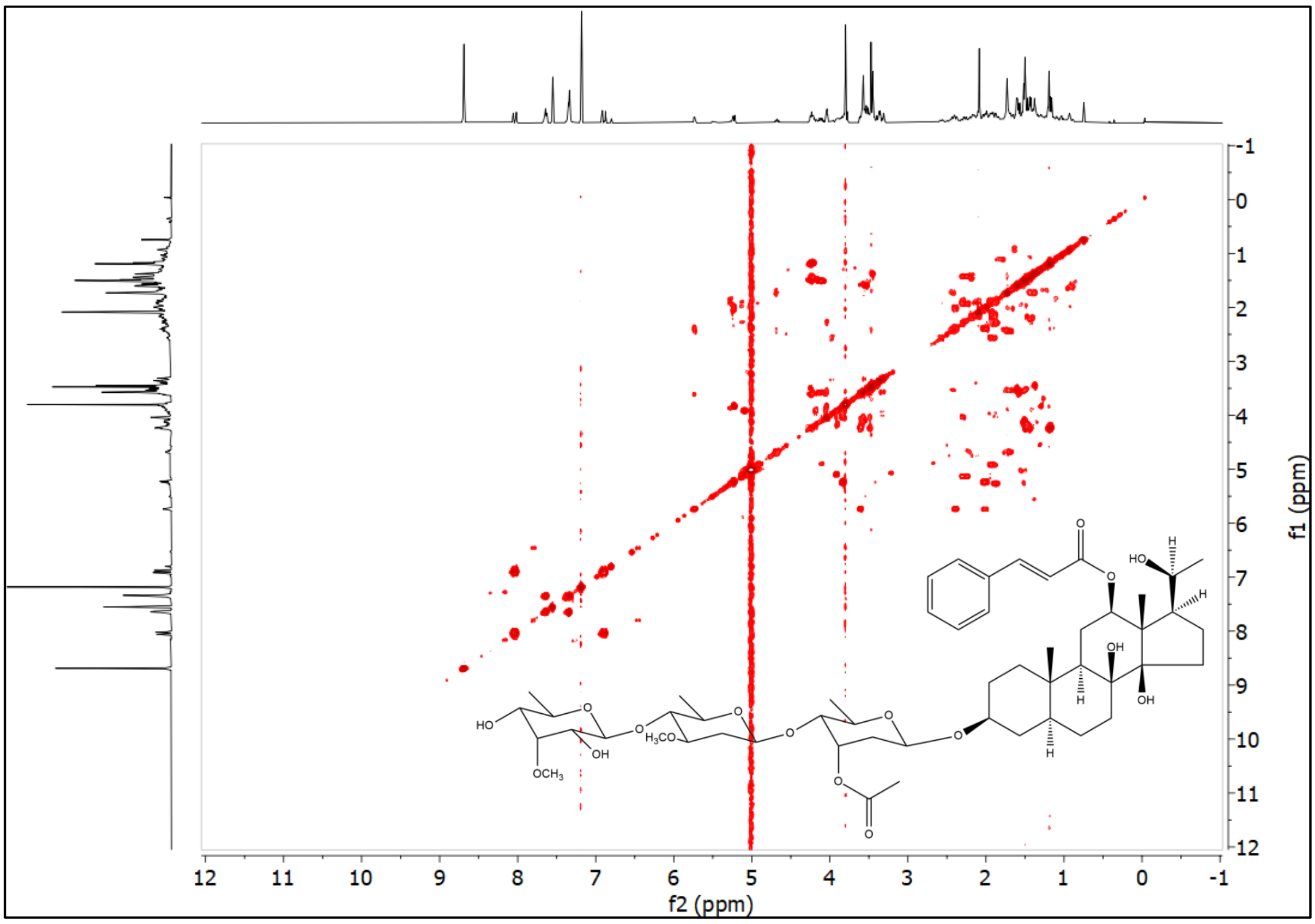

Figure S129. TOCSY spectrum (400 x 400 MHz, C_5D_5N) of compound 13.

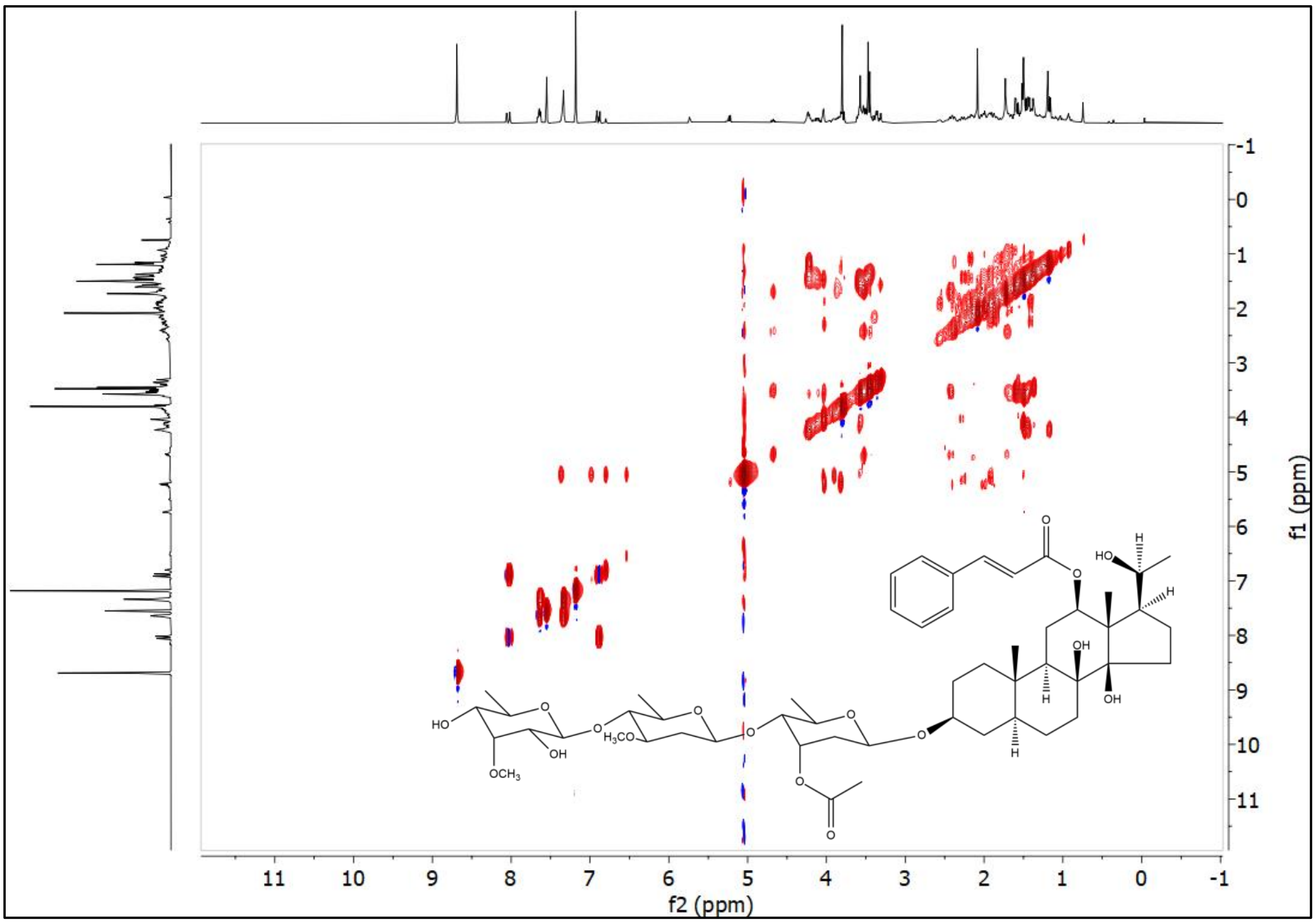

Figure S130. ROESY spectrum (400 x 400 MHz, C_5D_5N) of compound 13.

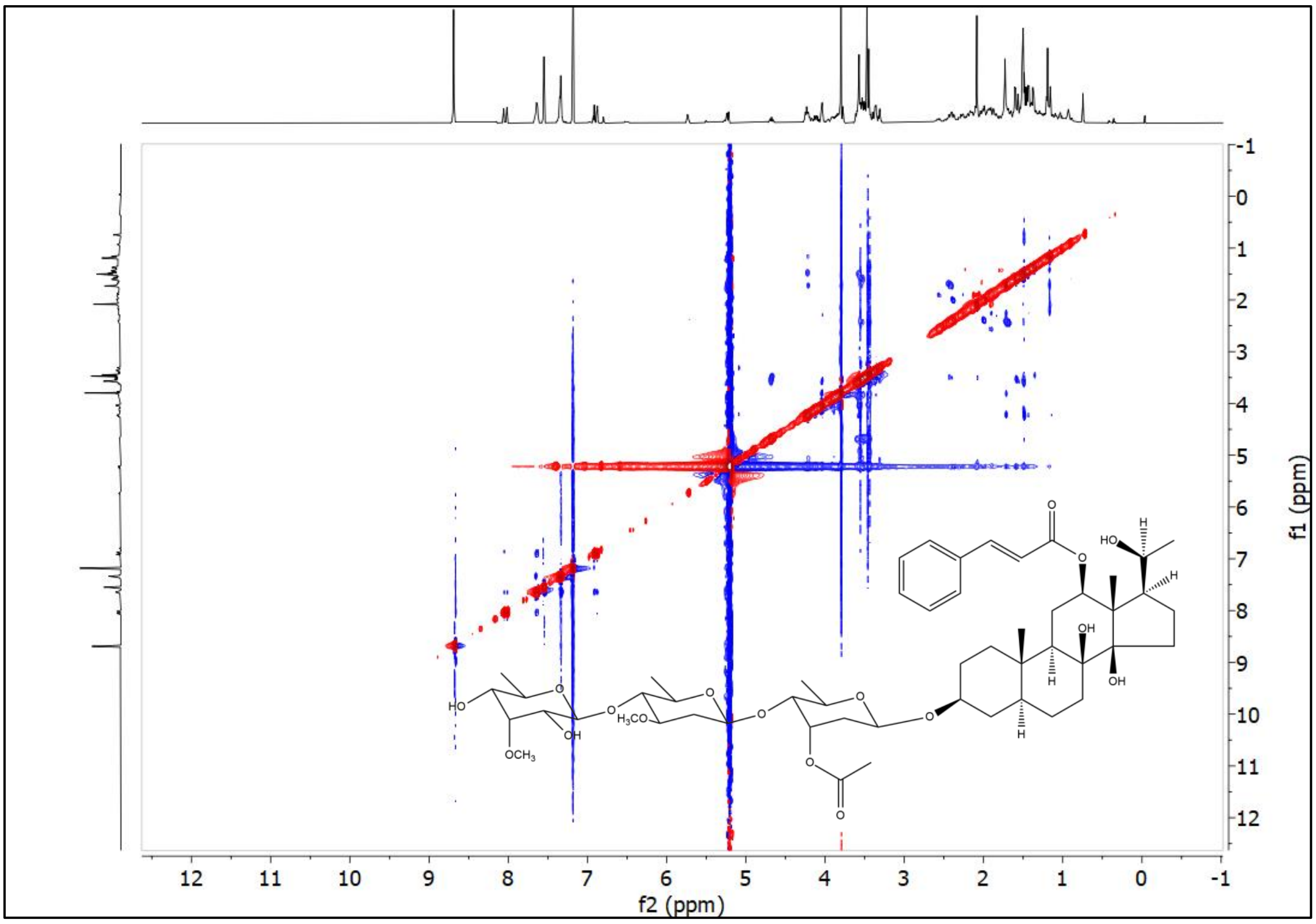

Figure S131. ATR-Infrared spectrum of compound 14.

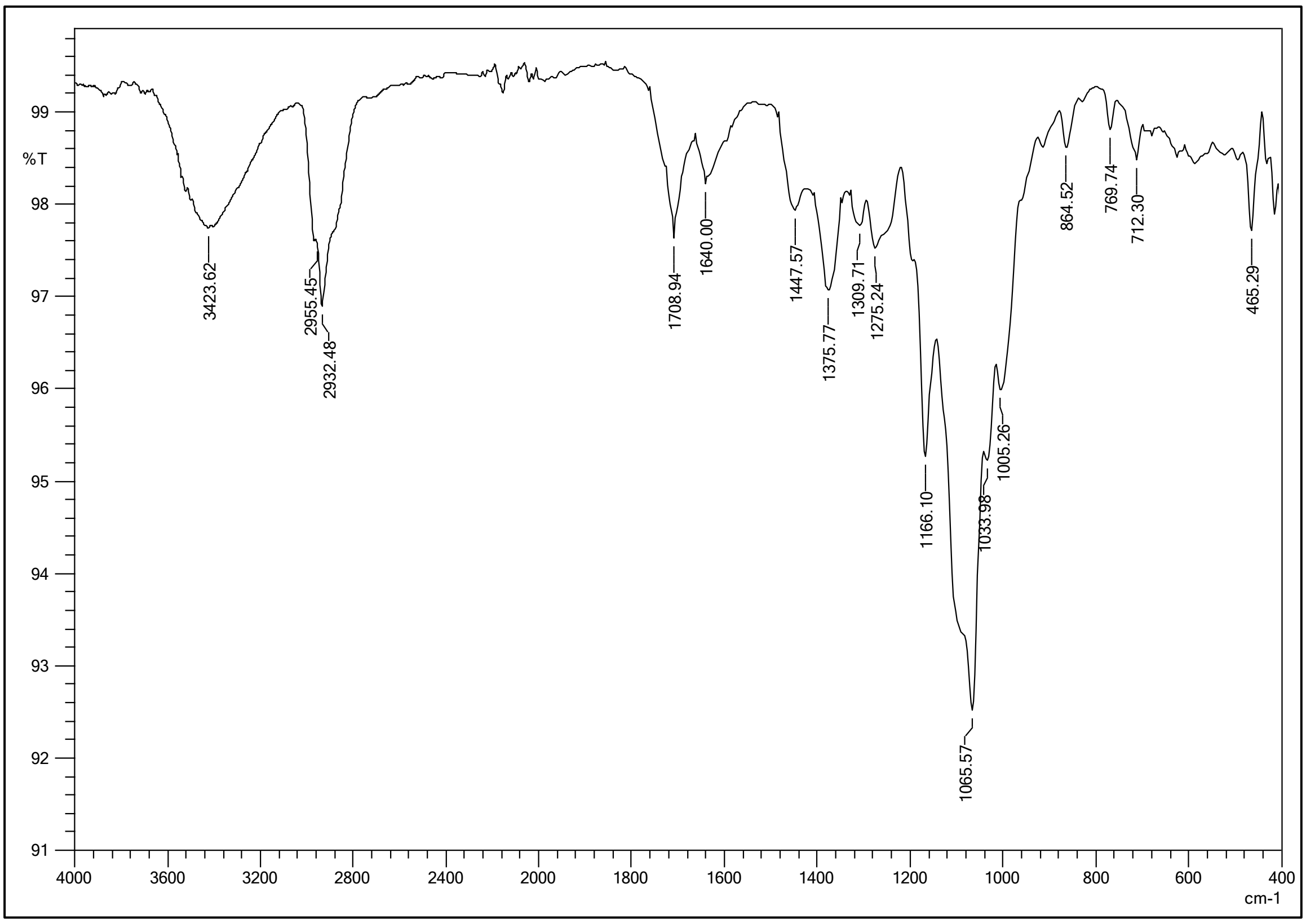

Figure S132. HR-ESI-MS mass spectrum of compound 14.

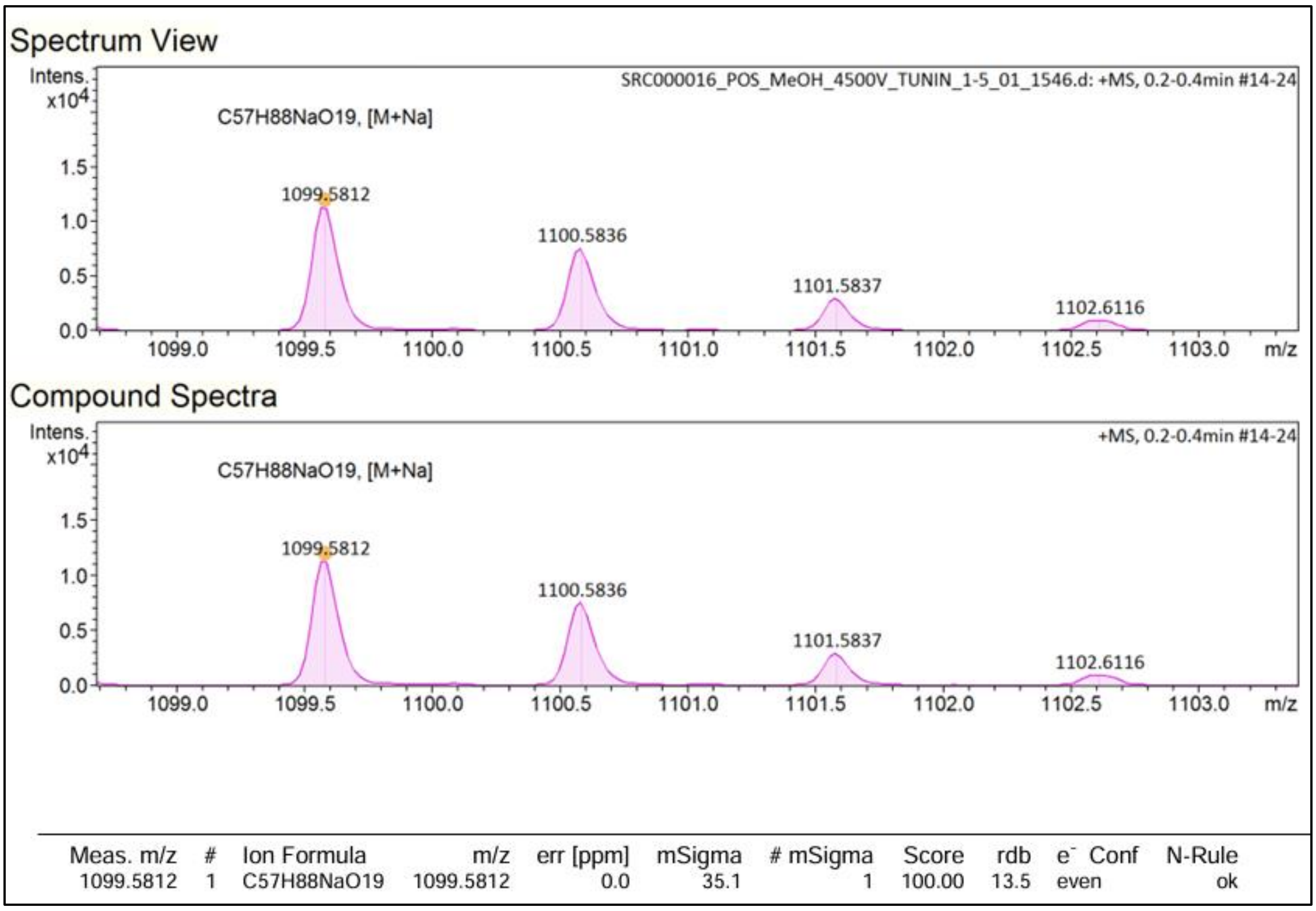

Figure S133. ^{1}H NMR spectrum (400 MHz, C_5D_5N) of compound 14

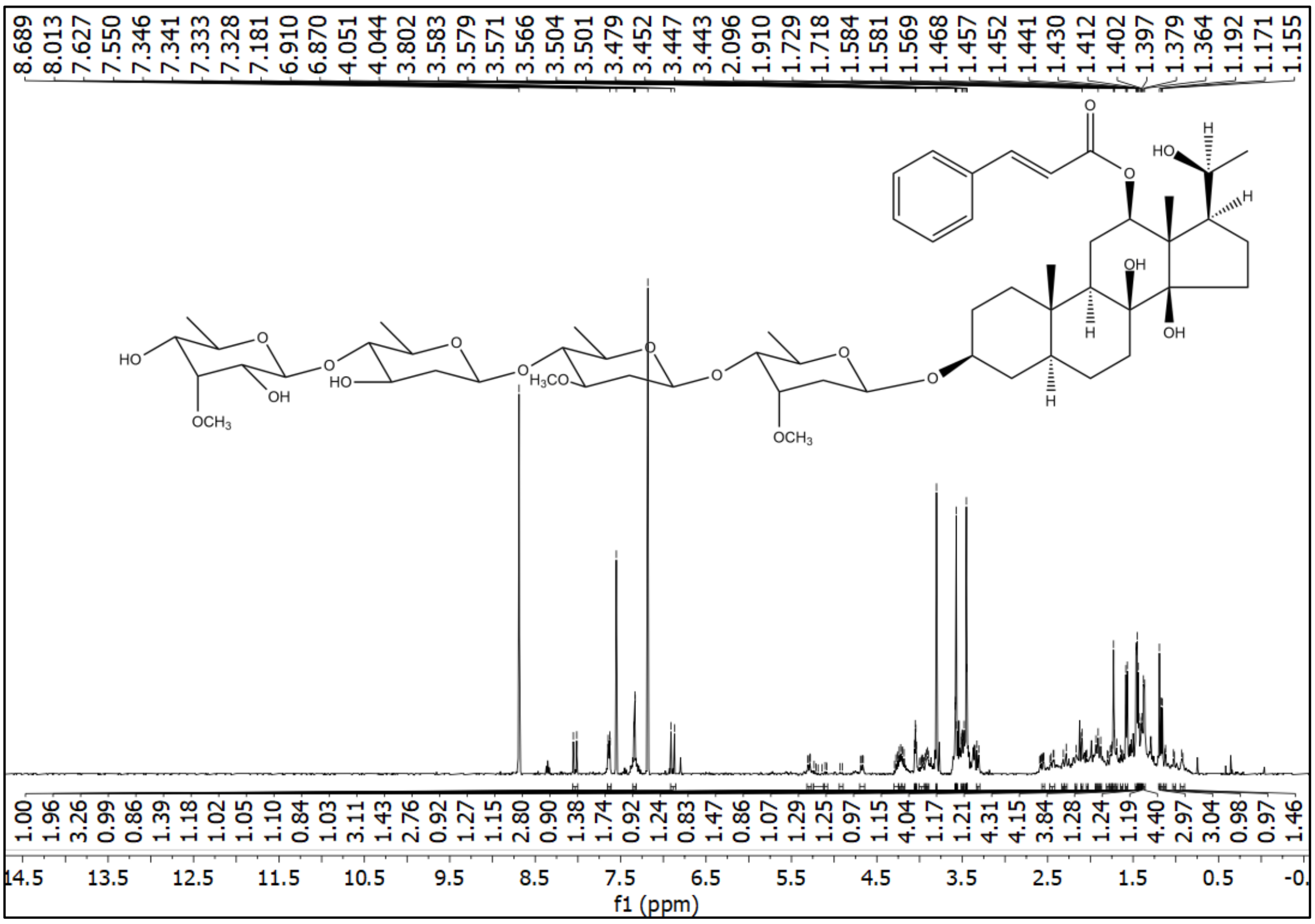

Figure S134. ^{13}C-BB NMR spectrum (100 MHz, C_5D_5N) of compound 14

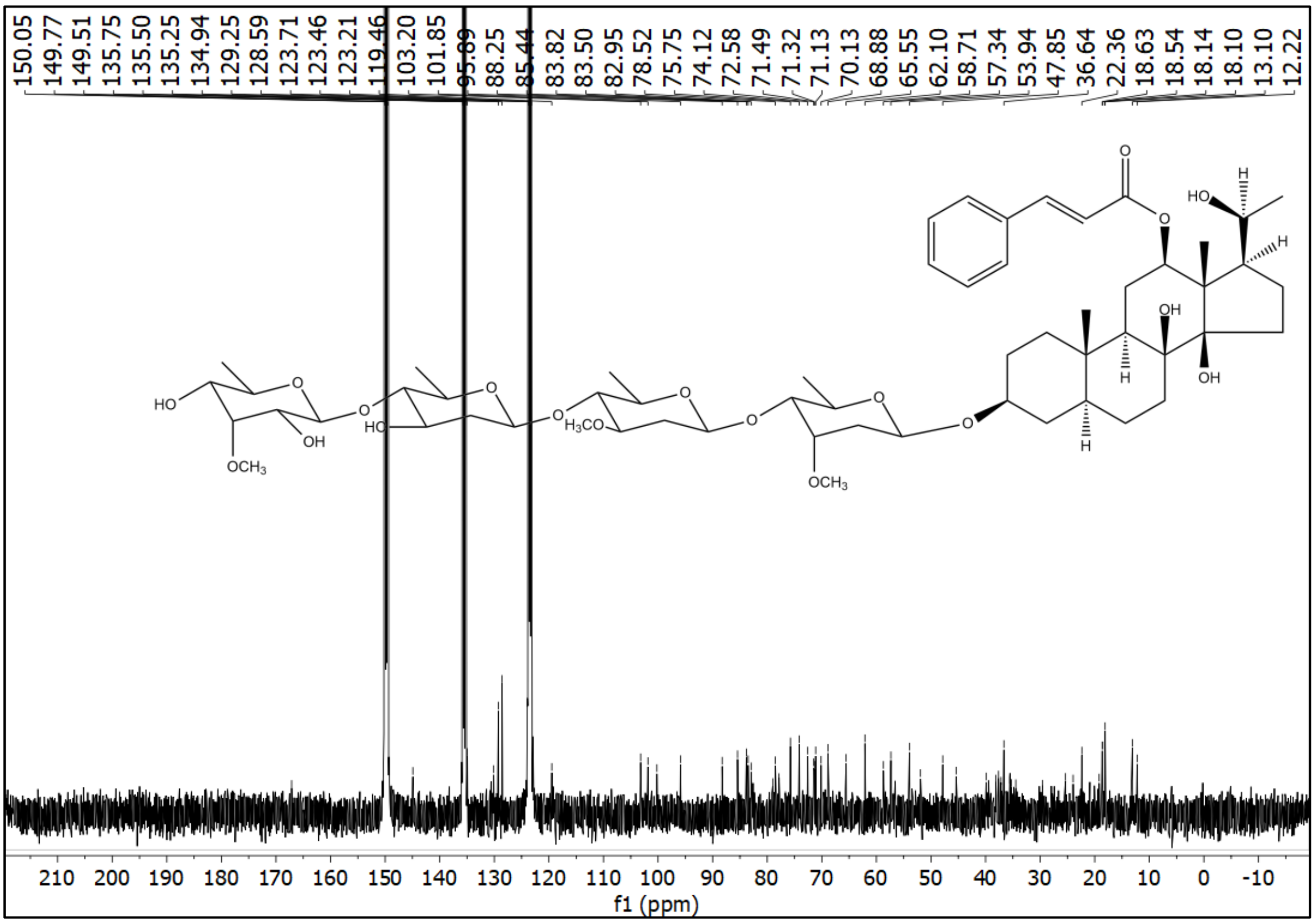

Figure S135. ^{13}C-DEPT135 NMR spectrum (100 MHz, C_5D_5N) of compound 14

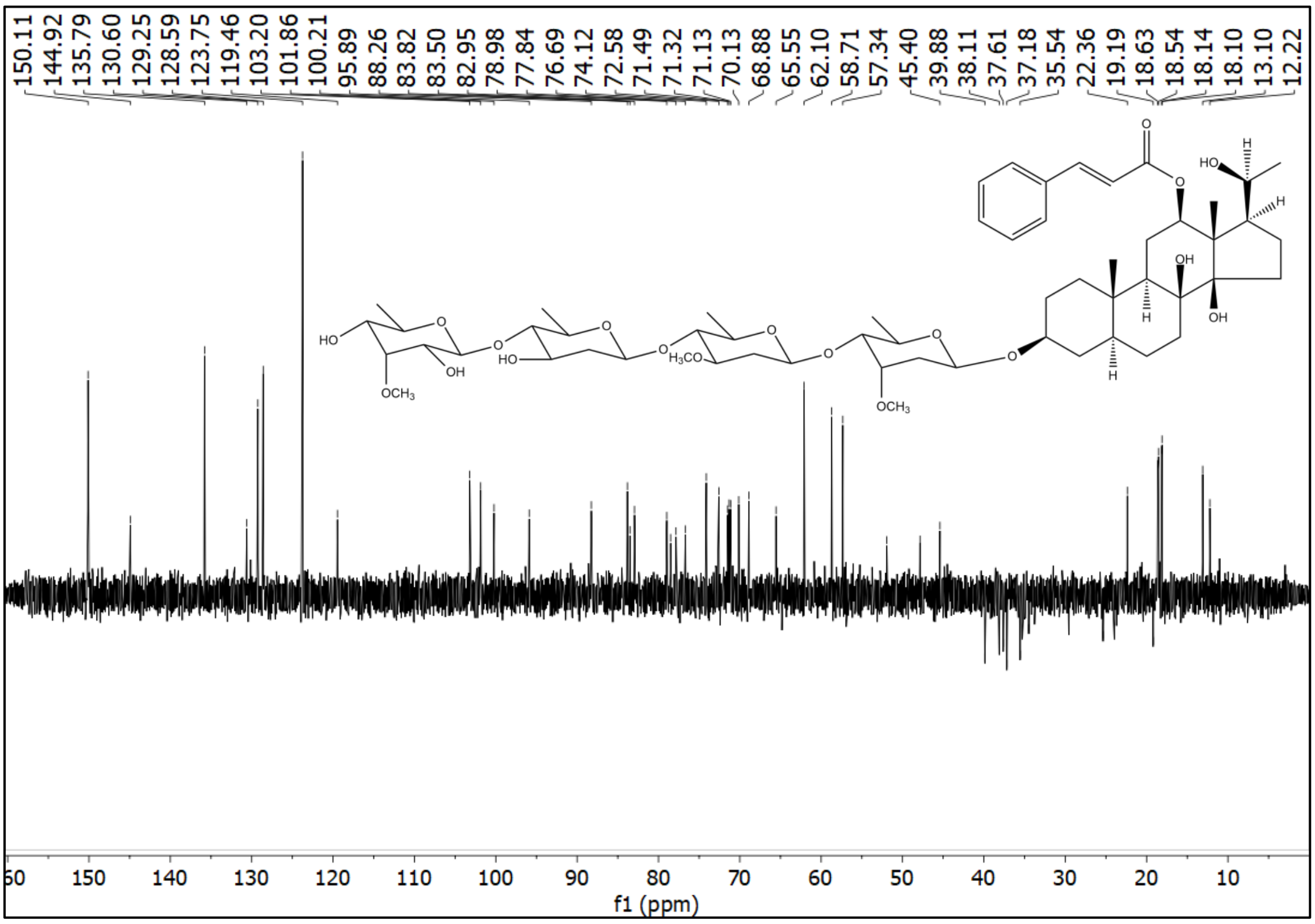

Figure S136. HSQC spectrum (400 x 100 MHz, C_5D_5N) of compound 14

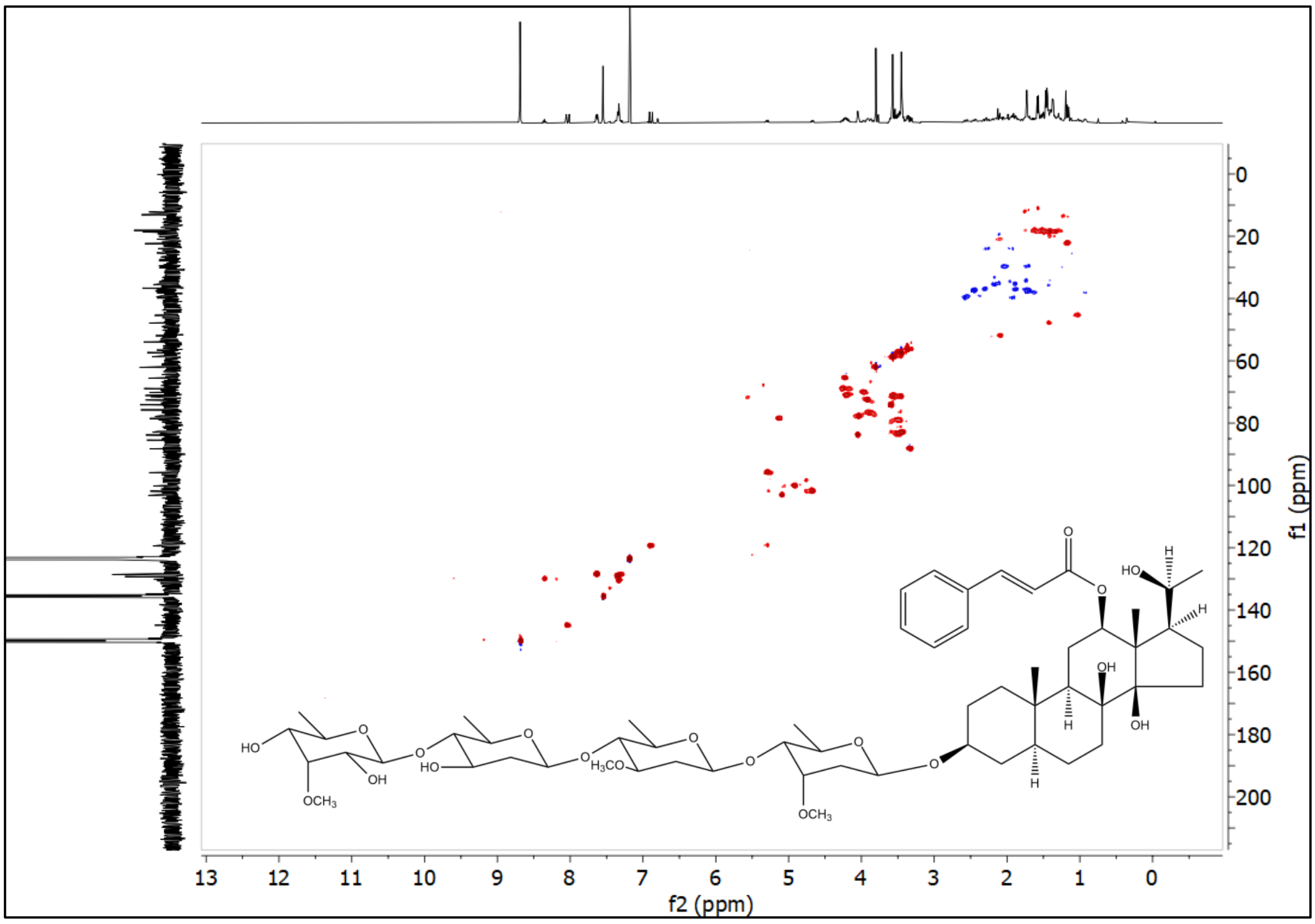

Figure S137. HMBC spectrum (400 x 100 MHz, C_5D_5N) of compound 14

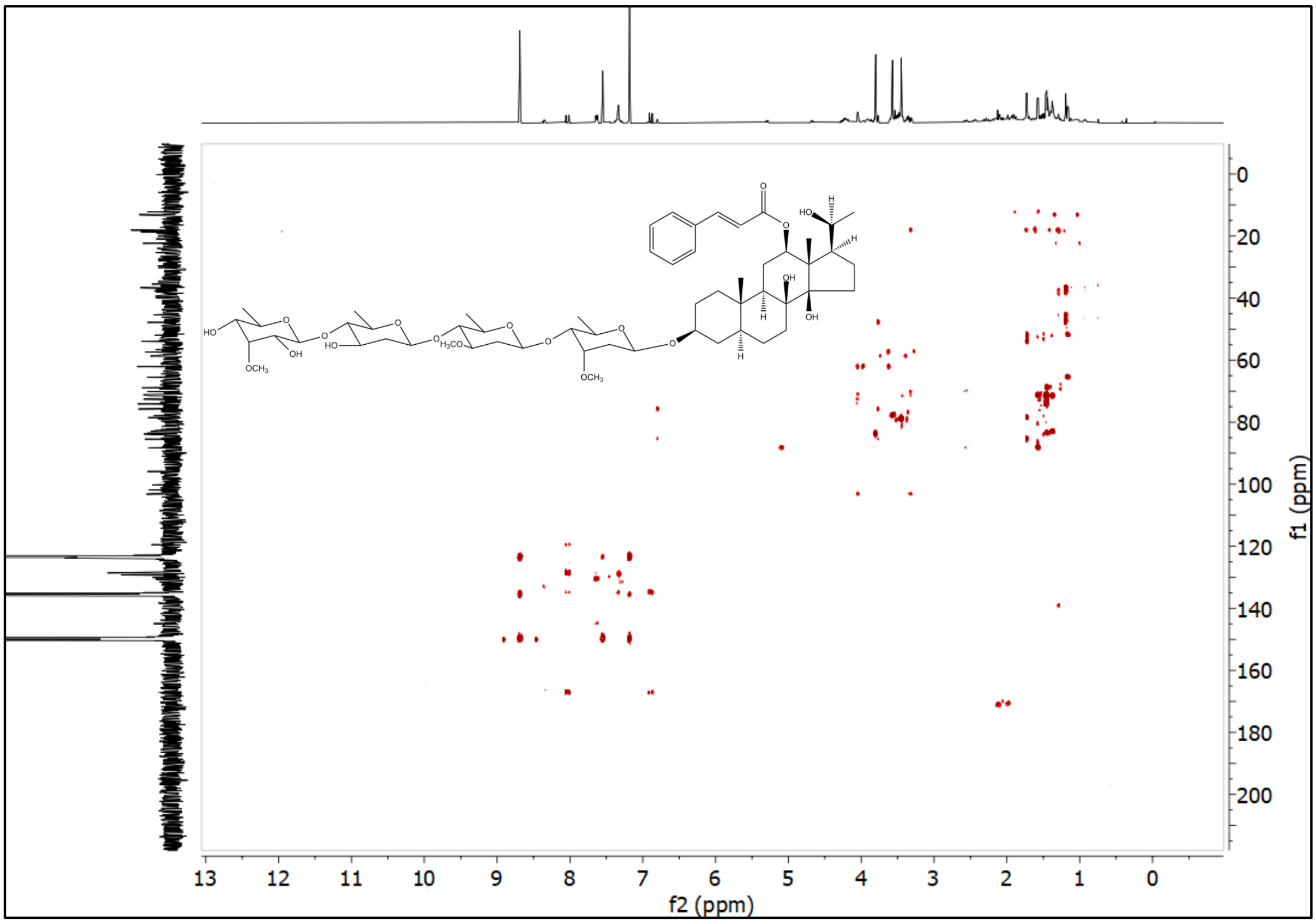

Figure S138. COSY spectrum (400 x 400 MHz, C_5D_5N) of compound 14.

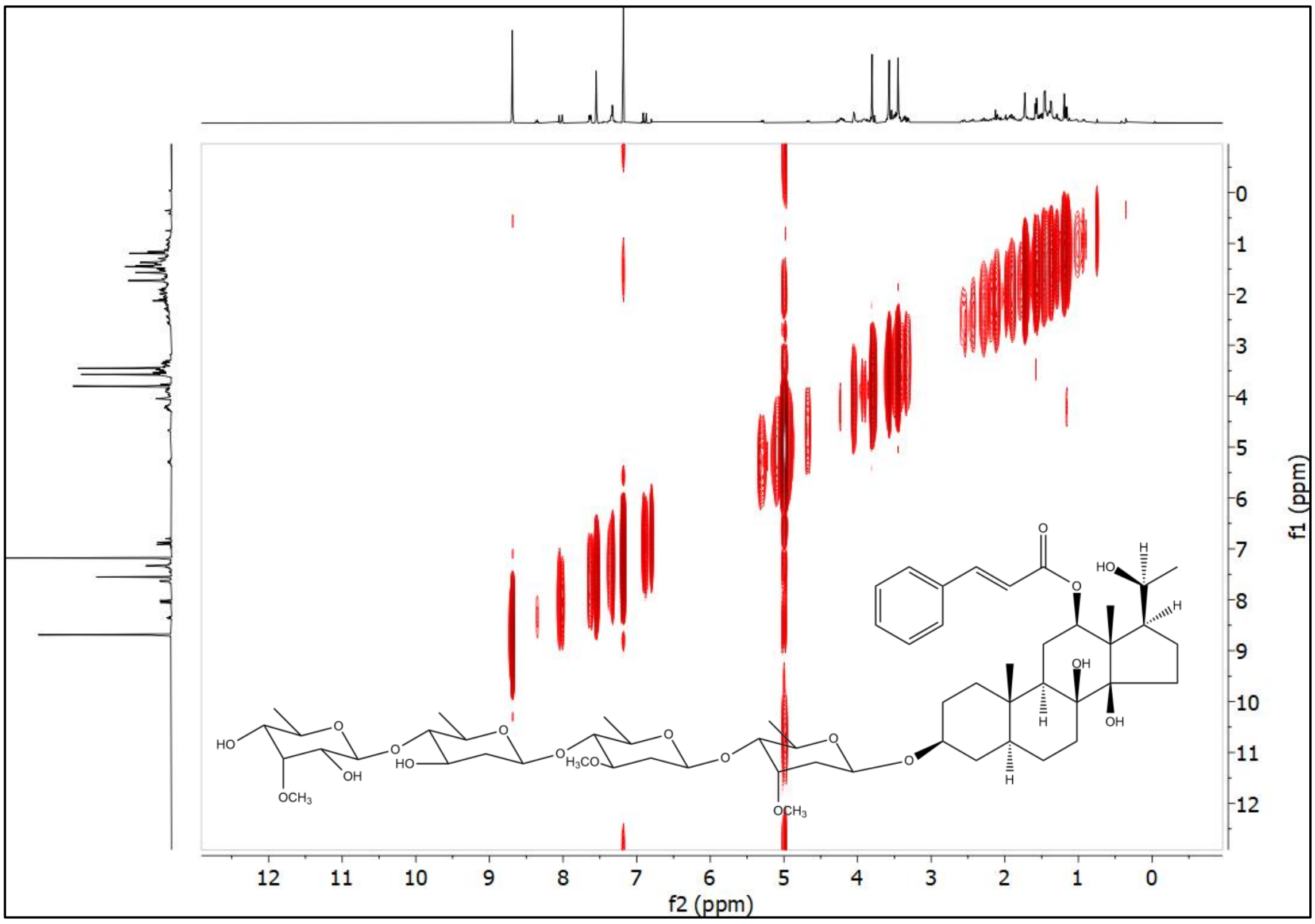

Figure S139. TOCSY spectrum (400 x 400 MHz, C_5D_5N) of compound 14.

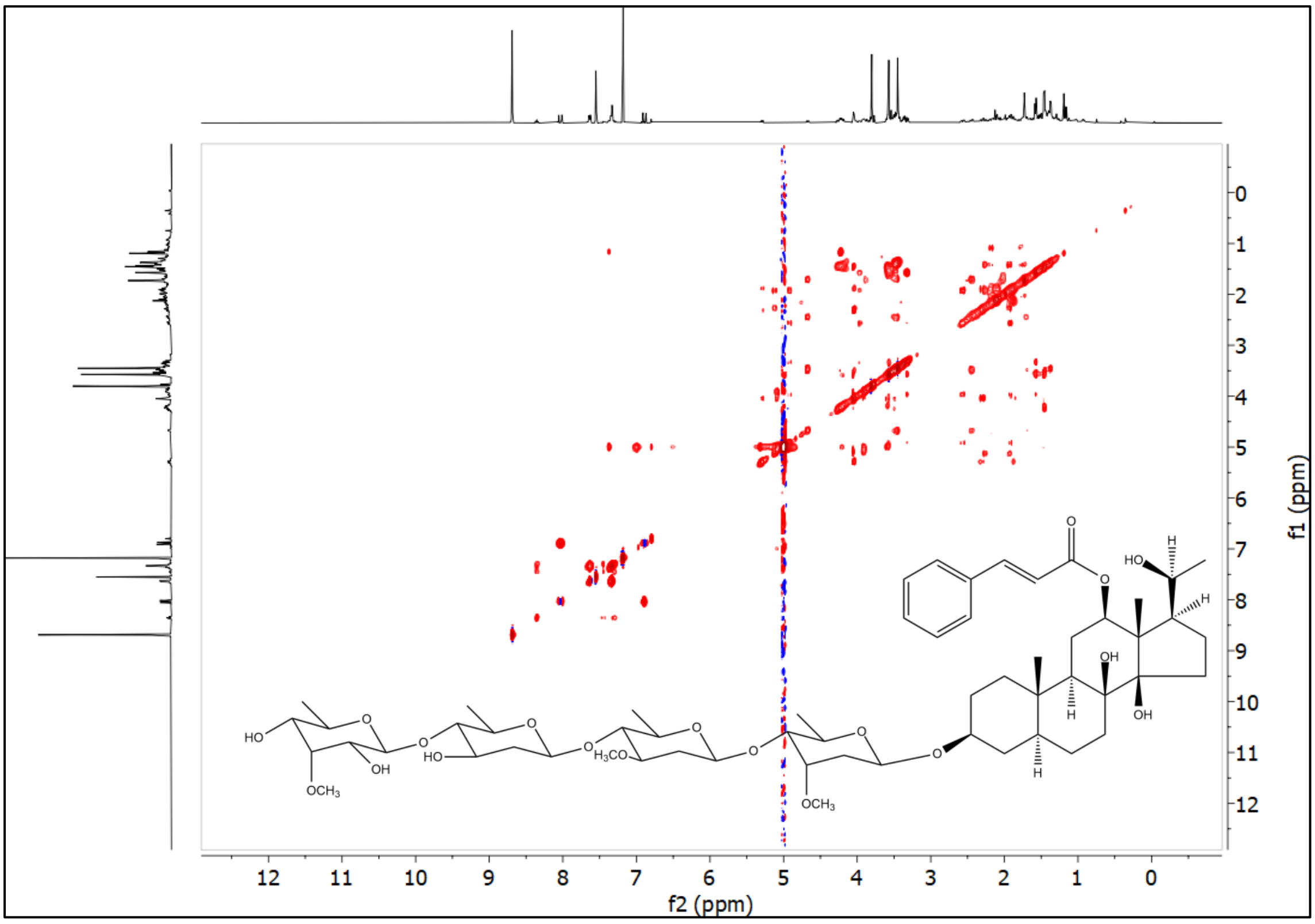

Figure S140. ROESY spectrum (400 x 400 MHz, C_5D_5N) of compound 14

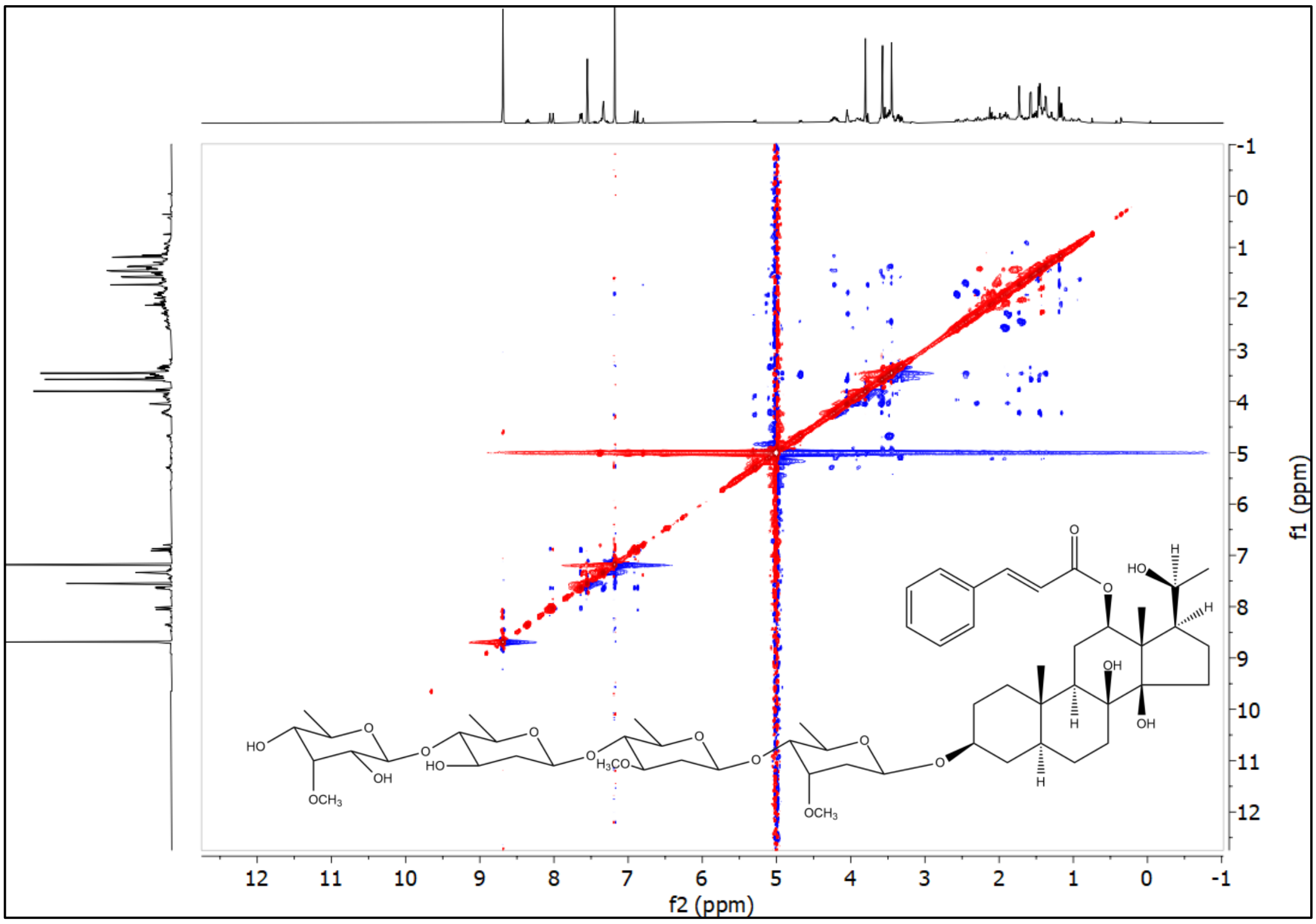

Figure S141. ATR-Infrared spectrum of compound 15.

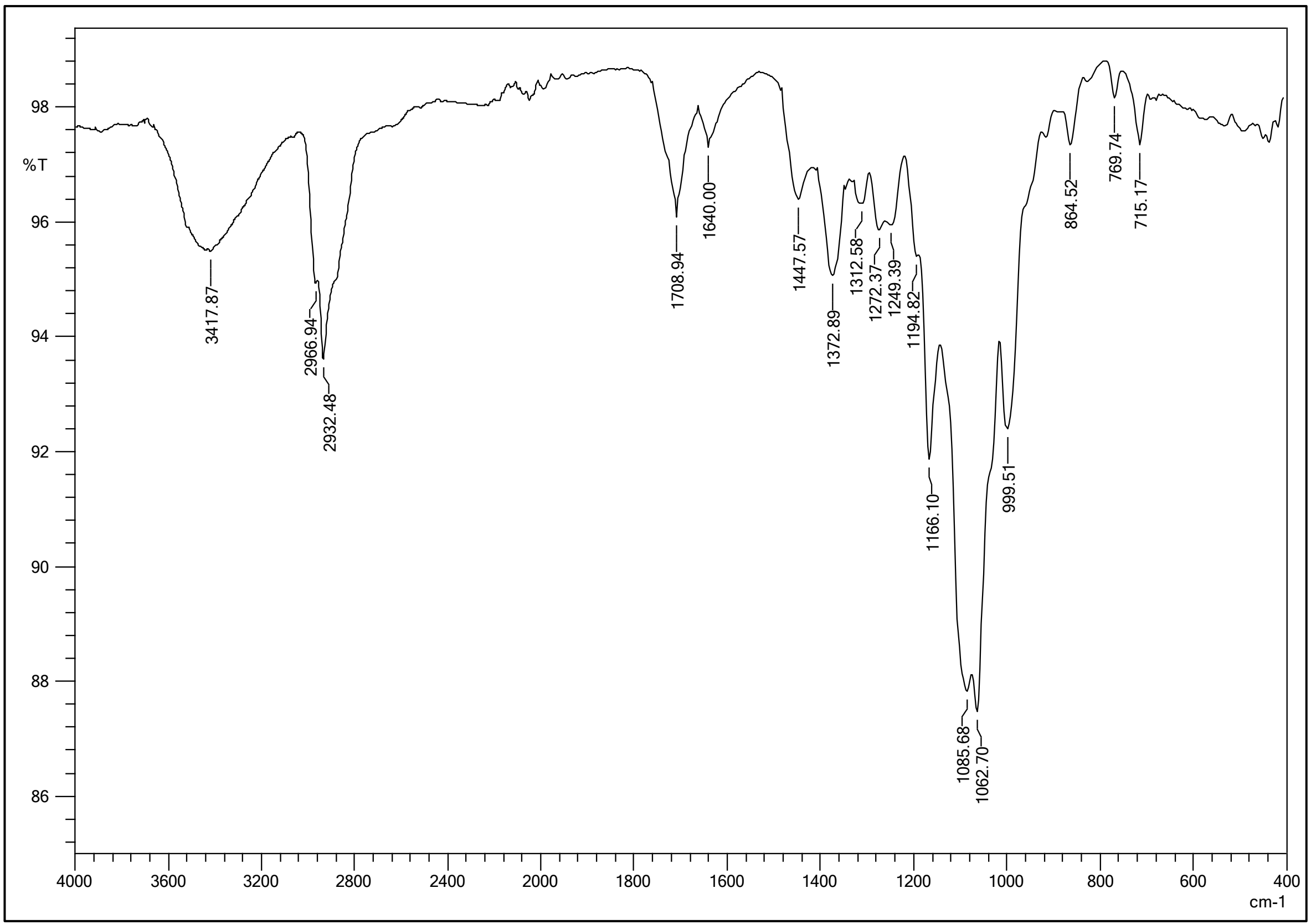

Figure S142. HR-ESI-MS mass spectrum of compound 15.

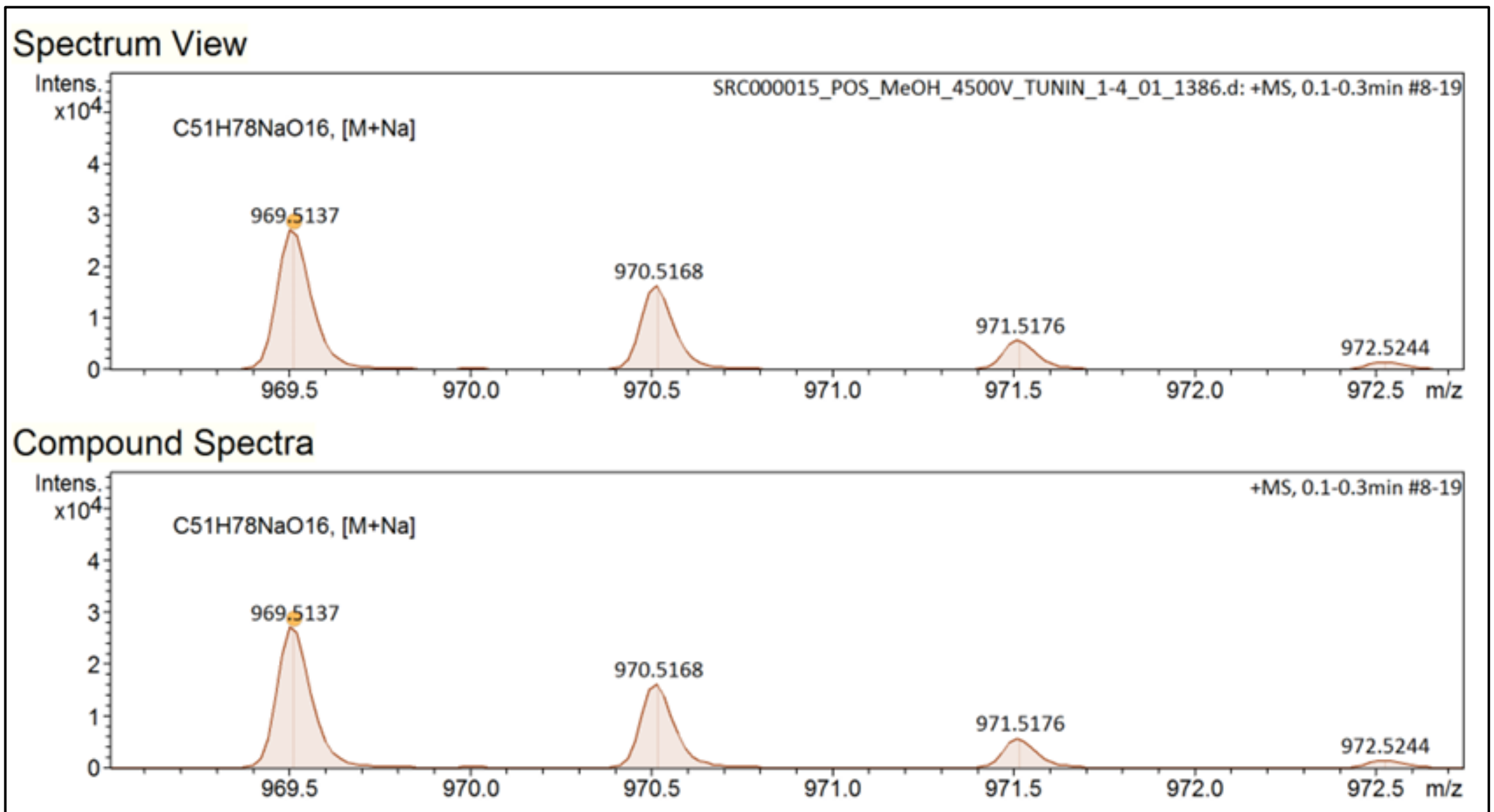

Meas. m/z	#	Ion Formula	m/z	err [ppm]	mSigma	# mSigma	Score	rdb	e⁻ Conf	N-Rule
969.5137	1	C51H78NaO16	969.5182	4.7	18.1	1	100.00	12.5	even	ok

Figure S143. ^{1}H NMR spectrum (500 MHz, C_5D_5N) of compound 15

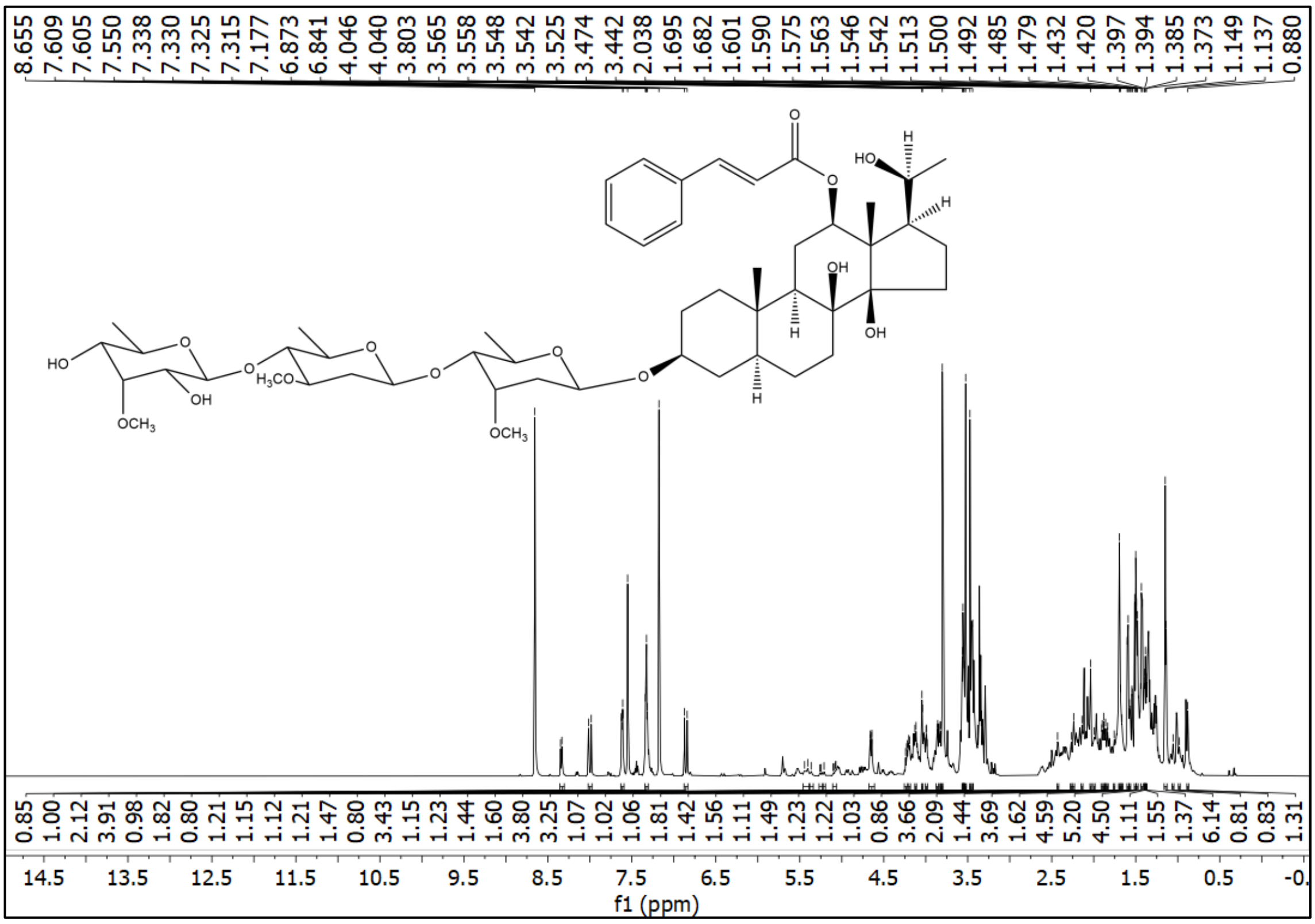

Figure S144. ^{13}C-BB NMR spectrum (125 MHz, C_5D_5N) of compound 15

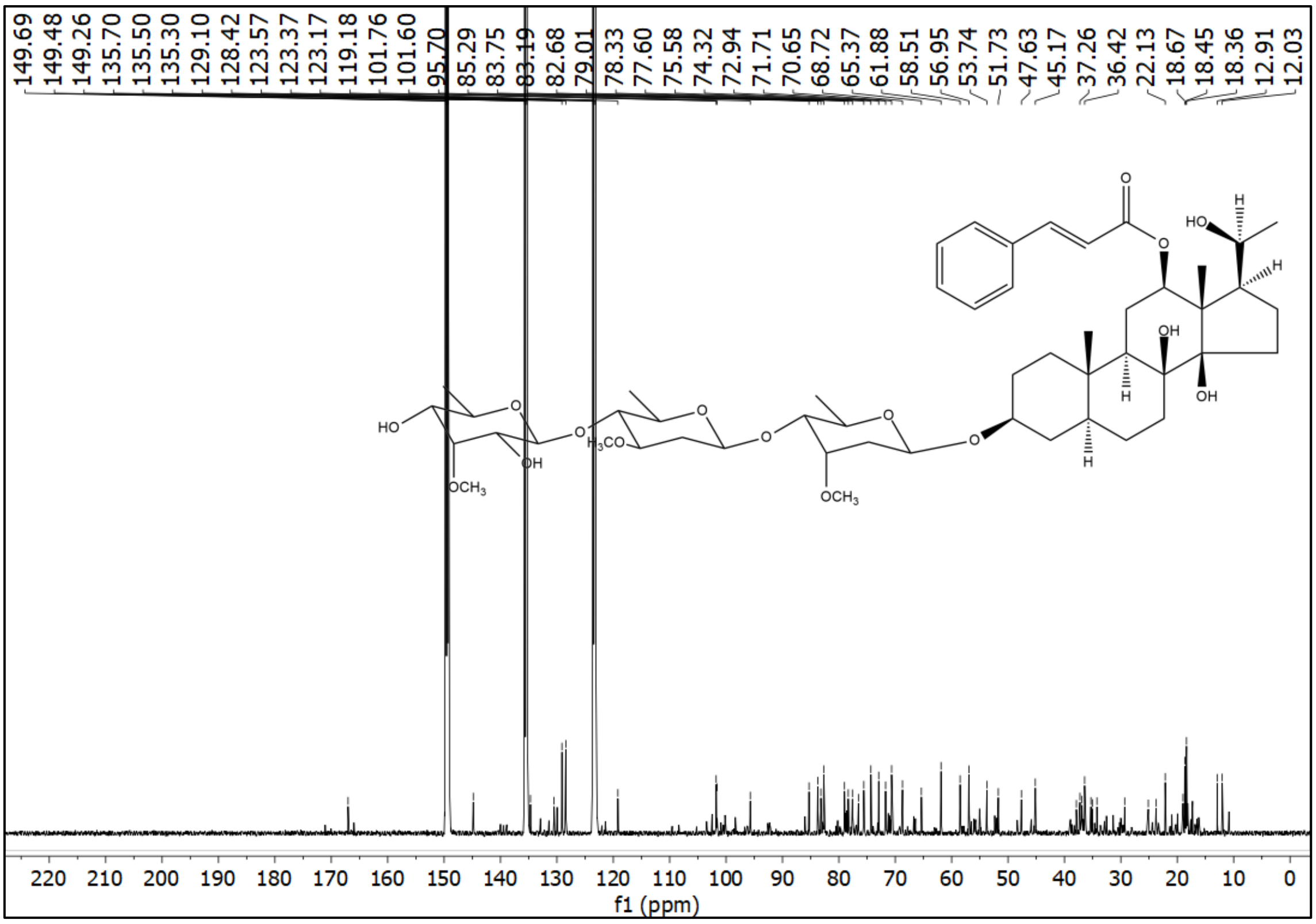

Figure S145. ^{13}C-DEPT135 NMR spectrum (125 MHz, C_5D_5N) of compound 15

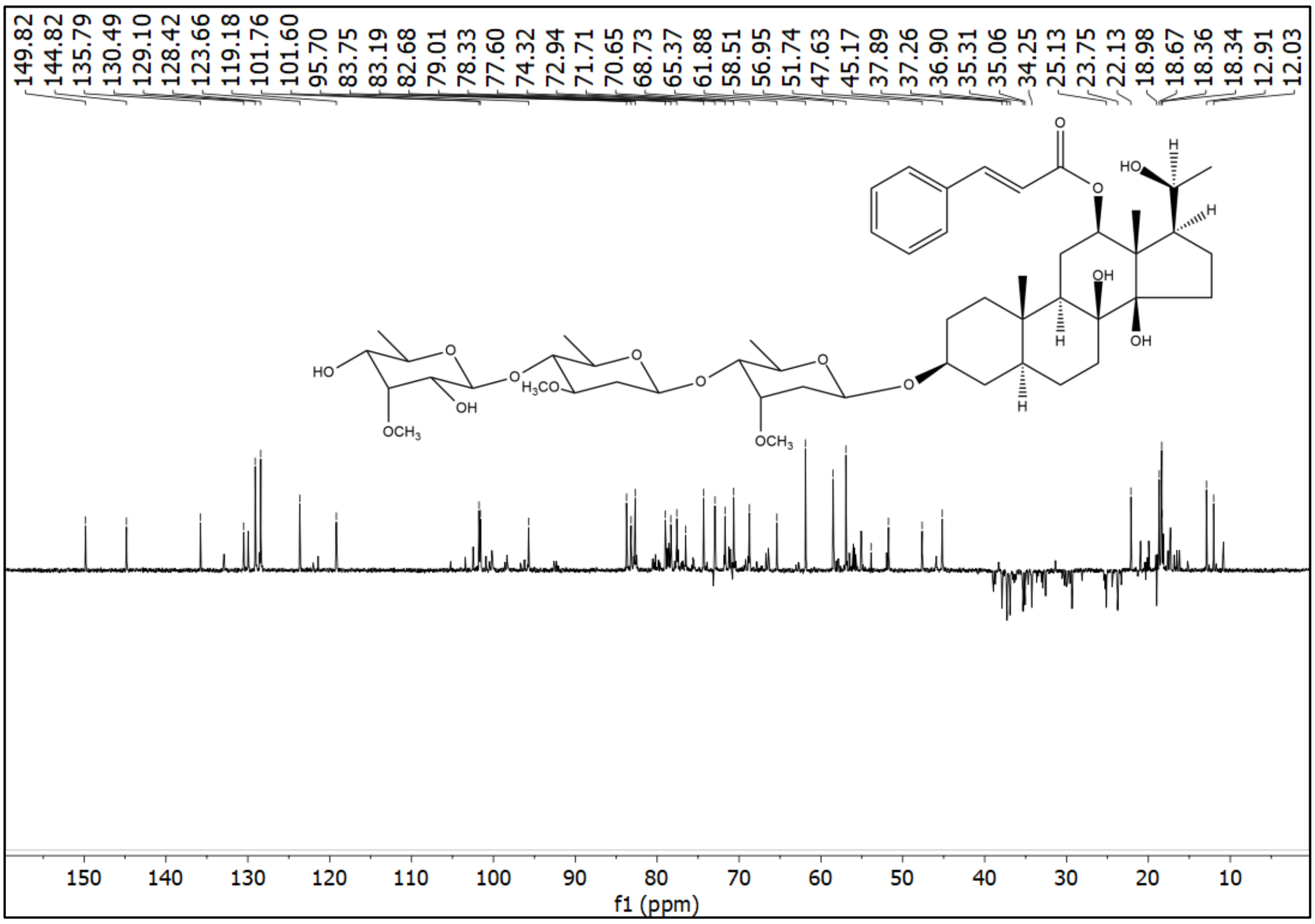

Figure S146. HSQC spectrum (500 x 125 MHz, C_5D_5N) of compound 15

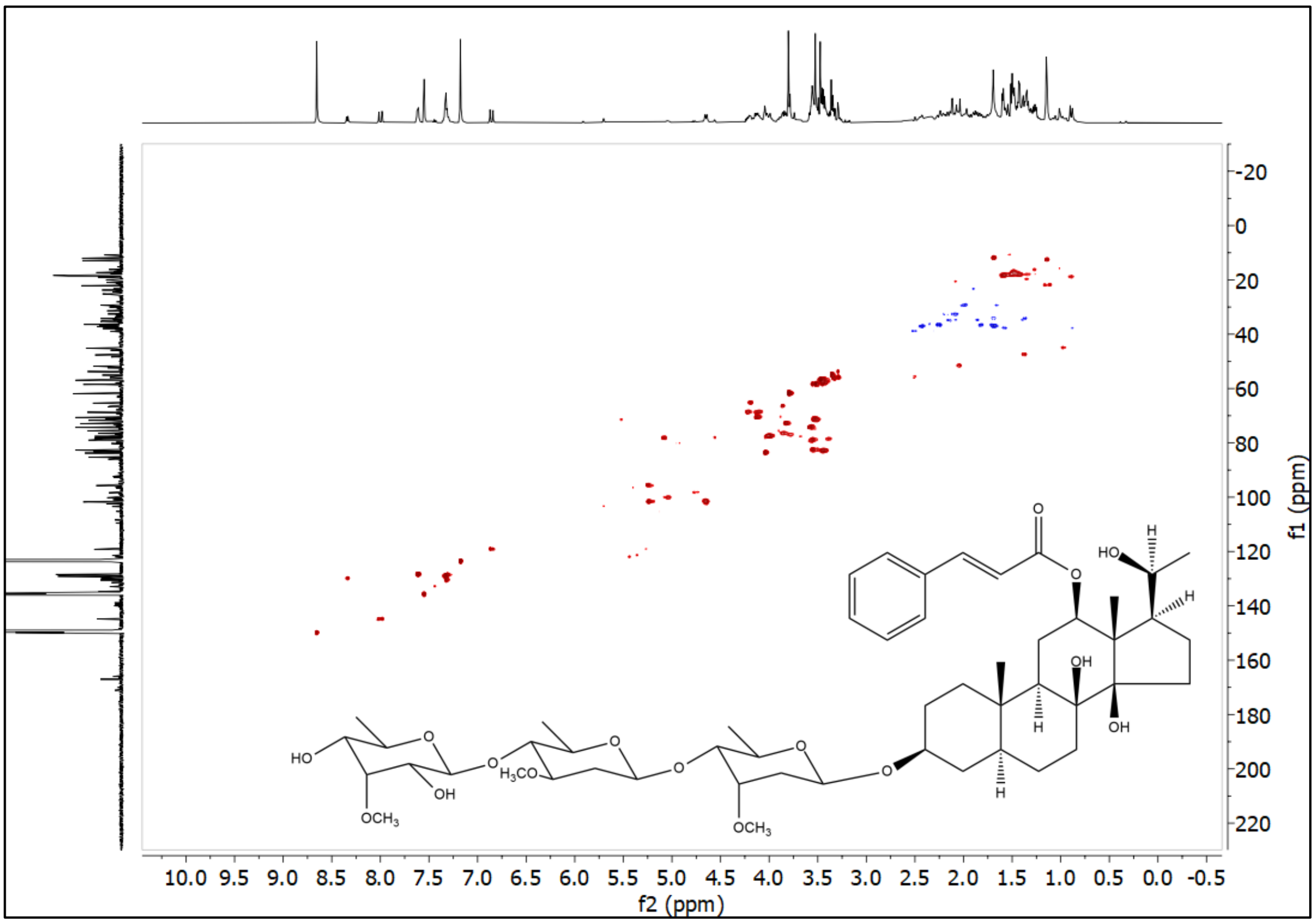

Figure S147. HMBC spectrum (500 x 125 MHz, C_5D_5N) of compound 15

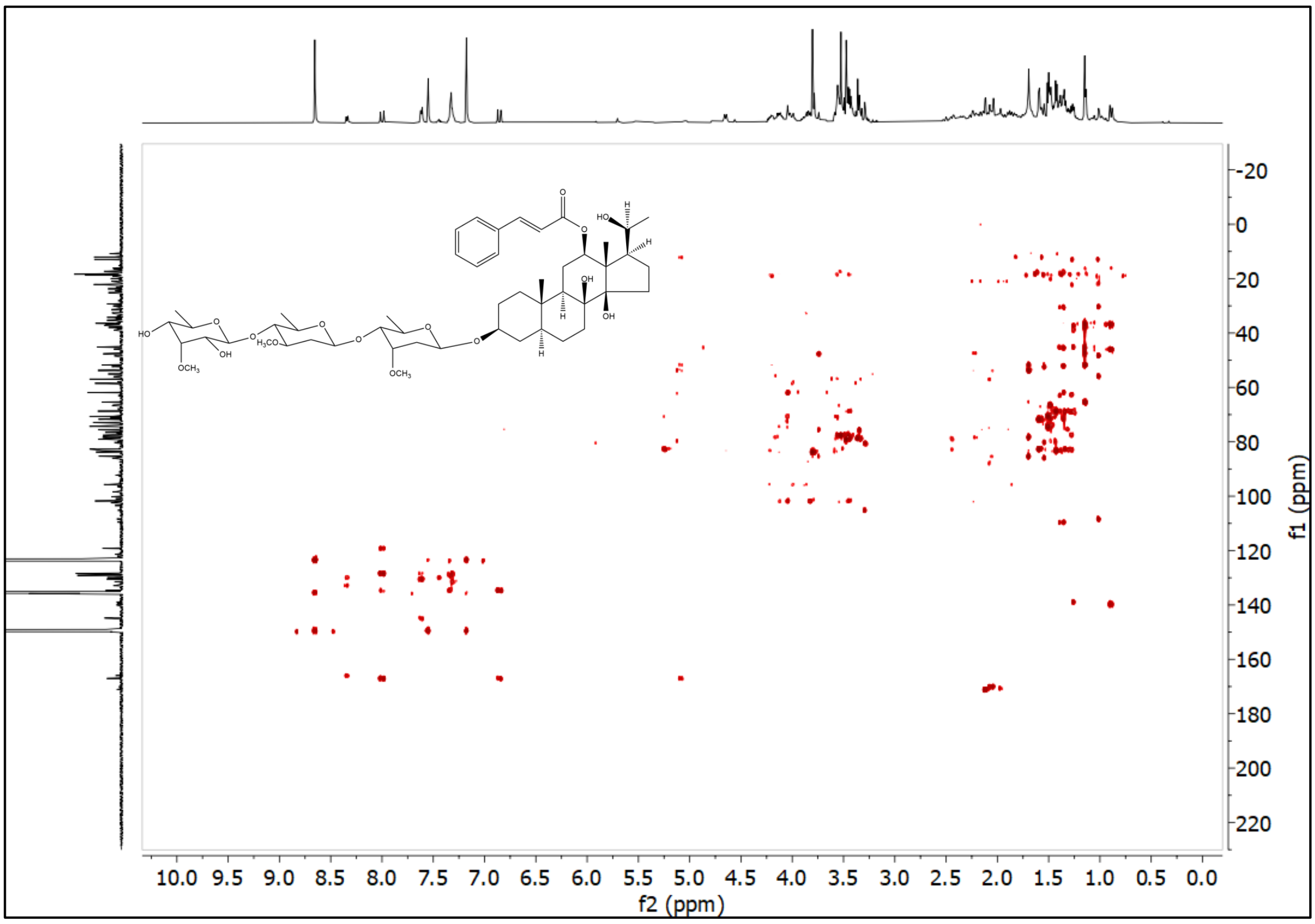

Figure S148. COSY spectrum (500 x 500 MHz, C_5D_5N) of compound 15.

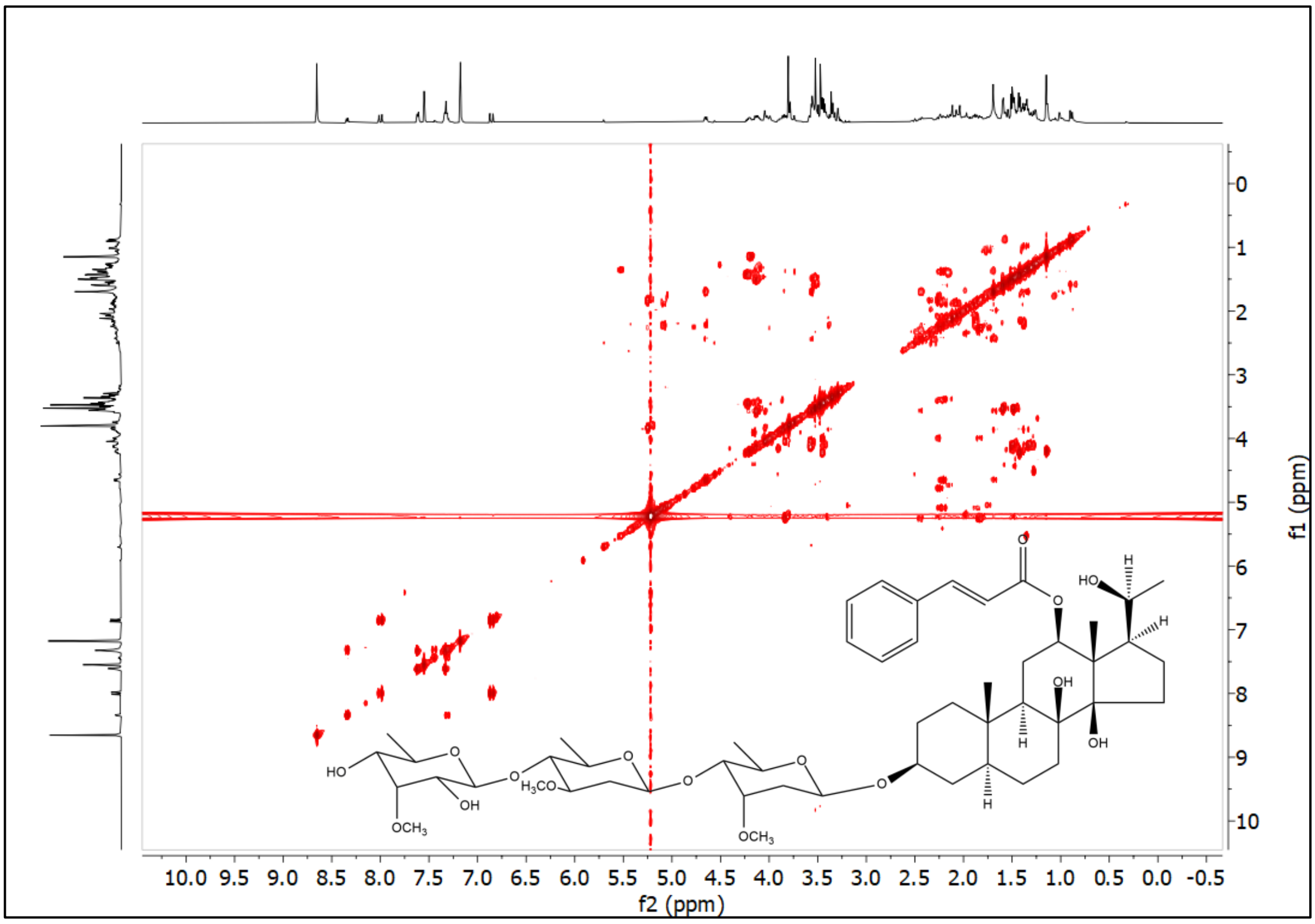

Figure S149. TOCSY spectrum (500 x 500 MHz, C_5D_5N) of compound 15.

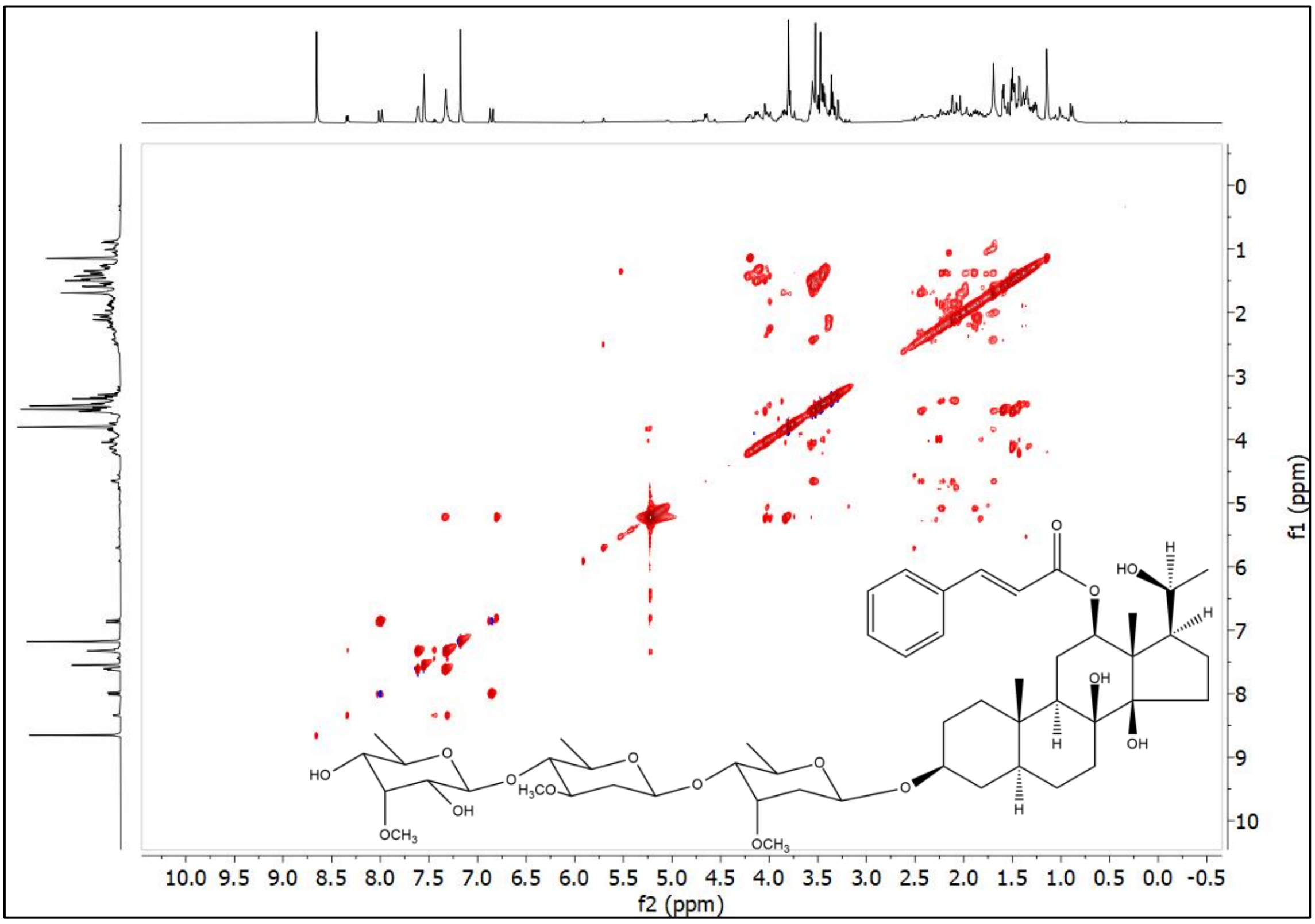

Figure S150. NOESY spectrum (500 x 500 MHz, C_5D_5N) of compound 15.

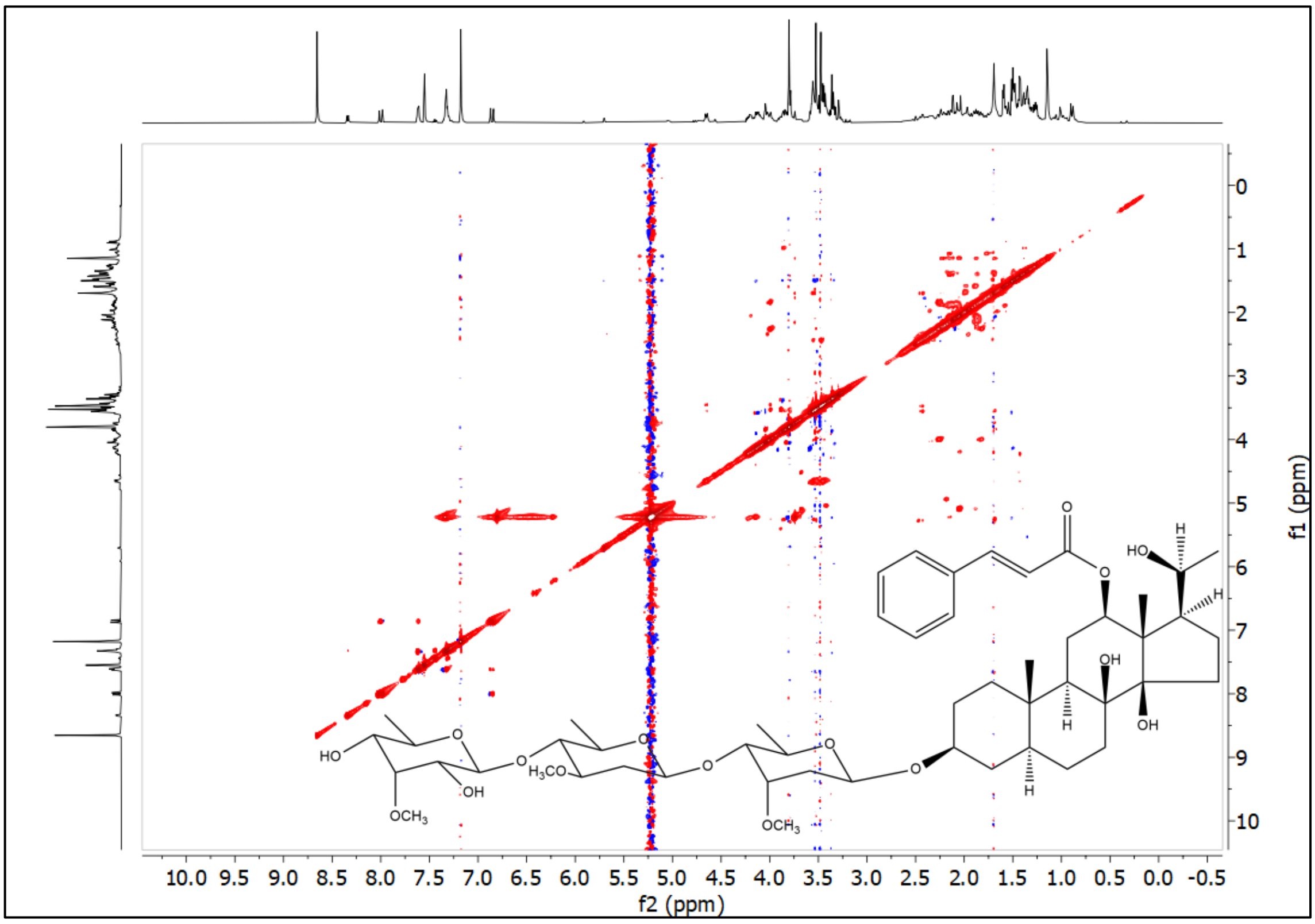

Figure S151. ATR-Infrared spectrum of compound 16.

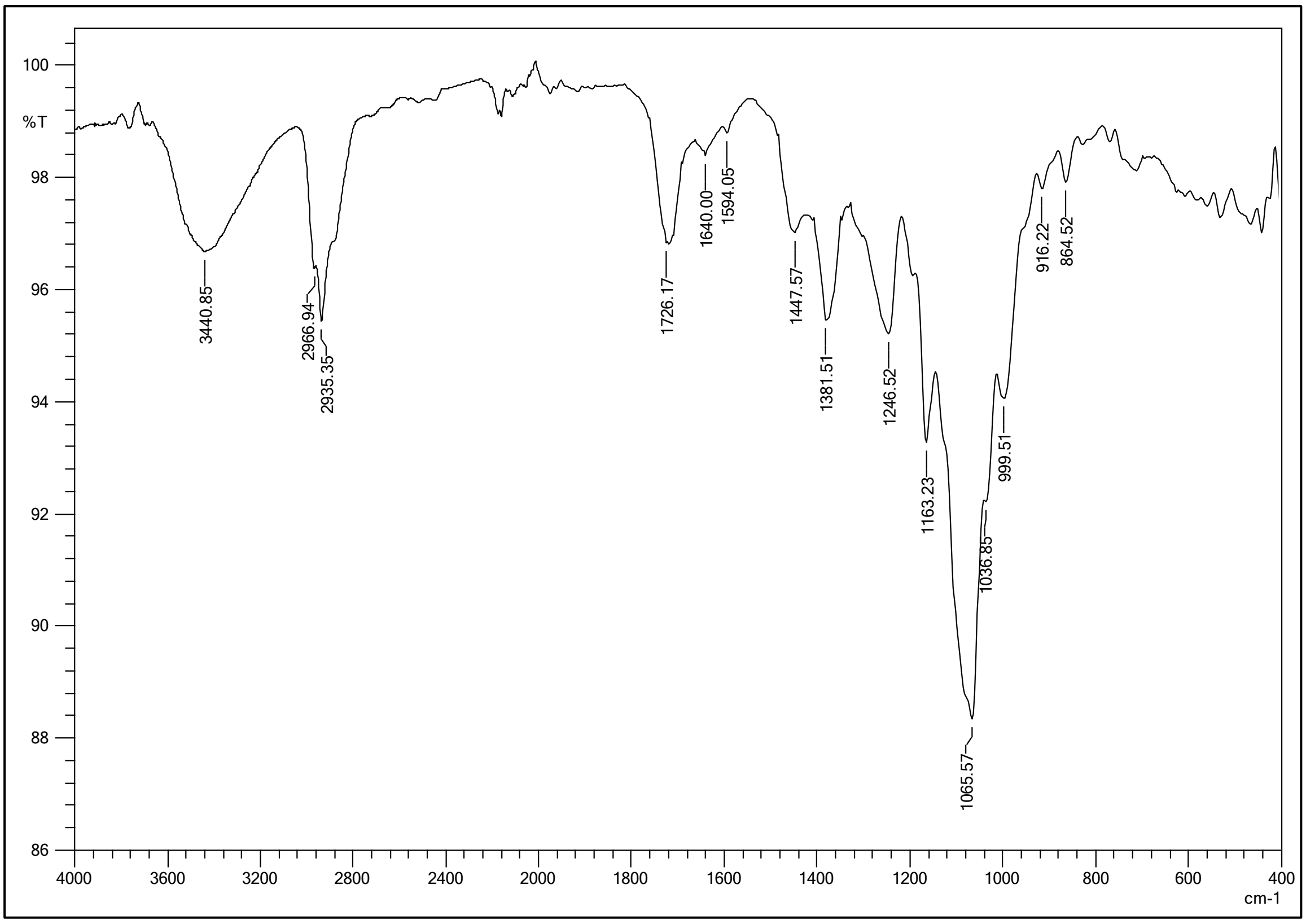

Figure S152. HR-ESI-MS mass spectrum of compound 16.

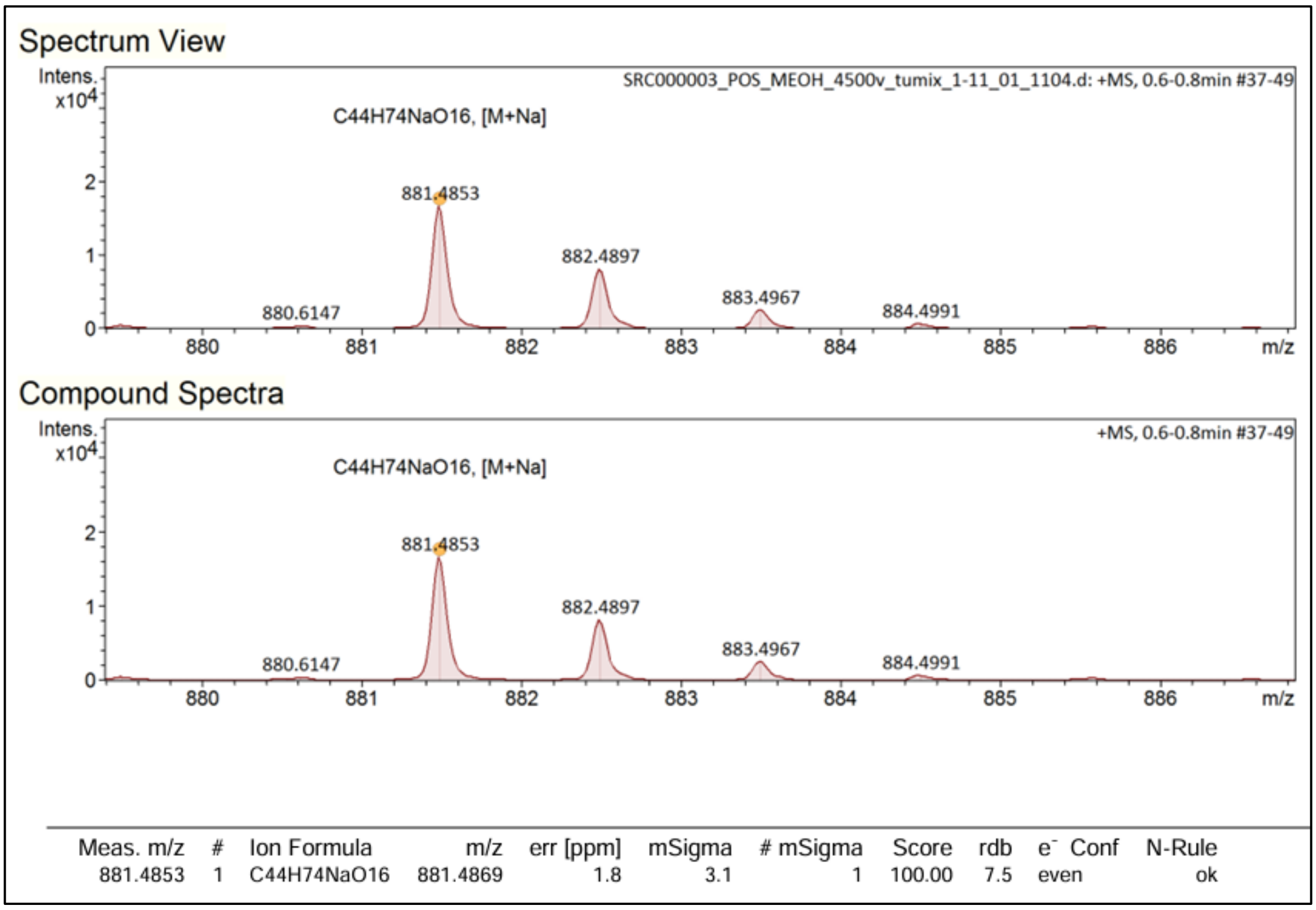

Figure S153. ^{1}H NMR spectrum (500 MHz, C_5D_5N) of compound 16

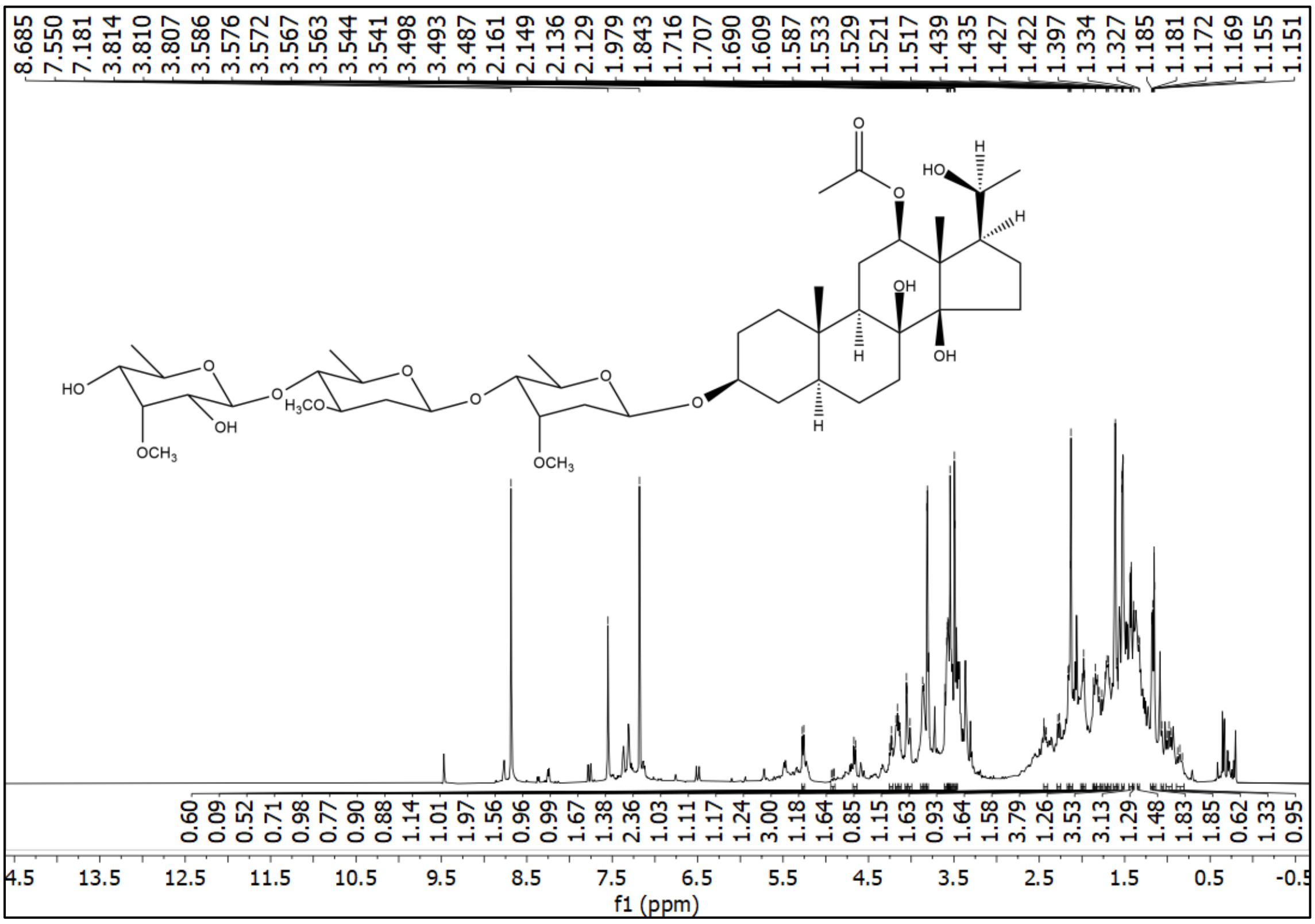

Figure S154. ^{13}C-BB NMR spectrum (125 MHz, C_5D_5N) of compound 16

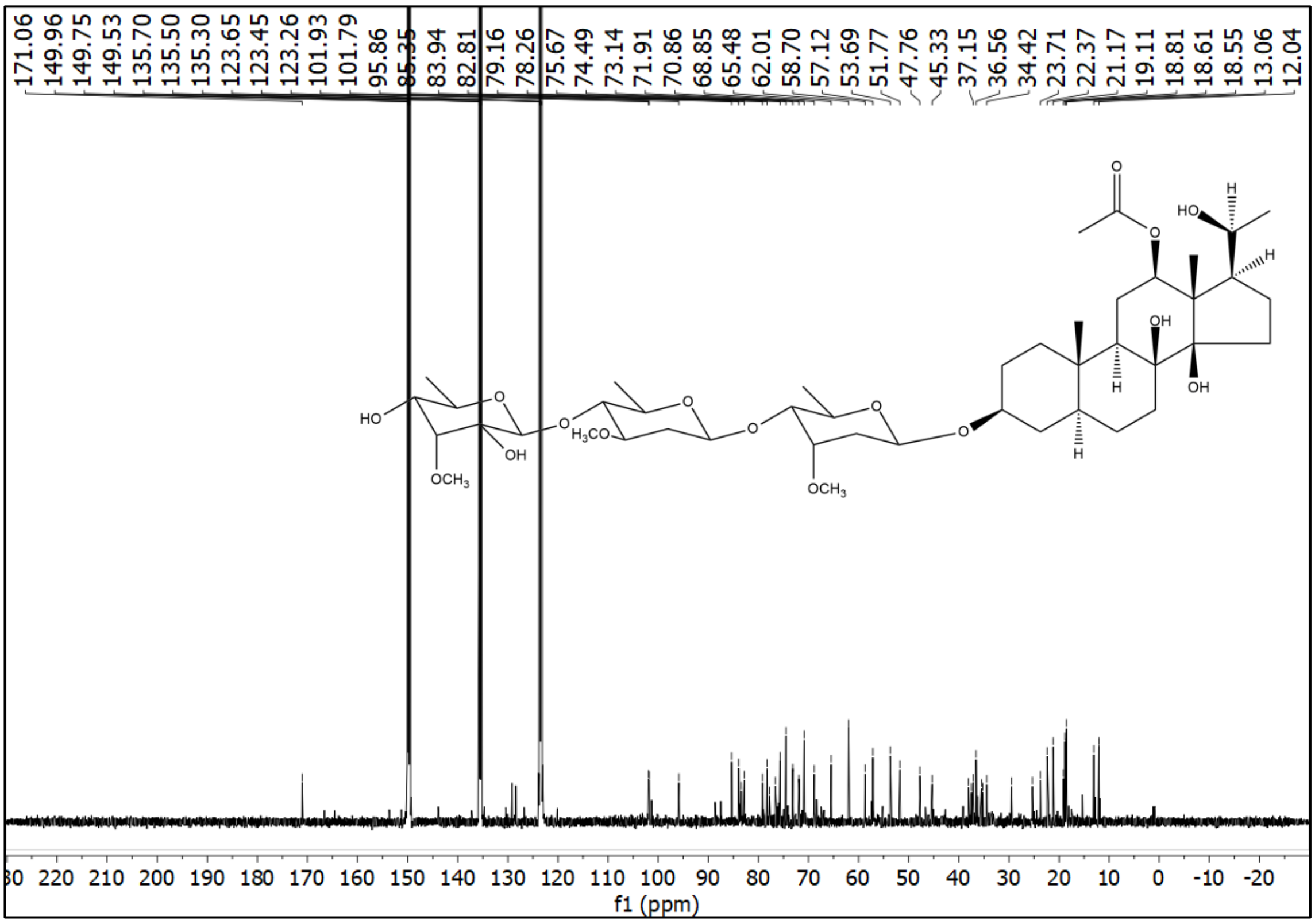

Figure S155. ^{13}C-DEPT135 NMR spectrum (125 MHz, C_5D_5N) of compound 16

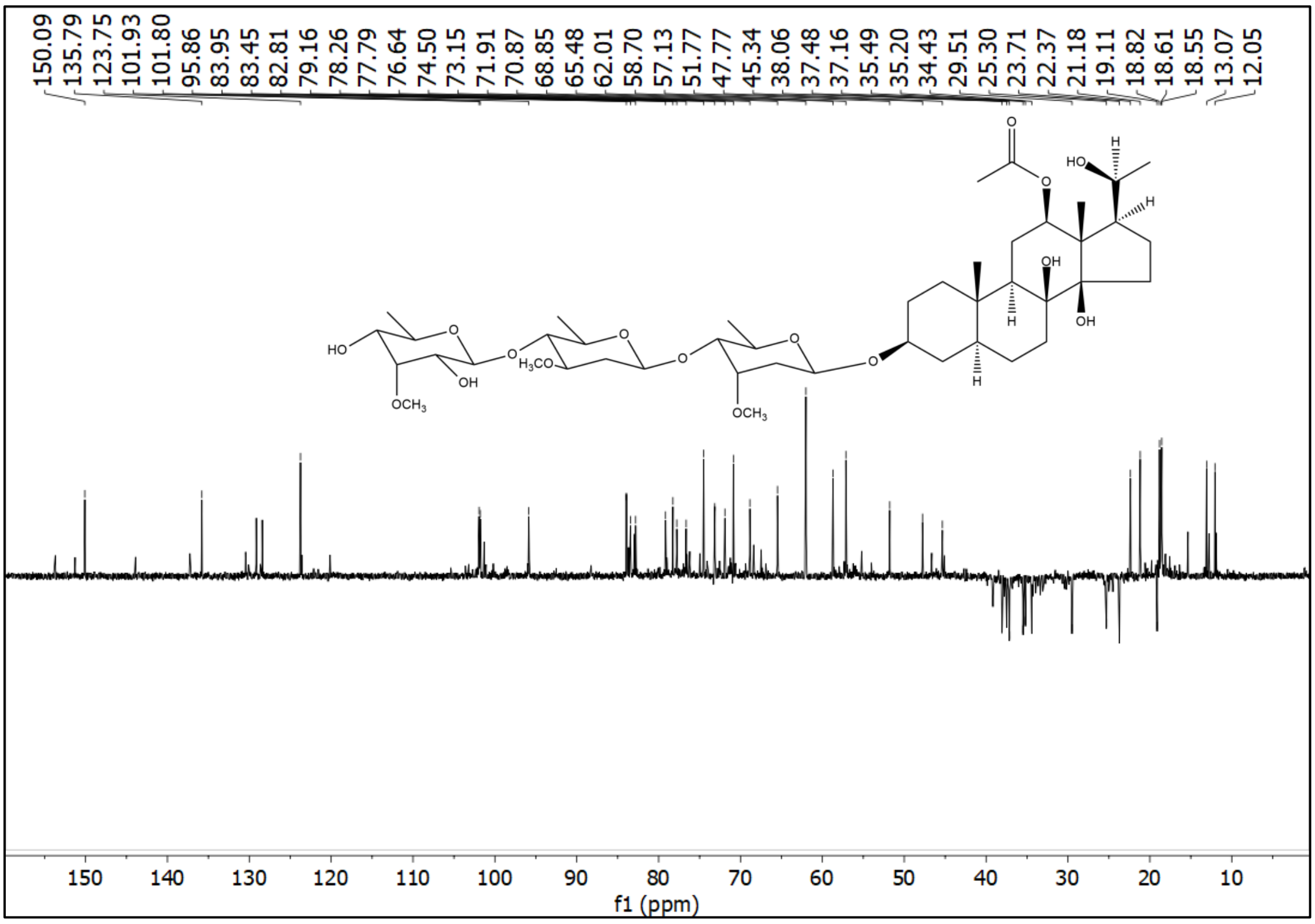

Figure S156. HSQC spectrum (500 x 125 MHz, C_5D_5N) of compound 16

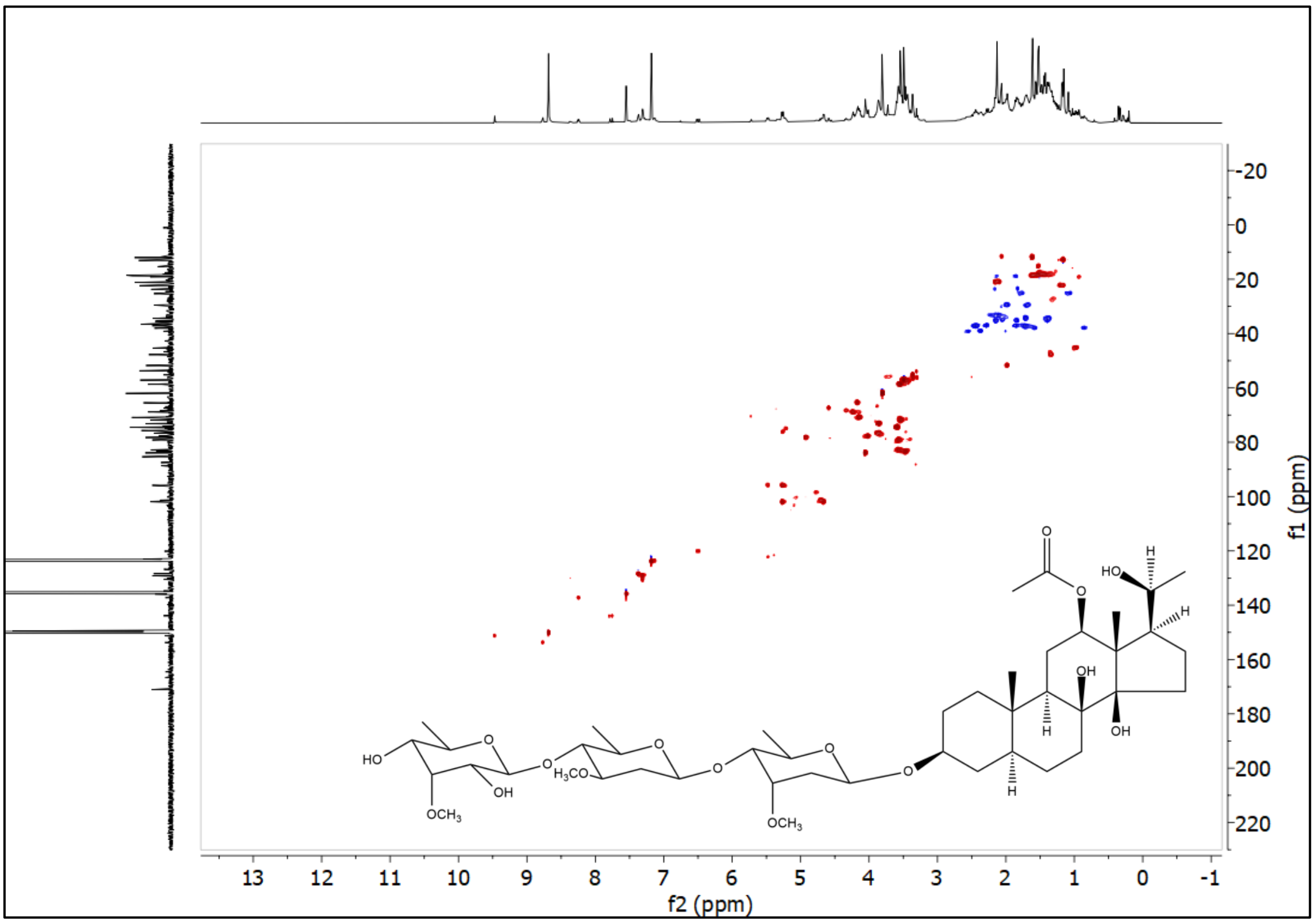

Figure S157. HMBC spectrum (500 x 125 MHz, C_5D_5N) of compound 16

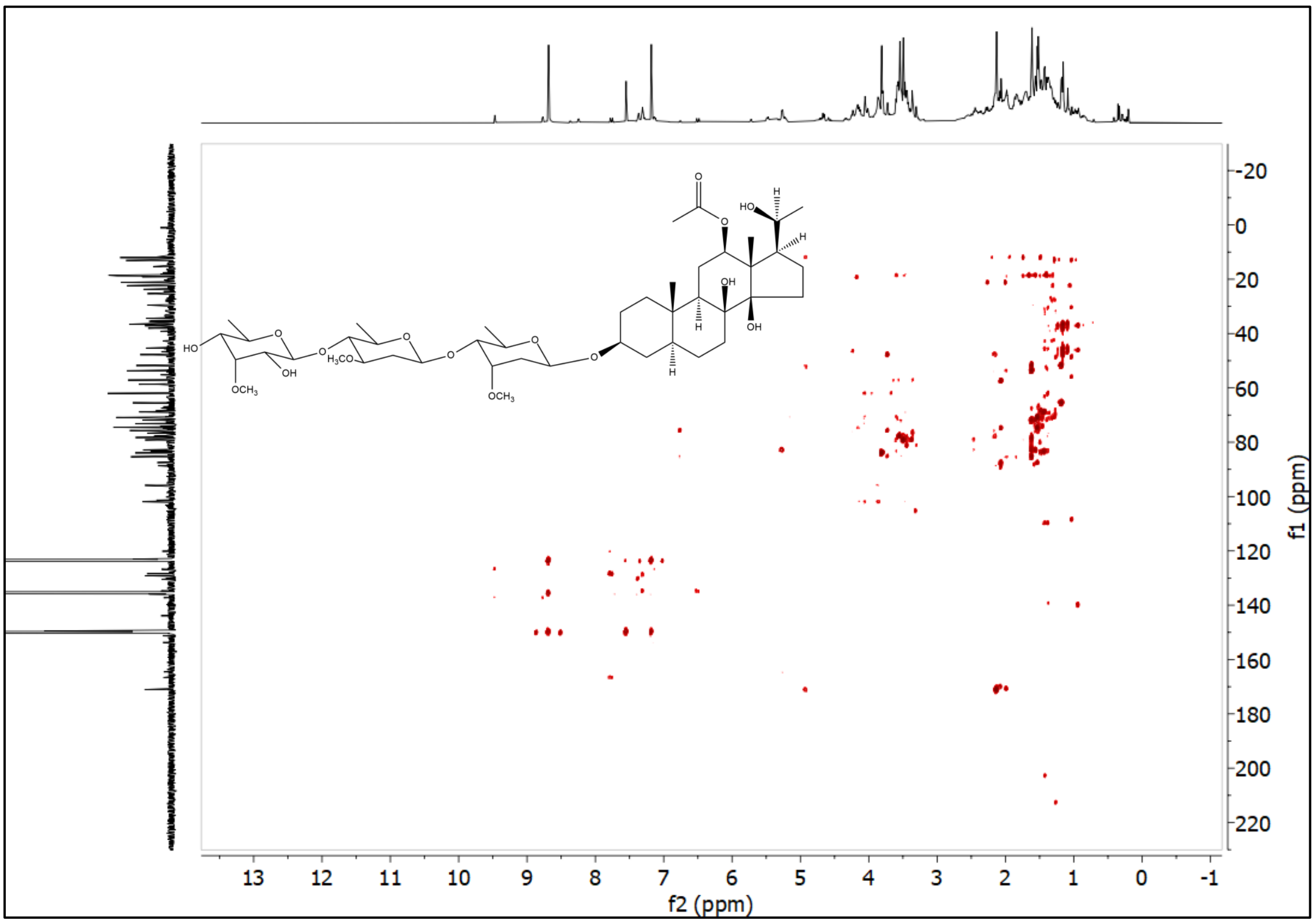

Figure S158. COSY spectrum (500 x 500 MHz, C_5D_5N) of compound 16

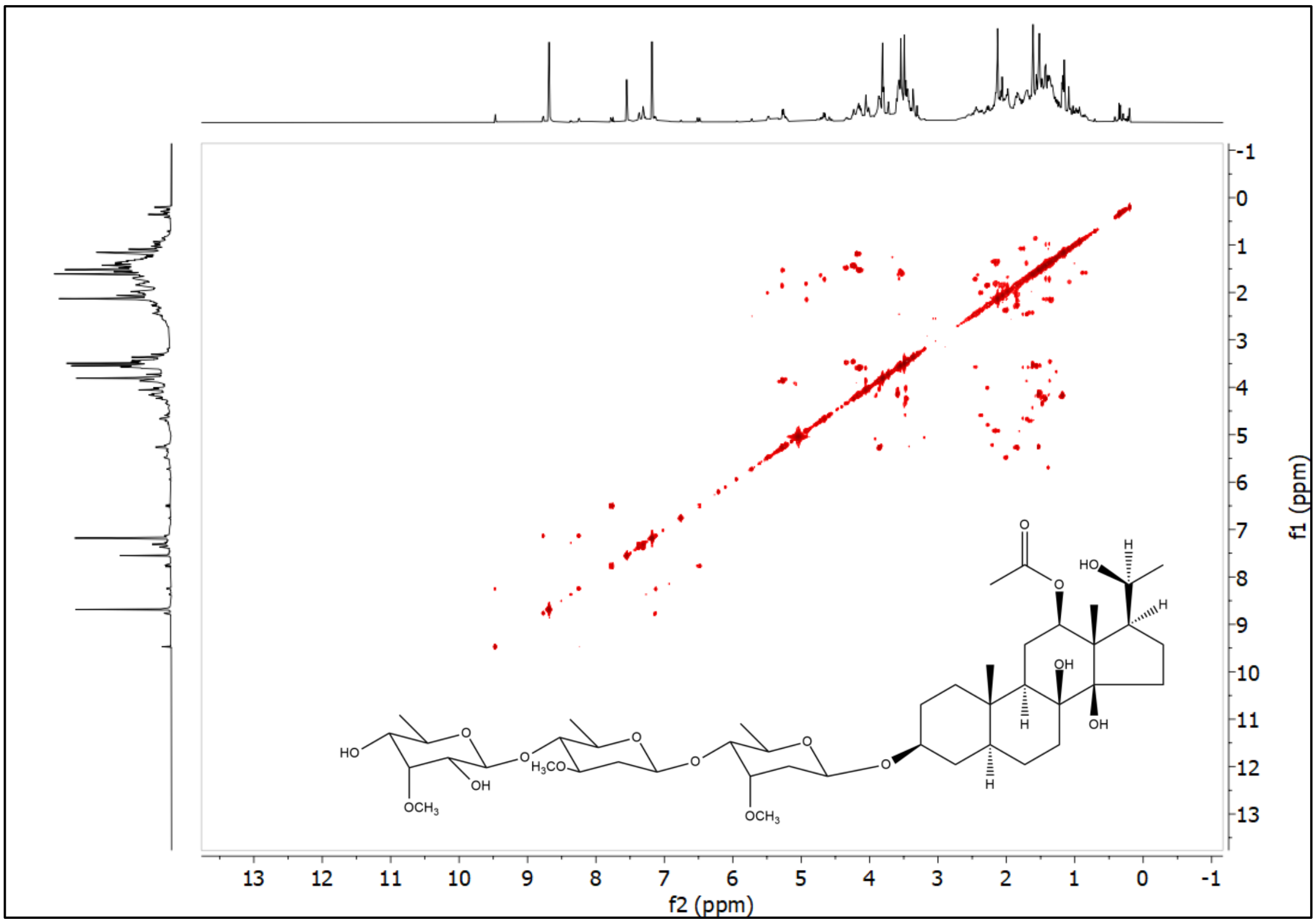

Figure S159. TOCSY spectrum (500 x 500 MHz, C_5D_5N) of compound 16.

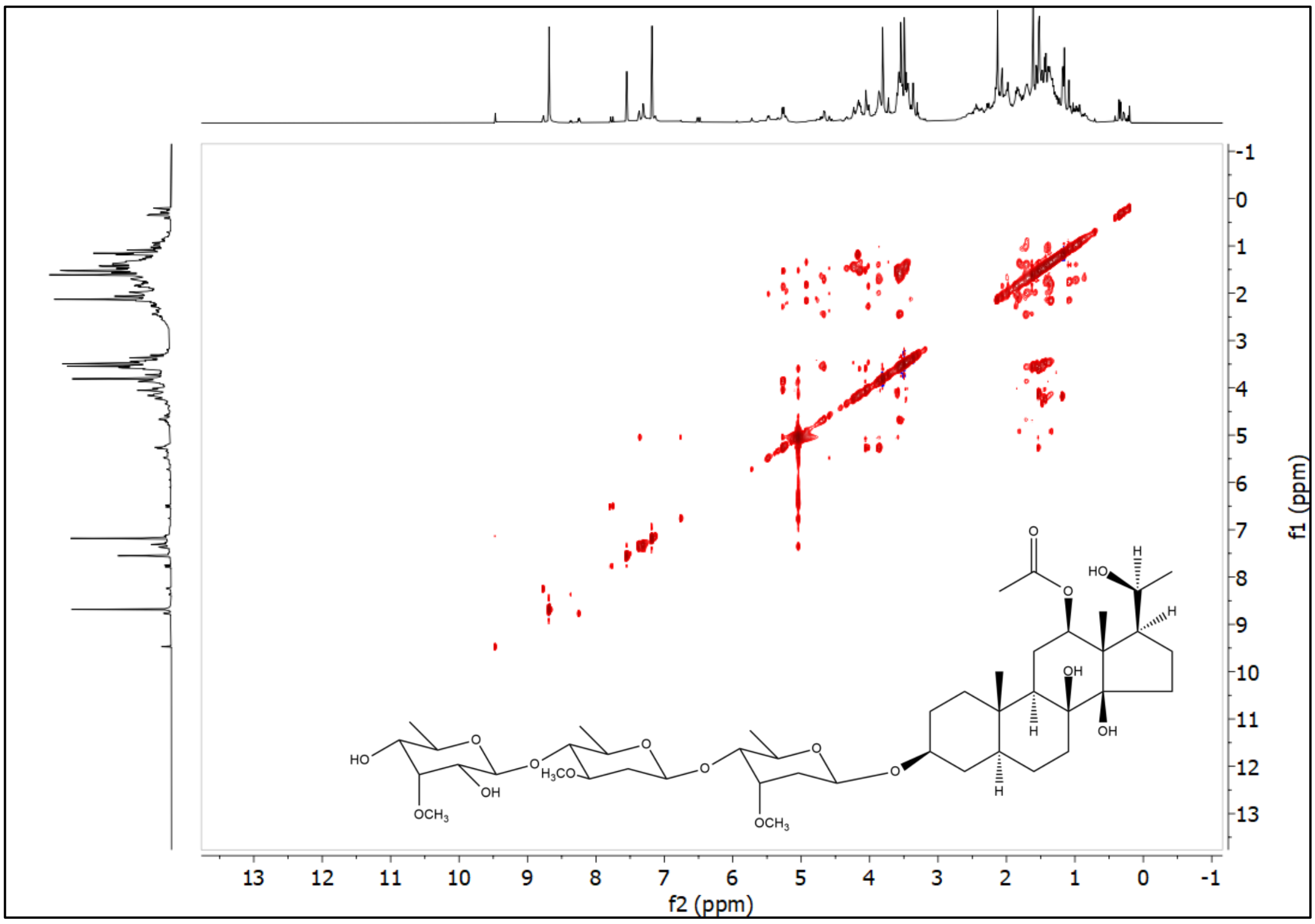

Figure S160. NOESY spectrum (500 x 500 MHz, C_5D_5N) of compound 16.

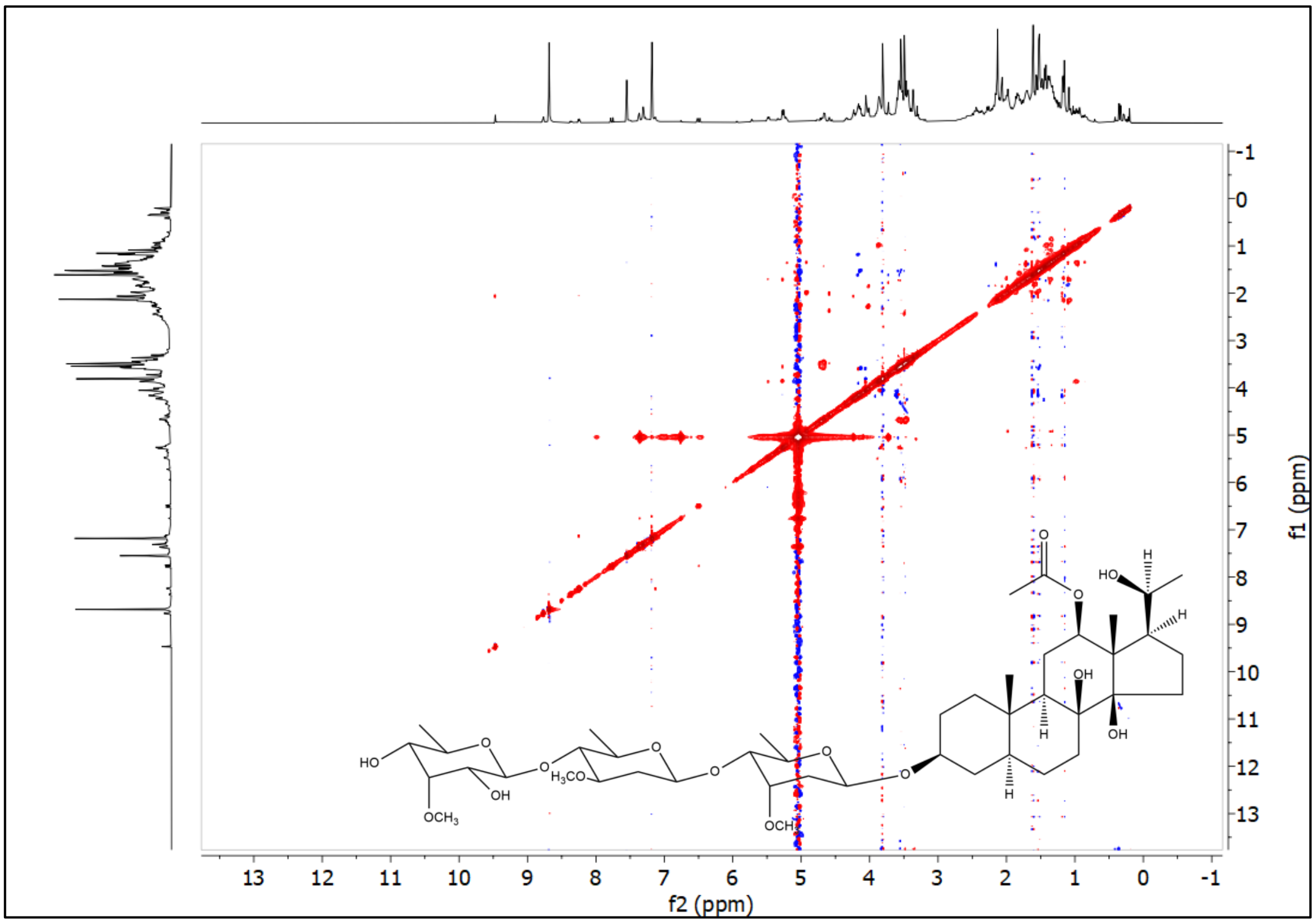

Figure S161. ATR-Infrared spectrum of compound 17.

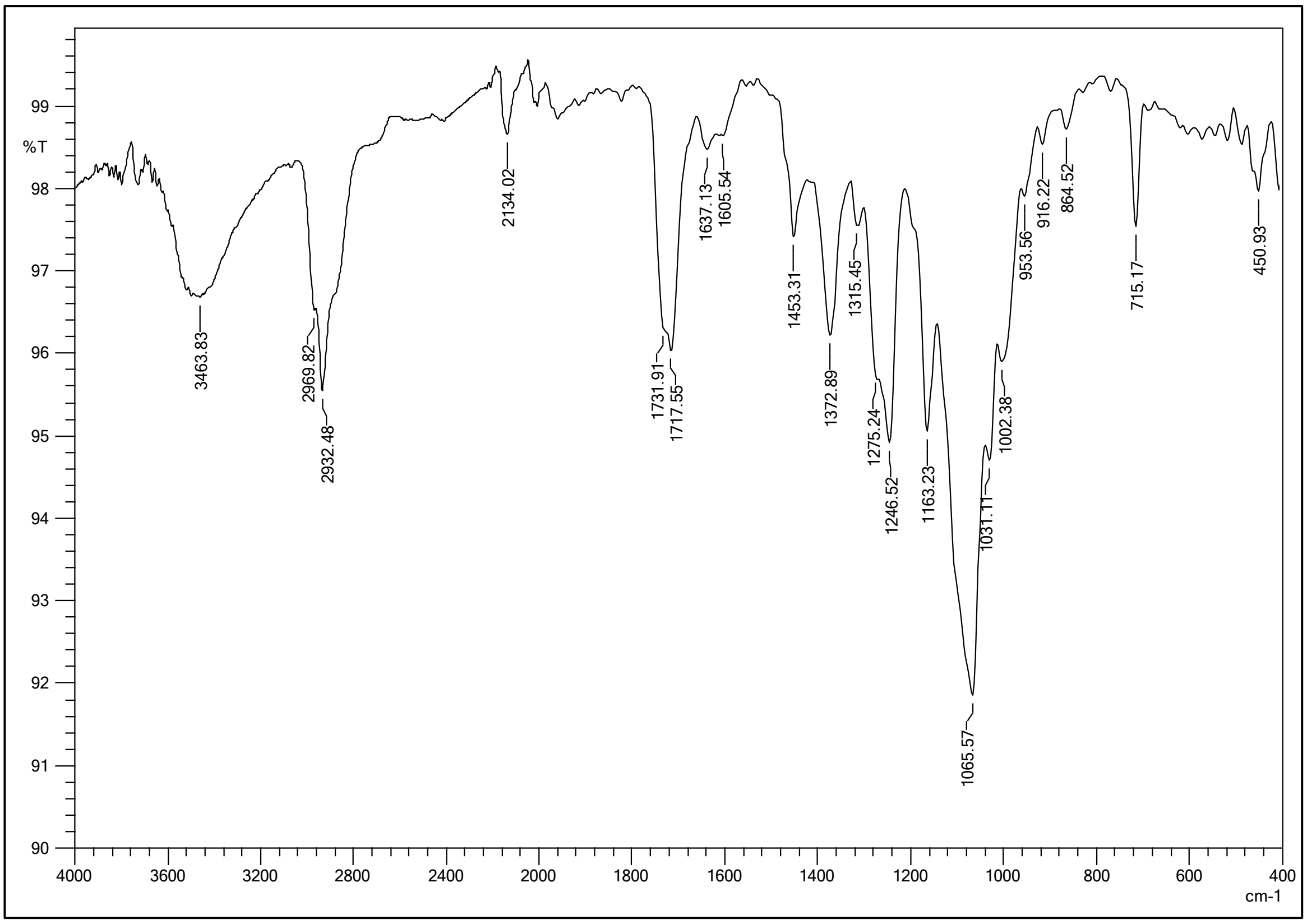

Figure S162. HR-ESI-MS mass spectrum of compound 17.

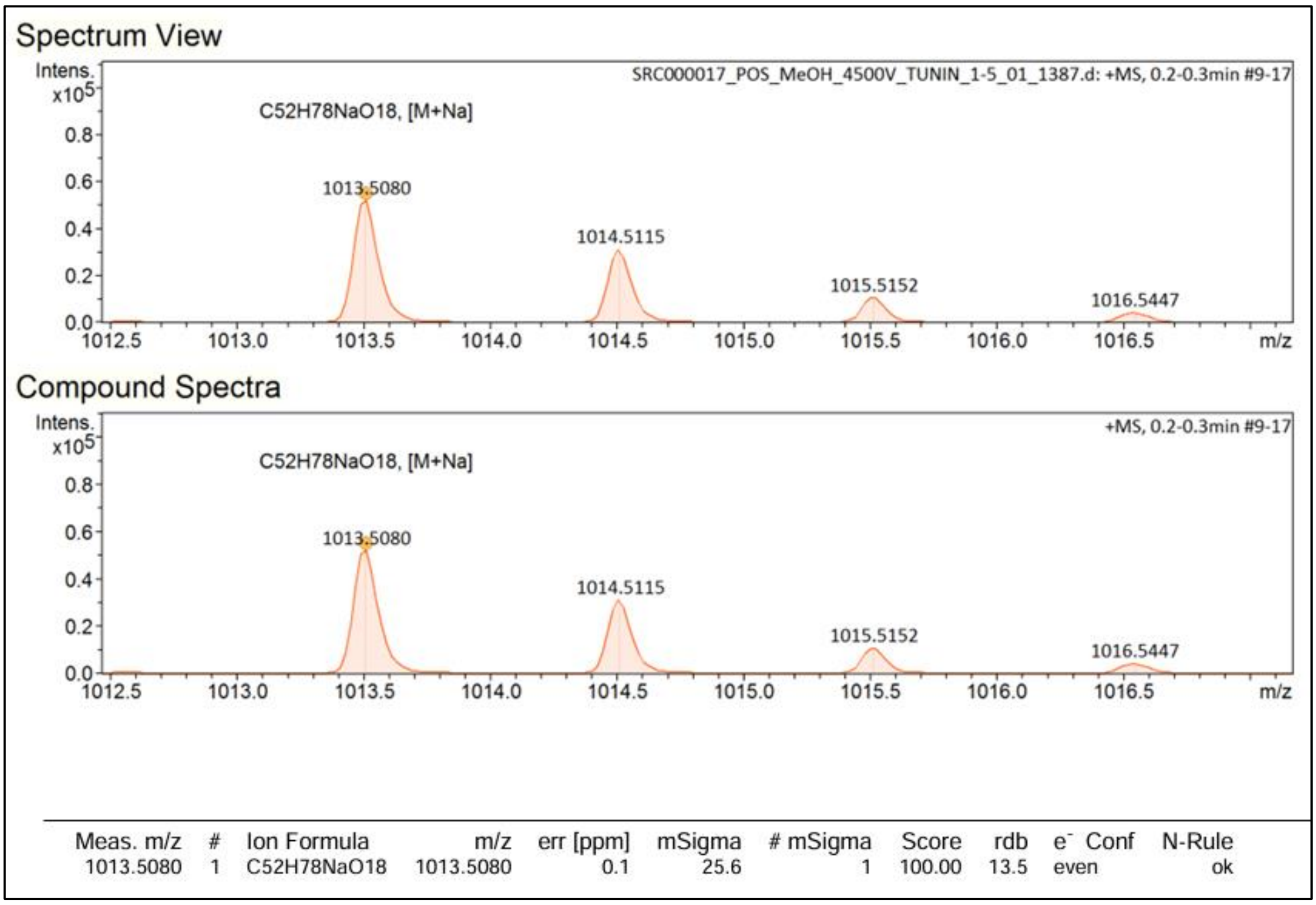

Figure S163. ^{1}H NMR spectrum (500 MHz, C_5D_5N) of compound 17

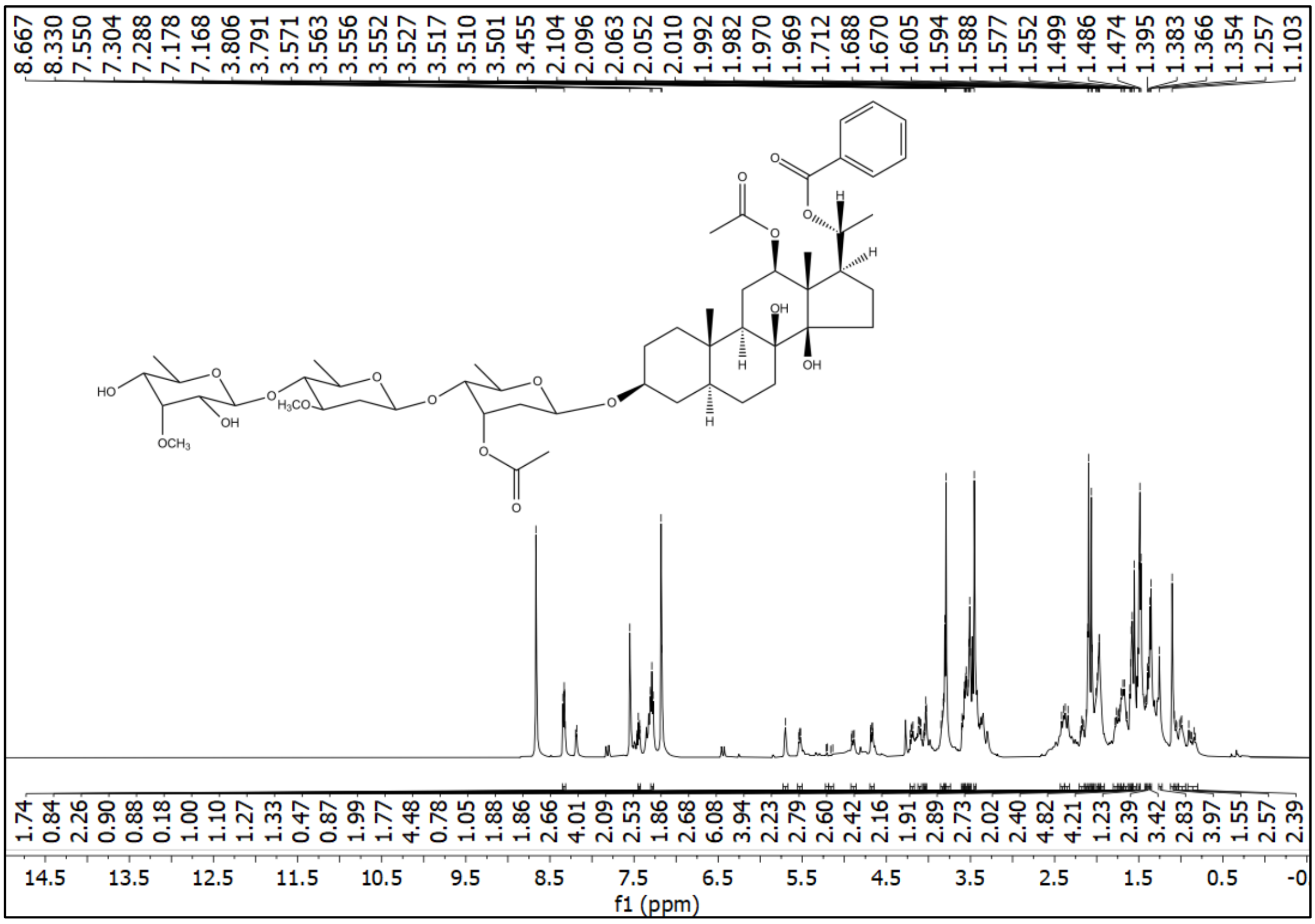

Figure S164. ^{13}C-BB NMR spectrum (125 MHz, C_5D_5N) of compound 17

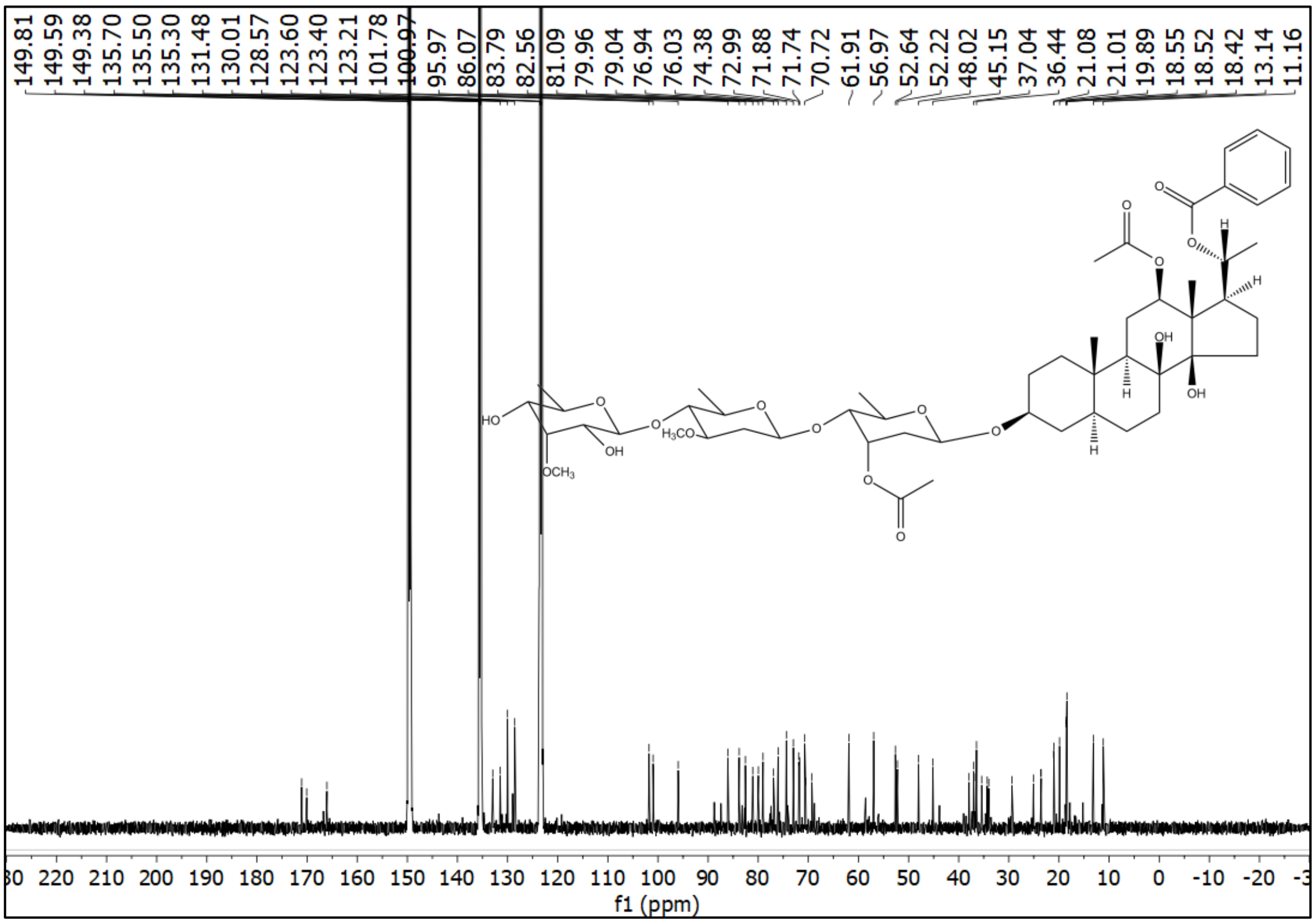

Figure S165. ^{13}C-DEPT135 NMR spectrum (125 MHz, C_5D_5N) of compound 17

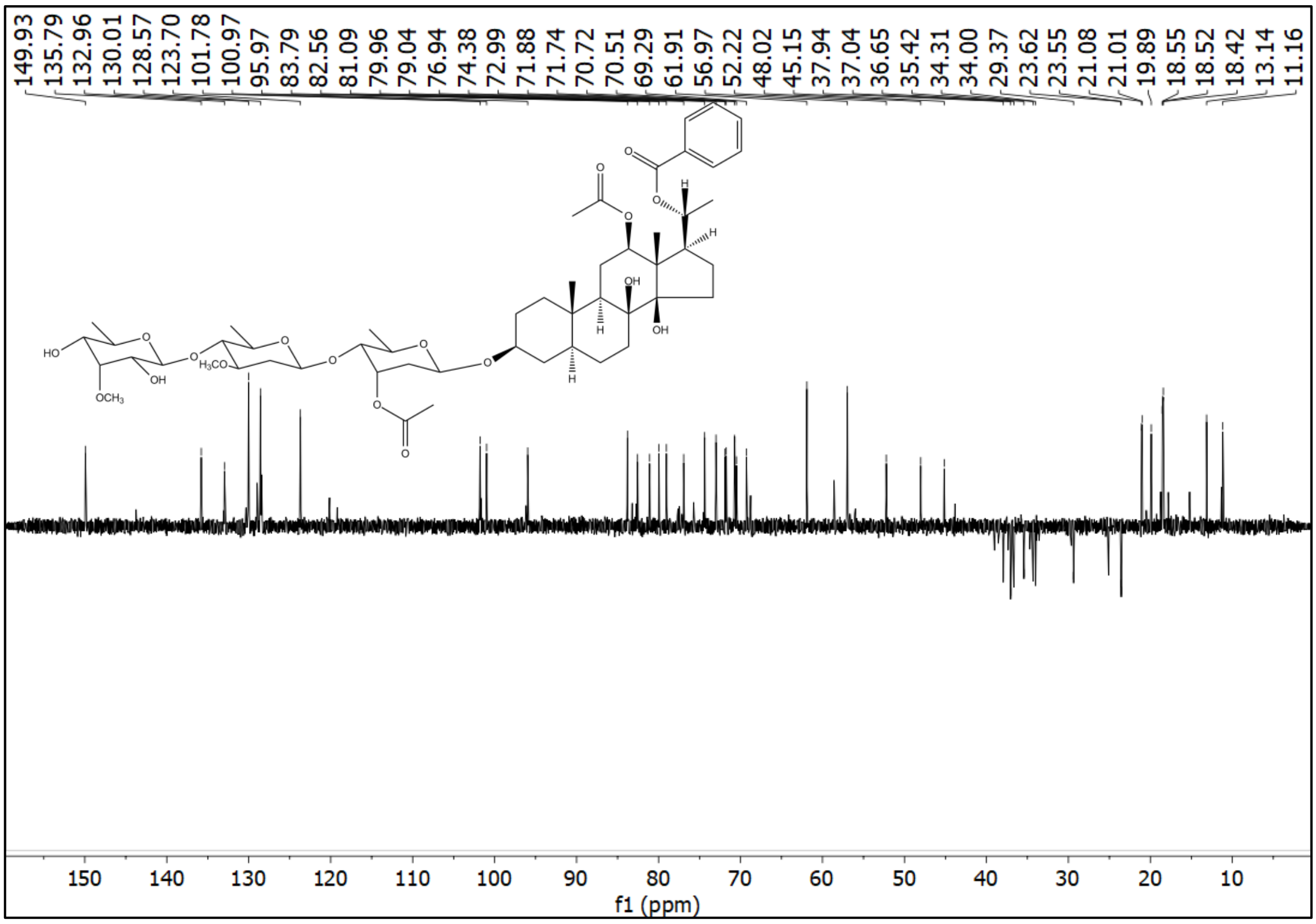

Figure S166. HSQC spectrum (500 x 125 MHz, C_5D_5N) of compound 17

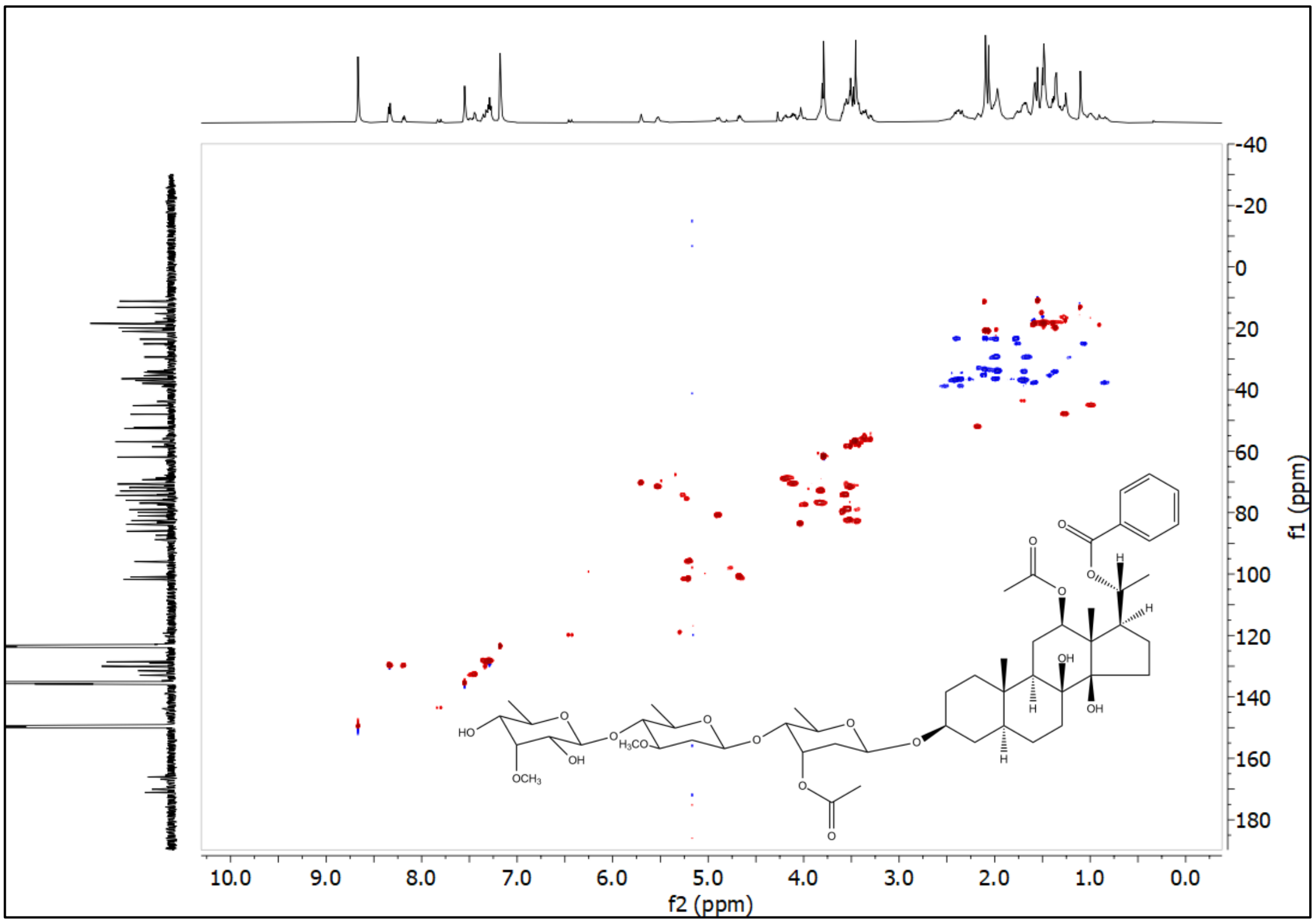

Figure S167. HMBC spectrum (500 x 125 MHz, C_5D_5N) of compound 17

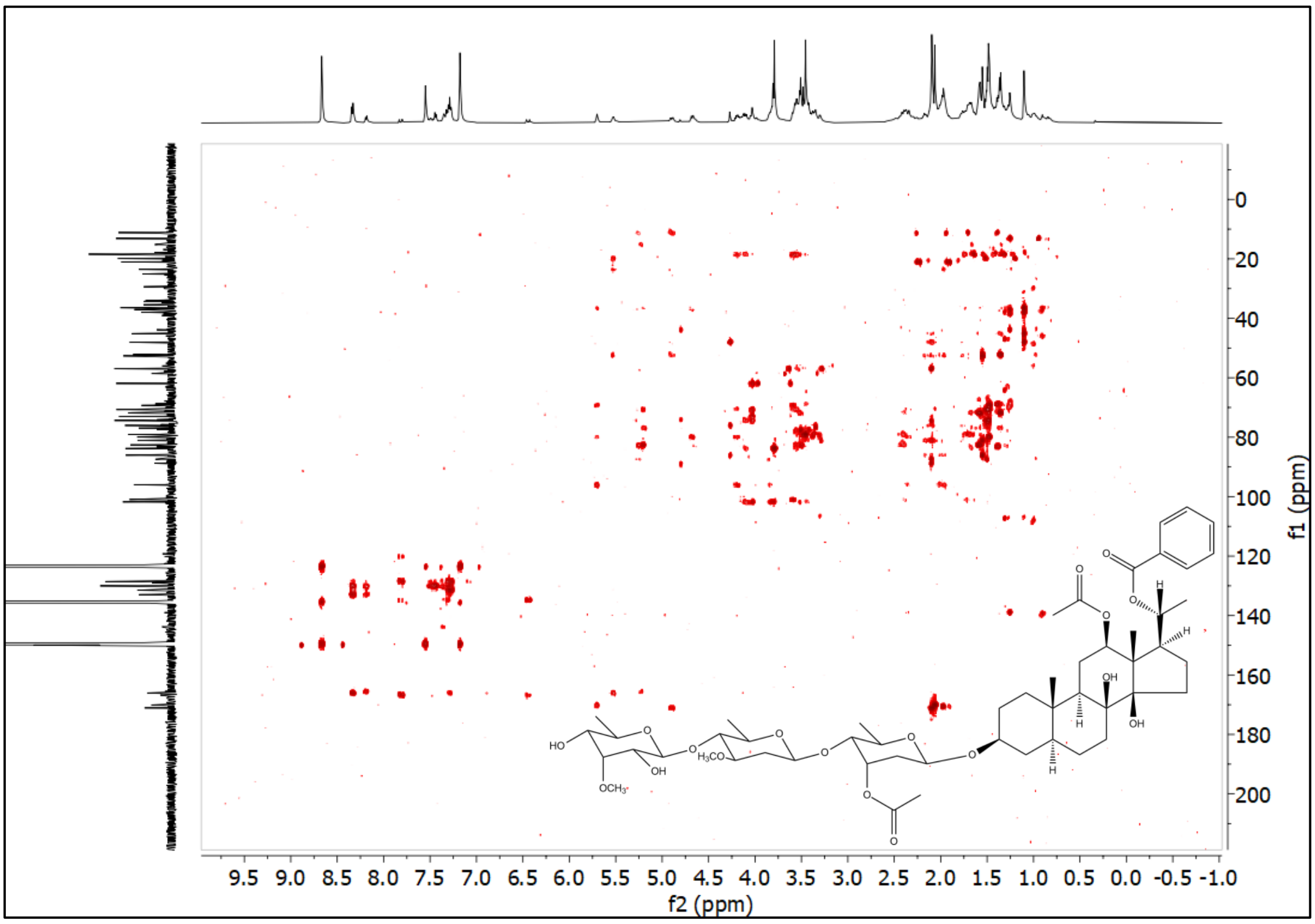

Figure S168. TOCSY spectrum (500 x 500 MHz, C_5D_5N) of compound 17

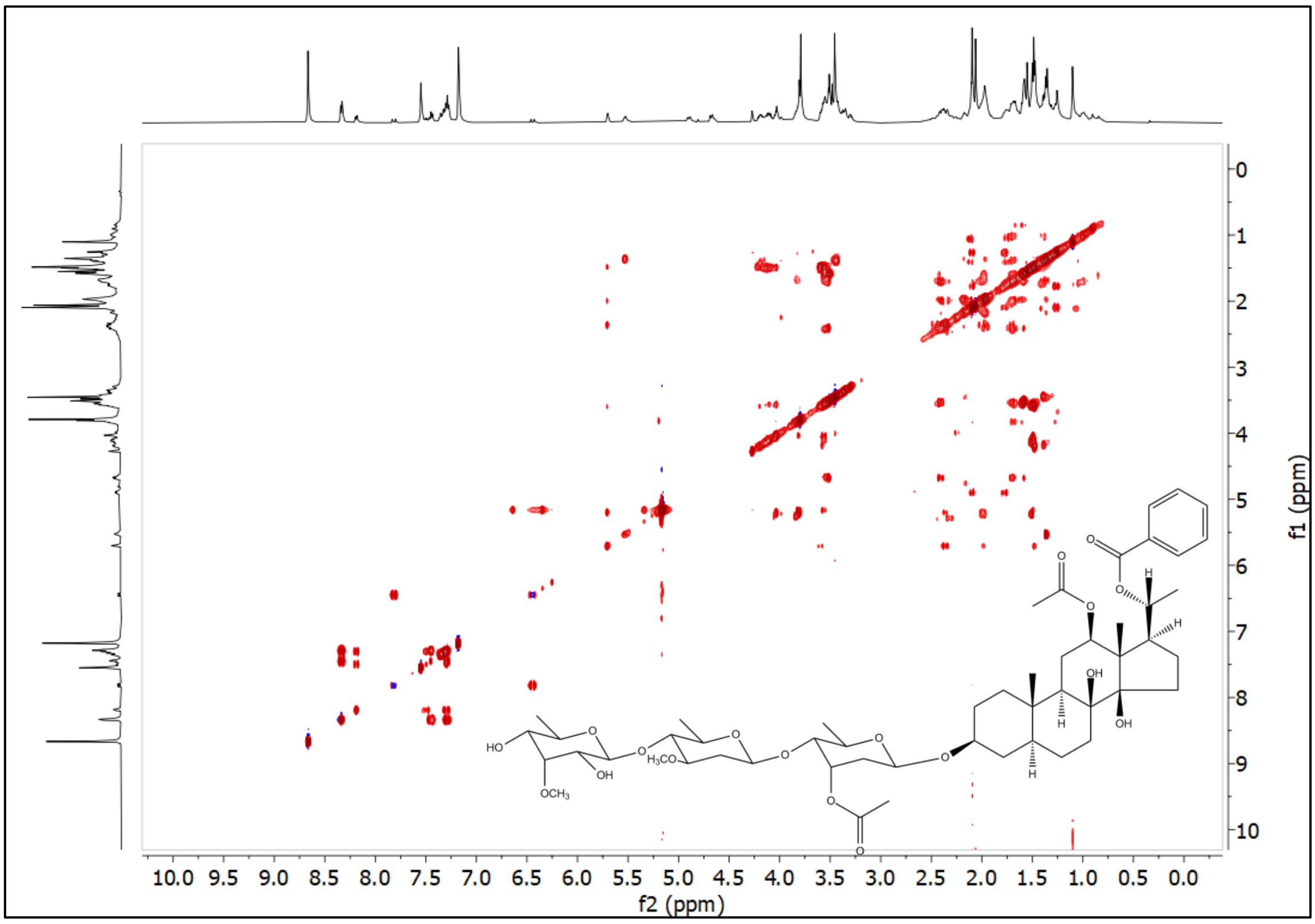

Figure S169. ROESY spectrum (500 x 500 MHz, C_5D_5N) of compound 17.

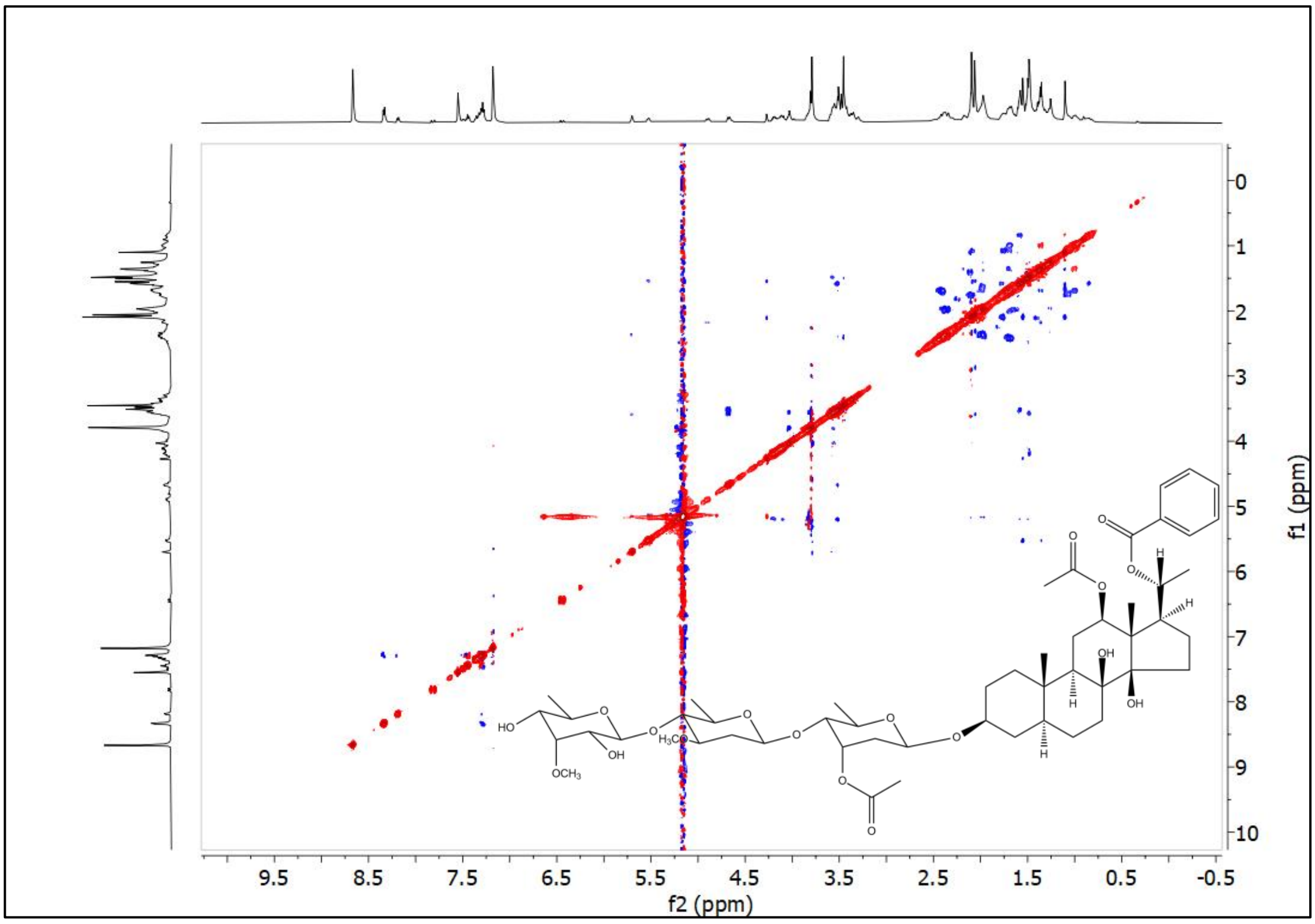

Figure S170. ATR-Infrared spectrum of compound 18.

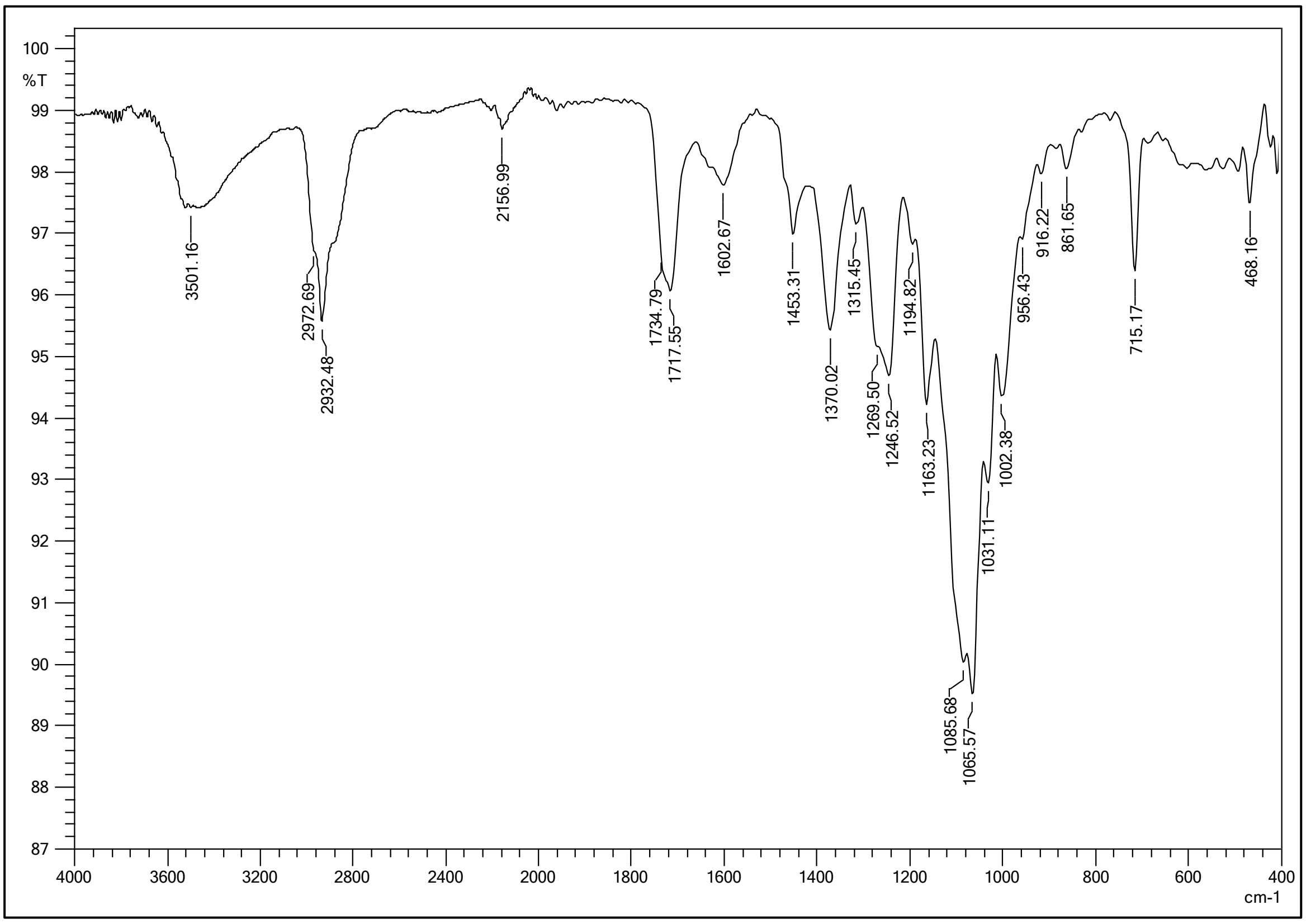

Figure S171. HR-ESI-MS mass spectrum of compound 18.

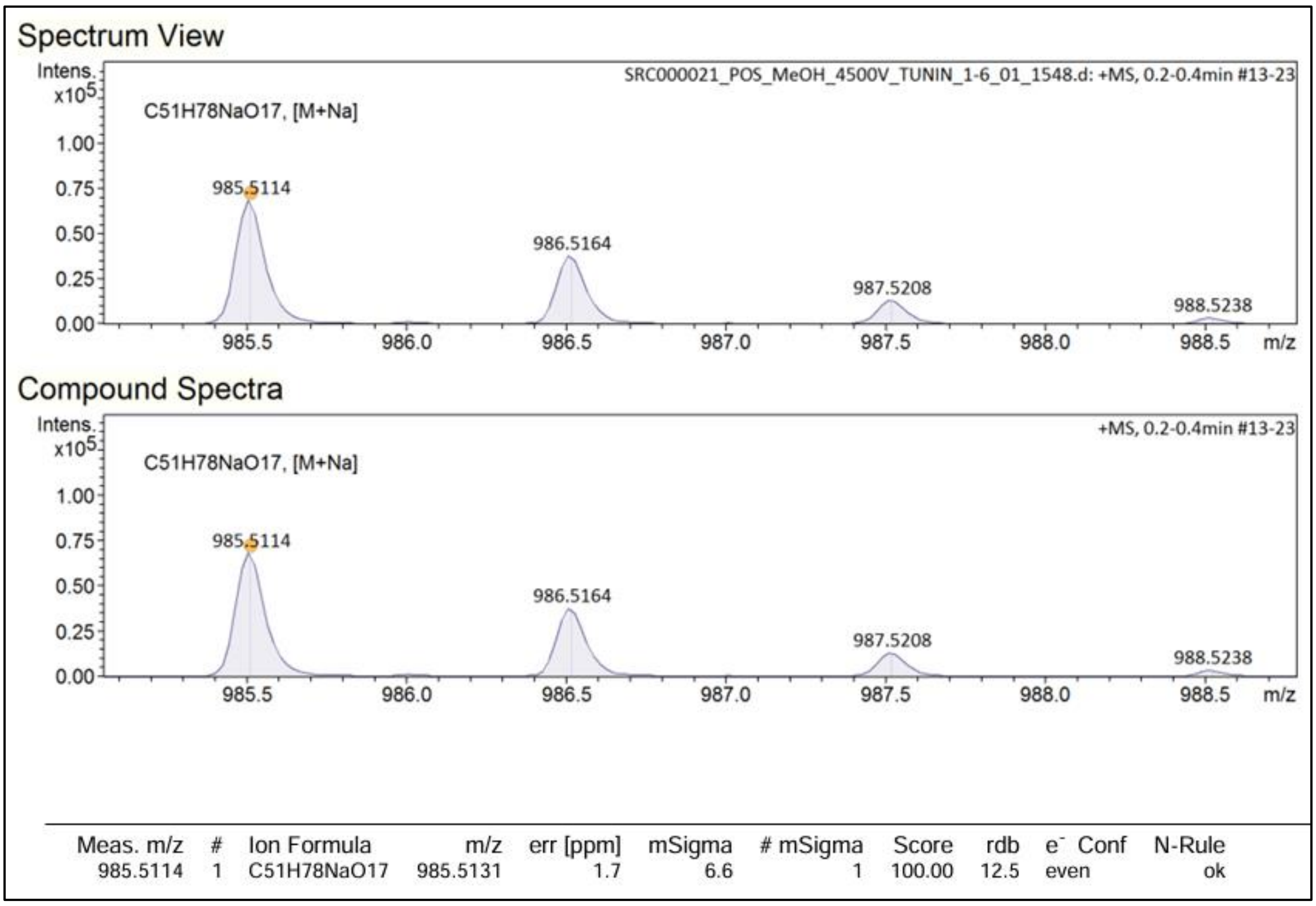

Figure S172. ^{1}H NMR spectrum (400 MHz, C_5D_5N) of compound 18

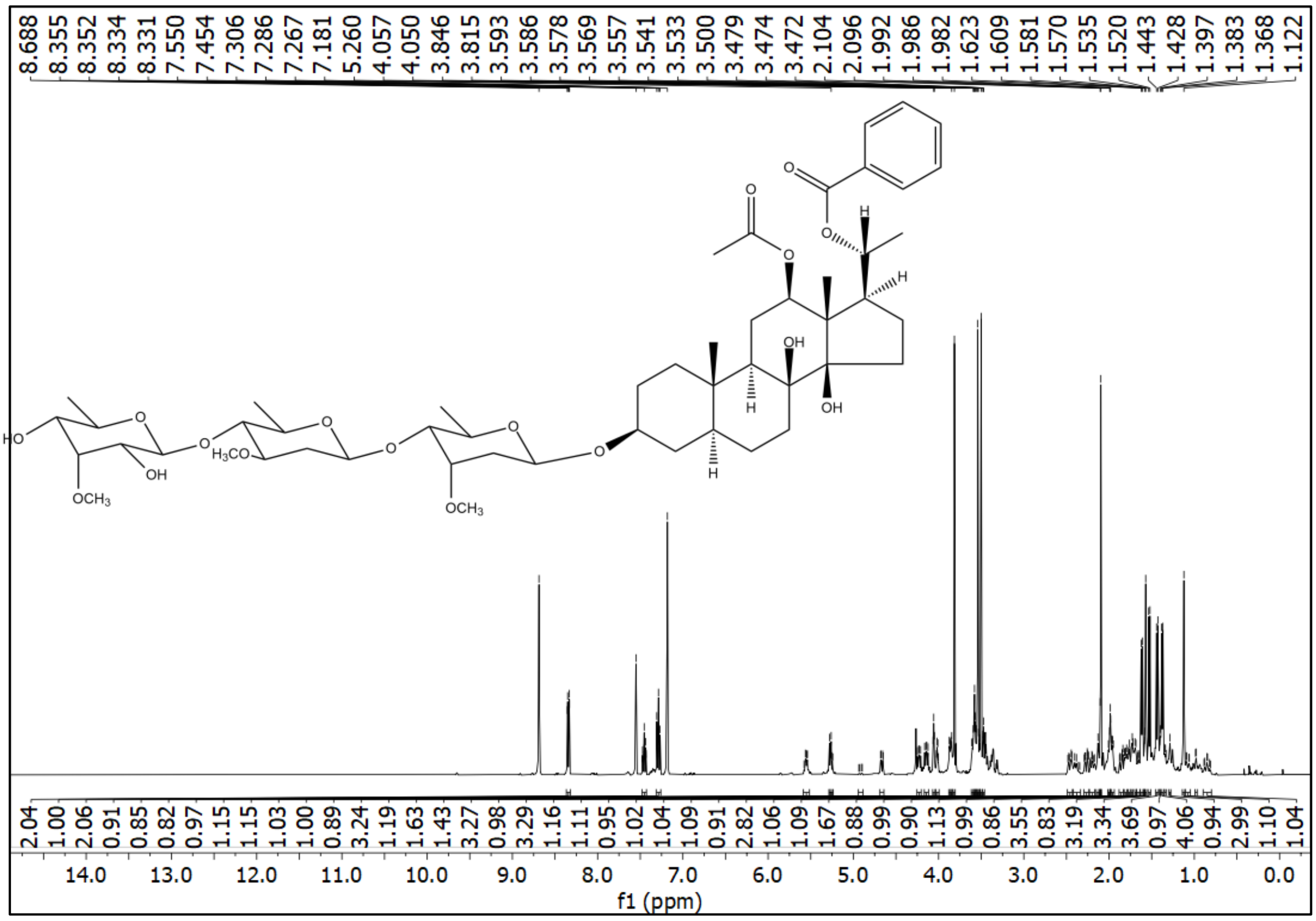

Figure S173. ^{13}C-BB NMR spectrum (100 MHz, C_5D_5N) of compound 18

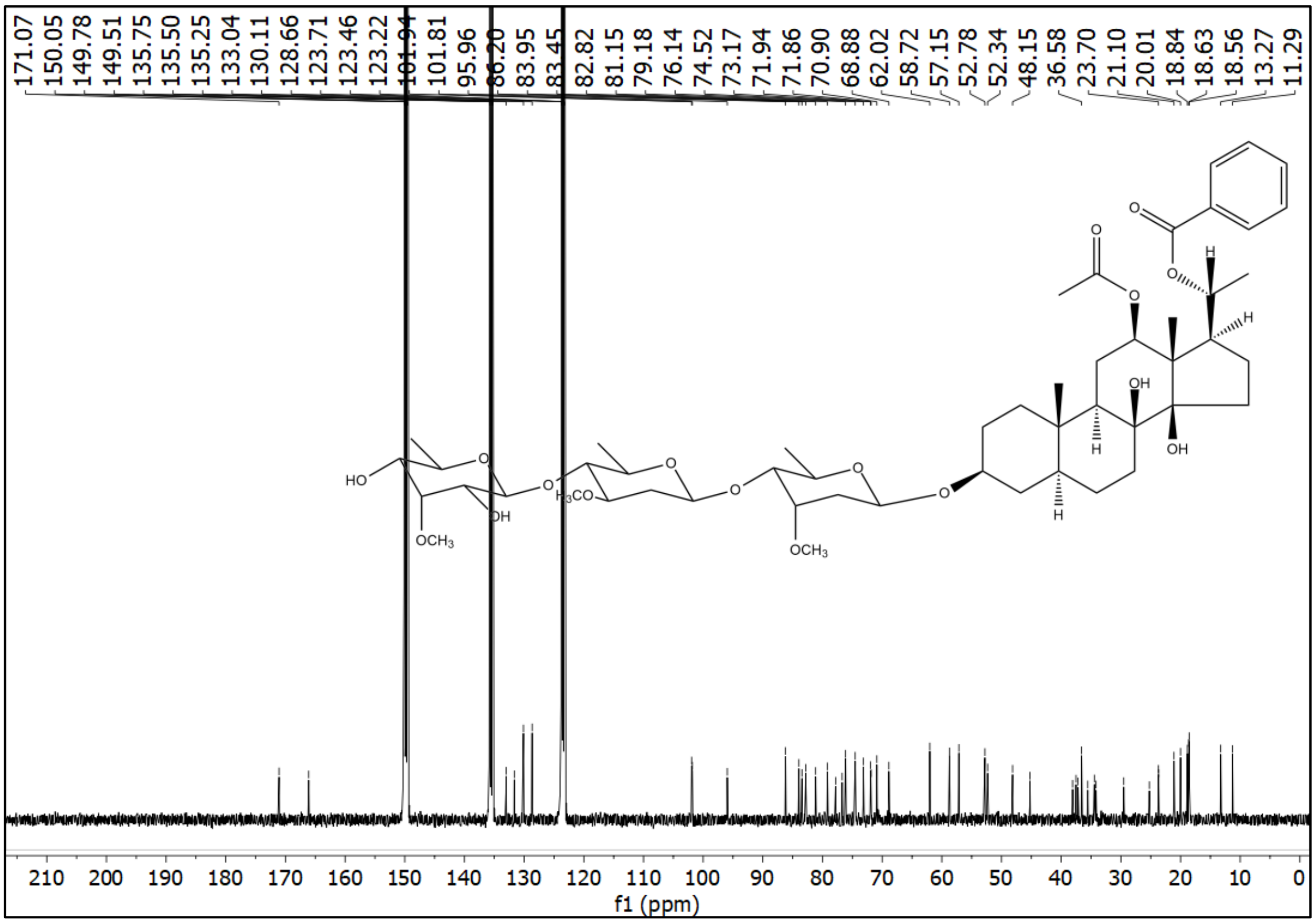

Figure S174. ^{13}C-DEPT135 NMR spectrum (100 MHz, C_5D_5N) of compound 18

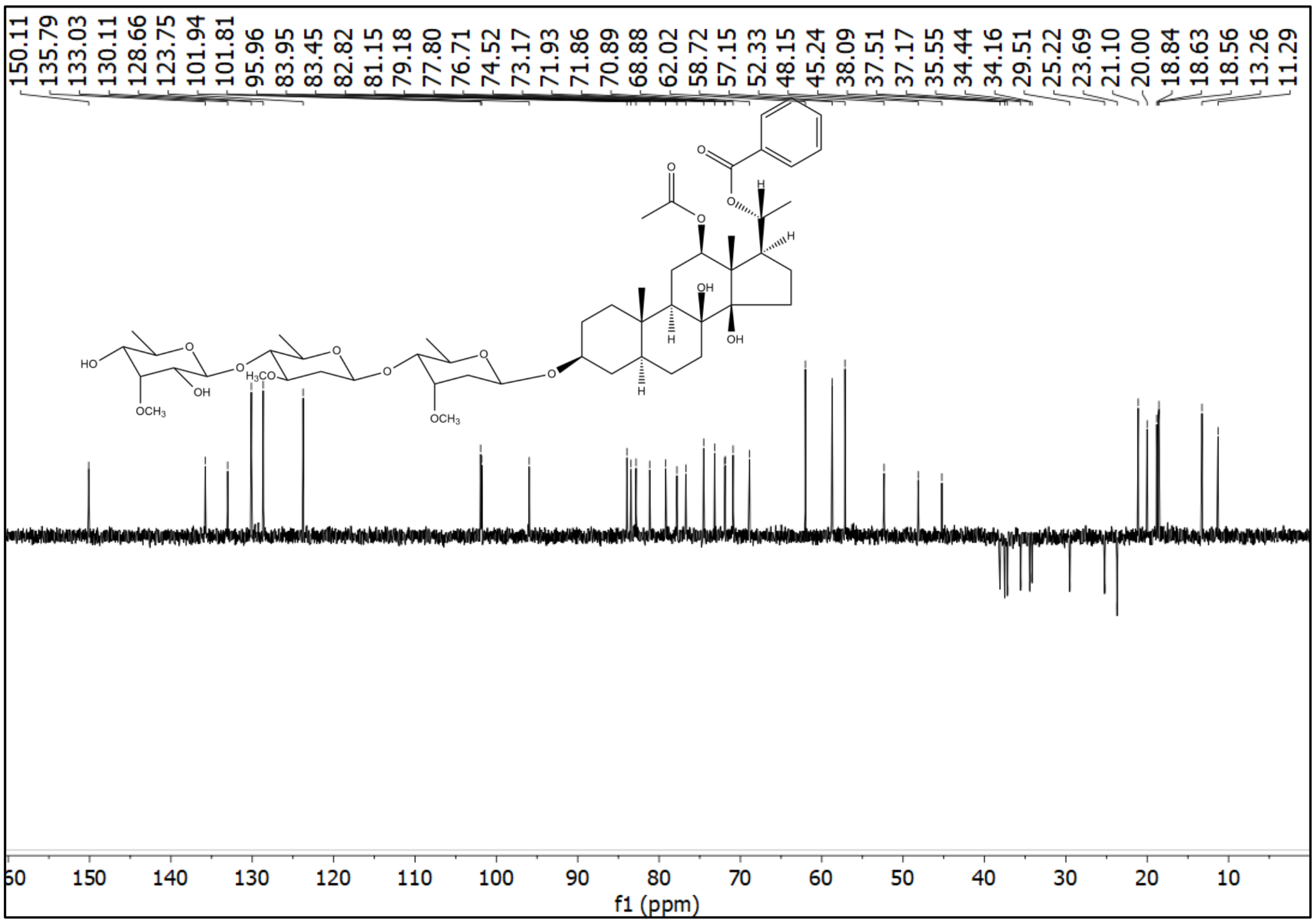

Figure S175. HSQC spectrum (400 x 100 MHz, C_5D_5N) of compound 18

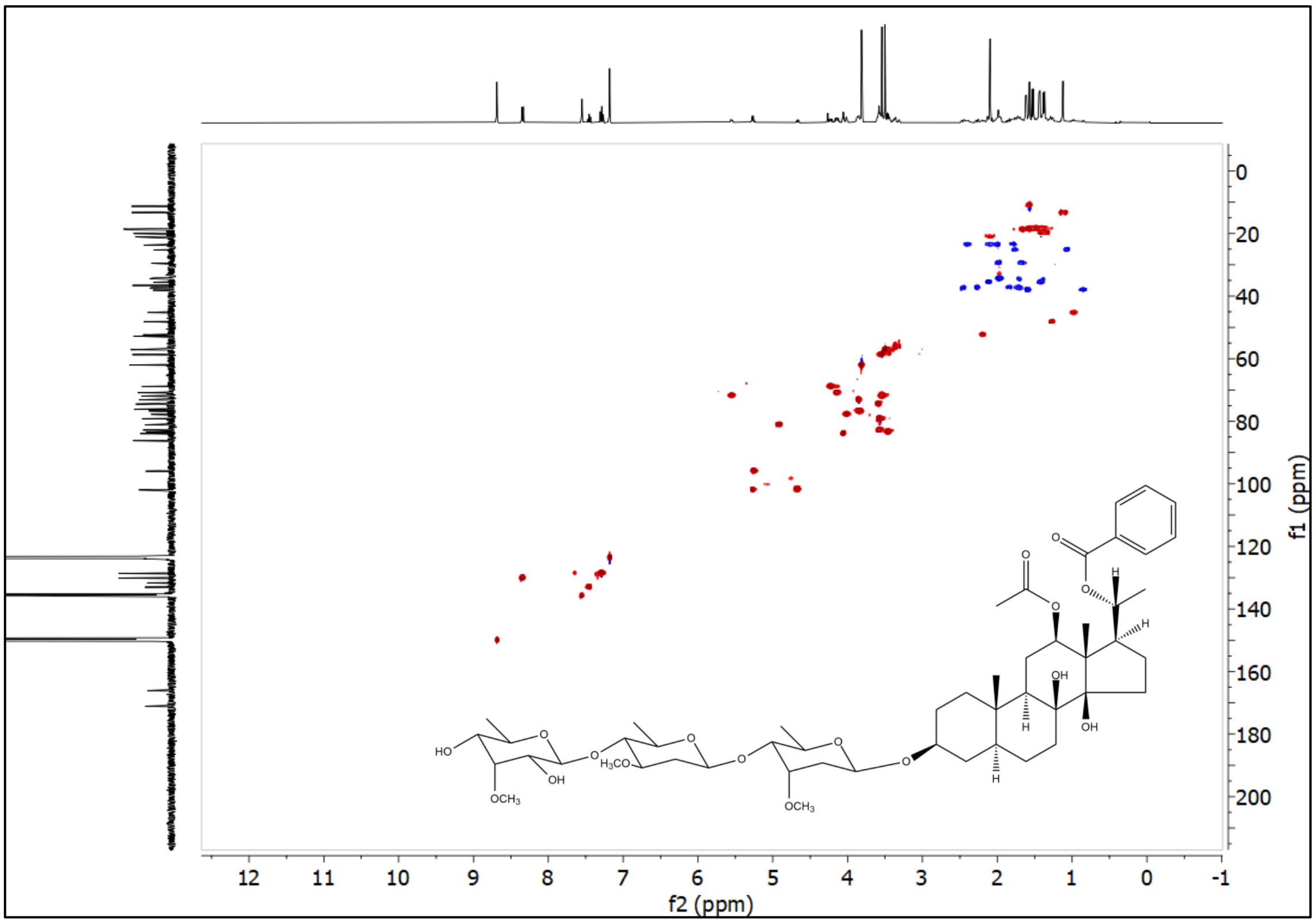

Figure S176. HMBC spectrum (400 x 100 MHz, C_5D_5N) of compound 18

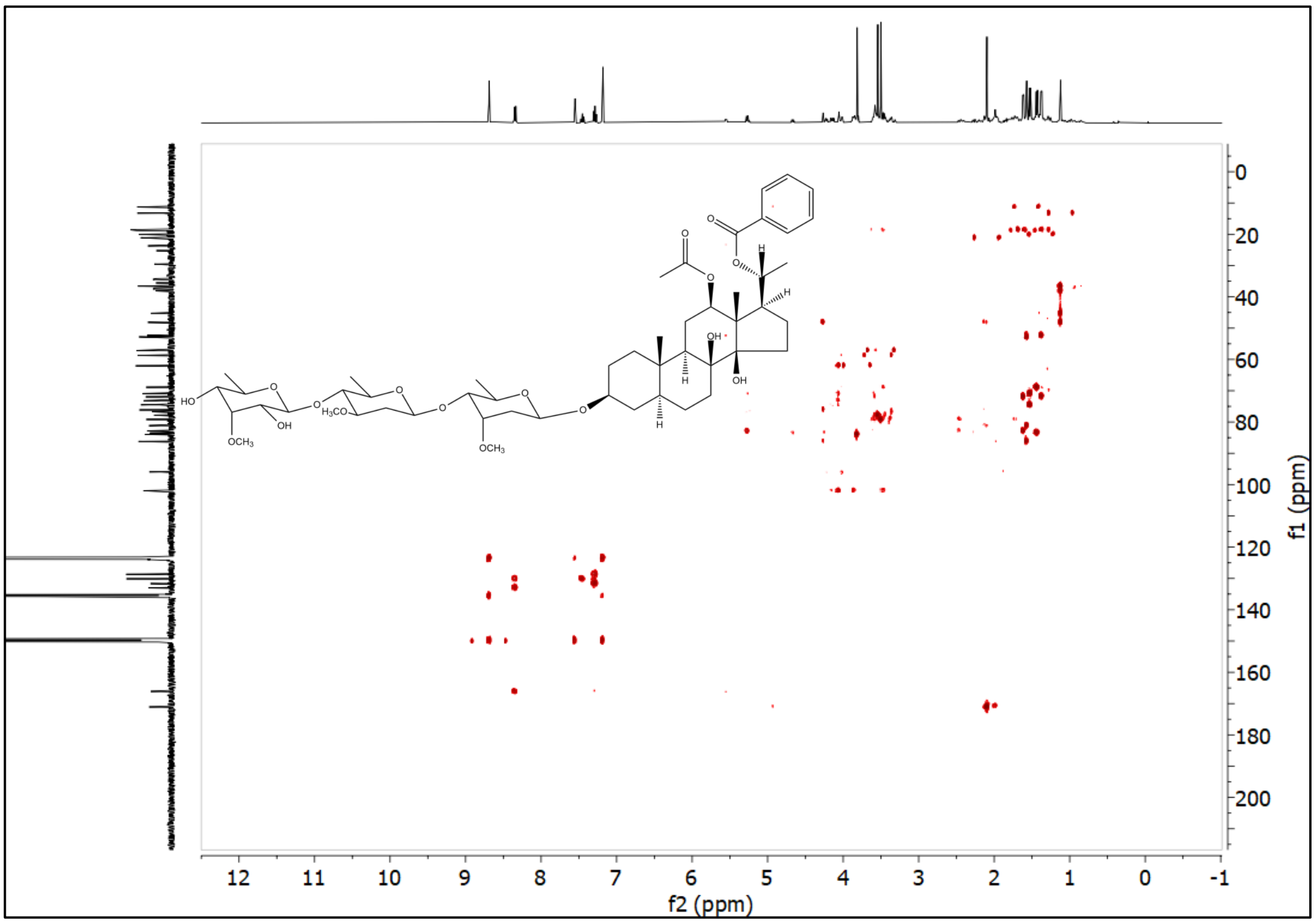

Figure S177. COSY spectrum (400 x 400 MHz, C_5D_5N) of compound 18.

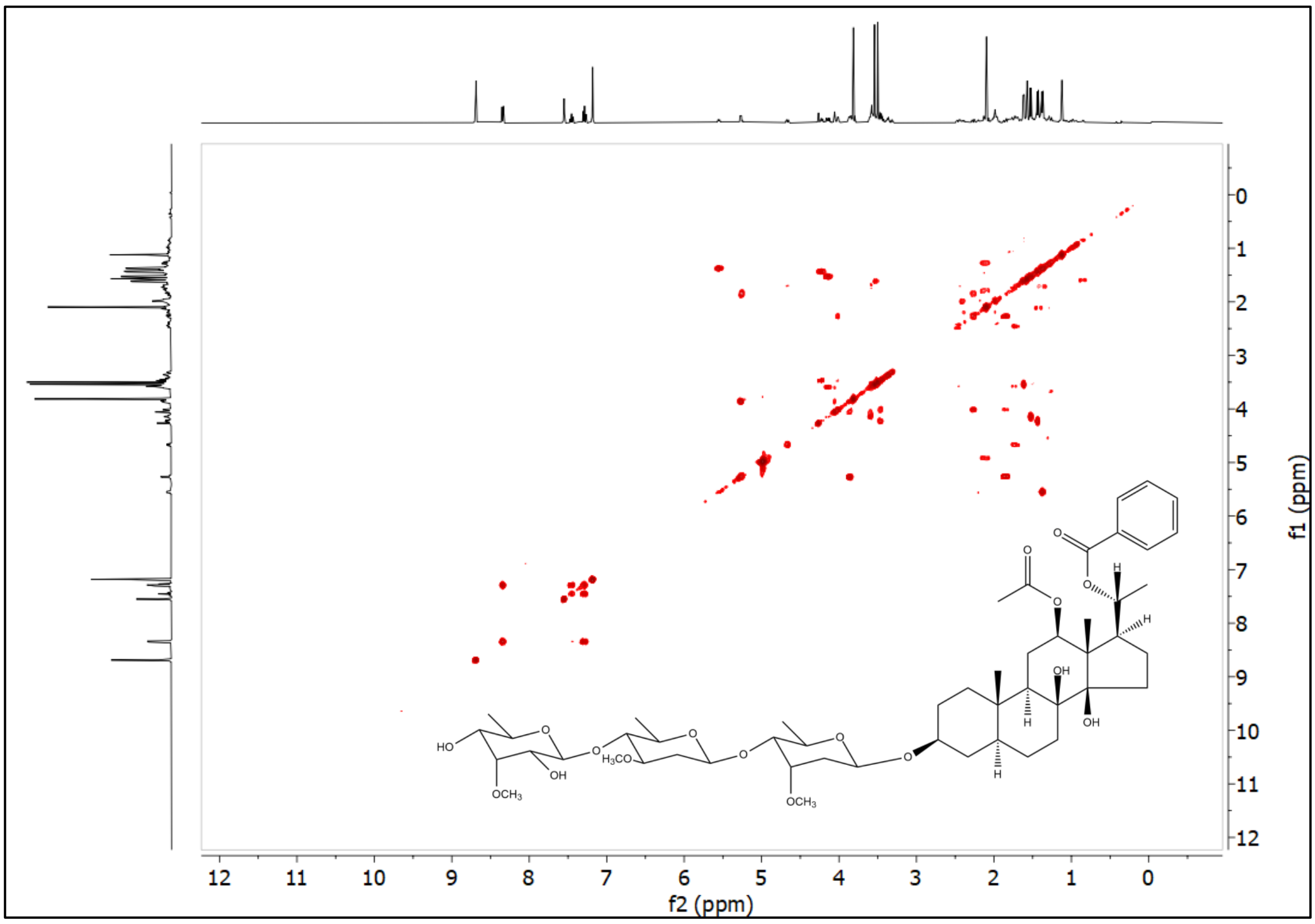

Figure S178. TOCSY spectrum (400 x 400 MHz, C_5D_5N) of compound 18.

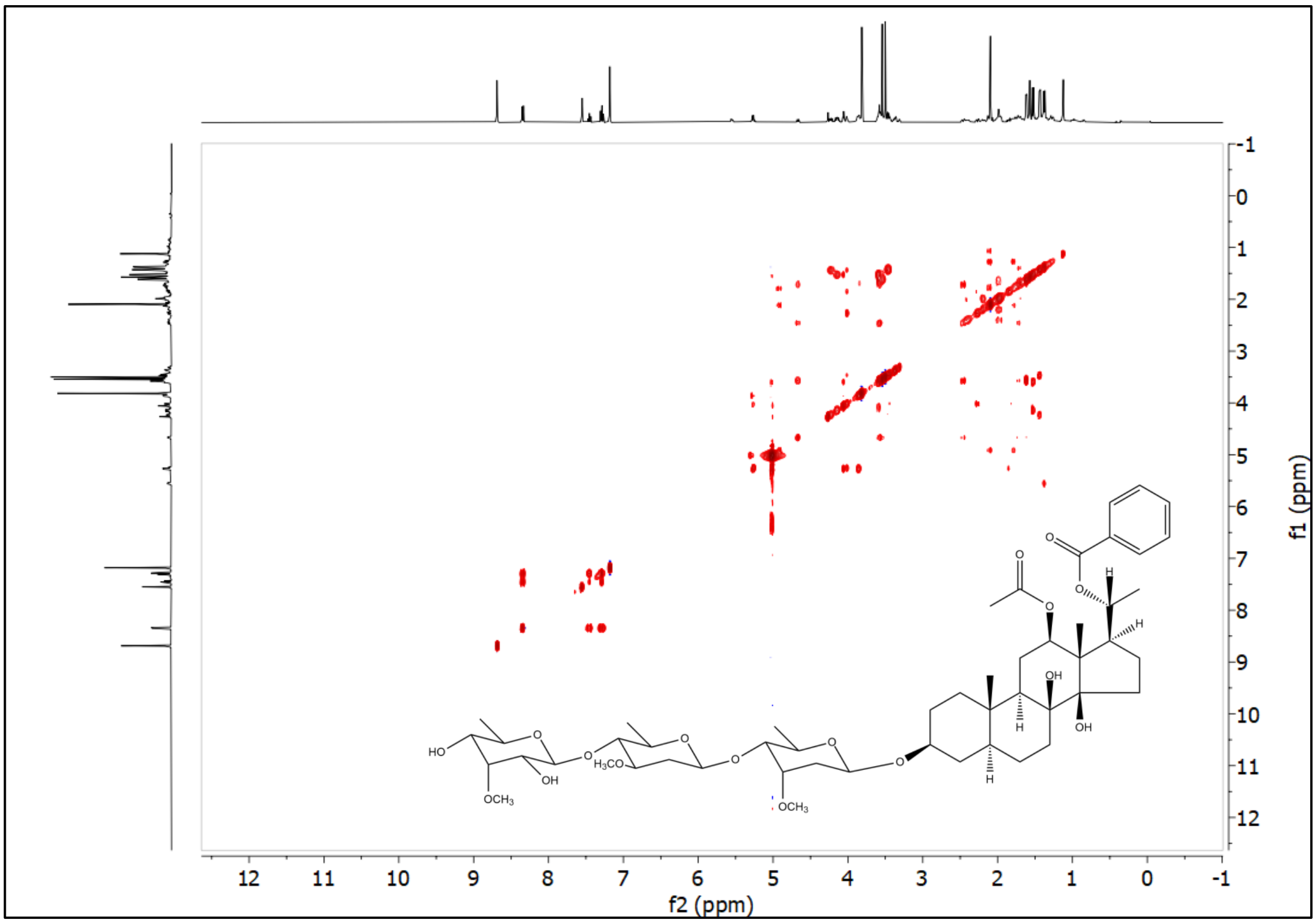

Figure S179. ROESY spectrum (400 x 400 MHz, C_5D_5N) of compound 18.

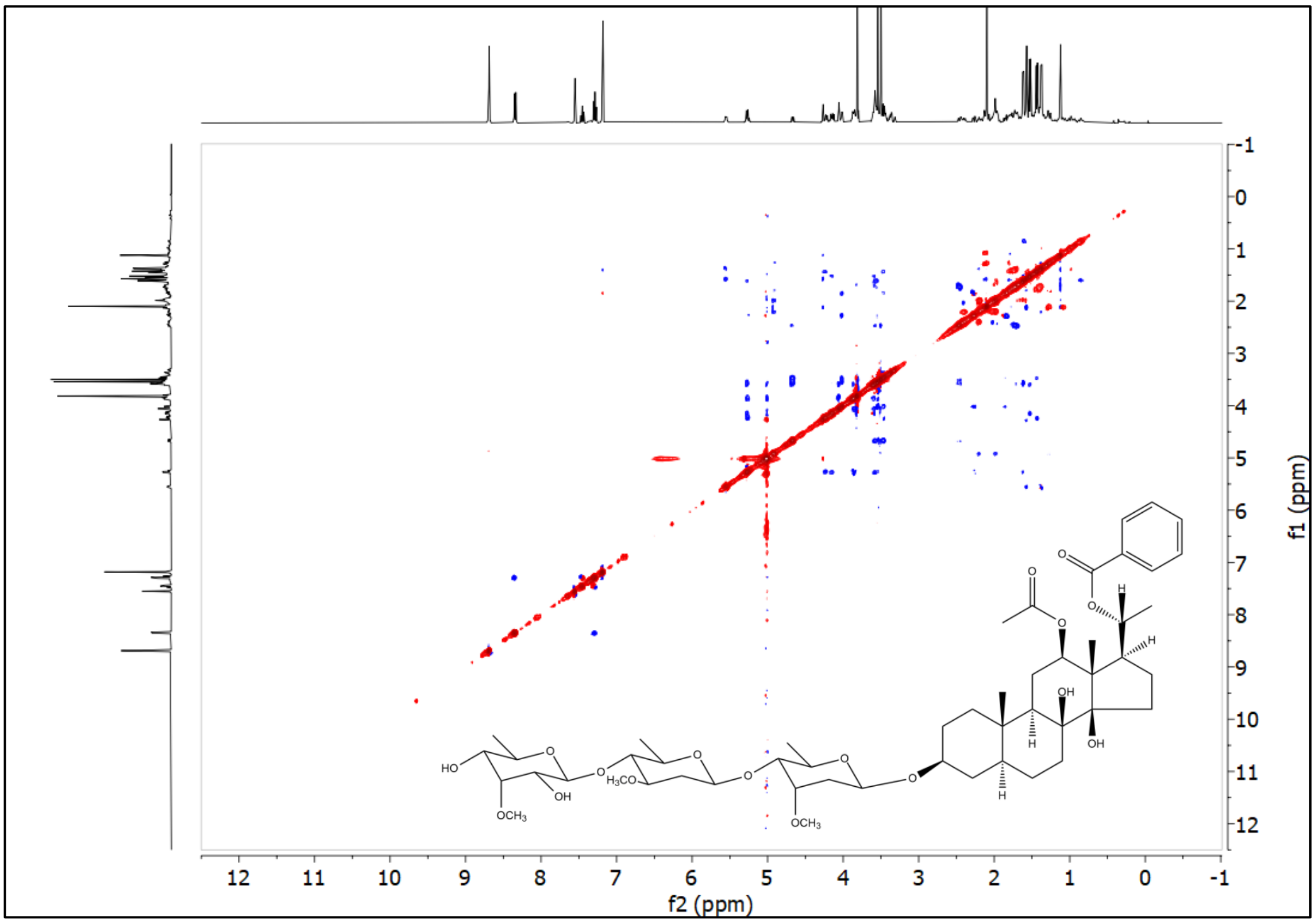

Figure S180. Effect of dimethyl sulfoxide (DMSO) concentration on nitric oxide (NO) production in LPS-stimulated J774.A1 macrophages. This control experiment demonstrates that the DMSO concentration used as the vehicle in the biological assays did not significantly affect NO production.

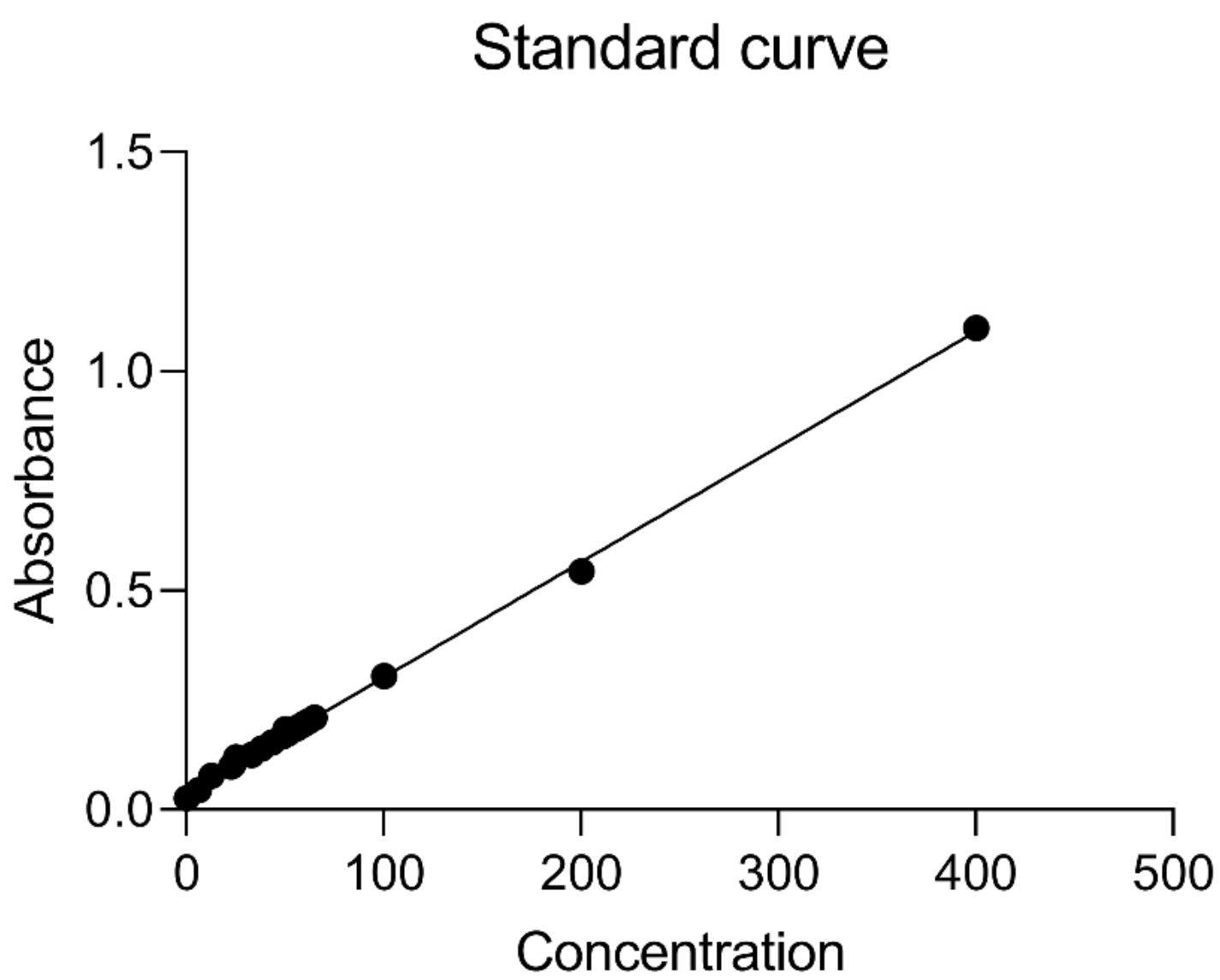

Supplement: Supplementary file 1 [file ao6c02994_si_001.pdf]
